# Altered metabolic gene expression in the brain of a triprolyl-human amylin transgenic mouse model of type 2 diabetes

**DOI:** 10.1038/s41598-019-51088-x

**Published:** 2019-10-10

**Authors:** Tina Nie, Shaoping Zhang, Greeshma Vazhoor Amarsingh, Hong Liu, Mark J. McCann, Garth J. S. Cooper

**Affiliations:** 1 School of Biological Sciences, Faculty of Science, the University of Auckland, Private Bag 92019, Auckland, 1142 New Zealand; 2 Food Nutrition & Health Team, AgResearch Ltd, Grasslands Research Centre, Palmerston North, 4442 New Zealand; 3 The Maurice Wilkins Centre for Molecular Biodiscovery, Faculty of Science, the University of Auckland, Auckland, New Zealand; 4 Centre for Advanced Discovery and Experimental Therapeutics, Division of Cardiovascular Sciences, Faculty of Biology Medicine & Health, School of Medical Sciences, the University of Manchester, Manchester, M13 9NT United Kingdom

**Keywords:** Type 2 diabetes, Experimental models of disease

## Abstract

Type 2 diabetes mellitus is a major health concern worldwide; however, the molecular mechanism underlying its development is poorly understood. The hormone amylin is postulated to be involved, as human amylin forms amyloid in the pancreases of diabetic patients, and oligomers have been shown to be cytotoxic to β-cells. As rodent amylin is non-amyloidogenic, mice expressing human amylin have been developed to investigate this hypothesis. However, it is not possible to differentiate the effects of amylin overexpression from β-cell loss in these models. We have developed transgenic mice that overexpress [^25, 28, 29^ triprolyl]human amylin, a non-amyloidogenic variant of amylin, designated the Line 44 model. This model allows us to investigate the effects of chronic overexpression of non-cytotoxic amylin. We characterised this model and found it developed obesity, hyperglycaemia and hyperinsulinaemia. This phenotype was associated with alterations in the expression of genes involved in the amylin, insulin and leptin signalling pathways within the brain. This included genes such as *c-Fos* (a marker of amylin activation); *Socs3* (a leptin inhibitor); and *Cart*, *Pomc* and *Npy* (neuropeptides that control appetite). We also examined Socs3 protein expression and phosphorylated Stat3 to determine if changes at the mRNA level would be reflected at the protein level.

## Introduction

Type 2 diabetes mellitus (T2DM) is widely recognised as one of the biggest health issues being faced by the world today. It is estimated over 415 million adults have diabetes, with this number expected to rise to 642 million by 2040^1^. Around 87–91% of these individuals have type 2 diabetes. T2DM is characterised by hyperglycaemia, insulin resistance, amyloid formation in the pancreatic islets and β-cell degeneration^2^. The molecular basis of T2DM remains obscure. There is need for a greater understanding of the causative mechanism underlying the development of insulin resistance, as well as new models in which to study this, without which developing efficacious new therapies is difficult.

Amylin (also known as islet amyloid polypeptide, IAPP) is a pancreatic hormone co-secreted with insulin from islet β-cells^3^. It acts on multiple tissues, with one of its major sites of action being the area postrema in the hindbrain^4^. From there, the neural signal is transmitted to the hypothalamus, the body’s major control centre for regulating appetite and energy homeostasis, where it acts to suppress food intake^5^. Amylin is also reported to have weight-lowering effects beyond its anorectic effect, although this has been less well-studied^6,7^. Amylin also functions as a non-competitive insulin antagonist in skeletal muscle and may play a role in the development of peripheral insulin resistance^8,9^. Islet amyloid, which consists primarily of amylin fibrils, is commonly found in the pancreas of diabetic patients^10,11^, and is associated with β-cell dysfunction and apoptosis^12–16^. These findings have led to the hypothesis that excessive expression of amylin may be one of the causative mechanisms of T2DM. The human amylin (hA) peptide contains an amyloidogenic sequence between residues 20 and 29^17^, which causes hA to spontaneously form insoluble aggregates in solution^18^. Rodent amylin, which has five amino acid substitutions in this region, is non-aggregating^17^. As hA forms amyloid but rodent amylin does not, transgenic mouse models which overexpress wild-type hA have been developed^19–24^. Some of these models develop hyperglycaemia, with penetrance and severity being gene dose-dependent, as well as β-cell loss and decreased insulin secretion. However, as amylin oligomers are cytotoxic to β-cells^13,15^, this self-limits the secretion of amylin and also curtails insulin production in human-amylin mice^23^. As such, it is difficult to differentiate the effects of hyperamylinaemia versus β-cell dysfunction.

In order to more clearly distinguish the pathophysiologic effect of amylin overexpression on regulation of metabolism *per se*, we have developed a transgenic mouse model carrying a modified human amylin gene that encodes [^25, 28, 29^ triprolyl]human amylin (tripro-hA). Tripro-hA incorporates three proline residue substitutions at positions 25, 28 and 29, which were derived from the structure of rodent amylin^25^. Tripro-hA does not undergo aggregation or amyloid formation which is cytotoxic^15^. We’ve termed these tripro-hA transgenic mice the Line 44 (L44) model. Tripro-hA has near-identical receptor binding properties to mouse amylin and acts as a full amylin agonist *in vitro* and *in vivo*. This peptide is registered as an adjunct therapy for insulin-requiring diabetes under the generic name pramlintide^25^. While previous studies have informed on the acute effects of amylin, few have investigated the effects of amylin over a longer time-period. Even ‘long-term’ amylin infusion studies may only last days to a few months^6,7,26,27^. This unique mouse model is expected to allow us to investigate, for the first time, the chronic metabolic effects of hyperamylinaemia itself, which precede and may drive amyloid formation^22^.

We studied the L44 model over a period of 400 days. L44 mice display a number of characteristics that closely mirror those of the human obese-diabetic condition. These include increased food intake, obesity, hyperglycaemia, and hyperinsulinaemia. We hypothesised that hyperamylinaemia gives rise to ligand-mediated amylin resistance in the brain, leading to changes in the expression of metabolic genes that drive hyperphagia, subsequent obesity and insulin resistance. We characterised the body weight, blood glucose, serum hormone and brain gene expression profiles in hemizygous (HEM) and homozygous (HOM) mice of this model and compared these to nontransgenic (NON) controls. In addition to amylin, we also examined insulin and leptin, two well-known hormones that are perturbed in the obese-diabetic state. Both insulin and leptin act in the brain to decrease food intake and lower body weight^28–30^. Insulin resistance is a defining characteristic of T2DM and patients with obesity are often leptin resistant. The expression of 43 genes involved in the amylin, insulin and leptin signal transduction pathways (Table 1) were examined in four brain regions of L44 transgenic mice over different disease stages; T1, prediabetic (100 days); T2, diabetes onset (blood glucose >11 mM for three consecutive weekly measurements); and T3, post-diabetic (400 days). Based on the gene expression results, we also investigated the levels of Socs3 protein and Stat3 phosphorylation by Western blotting to determine whether changes at the mRNA level would translate to altered protein levels or activation.

**Table 1:** Genes in the amylin, insulin and leptin signalling pathways targeted by the NanoString nCounter analysis.

Gene Target	
*Adrbk1* (β-adrenergic receptor kinase 1)	*Irs1* (insulin receptor substrate-1)
*Agrp* (agouti-related peptide)	*Irs2* (insulin receptor substrate-2)
*Akt1*	*Jak2* (janus kinase 2)
*Akt2*	*Lepr* (leptin receptor)
*Amylin*	*Lepr-b* (leptin receptor b)
*Arrb1* (β-arrestin 1)	*Mc4r* (melanocortin 4 receptor)
*Arrb2* (β-arrestin 2)	*Mch* (melanin concentrating hormone)
*Calcr* (calcitonin receptor)	*mTor* (mammalian target of rapamycin complex)
*Calcr-1a* (calcitonin receptor 1a)	*Npy* (neuropeptide Y)
*Calcr-1b* (calcitonin receptor 1b)	*Pde3b* (phosphodiesterase 3B)
*Cart* (cocaine and amphetamine regulated transcript)	*Pdk1* (3-phosphoinositide dependent kinase-1)
*c-Fos*	*Pias3* (protein inhibitor of activated STAT 3)
*Foxo1* (foxhead box protein O1)	*Pik3ca* (phosphoinositide-3 kinase catalytic subunit a)
*Gsk3a* (glycogen synthase kinase 3a)	*Pik3r1* (phosphoinositide-3 kinase regulatory subunit 1)
*Gsk3b* (glycogen synthase kinase 3b)	*Pomc* (pro-opiomelanocortin)
*Hcrt* (orexin)	*Ptp1b* (protein tyrosine phosphatase 1B)
*Hdac5* (histone deacetylase 5)	*Ramp1* (receptor activity modifying protein 1)
*Hdc* (histidine decarboxylase)	*Ramp2* (receptor activity modifying protein 2)
*Hrh1* (histamine receptor H1)	*Ramp3* (receptor activity modifying protein 3)
*Ins1* (insulin 1)	*Socs3* (suppressor of cytokine signalling 3)
*Ins2* (insulin 2)	*Stat3* (signal transducer and activator of transcription 3)
*Ir* (insulin receptor)	

## Results

### Changes in body weight and blood glucose levels at different disease stages in L44 mice

Mean body weight and blood glucose levels at collection time are shown in Table 2. All body weight and blood glucose measurements are available in the Figshare repository^31^. We found that HEM and HOM mice developed obesity and hyperglycaemia on a standard chow diet. Mice continued to gain weight throughout their lifespan, but the increase began to plateau with age.

**Table 2:** Mean final body weight and blood glucose measurements of L44 mice taken at T1, T2 and T3

Time Point	Nontransgenic (NON)		Hemizygous (HEM)		Homozygous (HOM)	
	Body Weight (g)	Blood Glucose (mM)	Body Weight (g)	Blood Glucose (mM)	Body Weight (g)	Blood Glucose (mM)
T1	30.97 ± 0.83	7.7 ± 0.2	32.44 ± 1.22	9.0 ± 0.6	32.76 ± 0.97	10.0 ± 0.2
T2	37.18 ± 1.60	7.3 ± 0.3	49.25 ± 1.25	17.2 ± 1.5	45.66 ± 2.04	16.3 ± 1.2
T3	47.34 ± 1.66	5.9 ± 0.2	54.62 ± 1.78	6.1 ± 0.2	54.37 ± 2.52	8.0 ± 0.8

All values are mean ± SEM. *n*: T1 NON = 9, T1 HEM = 9, T1 HOM = 8, T2 NON = 14, T2 HEM = 9, T2 HOM = 9, T3 NON = 17, T3 HEM = 17, T3 HOM = 16.

At T1 (100 days), NON mice had a mean weight of 30.97 g while HEM and HOM mice had similar weights with means of 32.44 g and 32.76 g respectively. ANOVA testing found no significant effect of genotype on body weight at this time (*p* = 0.43). NON mice had a mean blood glucose level of 7.7 mM, HEM mice had 9.0 mM, and HOM mice had 10.0 mM. There was a significant difference in blood glucose levels between NON and HOM mice (*p* = 0.0015). There was a trend towards significance between NON and HEM (*p* = 0.067), whereas the difference in blood glucose between HEM and HOM mice was nonsignificant (*p* = 0.22).

Mice were considered to be diabetic after three consecutive weekly blood glucose measurements >11 mM. The Kruskal-Wallis test found that NON controls had significantly lower body weight than diabetic HEM and HOM mice at T2 (diabetes onset) (*p* = 0.00052 vs HEM; *p* = 0.012 vs HOM). HEM mice had a mean body weight of 49.25 g and HOM mice a mean body weight of 45.66 g, compared to NON mice at 37.18 g. Diabetic HEM mice had a mean blood glucose level of 17.2 mM and HOM mice had 16.3 mM, compared to NON controls which had a mean blood glucose level of 7.3 mM. Diabetic mice that were not used for T2 sample collection eventually returned to normoglycaemia after a variable period of time (between a few weeks up to several months) and were normoglycaemic by T3.

At T3 (400 days), NON mice reached a mean body weight of 47.34 g, while HEM and HOM mice weighed 54.62 g and 54.37 g respectively. ANOVA analysis found the difference in body weight between NON and transgenic mice to be significant (*p* = 0.033 vs HEM; *p* = 0.044 vs HOM). However, there was no difference in body weight between HEM and HOM animals (*p* = 1.00). NON mice had a mean blood glucose level of 5.9 mM, HEM mice had 6.1 mM and HOM mice had 8.0 mM. The Kruskal-Wallis test found a significant difference in blood glucose levels between NON and HOM mice (*p* = 0.0068), but not between NON and HEM (*p* = 0.27) or HEM and HOM (*p* = 0.073).

Thus, our results show that mice carrying the tripro-hA transgene develop increased body weight whereas there was no difference between HEM and HOM mice, indicating body weight was not affected by transgene dose. Interestingly, we found that the percentage of L44 mice which became diabetic over the 400 days varied with genotype: 59% of HEM and 81% of HOM mice (data not shown). Additionally, 35% of NON mice also developed hyperglycaemia, although to a lesser degree and for a shorter period. This is a clear suggestion of transgene-dose effect on blood glucose. A chi-squared test found that genotype had a significant effect on the likelihood of developing hyperglycaemia (*p* = 0.028).

### Changes in circulating hormone levels at different disease stages in L44 mice

**Figure 1:**
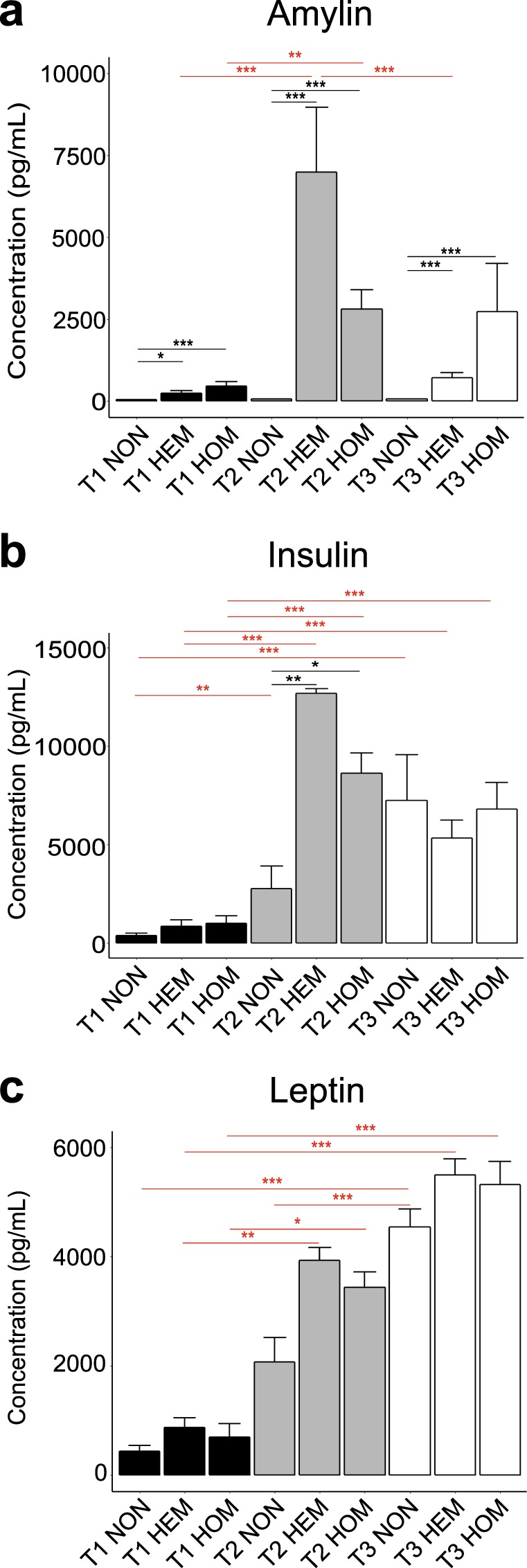
Mean serum concentrations of amylin (active, human and mouse) (**a**), insulin (**b)** and leptin (**c**) in L44 mice taken at T1, T2 and T3 (mean ± SEM). Black horizontal bars show a significant difference between genotypes within the same time point. Red horizontal bars show a significant difference between the same genotype across two time points. NON = nontransgenic, HEM = hemizygous, HOM = homozygous. *n*: pooled samples (individual mice): T1 NON = 5(9). T1 HEM = 5 (9), T1 HOM = 4(8), T2 NON = 7(14), T2 HEM = 5(9), T2 HOM = 5(9), T3 NON = 11(12), T3 HEM = 9(13), T3 HOM = 10(12). *p < 0.05, **p < 0.01, ***p < 0.001

Circulating serum hormone levels at each time point were analysed with a magnetic bead panel assay. The raw hormone concentration data can be found in the Figshare repository^31^. Figure 1a shows the mean circulating amylin concentration of each genotype at each time point. As expected, ANOVA testing found mice carrying the tripro-hA transgene had significantly greater levels of amylin than NON animals at each of these time points. At T1, amylin levels in HEM mice were almost 6-fold greater than NON (*p* = 0.025), and over 10-fold greater in HOM (*p* = 0.00097). At T2, HEM mice had 118-fold the concentration of NON mice (*p* < 0.0000), whereas HOM mice had 47-fold (*p* < 0.0000). At T3, amylin in HEM mice was elevated 14 times (*p* = < 0.0000), and HOM mice 55 times (*p* < 0.0000), over NON. However, amylin levels were not significantly different between HEM and HOM mice at any time point, indicating amylin levels were not gene-dose-dependent. In fact, HEM animals trended higher than HOM at T2, although this was not significant (*p* = 0.80). In transgenic animals, amylin concentration correlated with disease progression: levels were significantly elevated in HEM and HOM at T2 compared to T1 (*p* < 0.0000 and *p* = 0.0054 respectively), and compared with T3 in HEM animals (*p* = 2.5 × 10^−6^) but not HOM (*p* = 0.91).

As shown in Fig. 1b, at T1 insulin concentrations in HEM and HOM mice trended over two-fold higher than in NON mice, although this was not significant (*p* = 0.88 vs HEM; *p* = 0.75 vs HOM). However, they were significantly increased at T2 where levels were 5-fold higher in HEM mice (*p* = 0.00070) and 3-fold higher in HOM (*p* = 0.021) compared to NON controls. There was no difference between any of the genotypes at T3. Similar to amylin, there was no significant difference between HEM and HOM mice at any time point. Insulin concentrations at T2 and T3 were significantly elevated in transgenic mice compared to levels at T1 (*p* = 3 × 10^−7^ (T2 HEM); *p* = 0.00043 (T3 HEM); *p* = 0.0002 (T2 HOM); *p* = 0.0003 (T3 HOM)). In NON mice, insulin levels also significantly increased from T1 to later time points, although not to the same extent as diabetic mice (*p* = 0.003 (T2); *p* < 0.0000 (T3)). There were no significant changes between T2 and T3 in any genotype.

There were no differences in serum leptin levels between any of the genotypes within a time point (Fig. 1c). NON mice tended to have slightly lower leptin levels than transgenic animals; however, this did not reach significance at any time point. By contrast, leptin concentration increased over time. At T3, leptin concentration was significantly higher than at T1 in each genotype (*p* = 1 × 10^−7^ (NON); *p* < 0.0000 (HEM); *p* = 1 × 10^−7^ (HOM)). In transgenic mice, T2 levels were also significantly increased over T1 (*p* = 0.002 (HEM); *p = *0.018 (HOM)). In NON mice, the difference between T2 and T3 leptin concentration was significant (*p* = 0.00081).

### Altered expression of metabolic genes in different regions of the brain of L44 mice

We used the NanoString nCounter platform to investigate the expression of 43 genes involved in the amylin, insulin and leptin signal transduction pathways in the brain of L44 mice (Table 1). The brain was separated into four regions for analysis: the hindbrain (including the cerebellum), midbrain (including the forebrain and hypothalamus), left cortex and right cortex. Samples were collected in the morning – (AM; 10am-12pm) or afternoon – (PM, 1–3 pm). A preliminary analysis found that there were some differences in gene expression between the two collection times (unpublished data). Based on this, we decided to separate samples into AM and PM groups in order not to confound our results. There were no HEM samples in the AM group at T1.

Differential analysis of the mRNA counts produced from our target genes was performed using the NanoStringDiff R package. The raw NanoString data have been deposited in NCBI’s Gene Expression Omnibus and are accessible through GEO Series accession number GSE132940. Results from the NanoStringDiff analysis are presented in Supplementary Tables S1.

**Figure 2:**
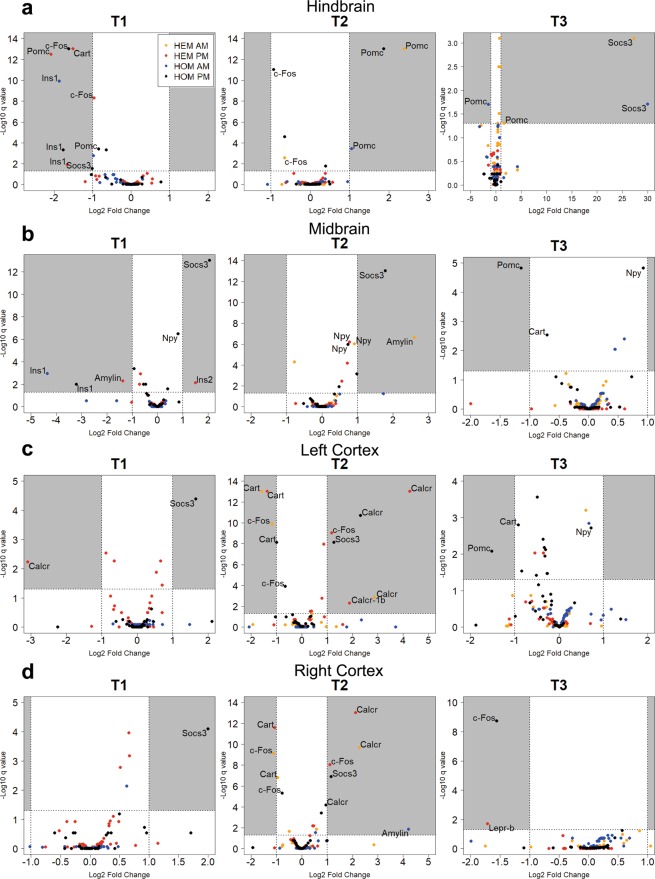
Differentially expressed genes in the hindbrain (**a**), midbrain (**b**), left cortex (**c**) and right cortex (**d**) of transgenic L44 mice compared to nontransgenic controls at T1, T2 and T3. mRNA for 43 genes in the amylin, insulin and leptin signalling pathways were counted by the NanoString nCounter system and analysed using the NanoStringDiff R package. Labelled genes that show a *q*-value < 0.05 (5% false discovery rate) and a log_2_ fold-change > |1| or consistently > |0.58| across multiple analyses. For the purposes of plotting data points on a logarithmic scale, genes with a *q* value of 0.00 × 10^−12^ were give the nominal value of 1.0 × 10^−13^ before -log_10_ transformation. There was no HEM AM group at T1. *n* = pooled samples (individual mice): **T1 AM:** NON = 2(4), HOM = 2(4), **T1 PM:** NON = 3(5), HEM = 2(3) (left cortex = 1(2)), HOM = 2(4), **T2 AM:** NON = 2(4), HEM = 2(3), HOM = 2(3), **T2 PM:** NON = 3(6), HEM = 3(6), HOM = 3(6), **T3 AM:** NON = 2(3), HEM = 2(3), HOM = 3(3), **T3 PM:** NON = 3(6), HEM = 2(4), HOM = 1(2)

Figure 2 shows the log_2_-fold change in gene expression in HEM and HOM animals when compared to NON controls. We applied a false discovery rate of 5% (*q* < 0.05 or –log_10_ *q* < 1.30, shown by the dotted line across the y-axis) and log_2_ fold-change threshold of |1| (representing a two-fold change, shown by the two dotted lines across the x-axis). Genes which met both these criteria were considered to be differentially expressed. These genes are labelled in Fig. 2. Genes that were consistently differently expressed across different analyses but fell short of the two-fold change threshold in some cases (but still showed a change greater than 1.5-fold) have also been labelled.

We found several genes that showed differential expression across multiple comparisons. *c-Fos*, a marker of neuronal amylin activation, was decreased at both T1 and T2 in hindbrain samples across both HEM and HOM genotypes (Fig. 2a). Interestingly, in HEM mice *c-Fos* was differentially expressed in both cortices at T2, but with expression lowered in the AM but elevated in the PM (Fig. 2c,d), consistent with circadian modulation of this response. In contrast, *c-Fos* expression was decreased in HOM animals in the PM, although this did not reach a two-fold change.

Expression of *Calcr*, which encodes the core component of the amylin receptors, was upregulated in both transgenic genotypes in both cortices at T2 (Fig. 2c,d). The *Calcr-1b* isoform was also significantly increased in the HEM PM group in the left cortex (Fig. 2c). *Ins1* expression was attenuated in the T1 hindbrain samples in both genotypes (Fig. 2a), as well as in the midbrain in T1 HOM animals (Fig. 2b). *Amylin* expression was decreased in the HEM midbrain at T1 (PM) and increased at T2 (AM, Fig. 2b). Amylin levels were also elevated in HOM animals in the right cortex at T2 (Fig. 2d).

Pomc is the precursor to α-melanocyte-stimulating hormone (α-MSH) which functions as an appetite suppressant. *Pomc* mRNA levels in the hindbrain were often differentially expressed and this fluctuated across the time points studied (Fig. 2a). At T1, *Pomc* was decreased in both genotypes in the PM. In contrast, hindbrain expression was increased in most groups at T2 (HEM AM, HOM AM and PM). At T3 in the AM, *Pomc* was upregulated in the HEM hindbrain but downregulated in HOM. In HOM mice only, *Pomc* was also decreased in the midbrain (Fig. 2b) and left cortex (Fig. 2c) at T3 in the PM.

In the T1 hindbrain, *Cart*, which also suppresses appetite and is expressed by *Pomc*-expressing neurons, was decreased in HEM PM samples (Fig. 2a). At T2, *Cart* levels were decreased in both cortices in HEM mice, as well as in the left cortex in HOM in the PM only (Fig. 2c,d). In contrast, the function of *Npy* is to increase food intake, opposing the action of *Pomc* and *Cart*. Although *Npy* never reached the two-fold change threshold, it was significantly upregulated by more than 1.5-fold in both the HEM and HOM midbrain at T2 (Fig. 2b). The HOM midbrain also showed higher expression at T1 and T3 (PM only). Given the distinct pattern of these expression changes, we have included *Npy* in the analysis as we believe this response to be biologically significant.

The expression of *Socs3*, an inhibitor of leptin, was consistently elevated in HOM animals in the midbrain and both cortices at T1 and T2 (Fig. 2b–d). Interestingly, this effect was only observed in HOM mice and only in samples collected in the PM. At T3, both HEM and HOM hindbrain showed dramatically increased *Socs3* expression but only in AM samples (Fig. 2a).

### Altered Socs3 protein expression in different regions of the brain of L44 mice

We investigated whether the alterations seen in our gene expression analysis also translated to changes in protein expression. We studied Socs3 by Western blotting as it showed consistent differences in mRNA levels between genotypes across several comparisons. We investigated selected groups based on the gene expression results: T1 and T3 in the hindbrain; and T1 and T2 in the midbrain, left and right cortices.

**Figure 3:**
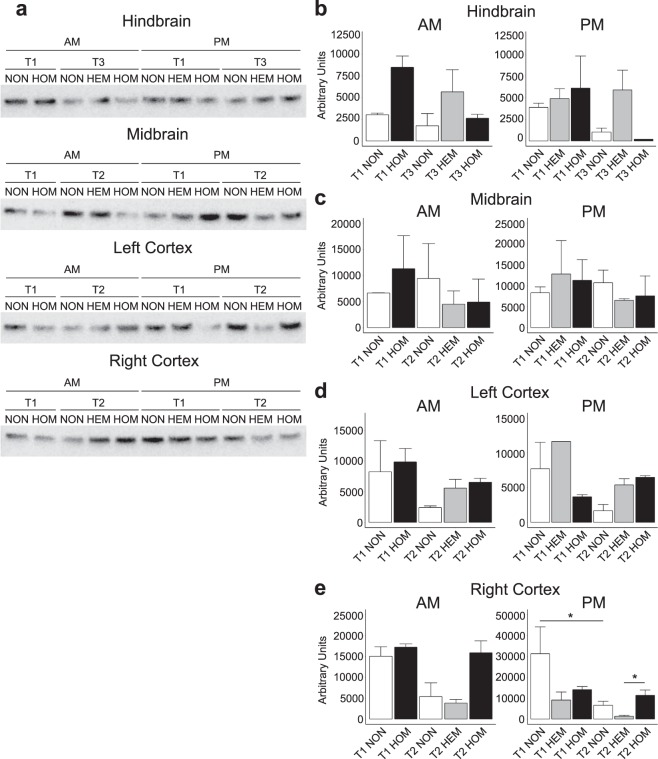
Socs3 Western blot bands at approximately 25 kDa (**a**). One band from each group has been shown. Mean intensity of Socs3 in the hindbrain (**b**), midbrain (**c**), left cortex (**d**) and right cortex (**e**). Mean ± SEM. Due to their large number, samples may have been run on different gels. A calibrator sample was repeated on each gel and results were calibrated to this prior to analysis. Total protein normalisation was performed with Ponceau S staining. Samples derive from the same experiment and blots were processed in parallel. Full blot images, raw data, total protein normalisation and calibrations are presented in Supplementary File S2. There was no HEM AM group at T1. *n* = pooled samples (individual mice): T1 AM: NON = 2(4), HOM = 2(4), T2 AM: NON = 2(4), HEM = 2(3), HOM = 2(3), T3 AM: NON = 2(3), HEM = 3(4), HOM = 5(8), T1 PM: NON = 3(5), HEM = 2(3) (left cortex = 1(2)), HOM = 2(4), T2 PM: NON = 5(10), HEM = 3(6), HOM = 3(6), T3 PM: NON = 4(7), HEM = 3(6), HOM = 1(2). *p < 0.05, differences between all other groups were nonsignificant.

Figure 3a shows a Socs3 protein band from each group analysed. In hindbrain samples (Fig. 3b) collected in the AM, two-way ANOVA analysis found no statistically significant effect of time (*p* = 0.15) or genotype (*p* = 0.12). In the PM, the effect of genotype was also nonsignificant (*p* = 0.065) but a significant effect of time point was present (*p* = 0.015). However, Tukey’s *post hoc* test found no significant differences between groups. At T1, there was a nonsignificant trend for HOM mice to have greater Socs3 levels than NON controls in the AM. At T3, there was a trend in the AM and PM for HEM mice to have higher SocsS3 levels than both NON and HOM counterparts. There also appeared to be a trend for Socs3 expression in HOM mice to decrease from T1 to T3 in both AM and PM. Generally, trends seen in AM samples were similar to those in PM samples.

In the midbrain, Socs3 levels were similar across groups (Fig. 3c). We did not find a significant effect of time (*p* = 0.39 (AM); *p* = 0.40 (PM)) or genotype (*p* = 0.91 (AM); *p* = 0.77 (PM)). Like the hindbrain, AM and PM results were evidently similar.

In the left cortex (Fig. 3d), we found no significant effect of time (*p* = 0.14) or genotype (*p* = 0.22) in the AM. In the PM, the effect of genotype was significant (*p* = 0.039) but not time point (*p* = 0.083). However, a subsequent *post hoc* test showed no significant differences between each group. AM and PM results were similar at T2 but not T1. There appeared to be a trend for NON mice to have decreased Socs3 expression from T1 to T2 in both AM and PM. Although nonsignificant, transgenic mice tended to show elevated Socs3 levels compared to NON mice at T2.

In the right cortex (Fig. 3e) in the AM, we found a significant effect of time point (*p* = 0.034) but not genotype (*p* = 0.10) on Socs3 expression. However, no significant differences were found when comparing groups with Tukey’s *post hoc* test. In the PM, both the time point (*p* = 0.0016) and genotype (*p* = 0.0049) effects were found to be significant. At T2, HOM mice tended to show elevated Socs3 expression compared to the other genotypes. The difference between HEM and HOM mice in the PM was statistically significant (*p* = 0.011). T2 NON levels were significantly decreased from T1 in the PM (*p* = 0.032). This trend was also seen in the AM. HEM mice also showed this downward trend in the PM, but only reached *p* = 0.051. Interestingly, Socs3 in HEM samples tended to be lower than in NON controls, showing the opposite trend to HOM mice, although this was nonsignificant. Like the left cortex, AM and PM results were similar at T2 but not at T1.

### Altered Stat3 phosphorylation in the right cortex of L44 mice

**Figure 4:**
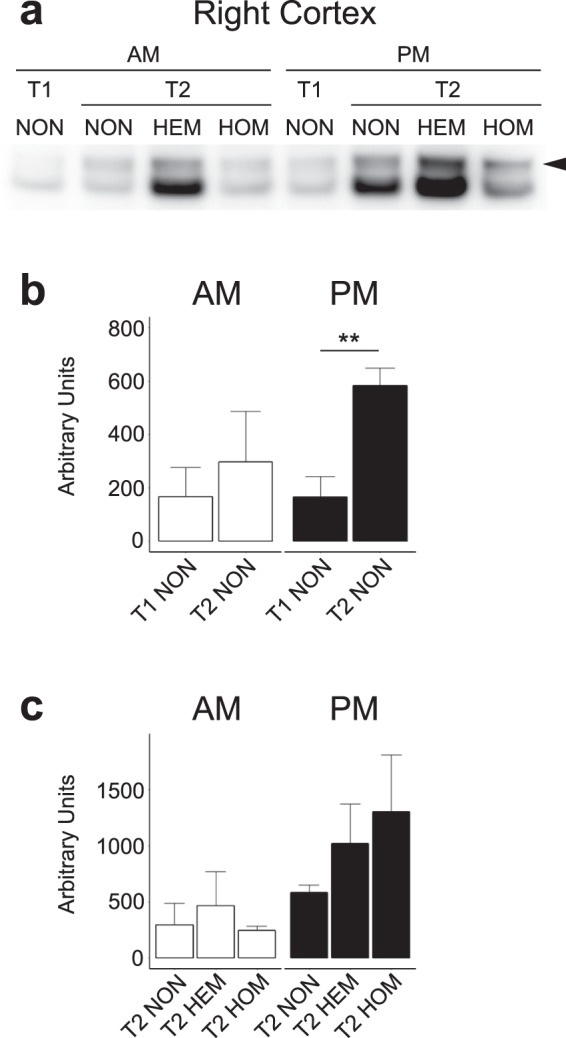
pSTAT3 Western blot bands at approximately 80 kDa indicated by the arrow (**a**). One band from each group has been shown. Mean intensity of pSTAT3 in the right cortex of nontransgenic mice (**b**) and at T2 (**c**). Mean ± SEM. Due to their large number, samples may have been run on different gels. A calibrator sample was repeated on each gel and results were calibrated to this prior to analysis. Total protein normalisation was performed with Ponceau S staining. Samples derive from the same experiment and blots were processed in parallel. Full blot images, raw data, total protein normalisation and calibrations are presented in Supplementary File S2. There was no HEM AM group at T1. *n* = pooled samples (individual mice): T1 NON: AM = 2(4), PM = 3(5), T2 NON: AM = 2(4), PM = 5(10), T2 HEM: AM = 2(3), PM = 3(6), T2 HOM: AM = 2(3), PM = 2(4). **p < 0.01, differences between all other groups were nonsignificant.

The leptin signalling pathway activates the transcription factor Stat3 by phosphorylation at Tyr705, which is inhibited by Socs3. We examined the levels of Tyr705 phosphorylated Stat3 (pStat3) by Western blot to determine whether altered Socs3 protein expression resulted in changes to Stat3 activation (Fig. 4a). We examined the right cortex groups which were found to have significant differences in Socs3 expression: T1 NON vs T2 NON, and T2 HEM, HOM and NON.

A *t*-test found T2 NON to have significantly greater levels of pStat3 than T1 NON in the PM (*p* = 0.0066). The same trend was seen in the AM, however this was nonsignificant (*p* = 0.62). These results are shown in Fig. 4b.

The Kruskal-Wallis test found no significant effect of genotype on the level of pStat3 in the right cortex at T2 in either AM (*p* = 0.87) or PM (*p* = 0.31). As shown in Fig. 4c, results in the AM were similar across genotype, however there was a nonsignificant trend for increased pStat3 in transgenic mice in the PM (correlating with transgene copy number). One HOM PM sample could not be included for analysis due to oversaturation, but fits the trend of the highest pStat3 levels being in this group.

## Discussion

This study has shown that mice which carry the tripro-hA transgene are significantly heavier than their nontransgenic counterparts at T2 (time of diabetes onset) and T3 (400 days). Although food intake was not measured in this study, we interpret the increase in body weight to be due to hyperphagia, although decreased physical activity may contribute. The proportion of mice which developed diabetes varies with genotype: 59% of HEM, 81% of HOM mice and 35% of NON. This is a clear suggestion of a gene-dosage effect; however, hyperamylinaemia was not enough to guarantee disease development. HOM mice also had more severe hyperglycaemia for a longer period of time (data not shown). Even prior to development of overt diabetes, both HEM and HOM mice had significantly higher blood glucose levels than NON controls at T1. At T3, transgenic mice were normoglycaemic but HOM animals still had significantly higher blood glucose levels than NON mice.

Around a third of NON mice developed diabetes, and while they had a lower mean body weight than transgenic animals they also tended to be overweight compared to wild-type FVB/n mice. NON mice were bred from HEM parents, and this may have influenced the offspring in several ways. Although breeders were monitored to ensure they were not hyperglycaemic, they would have been hyperamylinaemic. In addition, epigenetic or environmental factors, such as the *in utero* environment, lactation, microbiome, or stress may have had an effect. We did not investigate the cause of hyperglycaemia in NON animals in this study.

As expected, transgenic L44 mice were hyperamylinaemic compared to NON animals at all time points. We hypothesised that amylin overexpression drives changes in feeding behaviour and metabolism that results in the development of insulin resistance. Insulin levels in transgenic mice were significantly increased at T2 compared to T1 and higher than NON controls, supporting this hypothesis. At T2, amylin levels in HEM and HOM were also significantly increased compared to amylin levels at T1. This is likely due to the concurrent increase in insulin secretion at this stage. Amylin and insulin are co-secreted from β-cells and the tripro-hA transgene is expressed under the rat insulin 2 promotor, thus driving further overexpression when insulin expression is increased^3,24^. Amylin and insulin levels are also mirrored in transgenic mice at T3. Thus, while amylin overexpression drives the development of insulin resistance and increased insulin secretion, amylin secretion itself is also linked to the level of insulin secretion. In contrast, amylin levels in NON mice, which do not express the transgene, remained relatively low. However, insulin concentrations increased over time and at T3 NON mice had similar levels of insulin as their transgenic counterparts. A mechanism independent of circulating amylin levels drives this increase in these mice, and amylin and insulin levels become dissociated. It is known that insulin resistance increases with age and weight gain^32,33^. These are likely to be contributing factors, as our study was carried out over a long time period and NON mice tended to be overweight, although not to the extent of transgenic mice. Serum insulin levels were raised in all genotypes at T3 (compared to T1), indicating these animals may be partially insulin-resistant although not hyperglycaemic at this time. Leptin levels were not statistically different between genotypes at any time point. However, leptin levels increased over time as weight, and presumably fat mass, increased. This was expected as leptin is produced by adipocytes and patients with obesity are often leptin resistant^34,35^. In summary, transgenic mice were hyperamylinaemic at each time point, and hyperinsulinaemic at T2. While leptin levels were not statistically higher than NON controls, they climbed over time. As L44 mice developed obesity and diabetes despite high levels of amylin, insulin and leptin, they can be described as resistant to these hormones.

Our results reveal that the obese and diabetic phenotype of transgenic L44 mice is associated with several genes whose expression levels are altered in the brain. Analysis of the two cortices provided a useful internal control and results from the left and right cortices were similar in most cases as one would expect. Amylin is known to activate neurons in the area postrema of the hindbrain, where it induces expression of the transcription factor c-Fos^4,5,36–38^. Expression of *c-Fos* is decreased in the hindbrain at T1 in both HEM and HOM mice despite them being hyperamylinaemic compared to controls. This indicates amylin resistance is already present at the prediabetic stage, potentially driving hyperphagia and subsequently obesity. Hindbrain expression was also decreased during diabetes at T2. Together, these results indicate that hyperamylinaemia results in attenuated amylin signalling in the hindbrain in the prediabetic stage that continues during the early-stage of disease progression. To our knowledge, this shows for the first time that hyperamylinaemia leads to a decrease in activation of the amylin signalling pathway in the brain. Physiological evidence from a number of studies point to hyperamylinaemia-driven amylin resistance. For example, in a chronic infusion study, the anorectic effect of amylin was highest on the first day of infusion and decreased on the following days^39^; higher doses are needed to achieve an anorectic effect in some obese models^40^; and amylin levels are elevated in obese humans and mice^41,42^. However, our study is the first to provide molecular evidence for this phenomenon. These results support our hypothesis that chronic amylin overexpression leads to central amylin resistance. Interestingly, HEM mice showed decreased expression in the AM but increased expression in the PM in both cortices at T2, while HOM mice had decreased expression in the PM only. However, whether amylin has effects in the cortices has not been reported and the role of *c-Fos* here is unclear.

Calcr is a component of the amylin receptor. Its activity is modified by one of three proteins, Ramp1, Ramp2 and Ramp3, to form the three putative amylin receptors^43^. We found differential expression of *Calcr* in both cortices at T2, where it was increased in HEM animals in both the AM and PM, and in HOM animals in the PM. However, we cannot tell whether this upregulation results in increased levels of amylin receptors, as Calcr also functions on its own as the calcitonin receptor. Although all three *Ramp* mRNA transcripts were detected in each brain region, none were determined to be differentially expressed. A role for amylin in the cortical regions has not been previously reported.

*Ins1* was decreased in both the HEM and HOM hindbrain, as well as the midbrain of HOM mice at T1. While it is known that the brain expresses low levels of insulin, it is unclear what function locally expressed insulin may have and what effect the attenuated expression described here may have^44^. Likewise, expression of *amylin* in the brain has been previously described but there is little understanding of its function^45,46^. *Amylin* was differentially expressed in the midbrain at T1 (HEM PM) and T2 (HEM AM), as well as the right cortex at T2 (HOM AM), although there is no clear pattern or consistency in these gene expression changes. Thus, it is difficult to determine what, if any, effect this may have. We are also unable to determine whether these changes result from the endogenous amylin gene or the tripro-hA transgene. Future studies into the role of locally-expressed insulin and amylin in the brain in feeding or metabolism will be informative.

*Pomc* is expressed in both the hypothalamus and the nucleus of the solitary tract of the hindbrain where it influences feeding and metabolism^47^. *Pomc* expression is induced by insulin and leptin signalling and is processed to form α-MSH^48^, which acts via receptors such as Mc4r in the melanocortin system, leading to suppression of appetite^49,50^. α-MSH also has effects on hepatic glucose production^51^ and peripheral insulin sensitivity via increased lipogenesis^52^. We show here that *Pomc* was decreased in the prediabetic hindbrain in both HEM and HOM mice; this suggests downregulation of insulin and/or leptin signalling, in turn leading to hyperphagia, weight gain and further resistance to insulin and leptin. However, during diabetes *Pomc* was elevated in the hindbrain, perhaps suggesting a counter-regulatory effect and intact insulin and/or leptin signalling up to this point in the pathway.

Cart is another appetite-suppressing neuropeptide also expressed by Pomc neurons and stimulated by insulin and leptin^53,54^. *Cart* was differentially expressed in HEM animals, being decreased in the T1 hindbrain like *Pomc* (PM only). This likely also contributed to decreased appetite suppression by insulin and leptin. In HEM mice, it was also decreased in both cortices at T2, as well as the left cortex of HOM mice in the PM. What effect it may have on feeding or metabolism in the cortices is unknown. *Cart* is known to be expressed in distinct cortical regions^55^. In the rat brain, *Cart* mRNA was found mainly in the piriform and somatosensory cortices, suggesting it may be involved in smell and sensory functions^55^.

*Npy* works in opposition to *Pomc* and *Cart* in the melanocortin system in the hypothalamus. It delays the perception of fullness during a meal and promotes fat storage, and is normally suppressed by the insulin and leptin pathways^48,56,57^. *Npy* was increased by more than 1.5-fold in the midbrain (the hypothalamus was included in this region in this study) of transgenic mice in the PM during diabetes despite high levels of insulin. This reflects an inability of insulin and leptin to suppress *Npy* expression as normal, contributing to the obese phenotype of L44 transgenic mice. Additionally, HOM mice also displayed higher midbrain expression of *Npy* at the other time points, which may explain the prolonged and greater hyperglycaemia in these animals.

Socs3 is a well-known inhibitor of leptin signalling. It is activated by Stat3 (a transcription factor activated by leptin signalling) and binds the leptin receptor, thus forming a negative feedback loop^58–60^. In HOM animals, *Socs3* was increased in the midbrain and both cortices at T1 and T2 in the PM only. This indicates greater time-specific downregulation of leptin signalling before and during diabetes, and may have contributed to the greater disease severity in HOM mice. Despite the raised Socs3 mRNA levels at several points, this did not necessarily translate into measurably increased protein expression, although there were nonsignificant increases in both cortices at T2 and the HEM hindbrain at T3. There could be many reasons for this. Protein production is regulated by a number of processes and is not translated from all mRNA molecules, nor necessarily at a rate proportional to mRNA levels. Socs3 protein could also be degraded more rapidly. The results from tissue collected in the AM and PM tended to be more similar to each other in protein expression than the mRNA data from NanoString analysis, except at T1 in the cortices. This may be due to a delay in time for changes at the mRNA level to be translated and may not be apparent in protein levels at the time of sample collection. It is possible they may result in altered protein expression later in the day.

We did observe a significant increase in Socs3 protein expression in HOM mice compared to HEM at T2 in the right cortex in the PM. This was paralleled by a nonsignificant trend in the AM. The same pattern, although nonsignificant was also observed between HOM and NON mice. We examined the level of activated pStat3 in the right cortex at T2 but did not find a significant difference. Time point had a significant effect on Soc3 protein expression in NON animals, with a significant decrease from T1 to T2 in the right cortex in the PM. An equivalent but nonsignificant trend was also present in the AM and in the left cortex (both AM and PM). In the hindbrain, Socs3 expression also decreased at T3 compared to T1 although this was not significant. This is consistent with the gene expression results showing *Socs3* was increased at T3 in the hindbrain in transgenic mice compared to NON animals (AM only). This suggests there was an overall decrease in Socs3 protein in NON mice at later time points. At least in the right cortex, this translated into significantly increased pStat3 at T2 compared to T1. This may have been a mechanism to maintain greater leptin sensitivity in NON mice, which was missing in transgenic mice.

A striking aspect of the disease progression in L44 mice is that blood glucose levels return to normoglycaemia after a period of time. One hypothesis is that hyperglycaemia can resolve over time; however, by this stage β-cell dysfunction would normally prevent this from occurring. However, since L44 mice do not form cytotoxic amylin oligomers, this does not occur. While *c-Fos* expression in the hindbrain is attenuated in transgenic animals at T1 and T2, this was not significantly different from NON controls at T3. This suggests amylin sensitivity may be partially restored at this time. Although decreased at T1, *Pomc* levels are elevated at T2, suggesting restored insulin and/or leptin signalling pathways up to this point. This may have contributed to the eventual return to normoglycaemia. Studies into how this apparent re-sensitisation to amylin and insulin occurs could give insights on how to better treat and manage diabetes in human patients.

While we have discussed a number of genes that were differentially expressed, our data also show many genes that were not differentially expressed according to our criteria. However, it is still possible that these targets play a role in T2DM and may be modulated in ways other than gene expression, such as translation to protein, post-translational modifications, and degradation. The negative results from this study are also informative; consequently, the full NanoString dataset is publicly available (accessible at NCBI GEO database, accession GSE132940) and the differential gene expression analysis results are provided in Supplementary Tables S1.

**Figure 5:**
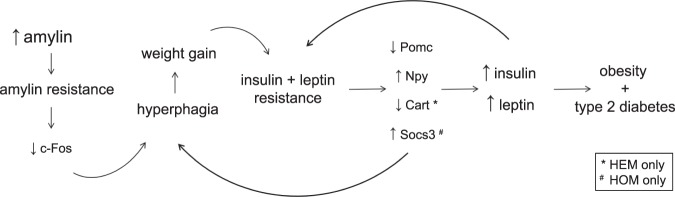
Summary of major findings and hypothesis for the development of obesity and diabetes in L44 mice.

In conclusion, overexpression of a non-cytotoxic amylin agonist in the L44 transgenic line causes obesity, hyperinsulinaemia, perturbed metabolic hormone signalling in the brain, and overt diabetes. Our chronic overexpression model allowed us to examine the effects of hyperamylinaemia over 400 days and provided data on a considerably longer timeframe than any study previously reported. Central resistance to amylin, insulin and leptin signalling, such as is indicated by attenuated expression of *c-Fos* and elevated levels of *Socs3*, led to the altered expression of neuropeptides in the melanocortin system. These appetite- and metabolism-controlling genes, *Pomc*, *Cart* and *Npy*, likely contributed to the obese and diabetic phenotype of L44 mice. These major findings have been summarised in Fig. 5. Hyperamylinaemia alone is not enough to guarantee the development of diabetes always. T2DM is a complex and heterogeneous disease which may be the result of a myriad of factors. However, hyperamylinaemia was clearly a large and significant contributor in this study. Further investigation into hyperamylinaemia and its effects to alter central metabolic gene expression is warranted to identify new therapeutic targets for the treatment of obesity and diabetes.

## Methods

### Animal protocols

The L44 model was developed on the FVB/n background by pronuclear microinjection into fertilised oocytes of the tripro-hA transgene, expressed under the rat insulin 2 promoter^24^. Multiple, separate, lines were created, which displayed a similar phenotype indicating insertional mutagenesis was not responsible. HEM mice have 9 ± 2 copies of the transgene and HOM mice have 18 ± 4 (unpublished data). All mice were bred and maintained at the Integrated Physiology Unit at the University of Auckland. Animal protocols were approved by the Animals Ethics Committee at the University of Auckland, and carried out in accordance with the New Zealand Animal Welfare Act (1999) and with the “Ethical principles and guidelines for scientific experiments on animals” of the Swiss Academy of Medical Sciences. Mice were maintained under a 12 hour light/dark cycle, 19–21 °C room temperature, 50–70% humidity, and housed 1–3 per cage with standard chow (Teklad Global Diet, Envigo) and tap water *ad libitum* at all times. Experimental mice were bred from HEM x HEM mating pairs. NON littermates were preferred as controls; if unavailable, age-matched controls from another litter were used. Non-fasting blood glucose was monitored weekly from weaning with a blood glucose monitor (Accu-Chek Advantage, Roche). The upper limit of detection was 33.3 mM. Body weight was also measured weekly using a digital balance.

We examined male mice at three time points: ***T1:**** Prediabetic (100 days):* before blood glucose levels become hyperglycaemic; ***T2:**** Diabetes onset:* blood glucose levels >11 mM for three consecutive measurements; ***T3:**** Post-diabetic (400 days):* after blood glucose levels have returned to normoglycaemic levels.

Mice were anaesthetised using isoflurane gas followed by cervical dislocation. Brain tissue was collected and sectioned into four regions: the hindbrain (including the cerebellum), midbrain (including the forebrain), left cerebral cortex and right cerebral cortex. Tissue for RNA extraction was stored in RNAlater (Thermo Fisher Scientific) overnight at 4 °C, then at −80 °C. Tissue for protein extraction was snap-frozen with liquid nitrogen and stored at −80 °C. Blood was collected from each animal through cardiac puncture and dispensed into a BD Microtainer SST Tube. Serum was separated by centrifugation and stored at −80 °C.

### Serum hormone quantification

The concentrations of amylin, insulin and leptin in serum were quantified using the MILLIPLEX MAP Mouse Metabolic Hormone Magnetic Bead Panel (Merck Millipore) in accordance to the manufacturer’s instructions.

### RNA isolation

RNA was extracted using the RNeasy Mini or Midi Lipid Tissue Kit (Qiagen) according to the manufacturer’s instructions.

### NanoString nCounter

The NanoString nCounter (NanoString Technologies) system, using a customised CodeSet of mRNA probes, was used to quantify the amount of mRNA for 43 target genes in each RNA sample. 5 µL of RNA sample was added to the hybridisation mix and incubated at 65 °C for 17 hours. Samples were run on the nCounter Prep Station, then run on the imager using 280 field of view counts. Various genes involved in the signalling and regulatory pathways of amylin, insulin and leptin were *a priori* targeted with the nCounter system, as listed in Table 1. Three reference genes, *Hprt1*, *B2m* and *Ubc*, evaluated by qPCR, were included for normalisation.

### SDS-PAGE and western blot

Protein was extracted from tissue in radioimmunoprecipitation assay (RIPA) buffer containing protease and phosphatase inhibitors (cOmplete Mini Protease Inhibitor Cocktail tablets and PhosSTOP Phosphatase Inhibitor Cocktail tablets, Roche). Equal amounts of protein (24.05 μg) were loaded onto an SDS-PAGE gel and separated. Samples were transferred to a nitrocellulose membrane (Genscript). The membrane was blocked using a 5% non-fat milk powder solution, then incubated overnight with the primary antibody anti-Socs3 (ab16030, Abcam) or anti-Stat3 phospho Y705 (ab76315, Abcam), both at 1/500 dilution, washed, then incubated with the secondary antibody horseradish peroxidase-conjugated goat anti-rabbit IgG (sc2004, Santa Cruz Biotechnology) at 1/50,000 dilution for 1–2 hours. Signal detection was performed using a chemiluminescent detection reagent (Amersham ECL Prime, GE Healthcare) and imaged using the Amersham Imager 600. Band intensity was calculated using the Image Studio Lite software (LI-COR Biosciences). Median local background subtraction was used, using the top and bottom area outside the selection with a border width of 3. Due to the large number of samples, it was necessary to run samples on multiple gels. A sample was repeated on each gel performed (a calibrator sample), which was used to calibrate the raw band intensity results across each image. Total protein normalisation was done by densitometric analysis of the membrane stained with Ponceau S as described previously^61^.

### Sample pooling

Due to their large number, samples were pooled together for analysis. Two samples were pooled only if they were of the same genotype and collection time. The same quantity of RNA or protein from each sample was mixed, thus results represent the mean of the two samples.

### Statistics

Statistical analysis was performed using programmes written in the R programming language (version 3.2.3). Body weight, blood glucose, serum hormone and Western blot data were analysed using ANOVA (with Tukey’s HSD *post hoc* test for multiple comparisons), the Kruskal–Wallis test by ranks (with Dunn’s *post hoc* test for multiple comparisons) or Student’s *t*-test. Data were checked for normality using quantile-quantile plots. If data did not fit a normal distribution, the data were log_10_ transformed or a non-parametric test was chosen. Gene expression was analysed using the NanoStringDiff R package^62^. All values are given as mean ± SEM. *P* values < 0.05 were considered significant.

## Supplementary information

Supplementary Tables S1

Supplementary File S2

## Data Availability

The gene expression datasets generated during the current study have been deposited in NCBI’s Gene Expression Omnibus and are accessible through GEO Series accession number GSE132940 [https://www.ncbi.nlm.nih.gov/geo/query/acc.cgi?acc=GSE132940]. The body weight, blood glucose and serum hormone datasets are available in the Figshare repository 10.17608/k6.auckland.8285909.v2. All other data generated or analysed during this study are included in this published article (including its Supplementary Information files).
