# Supplementary material for: Altered metabolic gene expression in the brain of a triprolyl-human amylin transgenic mouse model of type 2 diabetes
Source: Sci Rep. 2019 Oct 10;9:14588. doi: 10.1038/s41598-019-51088-x (PMC6787337; doi:10.1038/s41598-019-51088-x)
Supplement: Supplementary file 1 — Supplementary Tables S1 [file 41598_2019_51088_MOESM1_ESM.docx]

# Supplementary Tables S1. Results from NanoStringDiff Analysis

**Article title:** Altered metabolic gene expression in the brain of a triprolyl-human amylin transgenic mouse model of type 2 diabetes

**Journal:** Scientific Reports

**Authors:** Tina Nie, Shaoping Zhang, Greeshma Vazhoor Amarsingh, Hong Liu, Mark J. McCann, Garth J.S. Cooper

**Corresponding author:** Garth J.S. Cooper (School of Biological Sciences, Faculty of Science, the University of Auckland; The Maurice Wilkins Centre for Molecular Biodiscovery, Faculty of Science, the University of Auckland; Centre for Advanced Discovery and Experimental Therapeutics, Faculty of Biology, Medicine & Health, School of Medical Sciences, Division of Cardiovascular Sciences, the University of Manchester), [g.cooper@auckland.ac.nz](mailto:g.cooper@auckland.ac.nz)

Genes that are negatively differentially expressed compared to nontransgenic controls are highlighted in yellow. Genes that are positively differentially expressed compared to nontransgenic controls are highlighted in green.

Differentially expressed is defined as having a q value (adjusted p value) < 0.05 and a log_2_ fold-change > |1|.

Abbreviations:

HEM = hemizygous

HOM = homozygous

NON = nontransgenic

FC = fold-change

### T1 Hindbrain: HEM vs NON (PM)

| **Gene** | **log_2_ FC** | **Likelihood-ratio test** | **p value** | **q value** |
| --- | --- | --- | --- | --- |
| *Adrbk1* | 0.039162525 | 7.224054e-02 | 7.881021e-01 | 9.870303e-01 |
| *Agrp* | -0.825065530 | 4.809107e+00 | 2.830972e-02 | 1.521647e-01 |
| *Akt1* | 0.034076186 | 5.532451e-02 | 8.140445e-01 | 9.870303e-01 |
| *Akt2* | 0.126290256 | 7.876586e-01 | 3.748091e-01 | 7.007300e-01 |
| *Arrb1* | -0.010958232 | 5.590582e-03 | 9.403976e-01 | 9.870303e-01 |
| *Arrb2* | 0.006928962 | 2.375326e-03 | 9.611286e-01 | 9.870303e-01 |
| *Calcr* | 0.418231446 | 6.603953e+00 | 1.017526e-02 | 8.750726e-02 |
| *Calcr-1a* | 0.219780295 | 6.029298e-01 | 4.374624e-01 | 7.453048e-01 |
| *Calcr-1b* | 0.558100655 | 3.501896e+00 | 6.129862e-02 | 2.928712e-01 |
| *Cart* | -1.511422329 | 8.356723e+01 | 0.000000e+00 | 0.000000e+00 |
| *Foxo1* | -0.007074184 | 2.619846e-03 | 9.591786e-01 | 9.870303e-01 |
| *Gsk3a* | -0.026151024 | 2.937119e-02 | 8.639248e-01 | 9.870303e-01 |
| *Gsk3b* | -0.005357822 | 1.802207e-03 | 9.661380e-01 | 9.870303e-01 |
| *Hcrt* | -0.892325111 | 3.113538e+00 | 7.764415e-02 | 3.338698e-01 |
| *Hdac5* | 0.143692150 | 1.009170e+00 | 3.151018e-01 | 6.452084e-01 |
| *Hdc* | 0.020690908 | 1.363244e-02 | 9.070518e-01 | 9.870303e-01 |
| *Hrh1* | -0.154824397 | 1.039659e+00 | 3.079008e-01 | 6.452084e-01 |
| *Amylin* | -1.187417085 | 1.454972e+00 | 2.277318e-01 | 5.460389e-01 |
| *Ins1* | -1.662245274 | 1.060343e+01 | 1.128781e-03 | 1.213439e-02 |
| *Ins2* | 0.536574744 | 6.751633e-01 | 4.112572e-01 | 7.368359e-01 |
| *Ir* | -0.203696236 | 2.130058e+00 | 1.444353e-01 | 4.436226e-01 |
| *Irs1* | -0.194175490 | 1.516208e+00 | 2.181942e-01 | 5.460389e-01 |
| *Irs2* | 0.088794696 | 4.096579e-01 | 5.221432e-01 | 7.742123e-01 |
| *Jak2* | -0.103342755 | 4.868970e-01 | 4.853148e-01 | 7.453048e-01 |
| *Lepr* | -0.211363947 | 2.137243e+00 | 1.437601e-01 | 4.436226e-01 |
| *Lepr-b* | -0.262733939 | 1.633828e+00 | 2.011744e-01 | 5.460389e-01 |
| *Mc4r* | -0.112527111 | 5.164433e-01 | 4.723628e-01 | 7.453048e-01 |
| *Mch* | 0.003220922 | 2.642514e-04 | 9.870303e-01 | 9.870303e-01 |
| *Npy* | 0.132272797 | 9.124790e-01 | 3.394575e-01 | 6.634852e-01 |
| *Ptp1b* | 0.005933133 | 1.807308e-03 | 9.660902e-01 | 9.870303e-01 |
| *Pde3b* | -0.165975087 | 1.373141e+00 | 2.412730e-01 | 5.460389e-01 |
| *Pdk1* | -0.206612097 | 2.174329e+00 | 1.403307e-01 | 4.436226e-01 |
| *Pias3* | -0.234809113 | 2.703613e+00 | 1.001211e-01 | 3.913826e-01 |
| *Pik3ca* | -0.077262538 | 2.572977e-01 | 6.119827e-01 | 8.488793e-01 |
| *Pik3r1* | 0.058591924 | 1.674645e-01 | 6.823751e-01 | 9.169415e-01 |
| *Pomc* | -2.081530635 | 5.906113e+01 | 1.532108e-14 | 3.294032e-13 |
| *Ramp1* | 0.317602691 | 5.246970e+00 | 2.198502e-02 | 1.496541e-01 |
| *Ramp2* | 0.165194419 | 1.382375e+00 | 2.396971e-01 | 5.460389e-01 |
| *Ramp3* | -0.013975453 | 8.747212e-03 | 9.254853e-01 | 9.870303e-01 |
| *Socs3* | -0.921179204 | 5.068654e+00 | 2.436229e-02 | 1.496541e-01 |
| *Stat3* | -0.102213295 | 5.327159e-01 | 4.654673e-01 | 7.453048e-01 |
| *c-fos* | -0.960631515 | 3.935676e+01 | 3.530292e-10 | 5.060085e-09 |
| *mTor* | -0.074981332 | 2.801125e-01 | 5.966275e-01 | 8.488793e-01 |

### T1 Hindbrain: HOM vs NON (AM)

| **Gene** | **log_2_ FC** | **Likelihood-ratio test** | **p value** | **q value** |
| --- | --- | --- | --- | --- |
| *Adrbk1* | 0.114019516 | 5.062642e-01 | 4.767606e-01 | 9.801946e-01 |
| *Agrp* | -0.979989924 | 1.562630e+01 | 7.717384e-05 | 1.659238e-03 |
| *Akt1* | 0.297944098 | 3.432277e+00 | 6.393389e-02 | 3.054619e-01 |
| *Akt2* | 0.076598831 | 2.152568e-01 | 6.426780e-01 | 9.801946e-01 |
| *Arrb1* | 0.091997105 | 3.251440e-01 | 5.685326e-01 | 9.801946e-01 |
| *Arrb2* | -0.002116651 | 1.661041e-04 | 9.897170e-01 | 1.000000e+00 |
| *Calcr* | -0.441705406 | 6.136487e+00 | 1.324202e-02 | 1.138813e-01 |
| *Calcr-1a* | 0.064930128 | 5.994266e-02 | 8.065866e-01 | 9.801946e-01 |
| *Calcr-1b* | -0.383593762 | 1.967596e+00 | 1.607032e-01 | 5.934797e-01 |
| *Cart* | 0.082656672 | 2.458935e-01 | 6.199815e-01 | 9.801946e-01 |
| *Foxo1* | 0.128247529 | 6.255494e-01 | 4.289926e-01 | 9.801946e-01 |
| *Gsk3a* | 0.002862756 | 2.555060e-04 | 9.872467e-01 | 1.000000e+00 |
| *Gsk3b* | 0.060651187 | 1.388940e-01 | 7.093830e-01 | 9.801946e-01 |
| *Hcrt* | -0.612942188 | 4.212894e+00 | 4.011784e-02 | 2.616858e-01 |
| *Hdac5* | 0.042323403 | 6.990973e-02 | 7.914683e-01 | 9.801946e-01 |
| *Hdc* | -0.363151633 | 3.552978e+00 | 5.943867e-02 | 3.054619e-01 |
| *Hrh1* | -0.074729069 | 1.904526e-01 | 6.625402e-01 | 9.801946e-01 |
| *Amylin* | 25.875573736 | 0.000000e+00 | 1.000000e+00 | 1.000000e+00 |
| *Ins1* | -1.868136493 | 4.872296e+01 | 2.947975e-12 | 1.267629e-10 |
| *Ins2* | -0.808102357 | 1.456494e+00 | 2.274889e-01 | 6.521347e-01 |
| *Ir* | 0.059369277 | 1.346084e-01 | 7.137009e-01 | 9.801946e-01 |
| *Irs1* | -0.129978976 | 5.552428e-01 | 4.561834e-01 | 9.801946e-01 |
| *Irs2* | 0.123791037 | 5.876961e-01 | 4.433114e-01 | 9.801946e-01 |
| *Jak2* | -0.051403757 | 1.010923e-01 | 7.505228e-01 | 9.801946e-01 |
| *Lepr* | -0.221016447 | 1.802388e+00 | 1.794241e-01 | 5.934797e-01 |
| *Lepr-b* | -0.276952562 | 1.922015e+00 | 1.656347e-01 | 5.934797e-01 |
| *Mc4r* | -0.096702184 | 3.145939e-01 | 5.748748e-01 | 9.801946e-01 |
| *Mch* | -0.407443450 | 4.111204e+00 | 4.260002e-02 | 2.616858e-01 |
| *Npy* | -0.087648927 | 2.952204e-01 | 5.868945e-01 | 9.801946e-01 |
| *Ptp1b* | 0.056016017 | 1.211202e-01 | 7.278227e-01 | 9.801946e-01 |
| *Pde3b* | -0.026324085 | 2.663839e-02 | 8.703510e-01 | 9.848708e-01 |
| *Pdk1* | -0.203408438 | 1.587587e+00 | 2.076712e-01 | 6.378473e-01 |
| *Pias3* | -0.045209565 | 7.622810e-02 | 7.824756e-01 | 9.801946e-01 |
| *Pik3ca* | -0.071430830 | 1.847475e-01 | 6.673247e-01 | 9.801946e-01 |
| *Pik3r1* | 0.007138201 | 2.012868e-03 | 9.642149e-01 | 1.000000e+00 |
| *Pomc* | -0.469058234 | 6.462024e+00 | 1.102040e-02 | 1.138813e-01 |
| *Ramp1* | 0.010431428 | 4.242152e-03 | 9.480691e-01 | 1.000000e+00 |
| *Ramp2* | 0.117356090 | 5.175513e-01 | 4.718881e-01 | 9.801946e-01 |
| *Ramp3* | 0.061992676 | 1.337878e-01 | 7.145366e-01 | 9.801946e-01 |
| *Socs3* | -0.677280850 | 6.425428e+00 | 1.124978e-02 | 1.138813e-01 |
| *Stat3* | -0.033294351 | 4.274555e-02 | 8.362051e-01 | 9.801946e-01 |
| *c-fos* | -0.223601621 | 1.895308e+00 | 1.686045e-01 | 5.934797e-01 |
| *mTor* | 0.031773327 | 3.901281e-02 | 8.434233e-01 | 9.801946e-01 |

### T1 Hindbrain: HOM vs NON (PM)

| **Gene** | **log_2_ FC** | **Likelihood-ratio test** | **p value** | **q value** |
| --- | --- | --- | --- | --- |
| *Adrbk1* | 0.084832171 | 3.724844e-01 | 5.416532e-01 | 0.9156969575 |
| *Agrp* | -0.657636757 | 4.576295e+00 | 3.241723e-02 | 0.1991343975 |
| *Akt1* | -0.054544674 | 3.589423e-01 | 5.490943e-01 | 0.9156969575 |
| *Akt2* | 0.017083428 | 1.475679e-02 | 9.033128e-01 | 0.9468247524 |
| *Arrb1* | 0.143578405 | 9.488929e-01 | 3.300013e-01 | 0.9156969575 |
| *Arrb2* | 0.163847593 | 1.348341e+00 | 2.455684e-01 | 0.9156969575 |
| *Calcr* | 0.064276223 | 1.651581e-01 | 6.844513e-01 | 0.9156969575 |
| *Calcr-1a* | 0.186973722 | 5.771620e-01 | 4.474269e-01 | 0.9156969575 |
| *Calcr-1b* | 0.282049303 | 1.092692e+00 | 2.958754e-01 | 0.9156969575 |
| *Cart* | -0.639105273 | 1.672625e+01 | 4.317934e-05 | 0.0004641779 |
| *Foxo1* | -0.043973487 | 1.024321e-01 | 7.489303e-01 | 0.9201144060 |
| *Gsk3a* | 0.066393977 | 1.929771e-01 | 6.604503e-01 | 0.9156969575 |
| *Gsk3b* | 0.011465616 | 1.069190e-02 | 9.176442e-01 | 0.9468247524 |
| *Hcrt* | -1.030293807 | 5.841284e+00 | 1.565440e-02 | 0.1121898539 |
| *Hdac5* | -0.016889965 | 1.393716e-02 | 9.060235e-01 | 0.9468247524 |
| *Hdc* | 0.106496231 | 4.191904e-01 | 5.173413e-01 | 0.9156969575 |
| *Hrh1* | -0.134817625 | 8.442327e-01 | 3.581890e-01 | 0.9156969575 |
| *Amylin* | -0.499554235 | 5.601896e-01 | 4.541839e-01 | 0.9156969575 |
| *Ins1* | -1.760057680 | 1.684935e+01 | 4.046711e-05 | 0.0004641779 |
| *Ins2* | 0.776966623 | 2.253931e+00 | 1.332754e-01 | 0.5730844072 |
| *Ir* | 0.052462574 | 1.456329e-01 | 7.027442e-01 | 0.9156969575 |
| *Irs1* | -0.106754653 | 5.047833e-01 | 4.774059e-01 | 0.9156969575 |
| *Irs2* | 0.015161537 | 1.208176e-02 | 9.124752e-01 | 0.9468247524 |
| *Jak2* | -0.073602465 | 2.935886e-01 | 5.879300e-01 | 0.9156969575 |
| *Lepr* | 0.062440054 | 1.972746e-01 | 6.569298e-01 | 0.9156969575 |
| *Lepr-b* | -0.209392176 | 1.284834e+00 | 2.570022e-01 | 0.9156969575 |
| *Mc4r* | -0.269268517 | 3.161176e+00 | 7.540886e-02 | 0.3602867667 |
| *Mch* | -0.095981924 | 2.598922e-01 | 6.101942e-01 | 0.9156969575 |
| *Npy* | 0.023432597 | 3.084239e-02 | 8.605925e-01 | 0.9468247524 |
| *Ptp1b* | -0.003932601 | 8.367091e-04 | 9.769237e-01 | 0.9769236692 |
| *Pde3b* | -0.045902386 | 1.066749e-01 | 7.439619e-01 | 0.9201144060 |
| *Pdk1* | 0.055952586 | 1.636663e-01 | 6.858032e-01 | 0.9156969575 |
| *Pias3* | 0.069775720 | 2.496579e-01 | 6.173160e-01 | 0.9156969575 |
| *Pik3ca* | -0.063311337 | 1.784256e-01 | 6.727297e-01 | 0.9156969575 |
| *Pik3r1* | -0.019400487 | 1.969065e-02 | 8.884045e-01 | 0.9468247524 |
| *Pomc* | -0.839764418 | 1.832217e+01 | 1.865242e-05 | 0.0004010270 |
| *Ramp1* | -0.080690955 | 7.270577e-01 | 3.938386e-01 | 0.9156969575 |
| *Ramp2* | 0.287938735 | 4.337222e+00 | 3.728772e-02 | 0.2004214950 |
| *Ramp3* | 0.090407301 | 3.931051e-01 | 5.306717e-01 | 0.9156969575 |
| *Socs3* | -1.010313948 | 8.554751e+00 | 3.446216e-03 | 0.0296374619 |
| *Stat3* | -0.013114830 | 8.907996e-03 | 9.248056e-01 | 0.9468247524 |
| *c-fos* | -1.615748865 | 1.059076e+02 | 0.000000e+00 | 0.0000000000 |
| *mTor* | 0.095738155 | 4.660692e-01 | 4.948011e-01 | 0.9156969575 |

### T1 Midbrain: HEM vs NON (PM)

| **Gene** | **log_2_ FC** | **Likelihood-ratio test** | **p value** | **q value** |
| --- | --- | --- | --- | --- |
| *Adrbk1* | 0.1633765429 | 1.658616e+00 | 1.977904e-01 | 0.472499181 |
| *Agrp* | -0.7096087337 | 1.095922e+01 | 9.313897e-04 | 0.010012439 |
| *Akt1* | 0.0095963937 | 4.371243e-03 | 9.472860e-01 | 0.969840395 |
| *Akt2* | 0.2017834582 | 1.960831e+00 | 1.614245e-01 | 0.472499181 |
| *Arrb1* | 0.0940937221 | 4.489609e-01 | 5.028288e-01 | 0.703337854 |
| *Arrb2* | 0.0729354841 | 2.663724e-01 | 6.057757e-01 | 0.744238660 |
| *Calcr* | -0.0871350681 | 3.381504e-01 | 5.608988e-01 | 0.712749735 |
| *Calcr-1a* | -0.2265118942 | 1.097349e+00 | 2.948485e-01 | 0.595623252 |
| *Calcr-1b* | -0.2935603735 | 2.515188e+00 | 1.127542e-01 | 0.472499181 |
| *Cart* | 0.1331318801 | 8.877999e-01 | 3.460742e-01 | 0.595623252 |
| *Foxo1* | 0.0185677878 | 1.756607e-02 | 8.945595e-01 | 0.961651467 |
| *Gsk3a* | 0.1162611261 | 8.697849e-01 | 3.510149e-01 | 0.595623252 |
| *Gsk3b* | 0.0951828331 | 6.456184e-01 | 4.216836e-01 | 0.625254964 |
| *Hcrt* | -0.3869733108 | 6.411645e+00 | 1.133743e-02 | 0.097501920 |
| *Hdac5* | 0.1294596154 | 9.582552e-01 | 3.276269e-01 | 0.595623252 |
| *Hdc* | -0.2177386873 | 1.664973e+00 | 1.969332e-01 | 0.472499181 |
| *Hrh1* | 0.0430210744 | 8.577122e-02 | 7.696236e-01 | 0.911969778 |
| *Amylin* | -1.3720112998 | 1.356339e+01 | 2.306403e-04 | 0.004958767 |
| *Ins1* | -1.0153375712 | 2.928757e+00 | 8.701444e-02 | 0.415735681 |
| *Ins2* | 1.5083622948 | 1.201394e+01 | 5.280417e-04 | 0.007568597 |
| *Ir* | 0.0820140448 | 3.335613e-01 | 5.635696e-01 | 0.712749735 |
| *Irs1* | 0.2139571810 | 1.824810e+00 | 1.767418e-01 | 0.472499181 |
| *Irs2* | 0.1977478332 | 1.953327e+00 | 1.622288e-01 | 0.472499181 |
| *Jak2* | 0.2294886253 | 3.745120e+00 | 5.296191e-02 | 0.347687141 |
| *Lepr* | 0.0364439540 | 6.387244e-02 | 8.004769e-01 | 0.911969778 |
| *Lepr-b* | -0.1662067821 | 9.091135e-01 | 3.403497e-01 | 0.595623252 |
| *Mc4r* | 0.2540877179 | 3.075823e+00 | 7.946436e-02 | 0.415735681 |
| *Mch* | -0.1394499831 | 8.373900e-01 | 3.601443e-01 | 0.595623252 |
| *Npy* | 0.0996265026 | 4.401345e-01 | 5.070575e-01 | 0.703337854 |
| *Ptp1b* | 0.2673151139 | 3.634305e+00 | 5.660023e-02 | 0.347687141 |
| *Pde3b* | 0.1710071116 | 1.447413e+00 | 2.289435e-01 | 0.492228462 |
| *Pdk1* | 0.0858179449 | 3.712054e-01 | 5.423479e-01 | 0.712749735 |
| *Pias3* | 0.0001105497 | 4.700973e-07 | 9.994529e-01 | 0.999452941 |
| *Pik3ca* | 0.1626596041 | 1.795376e+00 | 1.802725e-01 | 0.472499181 |
| *Pik3r1* | 0.1560060130 | 1.940821e+00 | 1.635794e-01 | 0.472499181 |
| *Pomc* | 0.1343295541 | 6.830405e-01 | 4.085417e-01 | 0.625254964 |
| *Ramp1* | -0.0342513873 | 5.661694e-02 | 8.119252e-01 | 0.911969778 |
| *Ramp2* | 0.2100837000 | 2.159693e+00 | 1.416730e-01 | 0.472499181 |
| *Ramp3* | -0.1149817666 | 6.456865e-01 | 4.216591e-01 | 0.625254964 |
| *Socs3* | 0.0647433580 | 4.768852e-02 | 8.271354e-01 | 0.911969778 |
| *Stat3* | 0.1769802983 | 1.523860e+00 | 2.170363e-01 | 0.491187349 |
| *c-fos* | -0.6694505205 | 1.753817e+01 | 2.815966e-05 | 0.001210865 |
| *mTor* | -0.0099507377 | 4.958806e-03 | 9.438603e-01 | 0.969840395 |

### T1 Midbrain: HOM vs NON (AM)

| **Gene** | **log_2_ FC** | **Likelihood-ratio test** | **p value** | **q value** |
| --- | --- | --- | --- | --- |
| *Adrbk1* | 0.017019391 | 1.189290e-02 | 9.131592e-01 | 0.986808366 |
| *Agrp* | -0.059885747 | 5.585851e-02 | 8.131657e-01 | 0.986808366 |
| *Akt1* | 0.008084981 | 2.597795e-03 | 9.593506e-01 | 0.986808366 |
| *Akt2* | -0.002906207 | 9.854757e-01 | 9.854757e-01 | 0.986808366 |
| *Arrb1* | 0.002762724 | 2.733737e-04 | 9.868084e-01 | 0.986808366 |
| *Arrb2* | -0.012826923 | 6.911895e-03 | 9.337420e-01 | 0.986808366 |
| *Calcr* | 0.118539461 | 5.590427e-01 | 4.546462e-01 | 0.986808366 |
| *Calcr-1a* | 0.117198912 | 2.702094e-01 | 6.031913e-01 | 0.986808366 |
| *Calcr-1b* | -0.090248576 | 2.099728e-01 | 6.467888e-01 | 0.986808366 |
| *Cart* | 0.335553082 | 4.815584e+00 | 2.820352e-02 | 0.303187823 |
| *Foxo1* | -0.016972290 | 1.210150e-02 | 9.124040e-01 | 0.986808366 |
| *Gsk3a* | -0.075304440 | 2.053510e-01 | 6.504359e-01 | 0.986808366 |
| *Gsk3b* | 0.007416038 | 2.095420e-03 | 9.634890e-01 | 0.986808366 |
| *Hcrt* | 0.124559347 | 6.157715e-01 | 4.326230e-01 | 0.986808366 |
| *Hdac5* | -0.046323787 | 8.629425e-02 | 7.689422e-01 | 0.986808366 |
| *Hdc* | 0.090724096 | 2.590962e-01 | 6.107417e-01 | 0.986808366 |
| *Hrh1* | 0.018801671 | 1.446445e-02 | 9.042707e-01 | 0.986808366 |
| *Amylin* | -2.806905498 | 4.924034e+00 | 2.648560e-02 | 0.303187823 |
| *Ins1* | -4.352934363 | 1.763562e+01 | 2.675309e-05 | 0.001150383 |
| *Ins2* | -1.615468698 | 5.286244e+00 | 2.149453e-02 | 0.303187823 |
| *Ir* | -0.109717550 | 5.170035e-01 | 4.721227e-01 | 0.986808366 |
| *Irs1* | -0.228141981 | 1.789509e+00 | 1.809860e-01 | 0.986808366 |
| *Irs2* | 0.045845410 | 9.122414e-02 | 7.626266e-01 | 0.986808366 |
| *Jak2* | 0.064448166 | 1.573572e-01 | 6.916015e-01 | 0.986808366 |
| *Lepr* | 0.026941014 | 3.046558e-02 | 8.614380e-01 | 0.986808366 |
| *Lepr-b* | -0.105450762 | 3.299257e-01 | 5.657029e-01 | 0.986808366 |
| *Mc4r* | 0.162334763 | 1.074006e+00 | 3.000424e-01 | 0.986808366 |
| *Mch* | 0.116264758 | 8.977822e-01 | 3.433771e-01 | 0.986808366 |
| *Npy* | -0.143493190 | 7.685731e-01 | 3.806588e-01 | 0.986808366 |
| *Ptp1b* | 0.017691603 | 1.313892e-02 | 9.087423e-01 | 0.986808366 |
| *Pde3b* | -0.039516648 | 6.686442e-02 | 7.959581e-01 | 0.986808366 |
| *Pdk1* | -0.135327647 | 7.689927e-01 | 3.805289e-01 | 0.986808366 |
| *Pias3* | -0.120101869 | 5.970689e-01 | 4.396986e-01 | 0.986808366 |
| *Pik3ca* | -0.008027417 | 2.388079e-03 | 9.610245e-01 | 0.986808366 |
| *Pik3r1* | 0.101265519 | 6.787332e-01 | 4.100233e-01 | 0.986808366 |
| *Pomc* | 0.309524022 | 3.135877e+00 | 7.658719e-02 | 0.548874865 |
| *Ramp1* | 0.003797067 | 6.151064e-04 | 9.802134e-01 | 0.986808366 |
| *Ramp2* | 0.009014914 | 3.512292e-03 | 9.527414e-01 | 0.986808366 |
| *Ramp3* | 0.171011962 | 1.259950e+00 | 2.616605e-01 | 0.986808366 |
| *Socs3* | -0.197030185 | 4.806650e-01 | 4.881213e-01 | 0.986808366 |
| *Stat3* | -0.010067422 | 4.452015e-03 | 9.468019e-01 | 0.986808366 |
| *c-fos* | -0.319273362 | 4.271482e+00 | 3.875711e-02 | 0.333311120 |
| *mTor* | -0.161155350 | 1.094444e+00 | 2.954886e-01 | 0.986808366 |

### T1 Midbrain: HOM vs NON (PM)

| **Gene** | **log_2_ FC** | **Likelihood-ratio test** | **p value** | **q value** |
| --- | --- | --- | --- | --- |
| *Adrbk1* | 0.088827651 | 4.062487e-01 | 5.238797e-01 | 7.266718e-01 |
| *Agrp* | -0.927814285 | 1.750937e+01 | 2.858952e-05 | 4.097832e-04 |
| *Akt1* | -0.047843718 | 1.098864e-01 | 7.402735e-01 | 8.842156e-01 |
| *Akt2* | 0.011022652 | 5.695594e-03 | 9.398414e-01 | 9.924922e-01 |
| *Arrb1* | 0.136746613 | 1.065399e+00 | 3.019870e-01 | 6.738429e-01 |
| *Arrb2* | 0.242907654 | 3.607684e+00 | 5.751316e-02 | 2.473066e-01 |
| *Calcr* | -0.155167170 | 1.063350e+00 | 3.024524e-01 | 6.738429e-01 |
| *Calcr-1a* | 0.001784709 | 6.564499e-05 | 9.935355e-01 | 9.935355e-01 |
| *Calcr-1b* | -0.261908267 | 2.010601e+00 | 1.562034e-01 | 5.083682e-01 |
| *Cart* | -0.459843462 | 1.024372e+01 | 1.371506e-03 | 9.829129e-03 |
| *Foxo1* | 0.061471608 | 1.917338e-01 | 6.614775e-01 | 8.842156e-01 |
| *Gsk3a* | 0.113308645 | 8.218521e-01 | 3.646392e-01 | 6.738429e-01 |
| *Gsk3b* | 0.044606184 | 1.315502e-01 | 7.168301e-01 | 8.842156e-01 |
| *Hcrt* | -0.353687058 | 5.364103e+00 | 2.055535e-02 | 9.820890e-02 |
| *Hdac5* | -0.008122641 | 3.214708e-03 | 9.547855e-01 | 9.924922e-01 |
| *Hdc* | -0.432304559 | 6.308547e+00 | 1.201572e-02 | 6.458452e-02 |
| *Hrh1* | 0.020424566 | 1.937836e-02 | 8.892872e-01 | 9.924922e-01 |
| *Amylin* | -0.306245606 | 1.013580e+00 | 3.140468e-01 | 6.738429e-01 |
| *Ins1* | -3.209954644 | 1.027318e+01 | 1.349781e-03 | 9.829129e-03 |
| *Ins2* | 0.854197145 | 2.710678e+00 | 9.967863e-02 | 3.896528e-01 |
| *Ir* | 0.050839039 | 1.280543e-01 | 7.204580e-01 | 8.842156e-01 |
| *Irs1* | 0.015970984 | 9.844170e-03 | 9.209653e-01 | 9.924922e-01 |
| *Irs2* | 0.007602957 | 2.860676e-03 | 9.573453e-01 | 9.924922e-01 |
| *Jak2* | 0.145944281 | 1.410288e+00 | 2.350086e-01 | 6.736914e-01 |
| *Lepr* | 0.138604344 | 9.295833e-01 | 3.349712e-01 | 6.738429e-01 |
| *Lepr-b* | -0.148549347 | 7.273096e-01 | 3.937566e-01 | 6.738429e-01 |
| *Mc4r* | -0.115015059 | 6.131681e-01 | 4.335974e-01 | 6.738429e-01 |
| *Mch* | -0.121465737 | 6.357893e-01 | 4.252397e-01 | 6.738429e-01 |
| *Npy* | 0.818728581 | 3.198350e+01 | 1.554874e-08 | 3.342978e-07 |
| *Ptp1b* | 0.141674513 | 1.015121e+00 | 3.136791e-01 | 6.738429e-01 |
| *Pde3b* | 0.133905792 | 8.864022e-01 | 3.464542e-01 | 6.738429e-01 |
| *Pdk1* | 0.204563103 | 2.117731e+00 | 1.456021e-01 | 5.083682e-01 |
| *Pias3* | 0.199658560 | 1.923101e+00 | 1.655152e-01 | 5.083682e-01 |
| *Pik3ca* | 0.087489571 | 4.653496e-01 | 4.951344e-01 | 7.096926e-01 |
| *Pik3r1* | 0.088888576 | 5.994671e-01 | 4.387814e-01 | 6.738429e-01 |
| *Pomc* | -0.553231241 | 1.039615e+01 | 1.262782e-03 | 9.829129e-03 |
| *Ramp1* | 0.005583875 | 1.470495e-03 | 9.694110e-01 | 9.924922e-01 |
| *Ramp2* | 0.144432406 | 1.017737e+00 | 3.130564e-01 | 6.738429e-01 |
| *Ramp3* | -0.122973115 | 7.388978e-01 | 3.900142e-01 | 6.738429e-01 |
| *Socs3* | 2.063685028 | 8.362333e+01 | 0.000000e+00 | 0.000000e+00 |
| *Stat3* | 0.408044871 | 8.183316e+00 | 4.227740e-03 | 2.597041e-02 |
| *c-fos* | -0.112969413 | 5.173073e-01 | 4.719926e-01 | 6.998511e-01 |
| *mTor* | 0.051562962 | 1.337269e-01 | 7.145988e-01 | 8.842156e-01 |

### T1 Left Cortex: HEM vs NON (PM)

| **Gene** | **log_2_ FC** | **Likelihood-ratio test** | **p value** | **q value** |
| --- | --- | --- | --- | --- |
| *Adrbk1* | 0.292975218 | 2.523359e+00 | 1.121714e-01 | 0.321557990 |
| *Agrp* | -0.110616581 | 4.625864e-02 | 8.297063e-01 | 1.000000000 |
| *Akt1* | 0.079919613 | 1.877147e-01 | 6.648255e-01 | 1.000000000 |
| *Akt2* | 0.186032398 | 1.043031e+00 | 3.071176e-01 | 0.695055553 |
| *Arrb1* | 0.060701884 | 9.695726e-02 | 7.555121e-01 | 1.000000000 |
| *Arrb2* | -0.087276746 | 2.191090e-01 | 6.397197e-01 | 1.000000000 |
| *Calcr* | -3.090268065 | 1.194219e+01 | 5.487704e-04 | 0.005899281 |
| *Calcr-1a* | -16.082379111 | 0.000000e+00 | 1.000000e+00 | 1.000000000 |
| *Calcr-1b* | -0.508353887 | 2.540550e-01 | 6.142342e-01 | 1.000000000 |
| *Cart* | 0.702720269 | 7.835294e+00 | 5.123581e-03 | 0.036718994 |
| *Foxo1* | -0.638594721 | 4.064661e+00 | 4.378947e-02 | 0.188294713 |
| *Gsk3a* | 0.016649093 | 6.784063e-03 | 9.343561e-01 | 1.000000000 |
| *Gsk3b* | 0.002009041 | 9.985626e-05 | 9.920270e-01 | 1.000000000 |
| *Hcrt* | 0.356746616 | 4.588691e-01 | 4.981530e-01 | 0.944410463 |
| *Hdac5* | 0.300946486 | 2.758693e+00 | 9.672723e-02 | 0.319943914 |
| *Hdc* | 0.251885871 | 6.057440e-01 | 4.363948e-01 | 0.893570281 |
| *Hrh1* | 0.356756114 | 4.662360e+00 | 3.083078e-02 | 0.147302637 |
| *Amylin* | -10.587804553 | 1.351355e-01 | 7.131657e-01 | 1.000000000 |
| *Ins1* | -1.288498335 | 4.164238e-01 | 5.187269e-01 | 0.944410463 |
| *Ins2* | 0.467786929 | 1.851217e-02 | 8.917743e-01 | 1.000000000 |
| *Ir* | 0.108975039 | 3.825504e-01 | 5.362414e-01 | 0.944410463 |
| *Irs1* | 0.659667999 | 1.278863e+01 | 3.487321e-04 | 0.005495600 |
| *Irs2* | 0.538576014 | 1.001664e+01 | 1.551326e-03 | 0.013341401 |
| *Jak2* | 0.030959590 | 2.544315e-02 | 8.732678e-01 | 1.000000000 |
| *Lepr* | -0.646473563 | 1.261134e+01 | 3.834139e-04 | 0.005495600 |
| *Lepr-b* | -0.884165822 | 1.586140e+01 | 6.815477e-05 | 0.002930655 |
| *Mc4r* | -0.439035312 | 2.416810e+00 | 1.200392e-01 | 0.322605306 |
| *Mch* | -0.755369514 | 5.793217e+00 | 1.608812e-02 | 0.086473638 |
| *Npy* | -0.264394922 | 1.273684e+00 | 2.590767e-01 | 0.618905402 |
| *Ptp1b* | 0.341770960 | 3.588528e+00 | 5.817974e-02 | 0.227429910 |
| *Pde3b* | -0.047644316 | 7.381911e-02 | 7.858552e-01 | 1.000000000 |
| *Pdk1* | -0.252528539 | 1.730959e+00 | 1.882885e-01 | 0.476259102 |
| *Pias3* | 0.013801440 | 6.446836e-03 | 9.360049e-01 | 1.000000000 |
| *Pik3ca* | 0.054116025 | 7.684076e-02 | 7.816253e-01 | 1.000000000 |
| *Pik3r1* | -0.006761401 | 1.258454e-03 | 9.717012e-01 | 1.000000000 |
| *Pomc* | -0.669540918 | 3.273006e+00 | 7.042848e-02 | 0.252368711 |
| *Ramp1* | -0.076823555 | 1.728097e-01 | 6.776267e-01 | 1.000000000 |
| *Ramp2* | 0.416253986 | 6.033558e+00 | 1.403642e-02 | 0.086223693 |
| *Ramp3* | -0.167331211 | 9.164817e-01 | 3.384005e-01 | 0.727560978 |
| *Socs3* | 0.706126877 | 2.549078e+00 | 1.103587e-01 | 0.321557990 |
| *Stat3* | -0.019994360 | 9.549877e-03 | 9.221519e-01 | 1.000000000 |
| *c-fos* | 0.006265557 | 3.909242e-04 | 9.842254e-01 | 1.000000000 |
| *mTor* | 0.108933147 | 3.589754e-01 | 5.490759e-01 | 0.944410463 |

### T1 Left Cortex: HOM vs NON (AM)

| **Gene** | **log_2_ FC** | **Likelihood-ratio test** | **p value** | **q value** |
| --- | --- | --- | --- | --- |
| *Adrbk1* | 0.07002509 | 0.18797949 | 0.664603614 | 0.8218156 |
| *Agrp* | 0.35305993 | 0.65368959 | 0.418796635 | 0.8119286 |
| *Akt1* | -0.05835741 | 0.13304159 | 0.715299022 | 0.8218156 |
| *Akt2* | 0.02544943 | 0.02528467 | 0.873659780 | 0.8889275 |
| *Arrb1* | 0.10953867 | 0.76716112 | 0.381096718 | 0.8119286 |
| *Arrb2* | 0.08927872 | 0.31742229 | 0.573160888 | 0.8218156 |
| *Calcr* | 0.71082523 | 0.76296015 | 0.382403735 | 0.8119286 |
| *Calcr-1a* | 0.00000000 | 0.17122130 | 0.679028672 | 0.8218156 |
| *Calcr-1b* | 26.84144333 | 6.89454798 | 0.008645902 | 0.3717738 |
| *Cart* | -0.25586815 | 2.42030142 | 0.119771883 | 0.7821619 |
| *Foxo1* | -0.12467881 | 0.59908239 | 0.438928349 | 0.8119286 |
| *Gsk3a* | 0.09464328 | 0.58986485 | 0.442471387 | 0.8119286 |
| *Gsk3b* | 0.05024633 | 0.08976223 | 0.764479648 | 0.8218156 |
| *Hcrt* | -0.68471924 | 2.32477444 | 0.127328676 | 0.7821619 |
| *Hdac5* | 0.15994998 | 1.13662769 | 0.286366042 | 0.8119286 |
| *Hdc* | 0.03748813 | 0.01950533 | 0.888927468 | 0.8889275 |
| *Hrh1* | 0.05036255 | 0.09648327 | 0.756091376 | 0.8218156 |
| *Amylin* | 0.45151795 | 0.23976259 | 0.624377640 | 0.8218156 |
| *Ins1* | 1.48006051 | 1.36428458 | 0.242796305 | 0.8119286 |
| *Ins2* | -18.87830846 | 0.51716975 | 0.472051489 | 0.8119286 |
| *Ir* | 0.15982572 | 1.00223707 | 0.316769808 | 0.8119286 |
| *Irs1* | -0.09073679 | 0.27531393 | 0.599789211 | 0.8218156 |
| *Irs2* | 0.10945142 | 0.47119245 | 0.492439095 | 0.8144185 |
| *Jak2* | 0.17624891 | 1.18731827 | 0.275871161 | 0.8119286 |
| *Lepr* | 0.20405601 | 1.60539343 | 0.205140554 | 0.8119286 |
| *Lepr-b* | 0.06133675 | 0.11147261 | 0.738473779 | 0.8218156 |
| *Mc4r* | 0.27494957 | 2.65450904 | 0.103256633 | 0.7821619 |
| *Mch* | 0.28645509 | 1.70793204 | 0.191253898 | 0.8119286 |
| *Npy* | 0.07117522 | 0.33721042 | 0.561443862 | 0.8218156 |
| *Ptp1b* | 0.16834172 | 1.09671149 | 0.294988928 | 0.8119286 |
| *Pde3b* | 0.05913441 | 0.13614279 | 0.712146113 | 0.8218156 |
| *Pdk1* | 0.09903082 | 0.37552658 | 0.540007113 | 0.8218156 |
| *Pias3* | 0.06834152 | 0.17913575 | 0.672117022 | 0.8218156 |
| *Pik3ca* | 0.09235582 | 0.53647695 | 0.463896469 | 0.8119286 |
| *Pik3r1* | 0.10952565 | 0.73833602 | 0.390194431 | 0.8119286 |
| *Pomc* | 0.06179943 | 0.05085057 | 0.821589778 | 0.8616673 |
| *Ramp1* | -0.13759874 | 0.74792861 | 0.387132839 | 0.8119286 |
| *Ramp2* | 0.35476356 | 4.86245930 | 0.027447185 | 0.5901145 |
| *Ramp3* | -0.13961794 | 0.72854665 | 0.393354683 | 0.8119286 |
| *Socs3* | 0.46861201 | 3.97096093 | 0.046291348 | 0.6635093 |
| *Stat3* | 0.15424465 | 0.94184259 | 0.331804425 | 0.8119286 |
| *c-fos* | -0.25271064 | 2.41890551 | 0.119878661 | 0.7821619 |
| *mTor* | 0.06634816 | 0.17422641 | 0.676382659 | 0.8218156 |

### T1 Left Cortex: HOM vs NON (PM)

| **Gene** | **log_2_ FC** | **Likelihood-ratio test** | **p value** | **q value** |
| --- | --- | --- | --- | --- |
| *Adrbk1* | 0.0140011421 | 9.097154e-03 | 9.240138e-01 | 9.996639e-01 |
| *Agrp* | 0.4568297260 | 2.002321e+00 | 1.570586e-01 | 6.343841e-01 |
| *Akt1* | -0.0710836988 | 2.906567e-01 | 5.898000e-01 | 9.996639e-01 |
| *Akt2* | -0.1392946802 | 9.141339e-01 | 3.390199e-01 | 8.750857e-01 |
| *Arrb1* | 0.1621276673 | 1.107018e+00 | 2.927312e-01 | 8.750857e-01 |
| *Arrb2* | 0.1319655735 | 8.161272e-01 | 3.663149e-01 | 8.750857e-01 |
| *Calcr* | -0.5981140731 | 2.192484e+00 | 1.386855e-01 | 6.343841e-01 |
| *Calcr-1a* | -2.2437341620 | 1.774346e-07 | 9.996639e-01 | 9.996639e-01 |
| *Calcr-1b* | -0.1407199184 | 4.991002e-02 | 8.232199e-01 | 9.996639e-01 |
| *Cart* | -0.4058556755 | 3.786394e+00 | 5.167084e-02 | 5.554615e-01 |
| *Foxo1* | 0.1062253611 | 1.960796e-01 | 6.579040e-01 | 9.996639e-01 |
| *Gsk3a* | 0.0674960347 | 1.824337e-01 | 6.692900e-01 | 9.996639e-01 |
| *Gsk3b* | 0.0071337916 | 2.887916e-03 | 9.571428e-01 | 9.996639e-01 |
| *Hcrt* | 0.0313216843 | 6.085191e-03 | 9.378220e-01 | 9.996639e-01 |
| *Hdac5* | -0.0244742634 | 3.183345e-02 | 8.583937e-01 | 9.996639e-01 |
| *Hdc* | -0.1231366168 | 2.388057e-01 | 6.250700e-01 | 9.996639e-01 |
| *Hrh1* | 0.0304130357 | 5.266195e-02 | 8.184944e-01 | 9.996639e-01 |
| *Amylin* | 2.1089872233 | 2.649982e+00 | 1.035511e-01 | 6.343841e-01 |
| *Ins1* | 0.1252953343 | 1.891709e-02 | 8.906044e-01 | 9.996639e-01 |
| *Ins2* | 0.3012978974 | 1.509111e-02 | 9.022292e-01 | 9.996639e-01 |
| *Ir* | 0.2087847129 | 2.256015e+00 | 1.330961e-01 | 6.343841e-01 |
| *Irs1* | 0.0168922143 | 1.263542e-02 | 9.105004e-01 | 9.996639e-01 |
| *Irs2* | 0.1068922086 | 6.012001e-01 | 4.381205e-01 | 9.419591e-01 |
| *Jak2* | -0.0199554010 | 1.712196e-02 | 8.958933e-01 | 9.996639e-01 |
| *Lepr* | 0.2791638620 | 4.216805e+00 | 4.002547e-02 | 5.554615e-01 |
| *Lepr-b* | -0.0874708259 | 3.272992e-01 | 5.672538e-01 | 9.996639e-01 |
| *Mc4r* | 0.1939819672 | 8.269933e-01 | 3.631434e-01 | 8.750857e-01 |
| *Mch* | -0.0990077218 | 2.140170e-01 | 6.436370e-01 | 9.996639e-01 |
| *Npy* | 0.2534845685 | 1.952810e+00 | 1.622843e-01 | 6.343841e-01 |
| *Ptp1b* | -0.0008547828 | 3.098332e-05 | 9.955588e-01 | 9.996639e-01 |
| *Pde3b* | 0.0461472939 | 1.124287e-01 | 7.373958e-01 | 9.996639e-01 |
| *Pdk1* | -0.0014069770 | 8.676782e-05 | 9.925679e-01 | 9.996639e-01 |
| *Pias3* | 0.1613357923 | 1.428630e+00 | 2.319882e-01 | 7.927967e-01 |
| *Pik3ca* | 0.0489970102 | 1.001008e-01 | 7.517087e-01 | 9.996639e-01 |
| *Pik3r1* | -0.0185899146 | 1.585396e-02 | 8.998012e-01 | 9.996639e-01 |
| *Pomc* | -0.2929760920 | 1.382459e+00 | 2.396827e-01 | 7.927967e-01 |
| *Ramp1* | -0.2204854923 | 2.528195e+00 | 1.118281e-01 | 6.343841e-01 |
| *Ramp2* | 0.2036149419 | 2.238928e+00 | 1.345743e-01 | 6.343841e-01 |
| *Ramp3* | -0.1395999314 | 1.050929e+00 | 3.052932e-01 | 8.750857e-01 |
| *Socs3* | 1.6587287081 | 2.399554e+01 | 9.655910e-07 | 4.152041e-05 |
| *Stat3* | 0.4062707342 | 6.441475e+00 | 1.114861e-02 | 2.396950e-01 |
| *c-fos* | -0.2147840748 | 7.121651e-01 | 3.987260e-01 | 9.023799e-01 |
| *mTor* | 0.0545021999 | 1.431755e-01 | 7.051444e-01 | 9.996639e-01 |

### T1 Right Cortex: HEM vs NON (PM)

| **Gene** | **log_2_ FC** | **Likelihood-ratio test** | **p value** | **q value** |
| --- | --- | --- | --- | --- |
| *Adrbk1* | 0.325547436 | 4.270336e+00 | 3.878327e-02 | 0.1667680508 |
| *Agrp* | 0.448178036 | 1.256486e+00 | 2.623172e-01 | 0.6635082395 |
| *Akt1* | 0.194302910 | 1.732298e+00 | 1.881177e-01 | 0.5777899381 |
| *Akt2* | 0.216132033 | 1.832082e+00 | 1.758818e-01 | 0.5777899381 |
| *Arrb1* | 0.237018821 | 2.352350e+00 | 1.250943e-01 | 0.4482545615 |
| *Arrb2* | 0.072760117 | 2.451949e-01 | 6.204790e-01 | 0.8709918473 |
| *Calcr* | -0.268737627 | 1.511599e-01 | 6.974293e-01 | 0.8709918473 |
| *Calcr-1a* | 0.000000000 | 7.390213e-10 | 9.999783e-01 | 0.9999783096 |
| *Calcr-1b* | 1.152005532 | 1.261397e+00 | 2.613868e-01 | 0.6635082395 |
| *Cart* | -0.084153573 | 9.723398e-02 | 7.551746e-01 | 0.8709918473 |
| *Foxo1* | -0.130299818 | 7.032863e-01 | 4.016816e-01 | 0.6977966228 |
| *Gsk3a* | 0.049266958 | 9.691981e-02 | 7.555578e-01 | 0.8709918473 |
| *Gsk3b* | 0.024235456 | 2.421237e-02 | 8.763458e-01 | 0.9662273975 |
| *Hcrt* | -0.016469362 | 2.050445e-03 | 9.638827e-01 | 0.9868322534 |
| *Hdac5* | 0.321487509 | 4.745140e+00 | 2.938117e-02 | 0.1403766950 |
| *Hdc* | 0.192219619 | 6.449513e-01 | 4.219235e-01 | 0.6977966228 |
| *Hrh1* | 0.516432157 | 1.486461e+01 | 1.155097e-04 | 0.0016556388 |
| *Amylin* | -0.378558995 | 1.864551e-01 | 6.658835e-01 | 0.8709918473 |
| *Ins1* | 0.772427800 | 4.327879e-01 | 5.106242e-01 | 0.7841728055 |
| *Ins2* | -0.755344735 | 8.570220e-02 | 7.697137e-01 | 0.8709918473 |
| *Ir* | 0.139406839 | 9.920916e-01 | 3.192317e-01 | 0.6977966228 |
| *Irs1* | 0.345087222 | 6.131818e+00 | 1.327703e-02 | 0.1141824312 |
| *Irs2* | 0.659678177 | 2.213601e+01 | 2.540005e-06 | 0.0001092202 |
| *Jak2* | 0.148190863 | 9.207582e-01 | 3.372760e-01 | 0.6977966228 |
| *Lepr* | -0.009047078 | 2.868599e-03 | 9.572863e-01 | 0.9868322534 |
| *Lepr-b* | -0.393272403 | 5.246703e+00 | 2.198840e-02 | 0.1181876530 |
| *Mc4r* | 0.488624964 | 5.261658e+00 | 2.180025e-02 | 0.1181876530 |
| *Mch* | -0.099465254 | 2.030671e-01 | 6.522565e-01 | 0.8709918473 |
| *Npy* | -0.173949666 | 7.734092e-01 | 3.791645e-01 | 0.6977966228 |
| *Ptp1b* | 0.395671934 | 7.208253e+00 | 7.256910e-03 | 0.0780117865 |
| *Pde3b* | 0.042634964 | 9.514494e-02 | 7.577356e-01 | 0.8709918473 |
| *Pdk1* | 0.007530669 | 3.933067e-03 | 9.499941e-01 | 0.9868322534 |
| *Pias3* | 0.123944985 | 8.308131e-01 | 3.620375e-01 | 0.6977966228 |
| *Pik3ca* | 0.166780950 | 1.152882e+00 | 2.829466e-01 | 0.6759278871 |
| *Pik3r1* | -0.114032096 | 7.501875e-01 | 3.864169e-01 | 0.6977966228 |
| *Pomc* | -0.118107702 | 1.370814e-01 | 7.111999e-01 | 0.8709918473 |
| *Ramp1* | -0.076634055 | 3.908017e-01 | 5.318783e-01 | 0.7886471576 |
| *Ramp2* | 0.671288265 | 1.735756e+01 | 3.096646e-05 | 0.0006657789 |
| *Ramp3* | -0.252070543 | 5.651328e+00 | 1.744216e-02 | 0.1181876530 |
| *Socs3* | 0.346874888 | 6.496504e-01 | 4.202377e-01 | 0.6977966228 |
| *Stat3* | 0.122975577 | 5.277506e-01 | 4.675541e-01 | 0.7446232146 |
| *c-fos* | -0.522678802 | 3.456885e+00 | 6.298888e-02 | 0.2462292531 |
| *mTor* | 0.166356750 | 1.318233e+00 | 2.509094e-01 | 0.6635082395 |

### T1 Right Cortex: HOM vs NON (AM)

| **Gene** | **log_2_ FC** | **Likelihood-ratio test** | **p value** | **q value** |
| --- | --- | --- | --- | --- |
| *Adrbk1* | -6.333832e-04 | 2.783318e-05 | 0.9957906072 | 0.995790607 |
| *Agrp* | 4.799232e-01 | 1.462233e+00 | 0.2265751200 | 0.854632861 |
| *Akt1* | 1.610392e-02 | 1.009745e-02 | 0.9199584409 | 0.964834462 |
| *Akt2* | 3.381237e-02 | 4.530517e-02 | 0.8314438679 | 0.964834462 |
| *Arrb1* | 6.148924e-02 | 1.330978e-01 | 0.7152414978 | 0.922620213 |
| *Arrb2* | 1.807243e-01 | 1.287594e+00 | 0.2564917725 | 0.854632861 |
| *Calcr* | -8.063631e-01 | 8.590755e-01 | 0.3539975310 | 0.895405520 |
| *Calcr-1a* | 2.112168e+01 | 1.498487e-01 | 0.6986800324 | 0.922620213 |
| *Calcr-1b* | -1.022554e+00 | 1.312288e+00 | 0.2519807694 | 0.854632861 |
| *Cart* | 9.214310e-02 | 3.278412e-01 | 0.5669330702 | 0.922620213 |
| *Foxo1* | 2.362635e-01 | 2.206800e+00 | 0.1374034030 | 0.854632861 |
| *Gsk3a* | 1.879139e-02 | 1.186727e-02 | 0.9132524577 | 0.964834462 |
| *Gsk3b* | 2.503346e-02 | 2.202817e-02 | 0.8820121907 | 0.964834462 |
| *Hcrt* | -4.249501e-01 | 1.376228e+00 | 0.2407447061 | 0.854632861 |
| *Hdac5* | 2.356935e-01 | 2.137869e+00 | 0.1437013641 | 0.854632861 |
| *Hdc* | -7.119542e-02 | 7.732395e-02 | 0.7809572156 | 0.959461722 |
| *Hrh1* | 1.794215e-01 | 1.243947e+00 | 0.2647118150 | 0.854632861 |
| *Amylin* | 3.072036e-01 | 5.167287e-02 | 0.8201775101 | 0.964834462 |
| *Ins1* | -1.137097e+01 | 1.770840e-01 | 0.6738912908 | 0.922620213 |
| *Ins2* | -1.530671e+00 | 2.398656e+00 | 0.1214395285 | 0.854632861 |
| *Ir* | 6.562276e-02 | 1.723656e-01 | 0.6780179161 | 0.922620213 |
| *Irs1* | -6.534883e-02 | 1.495920e-01 | 0.6989255708 | 0.922620213 |
| *Irs2* | 6.867423e-02 | 1.891556e-01 | 0.6636203520 | 0.922620213 |
| *Jak2* | 1.893809e-01 | 1.252248e+00 | 0.2631236300 | 0.854632861 |
| *Lepr* | 1.236687e-01 | 5.998513e-01 | 0.4386347607 | 0.922620213 |
| *Lepr-b* | 1.932483e-01 | 1.175605e+00 | 0.2782525594 | 0.854632861 |
| *Mc4r* | 6.234740e-01 | 1.414587e+01 | 0.0001691682 | 0.007274234 |
| *Mch* | 2.938296e-01 | 2.036502e+00 | 0.1535624159 | 0.854632861 |
| *Npy* | -1.126039e-02 | 4.206358e-03 | 0.9482883016 | 0.970866594 |
| *Ptp1b* | 1.613881e-01 | 9.896149e-01 | 0.3198365103 | 0.895405520 |
| *Pde3b* | 1.067966e-01 | 4.529392e-01 | 0.5009424895 | 0.922620213 |
| *Pdk1* | 6.313064e-02 | 1.948857e-01 | 0.6588809843 | 0.922620213 |
| *Pias3* | -5.502354e-02 | 1.195587e-01 | 0.7295136565 | 0.922620213 |
| *Pik3ca* | -2.202221e-02 | 2.044907e-02 | 0.8862900002 | 0.964834462 |
| *Pik3r1* | 1.285783e-01 | 6.340068e-01 | 0.4258894084 | 0.922620213 |
| *Pomc* | 1.513914e-01 | 4.677624e-01 | 0.4940183858 | 0.922620213 |
| *Ramp1* | 9.281207e-02 | 3.426332e-01 | 0.5583132338 | 0.922620213 |
| *Ramp2* | 2.315571e-01 | 2.108434e+00 | 0.1464891687 | 0.854632861 |
| *Ramp3* | -2.301988e-01 | 2.044604e+00 | 0.1527467604 | 0.854632861 |
| *Socs3* | 1.770223e-01 | 6.414229e-01 | 0.4231960137 | 0.922620213 |
| *Stat3* | 1.113364e-01 | 4.997287e-01 | 0.4796193536 | 0.922620213 |
| *c-fos* | -1.520379e-01 | 9.037148e-01 | 0.3417875727 | 0.895405520 |
| *mTor* | 1.027977e-01 | 4.231627e-01 | 0.5153631430 | 0.922620213 |

### T1 Right Cortex: HOM vs NON (PM)

| **Gene** | **log_2_ FC** | **Likelihood-ratio test** | **p value** | **q value** |
| --- | --- | --- | --- | --- |
| *Adrbk1* | 1.321409e-01 | 6.980895e-01 | 4.034264e-01 | 9.913753e-01 |
| *Agrp* | 9.230712e-01 | 6.157199e+00 | 1.308783e-02 | 1.875922e-01 |
| *Akt1* | 2.802039e-03 | 3.746695e-04 | 9.845568e-01 | 9.996778e-01 |
| *Akt2* | -1.249252e-01 | 6.013864e-01 | 4.380495e-01 | 9.913753e-01 |
| *Arrb1* | 3.074070e-01 | 3.968351e+00 | 4.636316e-02 | 2.901592e-01 |
| *Arrb2* | 9.038851e-02 | 3.785023e-01 | 5.384059e-01 | 9.996778e-01 |
| *Calcr* | 9.620993e-01 | 3.381966e+00 | 6.591337e-02 | 2.901592e-01 |
| *Calcr-1a* | 9.985775e+00 | 1.630878e-07 | 9.996778e-01 | 9.996778e-01 |
| *Calcr-1b* | 4.198500e-01 | 9.460388e-02 | 7.584039e-01 | 9.996778e-01 |
| *Cart* | -8.960533e-02 | 1.100390e-01 | 7.400997e-01 | 9.996778e-01 |
| *Foxo1* | -1.676270e-01 | 1.159818e+00 | 2.815033e-01 | 8.389944e-01 |
| *Gsk3a* | -7.708117e-06 | 3.582345e-07 | 9.995224e-01 | 9.996778e-01 |
| *Gsk3b* | -5.879071e-02 | 1.440620e-01 | 7.042758e-01 | 9.996778e-01 |
| *Hcrt* | -3.121031e-01 | 6.101297e-01 | 4.347390e-01 | 9.913753e-01 |
| *Hdac5* | 3.247011e-02 | 4.761777e-02 | 8.272616e-01 | 9.996778e-01 |
| *Hdc* | -4.118254e-01 | 2.434354e+00 | 1.187028e-01 | 4.253516e-01 |
| *Hrh1* | -4.887245e-02 | 1.283453e-01 | 7.201539e-01 | 9.996778e-01 |
| *Amylin* | -1.494625e-01 | 3.008735e-02 | 8.622922e-01 | 9.996778e-01 |
| *Ins1* | 1.711681e+00 | 3.584647e+00 | 5.831581e-02 | 2.901592e-01 |
| *Ins2* | -4.289002e-01 | 3.013927e-02 | 8.621746e-01 | 9.996778e-01 |
| *Ir* | 8.402109e-02 | 3.586651e-01 | 5.492485e-01 | 9.996778e-01 |
| *Irs1* | -1.223755e-02 | 7.489828e-03 | 9.310341e-01 | 9.996778e-01 |
| *Irs2* | 9.690067e-02 | 4.675989e-01 | 4.940938e-01 | 9.996778e-01 |
| *Jak2* | -1.388132e-01 | 7.978315e-01 | 3.717425e-01 | 9.913753e-01 |
| *Lepr* | -3.356745e-02 | 3.962450e-02 | 8.422166e-01 | 9.996778e-01 |
| *Lepr-b* | -1.831637e-01 | 1.155599e+00 | 2.823802e-01 | 8.389944e-01 |
| *Mc4r* | -8.269647e-03 | 1.436913e-03 | 9.697621e-01 | 9.996778e-01 |
| *Mch* | 9.592180e-03 | 1.895092e-03 | 9.652769e-01 | 9.996778e-01 |
| *Npy* | 3.576278e-01 | 3.343306e+00 | 6.747889e-02 | 2.901592e-01 |
| *Ptp1b* | -3.898021e-02 | 6.871796e-02 | 7.932127e-01 | 9.996778e-01 |
| *Pde3b* | -4.375397e-02 | 9.903454e-02 | 7.529913e-01 | 9.996778e-01 |
| *Pdk1* | -5.353904e-03 | 1.290675e-03 | 9.713414e-01 | 9.996778e-01 |
| *Pias3* | 1.000056e-01 | 5.397292e-01 | 4.625450e-01 | 9.944717e-01 |
| *Pik3ca* | -2.618148e-02 | 2.812571e-02 | 8.668136e-01 | 9.996778e-01 |
| *Pik3r1* | -8.507934e-02 | 3.258871e-01 | 5.680910e-01 | 9.996778e-01 |
| *Pomc* | 1.427102e-01 | 2.076420e-01 | 6.486219e-01 | 9.996778e-01 |
| *Ramp1* | -2.844030e-01 | 3.714634e+00 | 5.393746e-02 | 2.901592e-01 |
| *Ramp2* | 2.772765e-01 | 2.918315e+00 | 8.757926e-02 | 3.423553e-01 |
| *Ramp3* | -2.538561e-01 | 3.629165e+00 | 5.677530e-02 | 2.901592e-01 |
| *Socs3* | 1.996754e+00 | 2.271423e+01 | 1.879708e-06 | 8.082746e-05 |
| *Stat3* | 4.980409e-01 | 8.781934e+00 | 3.042286e-03 | 6.540914e-02 |
| *c-fos* | -5.981645e-01 | 4.497803e+00 | 3.393843e-02 | 2.901592e-01 |
| *mTor* | 1.526193e-01 | 1.107287e+00 | 2.926725e-01 | 8.389944e-01 |

### T2 Hindbrain: HEM vs NON (AM)

| **Gene** | **log_2_ FC** | **Likelihood-ratio test** | **p value** | **q value** |
| --- | --- | --- | --- | --- |
| *Adrbk1* | 0.030194834 | 3.558043e-02 | 0.8503845710 | 0.993424844 |
| *Agrp* | 0.354756333 | 9.494897e-01 | 0.3298492160 | 0.993424844 |
| *Akt1* | -0.117314636 | 1.096051e+00 | 0.2951343737 | 0.993424844 |
| *Akt2* | 0.061748535 | 1.523150e-01 | 0.6963327929 | 0.993424844 |
| *Arrb1* | 0.112556747 | 4.696737e-01 | 0.4931373057 | 0.993424844 |
| *Arrb2* | 0.094750541 | 3.693219e-01 | 0.5433741052 | 0.993424844 |
| *Calcr* | 0.163734239 | 8.866598e-01 | 0.3463840891 | 0.993424844 |
| *Calcr-1a* | 0.304276904 | 1.414687e+00 | 0.2342799538 | 0.993424844 |
| *Calcr-1b* | 0.104592785 | 1.483296e-01 | 0.7001368273 | 0.993424844 |
| *Cart* | -0.001563244 | 6.791128e-05 | 0.9934248435 | 0.993424844 |
| *Foxo1* | 0.230783492 | 2.208513e+00 | 0.1372509366 | 0.843112896 |
| *Gsk3a* | 0.030210108 | 3.269135e-02 | 0.8565186755 | 0.993424844 |
| *Gsk3b* | 0.017005154 | 1.052679e-02 | 0.9182803379 | 0.993424844 |
| *Hcrt* | -0.719969445 | 1.753488e+00 | 0.1854387804 | 0.993424844 |
| *Hdac5* | 0.002314929 | 2.115172e-04 | 0.9883962692 | 0.993424844 |
| *Hdc* | 0.079941321 | 1.944074e-01 | 0.6592733803 | 0.993424844 |
| *Hrh1* | 0.172025046 | 1.059480e+00 | 0.3033338010 | 0.993424844 |
| *Amylin* | -0.169933314 | 3.870248e-02 | 0.8440392643 | 0.993424844 |
| *Ins1* | 0.038293952 | 2.661694e-03 | 0.9588541100 | 0.993424844 |
| *Ins2* | -0.290379361 | 2.438179e-01 | 0.6214621582 | 0.993424844 |
| *Ir* | 0.094852246 | 3.744457e-01 | 0.5405909137 | 0.993424844 |
| *Irs1* | -0.021819112 | 1.727252e-02 | 0.8954391500 | 0.993424844 |
| *Irs2* | 0.018783896 | 1.453979e-02 | 0.9040228814 | 0.993424844 |
| *Jak2* | -0.086417130 | 2.798663e-01 | 0.5967888656 | 0.993424844 |
| *Lepr* | 0.044935720 | 8.161326e-02 | 0.7751229544 | 0.993424844 |
| *Lepr-b* | 0.329111554 | 2.815654e+00 | 0.0933488653 | 0.843112896 |
| *Mc4r* | -0.270225359 | 2.569106e+00 | 0.1089692949 | 0.843112896 |
| *Mch* | -0.326716927 | 2.687789e+00 | 0.1011200626 | 0.843112896 |
| *Npy* | 0.040899447 | 7.031454e-02 | 0.7908793526 | 0.993424844 |
| *Ptp1b* | 0.014111440 | 8.714392e-03 | 0.9256247897 | 0.993424844 |
| *Pde3b* | -0.022356893 | 2.081625e-02 | 0.8852806427 | 0.993424844 |
| *Pdk1* | 0.088999705 | 3.311744e-01 | 0.5649683799 | 0.993424844 |
| *Pias3* | 0.179247262 | 1.305069e+00 | 0.2532893683 | 0.993424844 |
| *Pik3ca* | -0.061125570 | 1.410578e-01 | 0.7072317153 | 0.993424844 |
| *Pik3r1* | 0.011113925 | 5.075841e-03 | 0.9432028201 | 0.993424844 |
| *Pomc* | 2.389232172 | 9.901933e+01 | 0.0000000000 | 0.000000000 |
| *Ramp1* | -0.091684944 | 3.430357e-01 | 0.5580821783 | 0.993424844 |
| *Ramp2* | 0.241702386 | 2.376945e+00 | 0.1231381337 | 0.843112896 |
| *Ramp3* | 0.053861906 | 1.079492e-01 | 0.7424911635 | 0.993424844 |
| *Socs3* | 0.230556672 | 4.758968e-01 | 0.4902868166 | 0.993424844 |
| *Stat3* | 0.079381723 | 2.633574e-01 | 0.6078228958 | 0.993424844 |
| *c-fos* | -0.646755304 | 1.466793e+01 | 0.0001282094 | 0.002756501 |
| *mTor* | 0.116592261 | 5.656598e-01 | 0.4519887604 | 0.993424844 |

### T2 Hindbrain: HEM vs NON (PM)

| **Gene** | **log_2_ FC** | **Likelihood-ratio test** | **p value** | **q value** |
| --- | --- | --- | --- | --- |
| *Adrbk1* | 0.12972773 | 0.9800868818 | 0.322177357 | 0.66684304 |
| *Agrp* | 0.38136103 | 2.0390365958 | 0.153306715 | 0.61266447 |
| *Akt1* | 0.18847472 | 2.0232996033 | 0.154902224 | 0.61266447 |
| *Akt2* | 0.27583397 | 4.1111982315 | 0.042600170 | 0.36636147 |
| *Arrb1* | 0.16910515 | 1.6087829218 | 0.204662970 | 0.66684304 |
| *Arrb2* | 0.04853181 | 0.1359245861 | 0.712366618 | 0.89312601 |
| *Calcr* | -0.15993440 | 1.2075444844 | 0.271818976 | 0.66684304 |
| *Calcr-1a* | -0.23274011 | 1.0532066926 | 0.304769711 | 0.66684304 |
| *Calcr-1b* | -0.10619688 | 0.2394574811 | 0.624598225 | 0.81387041 |
| *Cart* | -0.41919093 | 8.8310251697 | 0.002961520 | 0.08304545 |
| *Foxo1* | 0.38197749 | 8.3473140958 | 0.003862579 | 0.08304545 |
| *Gsk3a* | 0.03029040 | 0.0497219835 | 0.823547751 | 0.90148249 |
| *Gsk3b* | 0.10853511 | 0.6559791590 | 0.417983051 | 0.71893085 |
| *Hcrt* | 0.57192483 | 2.0055144080 | 0.156728120 | 0.61266447 |
| *Hdac5* | 0.15108109 | 1.3004826085 | 0.254125088 | 0.66684304 |
| *Hdc* | 0.01432212 | 0.0088042720 | 0.925243340 | 0.94727294 |
| *Hrh1* | 0.28555064 | 4.1653756159 | 0.041258264 | 0.36636147 |
| *Amylin* | 0.09099994 | 0.0141800435 | 0.905211985 | 0.94727294 |
| *Ins1* | -0.61785255 | 0.8807951007 | 0.347984000 | 0.66684304 |
| *Ins2* | -0.00646407 | 0.0001757848 | 0.989421648 | 0.98942165 |
| *Ir* | 0.17283080 | 1.7151822591 | 0.190314377 | 0.66684304 |
| *Irs1* | 0.25783221 | 3.3498440341 | 0.067211381 | 0.48168157 |
| *Irs2* | 0.22041459 | 2.6981748492 | 0.100463194 | 0.61266447 |
| *Jak2* | -0.05502259 | 0.4310691125 | 0.511464817 | 0.81291420 |
| *Lepr* | -0.08423307 | 0.3940321763 | 0.530187497 | 0.81291420 |
| *Lepr-b* | 0.14702010 | 0.8495365278 | 0.356683485 | 0.66684304 |
| *Mc4r* | -0.04942941 | 0.1219184349 | 0.726963035 | 0.89312601 |
| *Mch* | 0.08312228 | 0.2532779186 | 0.614776393 | 0.81387041 |
| *Npy* | 0.08043796 | 0.3711865196 | 0.542358223 | 0.81291420 |
| *Ptp1b* | 0.14509002 | 1.1991655766 | 0.273488516 | 0.66684304 |
| *Pde3b* | -0.02996129 | 0.0512846897 | 0.820842708 | 0.90148249 |
| *Pdk1* | 0.03297202 | 0.0629091122 | 0.801955683 | 0.90148249 |
| *Pias3* | 0.03311900 | 0.0620096983 | 0.803347271 | 0.90148249 |
| *Pik3ca* | -0.02771051 | 0.0414934718 | 0.838588361 | 0.90148249 |
| *Pik3r1* | -0.11578329 | 0.7758171219 | 0.378423473 | 0.67800872 |
| *Pomc* | 0.24099862 | 1.4443407416 | 0.229438204 | 0.66684304 |
| *Ramp1* | 0.07011179 | 0.2816761222 | 0.595604726 | 0.81387041 |
| *Ramp2* | 0.32319998 | 5.7698528163 | 0.016303400 | 0.23368207 |
| *Ramp3* | 0.08404023 | 0.3604715614 | 0.548244459 | 0.81291420 |
| *Socs3* | 0.26365944 | 0.8567007133 | 0.354663624 | 0.66684304 |
| *Stat3* | 0.06839724 | 0.2704597511 | 0.603023534 | 0.81387041 |
| *c-fos* | -0.20844801 | 2.2723421162 | 0.131700736 | 0.61266447 |
| *mTor* | 0.13286747 | 1.0055721535 | 0.315965956 | 0.66684304 |

### T2 Hindbrain: HOM vs NON (AM)

| **Gene** | **log_2_ FC** | **Likelihood-ratio test** | **p value** | **q value** |
| --- | --- | --- | --- | --- |
| *Adrbk1* | -1.836764e-02 | 1.373823e-02 | 9.066935e-01 | 1.0000000000 |
| *Agrp* | 4.821563e-05 | 0.000000e+00 | 1.000000e+00 | 1.0000000000 |
| *Akt1* | -6.705834e-02 | 1.796893e-01 | 6.716404e-01 | 0.9745829923 |
| *Akt2* | 9.868483e-03 | 3.924521e-03 | 9.500484e-01 | 1.0000000000 |
| *Arrb1* | -5.063911e-04 | 8.883949e-06 | 9.976218e-01 | 1.0000000000 |
| *Arrb2* | 5.599488e-02 | 1.701916e-01 | 6.799416e-01 | 0.9745829923 |
| *Calcr* | 1.630358e-01 | 8.892958e-01 | 3.456682e-01 | 0.9632721940 |
| *Calcr-1a* | 7.337160e-02 | 7.251545e-02 | 7.877089e-01 | 0.9962200681 |
| *Calcr-1b* | -5.854398e-01 | 3.543807e+00 | 5.976814e-02 | 0.5145991789 |
| *Cart* | -2.993938e-01 | 3.109438e+00 | 7.783981e-02 | 0.5145991789 |
| *Foxo1* | 2.773542e-01 | 3.234809e+00 | 7.208869e-02 | 0.5145991789 |
| *Gsk3a* | 1.429141e-01 | 8.188696e-01 | 3.655109e-01 | 0.9632721940 |
| *Gsk3b* | -4.355957e-02 | 7.838513e-02 | 7.794979e-01 | 0.9962200681 |
| *Hcrt* | -1.737271e-02 | 1.230103e-03 | 9.720217e-01 | 1.0000000000 |
| *Hdac5* | 2.892150e-03 | 3.251984e-04 | 9.856123e-01 | 1.0000000000 |
| *Hdc* | 4.270138e-02 | 5.414267e-02 | 8.160054e-01 | 1.0000000000 |
| *Hrh1* | 2.354875e-02 | 2.018018e-02 | 8.870350e-01 | 1.0000000000 |
| *Amylin* | -1.077662e+00 | 8.402270e-01 | 3.593319e-01 | 0.9632721940 |
| *Ins1* | -2.486823e-01 | 8.336367e-02 | 7.727898e-01 | 0.9962200681 |
| *Ins2* | 9.497183e-01 | 3.963526e+00 | 4.649622e-02 | 0.5145991789 |
| *Ir* | 4.724278e-02 | 9.589979e-02 | 7.568067e-01 | 0.9962200681 |
| *Irs1* | -7.193380e-02 | 1.862593e-01 | 6.660483e-01 | 0.9745829923 |
| *Irs2* | 1.166473e-01 | 5.750942e-01 | 4.482417e-01 | 0.9632721940 |
| *Jak2* | 8.832438e-02 | 3.825354e-01 | 5.362495e-01 | 0.9745829923 |
| *Lepr* | 1.964366e-01 | 1.597099e+00 | 2.063148e-01 | 0.7161160090 |
| *Lepr-b* | 3.260228e-01 | 2.751602e+00 | 9.715702e-02 | 0.5222190006 |
| *Mc4r* | 2.871690e-01 | 2.990159e+00 | 8.377196e-02 | 0.5145991789 |
| *Mch* | -2.771633e-01 | 1.907983e+00 | 1.671875e-01 | 0.7161160090 |
| *Npy* | 7.195653e-02 | 2.221240e-01 | 6.374263e-01 | 0.9745829923 |
| *Ptp1b* | 1.102595e-01 | 5.209534e-01 | 4.704353e-01 | 0.9632721940 |
| *Pde3b* | 2.218216e-01 | 2.146051e+00 | 1.429371e-01 | 0.6829216883 |
| *Pdk1* | 1.210958e-01 | 6.282168e-01 | 4.280102e-01 | 0.9632721940 |
| *Pias3* | 8.154960e-02 | 2.757972e-01 | 5.994692e-01 | 0.9745829923 |
| *Pik3ca* | 6.638662e-02 | 2.652114e-01 | 6.065623e-01 | 0.9745829923 |
| *Pik3r1* | 7.643305e-02 | 3.087955e-01 | 5.784203e-01 | 0.9745829923 |
| *Pomc* | 1.051691e+00 | 1.986621e+01 | 8.305564e-06 | 0.0003571392 |
| *Ramp1* | -1.013537e-01 | 4.412813e-01 | 5.065047e-01 | 0.9745829923 |
| *Ramp2* | 3.870067e-01 | 6.160238e+00 | 1.306536e-02 | 0.2809053021 |
| *Ramp3* | 1.253716e-01 | 5.802781e-01 | 4.462033e-01 | 0.9632721940 |
| *Socs3* | 5.648797e-02 | 2.463698e-02 | 8.752750e-01 | 1.0000000000 |
| *Stat3* | 2.024362e-01 | 1.766076e+00 | 1.838685e-01 | 0.7161160090 |
| *c-fos* | 1.253666e-01 | 5.981358e-01 | 4.392902e-01 | 0.9632721940 |
| *mTor* | 1.712218e-01 | 1.527419e+00 | 2.165002e-01 | 0.7161160090 |

### T2 Hindrain: HOM vs NON (PM)

| **Gene** | **log_2_ FC** | **Likelihood-ratio test** | **p value** | **q value** |
| --- | --- | --- | --- | --- |
| *Adrbk1* | 0.057176350 | 2.259378e-01 | 6.345524e-01 | 9.347453e-01 |
| *Agrp* | -0.355292569 | 1.761048e+00 | 1.844939e-01 | 9.347453e-01 |
| *Akt1* | -0.137661664 | 2.025076e+00 | 1.547212e-01 | 9.347453e-01 |
| *Akt2* | 0.008465967 | 4.523269e-03 | 9.463785e-01 | 9.877068e-01 |
| *Arrb1* | 0.082717006 | 4.042340e-01 | 5.249107e-01 | 9.347453e-01 |
| *Arrb2* | 0.157850245 | 1.601227e+00 | 2.057294e-01 | 9.347453e-01 |
| *Calcr* | 0.038433017 | 7.528425e-02 | 7.837928e-01 | 9.347453e-01 |
| *Calcr-1a* | 0.211511721 | 8.635633e-01 | 3.527434e-01 | 9.347453e-01 |
| *Calcr-1b* | 0.477474012 | 3.536691e+00 | 6.002511e-02 | 5.162159e-01 |
| *Cart* | -0.648254433 | 2.275804e+01 | 1.837339e-06 | 2.633519e-05 |
| *Foxo1* | 0.145020465 | 1.421260e+00 | 2.331961e-01 | 9.347453e-01 |
| *Gsk3a* | 0.055678730 | 1.734574e-01 | 6.770572e-01 | 9.347453e-01 |
| *Gsk3b* | -0.009762350 | 5.687524e-03 | 9.398840e-01 | 9.877068e-01 |
| *Hcrt* | 0.114051776 | 4.784323e-02 | 8.268596e-01 | 9.347453e-01 |
| *Hdac5* | -0.034272667 | 7.472893e-02 | 7.845719e-01 | 9.347453e-01 |
| *Hdc* | 0.072341854 | 2.296232e-01 | 6.318034e-01 | 9.347453e-01 |
| *Hrh1* | -0.077547678 | 3.512331e-01 | 5.534159e-01 | 9.347453e-01 |
| *Amylin* | 0.117727741 | 3.683949e-02 | 8.477922e-01 | 9.347453e-01 |
| *Ins1* | 0.503703162 | 6.442742e-01 | 4.221673e-01 | 9.347453e-01 |
| *Ins2* | 0.500321807 | 9.673850e-01 | 3.253332e-01 | 9.347453e-01 |
| *Ir* | 0.036011235 | 8.870818e-02 | 7.658259e-01 | 9.347453e-01 |
| *Irs1* | -0.087898200 | 4.327789e-01 | 5.106285e-01 | 9.347453e-01 |
| *Irs2* | -0.029261253 | 5.817134e-02 | 8.094099e-01 | 9.347453e-01 |
| *Jak2* | 0.047871346 | 1.349704e-01 | 7.133332e-01 | 9.347453e-01 |
| *Lepr* | 0.043861953 | 1.247604e-01 | 7.239277e-01 | 9.347453e-01 |
| *Lepr-b* | -0.119844906 | 5.533624e-01 | 4.569471e-01 | 9.347453e-01 |
| *Mc4r* | -0.123535328 | 8.368798e-01 | 3.602907e-01 | 9.347453e-01 |
| *Mch* | 0.105397989 | 3.800546e-01 | 5.375740e-01 | 9.347453e-01 |
| *Npy* | 0.109431426 | 8.005045e-01 | 3.709426e-01 | 9.347453e-01 |
| *Ptp1b* | 0.038068104 | 9.863833e-02 | 7.534699e-01 | 9.347453e-01 |
| *Pde3b* | 0.052740946 | 1.816870e-01 | 6.699275e-01 | 9.347453e-01 |
| *Pdk1* | 0.103807659 | 7.249469e-01 | 3.945260e-01 | 9.347453e-01 |
| *Pias3* | 0.139815572 | 1.290059e+00 | 2.560371e-01 | 9.347453e-01 |
| *Pik3ca* | 0.039973789 | 8.922280e-02 | 7.651675e-01 | 9.347453e-01 |
| *Pik3r1* | 0.001876836 | 2.374025e-04 | 9.877068e-01 | 9.877068e-01 |
| *Pomc* | 1.868369778 | 1.289077e+02 | 0.000000e+00 | 0.000000e+00 |
| *Ramp1* | -0.048938531 | 1.594795e-01 | 6.896362e-01 | 9.347453e-01 |
| *Ramp2* | 0.389452903 | 1.001767e+01 | 1.550451e-03 | 1.666735e-02 |
| *Ramp3* | 0.059808992 | 2.106608e-01 | 6.462500e-01 | 9.347453e-01 |
| *Socs3* | 0.241958053 | 5.414084e-01 | 4.618496e-01 | 9.347453e-01 |
| *Stat3* | 0.005435261 | 1.919403e-03 | 9.650551e-01 | 9.877068e-01 |
| *c-fos* | -0.927698029 | 5.247706e+01 | 4.353184e-13 | 9.359347e-12 |
| *mTor* | 0.034243464 | 7.686119e-02 | 7.815970e-01 | 9.347453e-01 |

### T2 Midbrain: HEM vs NON (AM)

| **Gene** | **log_2_ FC** | **Likelihood-ratio test** | **p value** | **q value** |
| --- | --- | --- | --- | --- |
| *Adrbk1* | 0.087434603 | 3.683359e-01 | 5.439127e-01 | 8.238221e-01 |
| *Agrp* | -0.107678924 | 2.645089e-01 | 6.070393e-01 | 8.238221e-01 |
| *Akt1* | -0.019026024 | 1.405143e-02 | 9.056408e-01 | 9.865301e-01 |
| *Akt2* | 0.103854822 | 4.205939e-01 | 5.166409e-01 | 8.238221e-01 |
| *Arrb1* | 0.204468171 | 2.503118e+00 | 1.136212e-01 | 4.441555e-01 |
| *Arrb2* | 0.272443441 | 3.043545e+00 | 8.105872e-02 | 4.356906e-01 |
| *Calcr* | 0.126056306 | 6.023229e-01 | 4.376931e-01 | 8.182958e-01 |
| *Calcr-1a* | -0.009308714 | 1.772442e-03 | 9.664187e-01 | 9.865301e-01 |
| *Calcr-1b* | 0.217888899 | 1.203004e+00 | 2.727220e-01 | 6.569458e-01 |
| *Cart* | -0.258991767 | 2.676820e+00 | 1.018189e-01 | 4.378212e-01 |
| *Foxo1* | 0.224561672 | 2.060532e+00 | 1.511574e-01 | 5.416474e-01 |
| *Gsk3a* | 0.163820925 | 1.611727e+00 | 2.042492e-01 | 6.509946e-01 |
| *Gsk3b* | 0.072163355 | 2.537330e-01 | 6.144587e-01 | 8.238221e-01 |
| *Hcrt* | -0.276671279 | 2.838269e+00 | 9.204335e-02 | 4.378212e-01 |
| *Hdac5* | 0.007158937 | 2.050963e-03 | 9.638781e-01 | 9.865301e-01 |
| *Hdc* | -0.215766210 | 1.452206e+00 | 2.281742e-01 | 6.509946e-01 |
| *Hrh1* | -0.126570170 | 6.241818e-01 | 4.294975e-01 | 8.182958e-01 |
| *Amylin* | 2.603251694 | 3.398061e+01 | 5.566394e-09 | 2.393550e-07 |
| *Ins1* | -0.285291150 | 4.313373e-01 | 5.113335e-01 | 8.238221e-01 |
| *Ins2* | 0.008627361 | 4.940956e-04 | 9.822659e-01 | 9.865301e-01 |
| *Ir* | 0.183913719 | 1.367566e+00 | 2.422306e-01 | 6.509946e-01 |
| *Irs1* | -0.214326939 | 1.518392e+00 | 2.178630e-01 | 6.509946e-01 |
| *Irs2* | 0.002653744 | 2.850289e-04 | 9.865301e-01 | 9.865301e-01 |
| *Jak2* | 0.051000463 | 9.782276e-02 | 7.544582e-01 | 9.269058e-01 |
| *Lepr* | -0.013266419 | 6.986038e-03 | 9.333884e-01 | 9.865301e-01 |
| *Lepr-b* | -0.109079692 | 3.452737e-01 | 5.568008e-01 | 8.238221e-01 |
| *Mc4r* | 0.079302941 | 2.425000e-01 | 6.224063e-01 | 8.238221e-01 |
| *Mch* | -0.161243359 | 9.232711e-01 | 3.366175e-01 | 6.892645e-01 |
| *Npy* | 0.916472412 | 2.986973e+01 | 4.620697e-08 | 9.934499e-07 |
| *Ptp1b* | 0.039129376 | 6.319623e-02 | 8.015137e-01 | 9.573636e-01 |
| *Pde3b* | 0.071241715 | 2.041469e-01 | 6.513942e-01 | 8.238221e-01 |
| *Pdk1* | 0.414425682 | 6.979479e+00 | 8.244962e-03 | 8.863334e-02 |
| *Pias3* | 0.283574230 | 3.146564e+00 | 7.608702e-02 | 4.356906e-01 |
| *Pik3ca* | 0.071435411 | 2.205680e-01 | 6.386075e-01 | 8.238221e-01 |
| *Pik3r1* | 0.112206598 | 9.404013e-01 | 3.321747e-01 | 6.892645e-01 |
| *Pomc* | -0.003746228 | 4.752796e-04 | 9.826068e-01 | 9.865301e-01 |
| *Ramp1* | 0.121558945 | 1.191632e+00 | 2.750006e-01 | 6.569458e-01 |
| *Ramp2* | 0.347323405 | 4.830336e+00 | 2.796318e-02 | 2.404833e-01 |
| *Ramp3* | -0.089029861 | 3.211977e-01 | 5.708888e-01 | 8.238221e-01 |
| *Socs3* | 0.142820217 | 2.686394e-01 | 6.042460e-01 | 8.238221e-01 |
| *Stat3* | 0.281108239 | 3.226726e+00 | 7.244540e-02 | 4.356906e-01 |
| *c-fos* | -0.782631303 | 2.143280e+01 | 3.664477e-06 | 5.252417e-05 |
| *mTor* | 0.158308726 | 1.029688e+00 | 3.102321e-01 | 6.892645e-01 |

### T2 Midbrain: HEM vs NON (PM)

| **Gene** | **log_2_ FC** | **Likelihood-ratio test** | **p value** | **q value** |
| --- | --- | --- | --- | --- |
| *Adrbk1* | 0.09609118 | 0.562912680 | 4.530891e-01 | 8.133348e-01 |
| *Agrp* | -0.03217934 | 0.034048250 | 8.536042e-01 | 9.296496e-01 |
| *Akt1* | -0.02693784 | 0.043012278 | 8.357022e-01 | 9.296496e-01 |
| *Akt2* | 0.02143162 | 0.027223109 | 8.689485e-01 | 9.296496e-01 |
| *Arrb1* | 0.16155237 | 1.451860580 | 2.282296e-01 | 7.549134e-01 |
| *Arrb2* | 0.02626834 | 0.042823810 | 8.360574e-01 | 9.296496e-01 |
| *Calcr* | 0.06096707 | 0.213601189 | 6.439594e-01 | 9.296496e-01 |
| *Calcr-1a* | 0.05928981 | 0.106035827 | 7.447032e-01 | 9.296496e-01 |
| *Calcr-1b* | 0.18749402 | 1.338210371 | 2.473498e-01 | 7.597173e-01 |
| *Cart* | 0.22437863 | 2.795434116 | 9.453316e-02 | 4.982121e-01 |
| *Foxo1* | 0.08713171 | 0.472620986 | 4.917839e-01 | 8.133348e-01 |
| *Gsk3a* | 0.09674817 | 0.506921362 | 4.764746e-01 | 8.133348e-01 |
| *Gsk3b* | 0.03526731 | 0.069485377 | 7.920875e-01 | 9.296496e-01 |
| *Hcrt* | -0.13742134 | 1.072230190 | 3.004422e-01 | 7.912255e-01 |
| *Hdac5* | 0.02581045 | 0.040273152 | 8.409475e-01 | 9.296496e-01 |
| *Hdc* | -0.13947337 | 0.872751170 | 3.501948e-01 | 8.133348e-01 |
| *Hrh1* | -0.01148376 | 0.007794372 | 9.296496e-01 | 9.296496e-01 |
| *Amylin* | -0.23456627 | 0.531105732 | 4.661424e-01 | 8.133348e-01 |
| *Ins1* | -0.74229422 | 3.396608526 | 6.533061e-02 | 4.836796e-01 |
| *Ins2* | 0.31591420 | 0.567516214 | 4.512476e-01 | 8.133348e-01 |
| *Ir* | 0.01148655 | 0.008055036 | 9.284860e-01 | 9.296496e-01 |
| *Irs1* | -0.14616680 | 1.059607501 | 3.033047e-01 | 7.912255e-01 |
| *Irs2* | -0.01665472 | 0.016837467 | 8.967569e-01 | 9.296496e-01 |
| *Jak2* | 0.10224295 | 0.593463855 | 4.410828e-01 | 8.133348e-01 |
| *Lepr* | -0.23548847 | 3.294638675 | 6.950640e-02 | 4.836796e-01 |
| *Lepr-b* | -0.15458856 | 1.018773605 | 3.128101e-01 | 7.912255e-01 |
| *Mc4r* | -0.01750729 | 0.017272726 | 8.954385e-01 | 9.296496e-01 |
| *Mch* | -0.16749745 | 1.682937276 | 1.945346e-01 | 6.970824e-01 |
| *Npy* | 0.77430542 | 32.001508703 | 1.540529e-08 | 6.624274e-07 |
| *Ptp1b* | 0.04001141 | 0.098457724 | 7.536883e-01 | 9.296496e-01 |
| *Pde3b* | 0.02313564 | 0.032563765 | 8.567959e-01 | 9.296496e-01 |
| *Pdk1* | 0.20745963 | 2.638881106 | 1.042770e-01 | 4.982121e-01 |
| *Pias3* | 0.10242348 | 0.613519900 | 4.334656e-01 | 8.133348e-01 |
| *Pik3ca* | 0.02736990 | 0.042321371 | 8.370084e-01 | 9.296496e-01 |
| *Pik3r1* | 0.04541534 | 0.128096349 | 7.204140e-01 | 9.296496e-01 |
| *Pomc* | 0.55850461 | 13.345853099 | 2.589947e-04 | 3.712258e-03 |
| *Ramp1* | 0.02125749 | 0.027264363 | 8.688501e-01 | 9.296496e-01 |
| *Ramp2* | 0.17201700 | 1.781138536 | 1.820096e-01 | 6.970824e-01 |
| *Ramp3* | -0.18281135 | 2.062088187 | 1.510031e-01 | 6.493134e-01 |
| *Socs3* | 0.38637724 | 3.090749233 | 7.873855e-02 | 4.836796e-01 |
| *Stat3* | 0.24511927 | 3.730150326 | 5.343858e-02 | 4.836796e-01 |
| *c-fos* | 0.71477797 | 21.776752722 | 3.062889e-06 | 6.585212e-05 |
| *mTor* | 0.11425306 | 0.811455236 | 3.676904e-01 | 8.133348e-01 |

### T2 Midbrain: HOM vs NON (AM)

| **Gene** | **log_2_ FC** | **Likelihood-ratio test** | **p value** | **q value** |
| --- | --- | --- | --- | --- |
| *Adrbk1* | -0.087188652 | 0.305640077 | 0.580368014 | 0.96780761 |
| *Agrp* | -0.008215116 | 0.001628778 | 0.967807615 | 0.96780761 |
| *Akt1* | -0.154774651 | 0.927183482 | 0.335595866 | 0.96780761 |
| *Akt2* | -0.159812488 | 1.023541417 | 0.311680446 | 0.96780761 |
| *Arrb1* | -0.034888151 | 0.044861433 | 0.832259029 | 0.96780761 |
| *Arrb2* | 0.007770349 | 0.002508039 | 0.960058380 | 0.96780761 |
| *Calcr* | 0.114748415 | 0.518164315 | 0.471625785 | 0.96780761 |
| *Calcr-1a* | -0.092954211 | 0.177539225 | 0.673496576 | 0.96780761 |
| *Calcr-1b* | 0.269817388 | 1.890964729 | 0.169093150 | 0.96780761 |
| *Cart* | 0.225296744 | 3.182309207 | 0.074439461 | 0.80022420 |
| *Foxo1* | -0.023372422 | 0.022822422 | 0.879919774 | 0.96780761 |
| *Gsk3a* | 0.021375025 | 0.016380170 | 0.898160812 | 0.96780761 |
| *Gsk3b* | -0.042129291 | 0.064843209 | 0.798998615 | 0.96780761 |
| *Hcrt* | -0.265272766 | 2.742722633 | 0.097698181 | 0.84020436 |
| *Hdac5* | -0.040763703 | 0.065792554 | 0.797564323 | 0.96780761 |
| *Hdc* | -0.246815734 | 1.932897174 | 0.164441828 | 0.96780761 |
| *Hrh1* | -0.067041562 | 0.181732451 | 0.669888607 | 0.96780761 |
| *Amylin* | 1.729682086 | 10.313519171 | 0.001320592 | 0.05678546 |
| *Ins1* | -0.418333860 | 0.869300631 | 0.351148996 | 0.96780761 |
| *Ins2* | 0.140673191 | 0.148641195 | 0.699837277 | 0.96780761 |
| *Ir* | 0.006637555 | 0.001794362 | 0.966211785 | 0.96780761 |
| *Irs1* | -0.080877035 | 0.222676647 | 0.637007972 | 0.96780761 |
| *Irs2* | 0.052521286 | 0.115760480 | 0.733678871 | 0.96780761 |
| *Jak2* | -0.025145898 | 0.023931823 | 0.877058510 | 0.96780761 |
| *Lepr* | -0.051648617 | 0.109723208 | 0.740459480 | 0.96780761 |
| *Lepr-b* | 0.176125408 | 0.944752698 | 0.331058559 | 0.96780761 |
| *Mc4r* | -0.021341476 | 0.018101874 | 0.892973048 | 0.96780761 |
| *Mch* | 0.047981308 | 0.082815331 | 0.773517804 | 0.96780761 |
| *Npy* | 0.015403383 | 0.008880075 | 0.924923157 | 0.96780761 |
| *Ptp1b* | -0.087107230 | 0.315768248 | 0.574161941 | 0.96780761 |
| *Pde3b* | -0.152089513 | 0.961553657 | 0.326795745 | 0.96780761 |
| *Pdk1* | -0.162381677 | 1.092740449 | 0.295864811 | 0.96780761 |
| *Pias3* | 0.052821040 | 0.113077635 | 0.736667134 | 0.96780761 |
| *Pik3ca* | -0.058998055 | 0.128244204 | 0.720259499 | 0.96780761 |
| *Pik3r1* | -0.054599705 | 0.121888516 | 0.726995199 | 0.96780761 |
| *Pomc* | 0.501745183 | 8.863417381 | 0.002909424 | 0.06255261 |
| *Ramp1* | -0.072655208 | 0.209104849 | 0.647469921 | 0.96780761 |
| *Ramp2* | 0.116454504 | 0.561551495 | 0.453635796 | 0.96780761 |
| *Ramp3* | 0.082339762 | 0.284386235 | 0.593840629 | 0.96780761 |
| *Socs3* | 0.090511794 | 0.105371673 | 0.745476234 | 0.96780761 |
| *Stat3* | 0.098287886 | 0.410814560 | 0.521556356 | 0.96780761 |
| *c-fos* | 0.385162884 | 5.563254989 | 0.018341282 | 0.26289171 |
| *mTor* | -0.017354035 | 0.012418655 | 0.911268221 | 0.96780761 |

### T2 Midbrain: HOM vs NON (PM)

| **Gene** | **log_2_ FC** | **Likelihood-ratio test** | **p value** | **q value** |
| --- | --- | --- | --- | --- |
| *Adrbk1* | 0.018944157 | 0.021913876 | 8.823164e-01 | 9.764926e-01 |
| *Agrp* | 0.067153568 | 0.148526371 | 6.999476e-01 | 9.764926e-01 |
| *Akt1* | -0.170625487 | 1.908434636 | 1.671372e-01 | 5.528386e-01 |
| *Akt2* | 0.022369440 | 0.029012540 | 8.647501e-01 | 9.764926e-01 |
| *Arrb1* | 0.074304144 | 0.307539141 | 5.791942e-01 | 9.764926e-01 |
| *Arrb2* | 0.005709378 | 0.001949369 | 9.647835e-01 | 9.764926e-01 |
| *Calcr* | -0.042434814 | 0.099137299 | 7.528674e-01 | 9.764926e-01 |
| *Calcr-1a* | -0.063222564 | 0.113365049 | 7.363451e-01 | 9.764926e-01 |
| *Calcr-1b* | -0.101360845 | 0.401836364 | 5.261424e-01 | 9.764926e-01 |
| *Cart* | -0.139717887 | 1.170005726 | 2.794001e-01 | 7.736148e-01 |
| *Foxo1* | -0.105936133 | 0.685465112 | 4.077112e-01 | 9.739767e-01 |
| *Gsk3a* | 0.032328132 | 0.056001502 | 8.129311e-01 | 9.764926e-01 |
| *Gsk3b* | -0.054177960 | 0.161925024 | 6.873903e-01 | 9.764926e-01 |
| *Hcrt* | -0.259575754 | 3.763642297 | 5.237837e-02 | 2.815337e-01 |
| *Hdac5* | -0.128390080 | 1.502003927 | 2.203633e-01 | 6.768301e-01 |
| *Hdc* | -0.332346344 | 5.090964861 | 2.405081e-02 | 1.723641e-01 |
| *Hrh1* | -0.044868161 | 0.117188751 | 7.321037e-01 | 9.764926e-01 |
| *Amylin* | 0.984645728 | 16.457931666 | 4.974163e-05 | 7.129634e-04 |
| *Ins1* | -0.517045497 | 2.380355421 | 1.228696e-01 | 4.803084e-01 |
| *Ins2* | 0.058043951 | 0.028701047 | 8.654711e-01 | 9.764926e-01 |
| *Ir* | -0.016340385 | 0.016172173 | 8.988060e-01 | 9.764926e-01 |
| *Irs1* | -0.263006991 | 3.439098690 | 6.367042e-02 | 3.042031e-01 |
| *Irs2* | -0.053027984 | 0.170682817 | 6.795057e-01 | 9.764926e-01 |
| *Jak2* | 0.097793823 | 0.543103778 | 4.611493e-01 | 9.764926e-01 |
| *Lepr* | 0.022109251 | 0.028946546 | 8.649025e-01 | 9.764926e-01 |
| *Lepr-b* | -0.269994302 | 3.258702882 | 7.104530e-02 | 3.054948e-01 |
| *Mc4r* | -0.120786133 | 0.842398043 | 3.587118e-01 | 9.073299e-01 |
| *Mch* | -0.135376666 | 2.011709304 | 1.560894e-01 | 5.528386e-01 |
| *Npy* | 0.727040326 | 29.726042993 | 4.976180e-08 | 1.069879e-06 |
| *Ptp1b* | -0.012406897 | 0.009354918 | 9.229481e-01 | 9.764926e-01 |
| *Pde3b* | -0.034774183 | 0.071638628 | 7.889657e-01 | 9.764926e-01 |
| *Pdk1* | -0.135984820 | 1.129618794 | 2.878567e-01 | 7.736148e-01 |
| *Pias3* | -0.007744116 | 0.003503049 | 9.528035e-01 | 9.764926e-01 |
| *Pik3ca* | 0.037813989 | 0.079716123 | 7.776825e-01 | 9.764926e-01 |
| *Pik3r1* | -0.092335665 | 0.524427569 | 4.689591e-01 | 9.764926e-01 |
| *Pomc* | 0.482955087 | 10.606907821 | 1.126659e-03 | 1.211159e-02 |
| *Ramp1* | -0.066003528 | 0.261958031 | 6.087781e-01 | 9.764926e-01 |
| *Ramp2* | 0.003894613 | 0.000868268 | 9.764926e-01 | 9.764926e-01 |
| *Ramp3* | -0.042850216 | 0.111538720 | 7.383991e-01 | 9.764926e-01 |
| *Socs3* | 1.780148531 | 76.221907751 | 0.000000e+00 | 0.000000e+00 |
| *Stat3* | 0.351815560 | 7.300031126 | 6.895342e-03 | 5.929994e-02 |
| *c-fos* | -0.295633074 | 4.376476023 | 3.643826e-02 | 2.238350e-01 |
| *mTor* | -0.004363903 | 0.001203034 | 9.723311e-01 | 9.764926e-01 |

### T2 Left Cortex: HEM vs NON (AM)

| **Gene** | **log_2_ FC** | **Likelihood-ratio test** | **p value** | **q value** |
| --- | --- | --- | --- | --- |
| *Adrbk1* | -0.098148037 | 0.370449525 | 5.427593e-01 | 9.275538e-01 |
| *Agrp* | 0.771513457 | 3.225155749 | 7.251490e-02 | 3.464601e-01 |
| *Akt1* | -0.045903527 | 0.080547637 | 7.765566e-01 | 9.275538e-01 |
| *Akt2* | -0.271435078 | 2.724575942 | 9.881436e-02 | 4.249018e-01 |
| *Arrb1* | 0.049865670 | 0.181969278 | 6.696863e-01 | 9.275538e-01 |
| *Arrb2* | 0.169848197 | 1.083055869 | 2.980149e-01 | 7.119246e-01 |
| *Calcr* | 2.857817762 | 15.155989025 | 9.898384e-05 | 1.418768e-03 |
| *Calcr-1a* | -8.537095678 | 0.141701388 | 7.065954e-01 | 9.275538e-01 |
| *Calcr-1b* | 0.970796041 | 0.705810286 | 4.008382e-01 | 9.071602e-01 |
| *Cart* | -1.582284063 | 83.928531861 | 0.000000e+00 | 0.000000e+00 |
| *Foxo1* | -0.020095123 | 0.014766864 | 9.032800e-01 | 9.484841e-01 |
| *Gsk3a* | -0.107640042 | 0.398850319 | 5.276836e-01 | 9.275538e-01 |
| *Gsk3b* | -0.102861185 | 0.383324366 | 5.358294e-01 | 9.275538e-01 |
| *Hcrt* | -0.652901774 | 1.485233928 | 2.229574e-01 | 5.991981e-01 |
| *Hdac5* | -0.220156901 | 1.855313641 | 1.731666e-01 | 5.727820e-01 |
| *Hdc* | 0.160224788 | 0.341438382 | 5.590001e-01 | 9.275538e-01 |
| *Hrh1* | 0.020028050 | 0.014434732 | 9.043686e-01 | 9.484841e-01 |
| *Amylin* | 0.486134358 | 0.124376326 | 7.243356e-01 | 9.275538e-01 |
| *Ins1* | 1.369176723 | 0.059586779 | 8.071502e-01 | 9.347013e-01 |
| *Ins2* | -1.935946988 | 1.905778676 | 1.674329e-01 | 5.727820e-01 |
| *Ir* | 0.005807080 | 0.001258781 | 9.716976e-01 | 9.716976e-01 |
| *Irs1* | -0.216813796 | 1.489212358 | 2.223387e-01 | 5.991981e-01 |
| *Irs2* | -0.211493868 | 1.687140787 | 1.939783e-01 | 5.957905e-01 |
| *Jak2* | -0.301452093 | 3.301163477 | 6.923083e-02 | 3.464601e-01 |
| *Lepr* | -0.055913208 | 0.111302074 | 7.386666e-01 | 9.275538e-01 |
| *Lepr-b* | -0.348330524 | 3.301259550 | 6.922678e-02 | 3.464601e-01 |
| *Mc4r* | 0.130235749 | 0.565141710 | 4.521960e-01 | 9.275538e-01 |
| *Mch* | 0.120057820 | 0.288728567 | 5.910364e-01 | 9.275538e-01 |
| *Npy* | 0.409781643 | 7.925518961 | 4.874236e-03 | 5.239804e-02 |
| *Ptp1b* | -0.316737889 | 3.836729654 | 5.014123e-02 | 3.464601e-01 |
| *Pde3b* | -0.063138240 | 0.149286304 | 6.992183e-01 | 9.275538e-01 |
| *Pdk1* | 0.380473270 | 5.442347807 | 1.965423e-02 | 1.690264e-01 |
| *Pias3* | 0.053403013 | 0.105673294 | 7.451248e-01 | 9.275538e-01 |
| *Pik3ca* | -0.176200036 | 1.117105155 | 2.905426e-01 | 7.119246e-01 |
| *Pik3r1* | -0.035464393 | 0.048318728 | 8.260151e-01 | 9.347013e-01 |
| *Pomc* | -0.135613167 | 0.282054576 | 5.953577e-01 | 9.275538e-01 |
| *Ramp1* | -0.067909829 | 0.176017478 | 6.748183e-01 | 9.275538e-01 |
| *Ramp2* | 0.006216271 | 0.001402096 | 9.701305e-01 | 9.716976e-01 |
| *Ramp3* | 0.048297712 | 0.082488776 | 7.739526e-01 | 9.275538e-01 |
| *Socs3* | -0.364871132 | 2.075854506 | 1.496461e-01 | 5.727820e-01 |
| *Stat3* | -0.027862457 | 0.029524236 | 8.635742e-01 | 9.484841e-01 |
| *c-fos* | -1.168912738 | 47.279383872 | 6.155632e-12 | 1.323461e-10 |
| *mTor* | -0.077549923 | 0.230655212 | 6.310384e-01 | 9.275538e-01 |

### T2 Left Cortex: HEM vs NON (PM)

| **Gene** | **log_2_ FC** | **Likelihood-ratio test** | **p value** | **q value** |
| --- | --- | --- | --- | --- |
| *Adrbk1* | -0.002433383 | 3.353428e-04 | 9.853897e-01 | 9.853897e-01 |
| *Agrp* | -0.018829798 | 2.412853e-03 | 9.608230e-01 | 9.836998e-01 |
| *Akt1* | 0.059847676 | 2.268982e-01 | 6.338334e-01 | 9.257540e-01 |
| *Akt2* | -0.248425540 | 3.545540e+00 | 5.970573e-02 | 1.974882e-01 |
| *Arrb1* | 0.097900186 | 5.462953e-01 | 4.598354e-01 | 8.987691e-01 |
| *Arrb2* | 0.018451324 | 2.043816e-02 | 8.863201e-01 | 9.836998e-01 |
| *Calcr* | 4.264237245 | 1.748338e+02 | 0.000000e+00 | 0.000000e+00 |
| *Calcr-1a* | 26.926748717 | 4.342227e+00 | 3.717826e-02 | 1.598665e-01 |
| *Calcr-1b* | 1.888568421 | 1.151010e+01 | 6.921913e-04 | 4.960704e-03 |
| *Cart* | -1.367194965 | 8.930751e+01 | 0.000000e+00 | 0.000000e+00 |
| *Foxo1* | 0.143011373 | 1.160904e+00 | 2.812780e-01 | 6.719420e-01 |
| *Gsk3a* | 0.028090521 | 4.030561e-02 | 8.408842e-01 | 9.836998e-01 |
| *Gsk3b* | -0.026556662 | 3.823378e-02 | 8.449745e-01 | 9.836998e-01 |
| *Hcrt* | 0.889260871 | 5.268280e+00 | 2.171746e-02 | 1.037612e-01 |
| *Hdac5* | -0.037788343 | 8.483177e-02 | 7.708533e-01 | 9.836998e-01 |
| *Hdc* | 0.035544816 | 2.199816e-02 | 8.820920e-01 | 9.836998e-01 |
| *Hrh1* | 0.273170765 | 4.115491e+00 | 4.249220e-02 | 1.661059e-01 |
| *Amylin* | -1.095953954 | 1.491379e+00 | 2.220027e-01 | 5.966322e-01 |
| *Ins1* | 1.618425042 | 1.678382e+00 | 1.951396e-01 | 5.594002e-01 |
| *Ins2* | 23.795328761 | 1.234443e+00 | 2.665448e-01 | 6.719420e-01 |
| *Ir* | 0.071959837 | 3.047255e-01 | 5.809350e-01 | 9.257540e-01 |
| *Irs1* | -0.052685977 | 1.332655e-01 | 7.150700e-01 | 9.836998e-01 |
| *Irs2* | -0.113188957 | 7.263818e-01 | 3.940585e-01 | 8.472258e-01 |
| *Jak2* | -0.076485514 | 3.179873e-01 | 5.728197e-01 | 9.257540e-01 |
| *Lepr* | 0.016330481 | 1.512987e-02 | 9.021043e-01 | 9.836998e-01 |
| *Lepr-b* | -0.298567276 | 3.548393e+00 | 5.960313e-02 | 1.974882e-01 |
| *Mc4r* | 0.866383052 | 3.722579e+01 | 1.052133e-09 | 1.131043e-08 |
| *Mch* | 0.342752915 | 3.394080e+00 | 6.543084e-02 | 2.009661e-01 |
| *Npy* | 0.387751701 | 8.005670e+00 | 4.663110e-03 | 2.864482e-02 |
| *Ptp1b* | -0.124985633 | 9.210035e-01 | 3.372116e-01 | 7.631632e-01 |
| *Pde3b* | 0.011923047 | 8.381651e-03 | 9.270545e-01 | 9.836998e-01 |
| *Pdk1* | 0.340612207 | 7.559460e+00 | 5.969589e-03 | 3.208654e-02 |
| *Pias3* | 0.067065322 | 2.597767e-01 | 6.102736e-01 | 9.257540e-01 |
| *Pik3ca* | -0.062712224 | 2.111406e-01 | 6.458749e-01 | 9.257540e-01 |
| *Pik3r1* | 0.063985405 | 3.897861e-01 | 5.324119e-01 | 9.257540e-01 |
| *Pomc* | 0.799226222 | 1.379126e+01 | 2.042841e-04 | 1.756843e-03 |
| *Ramp1* | 0.068220947 | 2.785872e-01 | 5.976287e-01 | 9.257540e-01 |
| *Ramp2* | 0.064744446 | 2.380669e-01 | 6.256058e-01 | 9.257540e-01 |
| *Ramp3* | 0.018274346 | 1.840514e-02 | 8.920857e-01 | 9.836998e-01 |
| *Socs3* | 0.028466572 | 1.952367e-02 | 8.888756e-01 | 9.836998e-01 |
| *Stat3* | 0.100782349 | 6.032798e-01 | 4.373294e-01 | 8.954840e-01 |
| *c-fos* | 1.179346041 | 4.264448e+01 | 6.564949e-11 | 9.409760e-10 |
| *mTor* | 0.009073465 | 4.914666e-03 | 9.441103e-01 | 9.836998e-01 |

### T2 Left Cortex: HOM vs NON (AM)

| **Gene** | **log_2_ FC** | **Likelihood-ratio test** | **p value** | **q value** |
| --- | --- | --- | --- | --- |
| *Adrbk1* | 0.022393212 | 0.0190660474 | 0.890177304 | 0.9860215 |
| *Agrp* | 0.472093813 | 1.1218107466 | 0.289528887 | 0.9860215 |
| *Akt1* | 0.011001272 | 0.0047737265 | 0.944916262 | 0.9860215 |
| *Akt2* | -0.002732185 | 0.0003069608 | 0.986021541 | 0.9860215 |
| *Arrb1* | 0.123910004 | 0.5469615695 | 0.459561860 | 0.9860215 |
| *Arrb2* | 0.115608708 | 0.5382116968 | 0.463174804 | 0.9860215 |
| *Calcr* | 2.354094542 | 7.9779156662 | 0.004735143 | 0.2036111 |
| *Calcr-1a* | 30.415371850 | 1.0560223221 | 0.304124157 | 0.9860215 |
| *Calcr-1b* | 1.772926560 | 4.0331401218 | 0.044614827 | 0.9592188 |
| *Cart* | 0.158427254 | 0.9659001901 | 0.325704792 | 0.9860215 |
| *Foxo1* | 0.060364341 | 0.1471998386 | 0.701225785 | 0.9860215 |
| *Gsk3a* | -0.010829746 | 0.0039901816 | 0.949632819 | 0.9860215 |
| *Gsk3b* | 0.028824868 | 0.0300255837 | 0.862432193 | 0.9860215 |
| *Hcrt* | 0.218546062 | 0.2585317053 | 0.611130692 | 0.9860215 |
| *Hdac5* | -0.029575548 | 0.0430431454 | 0.835644053 | 0.9860215 |
| *Hdc* | 0.071591147 | 0.0700820189 | 0.791217398 | 0.9860215 |
| *Hrh1* | -0.143730072 | 0.8042279554 | 0.369832279 | 0.9860215 |
| *Amylin* | -2.071765825 | 0.5575133954 | 0.455263872 | 0.9860215 |
| *Ins1* | 3.712998293 | 2.7158326943 | 0.099357111 | 0.9860215 |
| *Ins2* | -2.573071450 | 2.6493208405 | 0.103594147 | 0.9860215 |
| *Ir* | 0.085555364 | 0.2971647881 | 0.585665328 | 0.9860215 |
| *Irs1* | 0.033664430 | 0.0389460693 | 0.843555544 | 0.9860215 |
| *Irs2* | -0.102462701 | 0.4265300459 | 0.513696532 | 0.9860215 |
| *Jak2* | 0.004443520 | 0.0006468287 | 0.979709728 | 0.9860215 |
| *Lepr* | -0.174390954 | 1.1853585850 | 0.276267788 | 0.9860215 |
| *Lepr-b* | -0.067346050 | 0.1362808689 | 0.712006689 | 0.9860215 |
| *Mc4r* | 0.190400809 | 1.3155972832 | 0.251383732 | 0.9860215 |
| *Mch* | 0.161523160 | 0.5611455320 | 0.453799058 | 0.9860215 |
| *Npy* | 0.118464497 | 0.5779210486 | 0.447128359 | 0.9860215 |
| *Ptp1b* | 0.006527932 | 0.0018028836 | 0.966131698 | 0.9860215 |
| *Pde3b* | -0.111981145 | 0.5041362712 | 0.477688297 | 0.9860215 |
| *Pdk1* | 0.054147955 | 0.1124895502 | 0.737327427 | 0.9860215 |
| *Pias3* | -0.082762660 | 0.2729521652 | 0.601358275 | 0.9860215 |
| *Pik3ca* | -0.047909064 | 0.0830172485 | 0.773249419 | 0.9860215 |
| *Pik3r1* | 0.093019086 | 0.3393685350 | 0.560193942 | 0.9860215 |
| *Pomc* | -0.128749974 | 0.2674518971 | 0.605046206 | 0.9860215 |
| *Ramp1* | -0.065056706 | 0.1736708164 | 0.676869832 | 0.9860215 |
| *Ramp2* | 0.128168531 | 0.6552739025 | 0.418233409 | 0.9860215 |
| *Ramp3* | -0.232587994 | 2.0555805922 | 0.151649439 | 0.9860215 |
| *Socs3* | -0.056756873 | 0.0573720003 | 0.810698878 | 0.9860215 |
| *Stat3* | 0.089775709 | 0.3302811079 | 0.565493608 | 0.9860215 |
| *c-fos* | -0.183956276 | 1.2029306222 | 0.272736727 | 0.9860215 |
| *mTor* | 0.019080003 | 0.0147117785 | 0.903459661 | 0.9860215 |

### T2 Left Cortex: HOM vs NON (PM)

| **Gene** | **log_2_ FC** | **Likelihood-ratio test** | **p value** | **q value** |
| --- | --- | --- | --- | --- |
| *Adrbk1* | -0.20582673 | 2.28071800 | 1.309912e-01 | 3.827279e-01 |
| *Agrp* | 0.39874338 | 1.06698421 | 3.016276e-01 | 4.542830e-01 |
| *Akt1* | -0.08917302 | 0.43261359 | 5.107093e-01 | 6.458970e-01 |
| *Akt2* | -0.20281718 | 2.15171873 | 1.424104e-01 | 3.827279e-01 |
| *Arrb1* | -0.03153582 | 0.05200046 | 8.196182e-01 | 8.690218e-01 |
| *Arrb2* | -0.02155890 | 0.02518436 | 8.739085e-01 | 8.739085e-01 |
| *Calcr* | 2.32395387 | 52.33337148 | 4.684031e-13 | 2.014133e-11 |
| *Calcr-1a* | 6.27704845 | 0.21510042 | 6.427988e-01 | 7.677874e-01 |
| *Calcr-1b* | 0.47180455 | 0.51710738 | 4.720782e-01 | 6.151322e-01 |
| *Cart* | -0.99031955 | 38.61412249 | 5.164389e-10 | 7.402291e-09 |
| *Foxo1* | -0.36526572 | 7.08495560 | 7.773375e-03 | 6.685102e-02 |
| *Gsk3a* | -0.11600440 | 0.84916199 | 3.567895e-01 | 4.949016e-01 |
| *Gsk3b* | -0.19024969 | 2.20544237 | 1.375244e-01 | 3.827279e-01 |
| *Hcrt* | -1.04216733 | 5.26489678 | 2.175971e-02 | 1.066607e-01 |
| *Hdac5* | -0.16452249 | 1.45513413 | 2.277059e-01 | 4.286498e-01 |
| *Hdc* | -0.61172773 | 5.35655788 | 2.064448e-02 | 1.066607e-01 |
| *Hrh1* | 0.03693264 | 0.06798656 | 7.942912e-01 | 8.690218e-01 |
| *Amylin* | 0.23684556 | 0.08263422 | 7.737588e-01 | 8.690218e-01 |
| *Ins1* | -17.73801764 | 0.05047894 | 8.222320e-01 | 8.690218e-01 |
| *Ins2* | -16.50510787 | 0.03572884 | 8.500766e-01 | 8.703165e-01 |
| *Ir* | -0.17182178 | 1.52108162 | 2.174559e-01 | 4.286498e-01 |
| *Irs1* | -0.18252575 | 1.44533613 | 2.292778e-01 | 4.286498e-01 |
| *Irs2* | -0.17280530 | 1.58261913 | 2.083838e-01 | 4.286498e-01 |
| *Jak2* | -0.12162392 | 1.04623030 | 3.063769e-01 | 4.542830e-01 |
| *Lepr* | -0.25761248 | 3.34101118 | 6.757307e-02 | 2.905642e-01 |
| *Lepr-b* | 0.16270540 | 1.05530610 | 3.042882e-01 | 4.542830e-01 |
| *Mc4r* | -0.15978071 | 1.20535980 | 2.722531e-01 | 4.502647e-01 |
| *Mch* | -0.31093402 | 2.49301308 | 1.143526e-01 | 3.827279e-01 |
| *Npy* | 0.36383252 | 6.23139724 | 1.255047e-02 | 8.994501e-02 |
| *Ptp1b* | -0.15756073 | 1.32702265 | 2.493356e-01 | 4.351176e-01 |
| *Pde3b* | -0.17939971 | 1.70174281 | 1.920602e-01 | 4.286498e-01 |
| *Pdk1* | -0.21314514 | 2.30483774 | 1.289717e-01 | 3.827279e-01 |
| *Pias3* | -0.10109158 | 0.53322117 | 4.652558e-01 | 6.151322e-01 |
| *Pik3ca* | -0.12514247 | 0.92717826 | 3.355972e-01 | 4.810227e-01 |
| *Pik3r1* | -0.07447703 | 0.30056239 | 5.835301e-01 | 7.169084e-01 |
| *Pomc* | 0.04654498 | 0.04686992 | 8.286022e-01 | 8.690218e-01 |
| *Ramp1* | -0.15574444 | 1.30679721 | 2.529754e-01 | 4.351176e-01 |
| *Ramp2* | -0.20871100 | 2.24878587 | 1.337193e-01 | 3.827279e-01 |
| *Ramp3* | -0.18234662 | 1.63709108 | 2.007251e-01 | 4.286498e-01 |
| *Socs3* | 1.27118436 | 38.91024885 | 4.437448e-10 | 7.402291e-09 |
| *Stat3* | 0.32156534 | 5.22032910 | 2.232433e-02 | 1.066607e-01 |
| *c-fos* | -0.64621253 | 19.28358531 | 1.126708e-05 | 1.211212e-04 |
| *mTor* | -0.16932901 | 1.54764402 | 2.134836e-01 | 4.286498e-01 |

### T2 Right Cortex: HEM vs NON (AM)

| **Gene** | **log_2_ FC** | **Likelihood-ratio test** | **p value** | **q value** |
| --- | --- | --- | --- | --- |
| *Adrbk1* | -0.22530866 | 1.912376e+00 | 1.666996e-01 | 4.249579e-01 |
| *Agrp* | 0.35199972 | 7.605272e-01 | 3.831636e-01 | 6.102235e-01 |
| *Akt1* | -0.09461564 | 3.439866e-01 | 5.575371e-01 | 7.978194e-01 |
| *Akt2* | -0.27709510 | 2.898396e+00 | 8.866775e-02 | 2.932856e-01 |
| *Arrb1* | -0.05168546 | 9.378157e-02 | 7.594236e-01 | 8.593478e-01 |
| *Arrb2* | 0.09006895 | 3.141049e-01 | 5.751721e-01 | 7.978194e-01 |
| *Calcr* | 2.27109697 | 4.776556e+01 | 4.803602e-12 | 2.065549e-10 |
| *Calcr-1a* | 27.40913191 | 4.227816e+00 | 3.976659e-02 | 2.137454e-01 |
| *Calcr-1b* | -0.77184701 | 9.141834e-01 | 3.390069e-01 | 5.901047e-01 |
| *Cart* | -0.95522852 | 3.274580e+01 | 1.050333e-08 | 1.505477e-07 |
| *Foxo1* | -0.49911533 | 9.126482e+00 | 2.519358e-03 | 2.166648e-02 |
| *Gsk3a* | -0.05866949 | 1.227813e-01 | 7.260373e-01 | 8.437731e-01 |
| *Gsk3b* | -0.16056583 | 8.988728e-01 | 3.430841e-01 | 5.901047e-01 |
| *Hcrt* | -0.75369067 | 2.030154e+00 | 1.542050e-01 | 4.249579e-01 |
| *Hdac5* | -0.01208321 | 5.571254e-03 | 9.405005e-01 | 9.628934e-01 |
| *Hdc* | -0.10337719 | 1.636018e-01 | 6.858619e-01 | 8.437731e-01 |
| *Hrh1* | -0.09240768 | 3.150577e-01 | 5.745931e-01 | 7.978194e-01 |
| *Amylin* | 2.85494820 | 1.876908e+00 | 1.706860e-01 | 4.249579e-01 |
| *Ins1* | -29.95329979 | 3.904283e+00 | 4.816316e-02 | 2.301129e-01 |
| *Ins2* | -25.95870945 | 1.521854e+00 | 2.173391e-01 | 4.918728e-01 |
| *Ir* | -0.17182849 | 1.118489e+00 | 2.902440e-01 | 5.901047e-01 |
| *Irs1* | -0.41122928 | 5.433618e+00 | 1.975272e-02 | 1.415611e-01 |
| *Irs2* | -0.30187321 | 3.477631e+00 | 6.220375e-02 | 2.674761e-01 |
| *Jak2* | -0.23430986 | 1.926465e+00 | 1.651457e-01 | 4.249579e-01 |
| *Lepr* | -0.28332255 | 2.988559e+00 | 8.385474e-02 | 2.932856e-01 |
| *Lepr-b* | -0.41174269 | 4.928764e+00 | 2.641321e-02 | 1.622526e-01 |
| *Mc4r* | 0.06538067 | 1.489676e-01 | 6.995239e-01 | 8.437731e-01 |
| *Mch* | -0.36978498 | 3.209496e+00 | 7.321202e-02 | 2.861924e-01 |
| *Npy* | 0.57749302 | 1.039196e+01 | 1.265653e-03 | 1.360577e-02 |
| *Ptp1b* | -0.21846779 | 1.815169e+00 | 1.778893e-01 | 4.249579e-01 |
| *Pde3b* | -0.03282695 | 4.098415e-02 | 8.395685e-01 | 8.805231e-01 |
| *Pdk1* | 0.16206887 | 9.736355e-01 | 3.237752e-01 | 5.901047e-01 |
| *Pias3* | -0.00503634 | 9.712645e-04 | 9.751379e-01 | 9.751379e-01 |
| *Pik3ca* | -0.15021156 | 7.872698e-01 | 3.749270e-01 | 6.102235e-01 |
| *Pik3r1* | 0.03750871 | 5.354932e-02 | 8.169984e-01 | 8.782733e-01 |
| *Pomc* | 0.20603079 | 5.526615e-01 | 4.572322e-01 | 7.021781e-01 |
| *Ramp1* | -0.16712872 | 1.082140e+00 | 2.982193e-01 | 5.901047e-01 |
| *Ramp2* | 0.04338245 | 7.011399e-02 | 7.911709e-01 | 8.723166e-01 |
| *Ramp3* | -0.16565972 | 1.001305e+00 | 3.169949e-01 | 5.901047e-01 |
| *Socs3* | -0.09662806 | 1.746120e-01 | 6.760451e-01 | 8.437731e-01 |
| *Stat3* | 0.07787396 | 2.329918e-01 | 6.293142e-01 | 8.437731e-01 |
| *c-fos* | -1.11915482 | 4.386345e+01 | 3.521028e-11 | 7.570210e-10 |
| *mTor* | -0.05728561 | 1.269126e-01 | 7.216549e-01 | 8.437731e-01 |

### T2 Right Cortex: HEM vs NON (PM)

| **Gene** | **log_2_ FC** | **Likelihood-ratio test** | **p value** | **q value** |
| --- | --- | --- | --- | --- |
| *Adrbk1* | -0.001147373 | 6.310332e-05 | 9.936619e-01 | 9.936619e-01 |
| *Agrp* | -0.220091409 | 4.328973e-01 | 5.105707e-01 | 8.670590e-01 |
| *Akt1* | 0.011612189 | 7.507257e-03 | 9.309541e-01 | 9.531197e-01 |
| *Akt2* | -0.262880727 | 3.891432e+00 | 4.853302e-02 | 2.086920e-01 |
| *Arrb1* | 0.129448341 | 1.615422e+00 | 2.037313e-01 | 5.658987e-01 |
| *Arrb2* | -0.016628204 | 1.589342e-02 | 8.996772e-01 | 9.448572e-01 |
| *Calcr* | 2.119592145 | 7.033353e+01 | 0.000000e+00 | 0.000000e+00 |
| *Calcr-1a* | 23.094115416 | 4.054886e-01 | 5.242682e-01 | 8.670590e-01 |
| *Calcr-1b* | 0.624125542 | 1.567524e+00 | 2.105670e-01 | 5.658987e-01 |
| *Cart* | -1.063644892 | 5.497895e+01 | 1.217915e-13 | 2.618517e-12 |
| *Foxo1* | -0.314962922 | 5.625425e+00 | 1.770177e-02 | 1.087395e-01 |
| *Gsk3a* | 0.094284295 | 4.595674e-01 | 4.978262e-01 | 8.670590e-01 |
| *Gsk3b* | -0.059900928 | 1.853758e-01 | 6.667935e-01 | 9.200521e-01 |
| *Hcrt* | 0.439484937 | 1.725750e+00 | 1.889546e-01 | 5.658987e-01 |
| *Hdac5* | -0.039893677 | 9.079113e-02 | 7.631738e-01 | 9.448572e-01 |
| *Hdc* | 0.035064014 | 2.668833e-02 | 8.702306e-01 | 9.448572e-01 |
| *Hrh1* | 0.073020683 | 2.936110e-01 | 5.879158e-01 | 9.195240e-01 |
| *Amylin* | 0.308335662 | 3.301513e-02 | 8.558177e-01 | 9.448572e-01 |
| *Ins1* | -1.078984080 | 4.126113e-01 | 5.206471e-01 | 8.670590e-01 |
| *Ins2* | -24.496031762 | 5.026587e-01 | 4.783343e-01 | 8.670590e-01 |
| *Ir* | 0.115319962 | 7.748945e-01 | 3.787072e-01 | 8.570741e-01 |
| *Irs1* | 0.057445610 | 1.648942e-01 | 6.846899e-01 | 9.200521e-01 |
| *Irs2* | -0.053878244 | 1.660975e-01 | 6.836036e-01 | 9.200521e-01 |
| *Jak2* | -0.093987530 | 4.604474e-01 | 4.974149e-01 | 8.670590e-01 |
| *Lepr* | -0.187761409 | 1.957351e+00 | 1.617969e-01 | 5.658987e-01 |
| *Lepr-b* | -0.301593004 | 4.122450e+00 | 4.231776e-02 | 2.021849e-01 |
| *Mc4r* | 0.465015801 | 1.133957e+01 | 7.587277e-04 | 6.525058e-03 |
| *Mch* | 0.169521751 | 1.008797e+00 | 3.151912e-01 | 7.972483e-01 |
| *Npy* | 0.438273785 | 1.152071e+01 | 6.882496e-04 | 6.525058e-03 |
| *Ptp1b* | -0.096848868 | 5.382942e-01 | 4.631405e-01 | 8.670590e-01 |
| *Pde3b* | 0.033900275 | 6.661874e-02 | 7.963251e-01 | 9.448572e-01 |
| *Pdk1* | 0.325574529 | 6.992288e+00 | 8.186164e-03 | 5.866751e-02 |
| *Pias3* | 0.184105683 | 1.946517e+00 | 1.629627e-01 | 5.658987e-01 |
| *Pik3ca* | 0.051607287 | 1.369972e-01 | 7.112847e-01 | 9.268255e-01 |
| *Pik3r1* | 0.027698264 | 4.476943e-02 | 8.324286e-01 | 9.448572e-01 |
| *Pomc* | -0.439737009 | 4.937434e+00 | 2.628102e-02 | 1.412605e-01 |
| *Ramp1* | -0.069167416 | 2.768704e-01 | 5.987598e-01 | 9.195240e-01 |
| *Ramp2* | 0.119057765 | 8.156489e-01 | 3.664554e-01 | 8.570741e-01 |
| *Ramp3* | 0.172434512 | 1.653506e+00 | 1.984824e-01 | 5.658987e-01 |
| *Socs3* | -0.022971867 | 1.550311e-02 | 9.009103e-01 | 9.448572e-01 |
| *Stat3* | 0.055272410 | 1.790190e-01 | 6.722177e-01 | 9.200521e-01 |
| *c-fos* | 1.115886156 | 3.825365e+01 | 6.212072e-10 | 8.903970e-09 |
| *mTor* | 0.035854134 | 8.289187e-02 | 7.734160e-01 | 9.448572e-01 |

### T2 Right Cortex: HOM vs NON (AM)

| **Gene** | **log_2_ FC** | **Likelihood-ratio test** | **p value** | **q value** |
| --- | --- | --- | --- | --- |
| *Adrbk1* | -0.01690013 | 1.069384e-02 | 0.9176367566 | 0.94007784 |
| *Agrp* | 0.32980524 | 6.527794e-01 | 0.4191207197 | 0.92232855 |
| *Akt1* | -0.08409430 | 2.736587e-01 | 0.6008879597 | 0.92232855 |
| *Akt2* | -0.15453187 | 9.073874e-01 | 0.3408085684 | 0.92232855 |
| *Arrb1* | 0.10132121 | 3.572496e-01 | 0.5500377085 | 0.92232855 |
| *Arrb2* | 0.17434360 | 1.165008e+00 | 0.2804292513 | 0.92232855 |
| *Calcr* | 0.98355409 | 5.712151e+00 | 0.0168478791 | 0.18111470 |
| *Calcr-1a* | -8.53709568 | 2.608189e-01 | 0.6095581025 | 0.92232855 |
| *Calcr-1b* | 0.18045991 | 7.679246e-02 | 0.7816921699 | 0.92232855 |
| *Cart* | -0.18155443 | 1.207005e+00 | 0.2719260577 | 0.92232855 |
| *Foxo1* | 0.15618394 | 9.149520e-01 | 0.3388039145 | 0.92232855 |
| *Gsk3a* | 0.06279724 | 1.321247e-01 | 0.7162391368 | 0.92232855 |
| *Gsk3b* | 0.03435667 | 4.117330e-02 | 0.8392037796 | 0.92527596 |
| *Hcrt* | 0.50297268 | 1.554607e+00 | 0.2124566447 | 0.92232855 |
| *Hdac5* | 0.05324716 | 1.089831e-01 | 0.7413047904 | 0.92232855 |
| *Hdc* | -0.08627530 | 1.169676e-01 | 0.7323469112 | 0.92232855 |
| *Hrh1* | -0.04346180 | 7.040525e-02 | 0.7907476428 | 0.92232855 |
| *Amylin* | 4.21138371 | 1.296855e+01 | 0.0003167675 | 0.01362100 |
| *Ins1* | -24.11167082 | 3.666537e+00 | 0.0555154319 | 0.47743271 |
| *Ins2* | -0.86833975 | 1.342025e-01 | 0.7141139213 | 0.92232855 |
| *Ir* | 0.14946846 | 8.572533e-01 | 0.3545084871 | 0.92232855 |
| *Irs1* | 0.01772125 | 1.054354e-02 | 0.9182155690 | 0.94007784 |
| *Irs2* | -0.08938849 | 3.059495e-01 | 0.5801764239 | 0.92232855 |
| *Jak2* | 0.13842542 | 6.645599e-01 | 0.4149547359 | 0.92232855 |
| *Lepr* | -0.06849910 | 1.748228e-01 | 0.6758607035 | 0.92232855 |
| *Lepr-b* | -0.21865155 | 1.435196e+00 | 0.2309182681 | 0.92232855 |
| *Mc4r* | 0.53904683 | 1.020618e+01 | 0.0013997110 | 0.03009379 |
| *Mch* | -0.05788788 | 8.431538e-02 | 0.7715323478 | 0.92232855 |
| *Npy* | 0.15308354 | 7.577024e-01 | 0.3840485023 | 0.92232855 |
| *Ptp1b* | 0.04133639 | 6.515479e-02 | 0.7985266465 | 0.92232855 |
| *Pde3b* | -0.02545141 | 2.466250e-02 | 0.8752109788 | 0.94007784 |
| *Pdk1* | 0.11938364 | 5.664213e-01 | 0.4516845293 | 0.92232855 |
| *Pias3* | 0.15704382 | 9.346132e-01 | 0.3336670577 | 0.92232855 |
| *Pik3ca* | 0.03975692 | 5.469811e-02 | 0.8150810420 | 0.92232855 |
| *Pik3r1* | 0.14974537 | 8.519661e-01 | 0.3559967383 | 0.92232855 |
| *Pomc* | 0.65157197 | 6.179644e+00 | 0.0129228304 | 0.18111470 |
| *Ramp1* | 0.00122037 | 5.009033e-05 | 0.9943530575 | 0.99435306 |
| *Ramp2* | 0.26013003 | 2.542154e+00 | 0.1108434835 | 0.79437830 |
| *Ramp3* | -0.04927591 | 8.865485e-02 | 0.7658942512 | 0.92232855 |
| *Socs3* | 0.12814869 | 3.309997e-01 | 0.5650710207 | 0.92232855 |
| *Stat3* | 0.13267054 | 6.798975e-01 | 0.4096220049 | 0.92232855 |
| *c-fos* | 0.11992714 | 5.146290e-01 | 0.4731418145 | 0.92232855 |
| *mTor* | 0.06896816 | 1.842071e-01 | 0.6677823743 | 0.92232855 |

### T2 Right Cortex: HOM vs NON (PM)

| **Gene** | **log_2_ FC** | **Likelihood-ratio test** | **p value** | **q value** |
| --- | --- | --- | --- | --- |
| *Adrbk1* | -0.148790845 | 1.225419e+00 | 2.682998e-01 | 6.352070e-01 |
| *Agrp* | -0.034771581 | 1.357935e-02 | 9.072321e-01 | 9.789802e-01 |
| *Akt1* | -0.094478105 | 4.840433e-01 | 4.865965e-01 | 8.043234e-01 |
| *Akt2* | -0.290681550 | 4.496560e+00 | 3.396312e-02 | 1.644186e-01 |
| *Arrb1* | 0.095645529 | 4.748215e-01 | 4.907774e-01 | 8.043234e-01 |
| *Arrb2* | 0.056150196 | 1.753053e-01 | 6.754393e-01 | 8.949486e-01 |
| *Calcr* | 0.952961985 | 2.099412e+01 | 4.606959e-06 | 6.603308e-05 |
| *Calcr-1a* | -21.316610570 | 4.065115e-01 | 5.237454e-01 | 8.043234e-01 |
| *Calcr-1b* | 1.001640542 | 4.474056e+00 | 3.441320e-02 | 1.644186e-01 |
| *Cart* | -0.336988900 | 4.603076e+00 | 3.191464e-02 | 1.644186e-01 |
| *Foxo1* | -0.148212569 | 1.163829e+00 | 2.806728e-01 | 6.352070e-01 |
| *Gsk3a* | -0.004135267 | 9.246554e-04 | 9.757415e-01 | 9.789802e-01 |
| *Gsk3b* | -0.114263814 | 6.815696e-01 | 4.090468e-01 | 8.043234e-01 |
| *Hcrt* | -0.095721328 | 7.279018e-02 | 7.873168e-01 | 9.789802e-01 |
| *Hdac5* | -0.230763268 | 2.931186e+00 | 8.688365e-02 | 2.978742e-01 |
| *Hdc* | -0.155222686 | 5.876355e-01 | 4.433349e-01 | 8.043234e-01 |
| *Hrh1* | -0.215101828 | 2.402419e+00 | 1.211478e-01 | 3.720968e-01 |
| *Amylin* | -0.015178356 | 6.941899e-04 | 9.789802e-01 | 9.789802e-01 |
| *Ins1* | 20.662187794 | 1.224555e+00 | 2.684686e-01 | 6.352070e-01 |
| *Ins2* | -1.935453413 | 5.684101e-01 | 4.508914e-01 | 8.043234e-01 |
| *Ir* | 0.033981067 | 6.360325e-02 | 8.008889e-01 | 9.789802e-01 |
| *Irs1* | -0.063829844 | 1.919679e-01 | 6.612838e-01 | 8.949486e-01 |
| *Irs2* | -0.293749550 | 4.650602e+00 | 3.104267e-02 | 1.644186e-01 |
| *Jak2* | -0.010017341 | 7.296230e-03 | 9.319291e-01 | 9.789802e-01 |
| *Lepr* | 0.187085825 | 1.878197e+00 | 1.705393e-01 | 4.888792e-01 |
| *Lepr-b* | -0.068037637 | 2.028641e-01 | 6.524189e-01 | 8.949486e-01 |
| *Mc4r* | -0.187344238 | 1.734804e+00 | 1.877986e-01 | 5.047086e-01 |
| *Mch* | 0.027902392 | 2.686732e-02 | 8.698000e-01 | 9.789802e-01 |
| *Npy* | 0.339952142 | 5.365007e+00 | 2.054471e-02 | 1.644186e-01 |
| *Ptp1b* | -0.095631828 | 5.048670e-01 | 4.773694e-01 | 8.043234e-01 |
| *Pde3b* | -0.020748051 | 2.348601e-02 | 8.782000e-01 | 9.789802e-01 |
| *Pdk1* | 0.019128152 | 2.024455e-02 | 8.868562e-01 | 9.789802e-01 |
| *Pias3* | 0.088074032 | 4.198958e-01 | 5.169891e-01 | 8.043234e-01 |
| *Pik3ca* | 0.052204233 | 1.625484e-01 | 6.868211e-01 | 8.949486e-01 |
| *Pik3r1* | -0.055966535 | 1.733947e-01 | 6.771123e-01 | 8.949486e-01 |
| *Pomc* | 0.775404371 | 1.705166e+01 | 3.637660e-05 | 3.910485e-04 |
| *Ramp1* | -0.229132696 | 2.873390e+00 | 9.005498e-02 | 2.978742e-01 |
| *Ramp2* | 0.004817572 | 1.317379e-03 | 9.710466e-01 | 9.789802e-01 |
| *Ramp3* | -0.251480545 | 3.252751e+00 | 7.130371e-02 | 2.787327e-01 |
| *Socs3* | 1.164566661 | 3.518180e+01 | 3.003166e-09 | 1.291361e-07 |
| *Stat3* | 0.251483544 | 3.343139e+00 | 6.748575e-02 | 2.787327e-01 |
| *c-fos* | -0.773862206 | 2.685278e+01 | 2.195577e-07 | 4.720491e-06 |
| *mTor* | -0.113283428 | 7.087142e-01 | 3.998710e-01 | 8.043234e-01 |

### T3 Hindbrain: HEM vs NON (AM)

| **Gene** | **log_2_ FC** | **Likelihood-ratio test** | **p value** | **q value** |
| --- | --- | --- | --- | --- |
| *Adrbk1* | 0.125849806 | 4.709598e-01 | 4.925459e-01 | 0.5821127034 |
| *Agrp* | -2.930262791 | 6.376118e+00 | 1.156662e-02 | 0.0552627199 |
| *Akt1* | 0.426345291 | 5.827450e+00 | 1.577798e-02 | 0.0678453043 |
| *Akt2* | 0.726487100 | 1.699788e+01 | 3.742152e-05 | 0.0008045626 |
| *Arrb1* | 0.620655144 | 1.324737e+01 | 2.729630e-04 | 0.0031764721 |
| *Arrb2* | 0.435401474 | 7.163123e+00 | 7.441750e-03 | 0.0495146546 |
| *Calcr* | 0.333928247 | 8.353903e-01 | 3.607185e-01 | 0.5722259180 |
| *Calcr-1a* | 0.329625787 | 4.609308e-01 | 4.971893e-01 | 0.5821127034 |
| *Calcr-1b* | 0.377279655 | 5.985021e-01 | 4.391501e-01 | 0.5722259180 |
| *Cart* | 0.014711110 | 6.297717e-04 | 9.799790e-01 | 0.9799789864 |
| *Foxo1* | 0.381952557 | 5.454809e+00 | 1.951454e-02 | 0.0762841074 |
| *Gsk3a* | 0.320973259 | 3.669974e+00 | 5.540104e-02 | 0.1467329807 |
| *Gsk3b* | 0.311694014 | 3.593364e+00 | 5.801071e-02 | 0.1467329807 |
| *Hcrt* | -1.493457008 | 3.708141e+00 | 5.414767e-02 | 0.1467329807 |
| *Hdac5* | 0.198088848 | 9.654880e-01 | 3.258080e-01 | 0.5388363645 |
| *Hdc* | 0.278788719 | 1.128145e+00 | 2.881713e-01 | 0.5387550917 |
| *Hrh1* | -0.328198001 | 6.380714e-01 | 4.244100e-01 | 0.5722259180 |
| *Amylin* | 1.979490038 | 7.578311e-01 | 3.840081e-01 | 0.5722259180 |
| *Ins1* | 4.298293892 | 1.326009e+00 | 2.495165e-01 | 0.4876913726 |
| *Ins2* | -0.582019794 | 6.987603e-01 | 4.032006e-01 | 0.5722259180 |
| *Ir* | 0.200993592 | 7.494689e-01 | 3.866444e-01 | 0.5722259180 |
| *Irs1* | 0.241498186 | 9.748729e-01 | 3.234679e-01 | 0.5388363645 |
| *Irs2* | 0.417465618 | 6.474721e+00 | 1.094195e-02 | 0.0552627199 |
| *Jak2* | 0.350796019 | 1.374750e+00 | 2.409975e-01 | 0.4876913726 |
| *Lepr* | 0.292631480 | 5.364551e-01 | 4.639056e-01 | 0.5821127034 |
| *Lepr-b* | 0.240356294 | 1.337186e-01 | 7.146072e-01 | 0.7682027709 |
| *Mc4r* | 0.037542791 | 1.354420e-02 | 9.073518e-01 | 0.9516128157 |
| *Mch* | -0.209659881 | 2.614587e-01 | 6.091198e-01 | 0.6892671115 |
| *Npy* | 0.108631504 | 4.530551e-01 | 5.008877e-01 | 0.5821127034 |
| *Ptp1b* | 0.335875248 | 4.240773e+00 | 3.946418e-02 | 0.1305353780 |
| *Pde3b* | 0.769834193 | 4.009290e+00 | 4.525019e-02 | 0.1389827242 |
| *Pdk1* | 0.467017951 | 4.254696e+00 | 3.914195e-02 | 0.1305353780 |
| *Pias3* | 0.068633183 | 1.572209e-01 | 6.917282e-01 | 0.7626746735 |
| *Pik3ca* | 0.323323384 | 2.465102e+00 | 1.164002e-01 | 0.2780670313 |
| *Pik3r1* | -0.227548176 | 1.979714e+00 | 1.594205e-01 | 0.3437230912 |
| *Pomc* | 1.615134260 | 7.019977e+00 | 8.060525e-03 | 0.0495146546 |
| *Ramp1* | -0.278591248 | 1.010526e+00 | 3.147768e-01 | 0.5388363645 |
| *Ramp2* | 0.127079318 | 6.092921e-01 | 4.350545e-01 | 0.5722259180 |
| *Ramp3* | 0.009972924 | 2.792419e-03 | 9.578567e-01 | 0.9799789864 |
| *Socs3* | 27.290684171 | 1.710429e+01 | 3.538226e-05 | 0.0008045626 |
| *Stat3* | 0.666576792 | 8.486617e+00 | 3.577684e-03 | 0.0307680820 |
| *c-fos* | -0.522300872 | 1.975444e+00 | 1.598712e-01 | 0.3437230912 |
| *mTor* | 0.919774125 | 1.309880e+01 | 2.954858e-04 | 0.0031764721 |

### T3 Hindbrain: HEM vs NON (PM)

| **Gene** | **log_2_ FC** | **Likelihood-ratio test** | **p value** | **q value** |
| --- | --- | --- | --- | --- |
| *Adrbk1* | -0.173033173 | 1.7655002061 | 0.183939946 | 0.6084167 |
| *Agrp* | -0.804078487 | 4.8202680720 | 0.028126971 | 0.2418919 |
| *Akt1* | 0.095967944 | 0.4646160732 | 0.495474510 | 0.9917768 |
| *Akt2* | -0.001811148 | 0.0001062231 | 0.991776780 | 0.9917768 |
| *Arrb1* | 0.349248991 | 8.1051605936 | 0.004413941 | 0.1897995 |
| *Arrb2* | 0.095605299 | 0.6509395132 | 0.419776979 | 0.9666177 |
| *Calcr* | 0.436736994 | 3.4762336032 | 0.062256287 | 0.3824315 |
| *Calcr-1a* | -0.004614908 | 0.0002726899 | 0.986824872 | 0.9917768 |
| *Calcr-1b* | 0.051812150 | 0.0255708987 | 0.872952733 | 0.9917768 |
| *Cart* | 0.394324323 | 1.4642298327 | 0.226258307 | 0.6949362 |
| *Foxo1* | 0.084413151 | 0.5207676657 | 0.470514401 | 0.9917768 |
| *Gsk3a* | 0.009663708 | 0.0076929021 | 0.930107815 | 0.9917768 |
| *Gsk3b* | 0.039985716 | 0.1037272419 | 0.747401832 | 0.9917768 |
| *Hcrt* | -0.481419548 | 0.7069886957 | 0.400445308 | 0.9666177 |
| *Hdac5* | -0.221259979 | 3.2149074740 | 0.072970317 | 0.3922155 |
| *Hdc* | 0.019263792 | 0.0149200963 | 0.902781930 | 0.9917768 |
| *Hrh1* | -0.054601159 | 0.1440497287 | 0.704287770 | 0.9917768 |
| *Amylin* | -0.238676434 | 0.2021999286 | 0.652950956 | 0.9917768 |
| *Ins1* | -1.487049263 | 3.6945035363 | 0.054592027 | 0.3824315 |
| *Ins2* | 0.943481526 | 2.0555945317 | 0.151648051 | 0.5434055 |
| *Ir* | 0.046342054 | 0.0825481960 | 0.773873421 | 0.9917768 |
| *Irs1* | -0.179920821 | 1.1116599617 | 0.291721394 | 0.8362680 |
| *Irs2* | 0.101013504 | 0.7492805803 | 0.386704099 | 0.9666177 |
| *Jak2* | 0.033669538 | 0.0293207630 | 0.864040508 | 0.9917768 |
| *Lepr* | 0.191413949 | 0.6306687213 | 0.427110129 | 0.9666177 |
| *Lepr-b* | 0.205103673 | 0.2413534996 | 0.623230251 | 0.9917768 |
| *Mc4r* | 0.100855779 | 0.3560308602 | 0.550718900 | 0.9917768 |
| *Mch* | -0.006505461 | 0.0011538354 | 0.972902547 | 0.9917768 |
| *Npy* | -0.006319442 | 0.0029554893 | 0.956644832 | 0.9917768 |
| *Ptp1b* | -0.075102574 | 0.3779552791 | 0.538699573 | 0.9917768 |
| *Pde3b* | -0.040836090 | 0.0335200915 | 0.854731380 | 0.9917768 |
| *Pdk1* | 0.033951052 | 0.0488122873 | 0.825143022 | 0.9917768 |
| *Pias3* | -0.025793019 | 0.0309918443 | 0.860258585 | 0.9917768 |
| *Pik3ca* | 0.042593761 | 0.0919556399 | 0.761705532 | 0.9917768 |
| *Pik3r1* | -0.423242218 | 6.6639389445 | 0.009838321 | 0.2115239 |
| *Pomc* | 0.914560334 | 2.3932727578 | 0.121858277 | 0.4763551 |
| *Ramp1* | -0.417516836 | 5.3323671807 | 0.020932935 | 0.2250291 |
| *Ramp2* | 0.047449367 | 0.1463423628 | 0.702055523 | 0.9917768 |
| *Ramp3* | -0.241486217 | 2.5669455254 | 0.109118242 | 0.4692084 |
| *Socs3* | -0.929317281 | 5.6549832824 | 0.017405841 | 0.2250291 |
| *Stat3* | 0.248633264 | 2.8104967270 | 0.093649374 | 0.4474359 |
| *c-fos* | -0.036651370 | 0.0040114205 | 0.949499128 | 0.9917768 |
| *mTor* | 0.063937766 | 0.1778471421 | 0.673229938 | 0.9917768 |

### T3 Hindbrain: HOM vs NON (AM)

| **Gene** | **log_2_ FC** | **Likelihood-ratio test** | **p value** | **q value** |
| --- | --- | --- | --- | --- |
| *Adrbk1* | 0.28093354 | 2.247767e+00 | 0.133807345 | 0.41097970 |
| *Agrp* | -3.23376258 | 7.312122e+00 | 0.006849100 | 0.05890226 |
| *Akt1* | 0.28399109 | 3.874233e+00 | 0.049032715 | 0.25841566 |
| *Akt2* | 0.35867222 | 1.697605e+00 | 0.192601508 | 0.41174491 |
| *Arrb1* | 0.34124121 | 2.676809e+00 | 0.101819580 | 0.36485349 |
| *Arrb2* | 0.28033433 | 3.483862e+00 | 0.061969974 | 0.25841566 |
| *Calcr* | -0.19267361 | 5.887293e-01 | 0.442910912 | 0.63965847 |
| *Calcr-1a* | 0.03486924 | 5.014532e-03 | 0.943546297 | 0.98901680 |
| *Calcr-1b* | -0.40917129 | 4.885817e-01 | 0.484560674 | 0.67213255 |
| *Cart* | 0.10614084 | 7.370104e-02 | 0.786022376 | 0.86414444 |
| *Foxo1* | 0.19016596 | 1.520937e+00 | 0.217477776 | 0.41174491 |
| *Gsk3a* | 0.13734754 | 8.347781e-01 | 0.360894528 | 0.57639520 |
| *Gsk3b* | 0.27993957 | 3.476692e+00 | 0.062239044 | 0.25841566 |
| *Hcrt* | -1.80015001 | 4.148590e+00 | 0.041669199 | 0.25596793 |
| *Hdac5* | 0.22934962 | 1.519457e+00 | 0.217701654 | 0.41174491 |
| *Hdc* | 0.24645736 | 8.815750e-01 | 0.347770665 | 0.57639520 |
| *Hrh1* | -0.49304650 | 1.702862e+00 | 0.191914090 | 0.41174491 |
| *Amylin* | 1.57442662 | 3.917160e-01 | 0.531398777 | 0.70654626 |
| *Ins1* | 4.28531797 | 1.442030e+00 | 0.229811114 | 0.41174491 |
| *Ins2* | -1.67791714 | 3.377149e+00 | 0.066106331 | 0.25841566 |
| *Ir* | 0.26838056 | 1.661640e+00 | 0.197382055 | 0.41174491 |
| *Irs1* | 0.23227265 | 7.537540e-01 | 0.385290295 | 0.59169581 |
| *Irs2* | 0.42661560 | 7.469912e+00 | 0.006273860 | 0.05890226 |
| *Jak2* | 0.41335470 | 1.599536e+00 | 0.205968958 | 0.41174491 |
| *Lepr* | 0.16034299 | 1.702479e-01 | 0.679891604 | 0.79014430 |
| *Lepr-b* | -0.00424393 | 4.602168e-05 | 0.994587250 | 0.99458725 |
| *Mc4r* | 0.01051893 | 1.815186e-03 | 0.966016411 | 0.98901680 |
| *Mch* | -0.39155532 | 1.474148e+00 | 0.224692314 | 0.41174491 |
| *Npy* | 0.08055363 | 2.861899e-01 | 0.592672572 | 0.70791446 |
| *Ptp1b* | 0.44877463 | 8.382075e+00 | 0.003789396 | 0.05431468 |
| *Pde3b* | 0.45613161 | 8.312111e-01 | 0.361922565 | 0.57639520 |
| *Pdk1* | 0.16425356 | 3.714165e-01 | 0.542233180 | 0.70654626 |
| *Pias3* | -0.06408719 | 8.875420e-02 | 0.765766947 | 0.86414444 |
| *Pik3ca* | 0.34623261 | 2.397329e+00 | 0.121542626 | 0.40202561 |
| *Pik3r1* | 0.16600597 | 3.244422e-01 | 0.568950209 | 0.70791446 |
| *Pomc* | -1.46937771 | 1.096511e+01 | 0.000928434 | 0.01996133 |
| *Ramp1* | -0.30729550 | 1.529294e+00 | 0.216218375 | 0.41174491 |
| *Ramp2* | 0.03848316 | 6.168307e-02 | 0.803855293 | 0.86414444 |
| *Ramp3* | -0.12726859 | 2.981282e-01 | 0.585058237 | 0.70791446 |
| *Socs3* | 29.99835351 | 1.228733e+01 | 0.000456043 | 0.01960985 |
| *Stat3* | 0.28744899 | 5.800994e-01 | 0.446273353 | 0.63965847 |
| *c-fos* | 0.77530308 | 2.096629e+00 | 0.147624276 | 0.41174491 |
| *mTor* | 0.71543679 | 6.039535e+00 | 0.013988968 | 0.10025427 |

### T3 Hindbrain: HOM vs NON (PM)

| **Gene** | **log_2_ FC** | **Likelihood-ratio test** | **p value** | **q value** |
| --- | --- | --- | --- | --- |
| *Adrbk1* | -0.045620318 | 6.892560e-02 | 0.792907576 | 0.9867800 |
| *Agrp* | -0.865272808 | 2.033138e+00 | 0.153902537 | 0.7214803 |
| *Akt1* | 0.241394500 | 1.306518e+00 | 0.253026145 | 0.7843822 |
| *Akt2* | 0.048646059 | 2.471115e-02 | 0.875088951 | 0.9946338 |
| *Arrb1* | 0.232178240 | 1.272744e+00 | 0.259252575 | 0.7843822 |
| *Arrb2* | 0.102132633 | 3.234971e-01 | 0.569513579 | 0.9670008 |
| *Calcr* | 0.082724193 | 3.442563e-02 | 0.852804369 | 0.9946338 |
| *Calcr-1a* | -0.223957885 | 2.194836e-01 | 0.639433709 | 0.9670008 |
| *Calcr-1b* | -0.060760879 | 1.369935e-02 | 0.906825000 | 0.9953739 |
| *Cart* | -0.095772567 | 7.075925e-02 | 0.790234501 | 0.9867800 |
| *Foxo1* | 0.368385612 | 6.849790e+00 | 0.008865169 | 0.3812023 |
| *Gsk3a* | 0.045029905 | 6.224562e-02 | 0.802981214 | 0.9867800 |
| *Gsk3b* | -0.026540670 | 2.318434e-02 | 0.878978688 | 0.9946338 |
| *Hcrt* | -15.133187814 | 4.750670e+00 | 0.029286884 | 0.5882014 |
| *Hdac5* | -0.287632034 | 2.681456e+00 | 0.101522872 | 0.5882014 |
| *Hdc* | -0.014360905 | 2.850128e-03 | 0.957423891 | 0.9953739 |
| *Hrh1* | -0.156698261 | 7.172047e-01 | 0.397062367 | 0.9485379 |
| *Amylin* | -2.296022734 | 2.939511e+00 | 0.086436918 | 0.5882014 |
| *Ins1* | -2.358280419 | 2.668769e+00 | 0.102335176 | 0.5882014 |
| *Ins2* | 1.000788644 | 9.983567e-01 | 0.317708463 | 0.8538415 |
| *Ir* | 0.161447108 | 4.641646e-01 | 0.495684053 | 0.9670008 |
| *Irs1* | -0.266201585 | 9.190493e-01 | 0.337724710 | 0.8542449 |
| *Irs2* | 0.227166141 | 2.562393e+00 | 0.109432815 | 0.5882014 |
| *Jak2* | 0.131428874 | 1.427581e-01 | 0.705554385 | 0.9786722 |
| *Lepr* | 0.008631879 | 4.317608e-04 | 0.983422066 | 0.9953739 |
| *Lepr-b* | -0.320929650 | 2.743603e-01 | 0.600421739 | 0.9670008 |
| *Mc4r* | -0.240793299 | 5.797745e-01 | 0.446400726 | 0.9670008 |
| *Mch* | -0.721947192 | 3.940960e+00 | 0.047123870 | 0.5882014 |
| *Npy* | -0.087120269 | 2.685959e-01 | 0.604275221 | 0.9670008 |
| *Ptp1b* | -0.043458498 | 6.210904e-02 | 0.803193039 | 0.9867800 |
| *Pde3b* | 0.255574546 | 4.520602e-01 | 0.501358209 | 0.9670008 |
| *Pdk1* | -0.003000223 | 1.361549e-04 | 0.990690061 | 0.9953739 |
| *Pias3* | 0.110133286 | 1.689632e-01 | 0.681034875 | 0.9761500 |
| *Pik3ca* | 0.117757067 | 2.296985e-01 | 0.631747457 | 0.9670008 |
| *Pik3r1* | -0.277686053 | 1.400074e+00 | 0.236711248 | 0.7843822 |
| *Pomc* | 0.778997720 | 3.512759e+00 | 0.060897965 | 0.5882014 |
| *Ramp1* | -0.298875605 | 1.198500e+00 | 0.273621697 | 0.7843822 |
| *Ramp2* | 0.194707051 | 1.474209e+00 | 0.224682803 | 0.7843822 |
| *Ramp3* | 0.101870046 | 2.031835e-01 | 0.652163363 | 0.9670008 |
| *Socs3* | -0.002920039 | 3.361615e-05 | 0.995373936 | 0.9953739 |
| *Stat3* | 0.192150358 | 4.423052e-01 | 0.506011937 | 0.9670008 |
| *c-fos* | -1.247504167 | 1.902613e+00 | 0.167786117 | 0.7214803 |
| *mTor* | 0.189930889 | 4.158748e-01 | 0.519002666 | 0.9670008 |

### T3 Midbrain: HEM vs NON (AM)

| **Gene** | **log_2_ FC** | **Likelihood-ratio test** | **p value** | **q value** |
| --- | --- | --- | --- | --- |
| *Adrbk1* | 0.140245600 | 3.180942e+00 | 0.074501750 | 0.51059453 |
| *Agrp* | 0.295468126 | 2.905205e+00 | 0.088294032 | 0.51059453 |
| *Akt1* | 0.089027031 | 5.695558e-01 | 0.450435500 | 0.76714039 |
| *Akt2* | 0.075494889 | 3.814500e-01 | 0.536828232 | 0.79032157 |
| *Arrb1* | 0.055339214 | 3.569161e-01 | 0.550223961 | 0.79032157 |
| *Arrb2* | 0.035901565 | 9.230072e-02 | 0.761272390 | 0.88472197 |
| *Calcr* | -0.062925353 | 2.487373e-01 | 0.617965585 | 0.80713678 |
| *Calcr-1a* | -0.079960863 | 1.968743e-01 | 0.657255733 | 0.80748561 |
| *Calcr-1b* | -0.159657294 | 1.004345e+00 | 0.316261431 | 0.67996208 |
| *Cart* | -0.380474156 | 1.017501e+01 | 0.001423569 | 0.06121347 |
| *Foxo1* | 0.081011972 | 6.741699e-01 | 0.411601542 | 0.73745276 |
| *Gsk3a* | 0.112495590 | 1.558893e+00 | 0.211827435 | 0.65061284 |
| *Gsk3b* | -0.004345629 | 1.186382e-03 | 0.972523179 | 0.98933061 |
| *Hcrt* | -0.335486431 | 6.640328e+00 | 0.009969560 | 0.14289702 |
| *Hdac5* | -0.029856336 | 6.240652e-02 | 0.802731995 | 0.89734989 |
| *Hdc* | -0.155279377 | 1.261420e+00 | 0.261382563 | 0.67996208 |
| *Hrh1* | -0.001665023 | 1.788236e-04 | 0.989330610 | 0.98933061 |
| *Amylin* | -0.119983829 | 5.542699e-02 | 0.813875483 | 0.89734989 |
| *Ins1* | -0.568625093 | 4.950284e-01 | 0.481692801 | 0.76714039 |
| *Ins2* | -0.036786524 | 1.125825e-02 | 0.915499121 | 0.96236120 |
| *Ir* | 0.055700438 | 2.211025e-01 | 0.638201176 | 0.80713678 |
| *Irs1* | 0.145893379 | 1.147885e+00 | 0.283992345 | 0.67996208 |
| *Irs2* | -0.012287582 | 1.070337e-02 | 0.917600210 | 0.96236120 |
| *Jak2* | 0.198880006 | 4.115002e+00 | 0.042504472 | 0.36553846 |
| *Lepr* | 0.151247872 | 1.578666e+00 | 0.208952961 | 0.65061284 |
| *Lepr-b* | -0.048125975 | 1.077796e-01 | 0.742686305 | 0.88472197 |
| *Mc4r* | 0.085863398 | 4.968397e-01 | 0.480892019 | 0.76714039 |
| *Mch* | -0.192112026 | 2.318743e+00 | 0.127823250 | 0.61071108 |
| *Npy* | 0.250435150 | 5.995892e+00 | 0.014339227 | 0.15414669 |
| *Ptp1b* | 0.132738002 | 1.261517e+00 | 0.261364210 | 0.67996208 |
| *Pde3b* | 0.199323809 | 2.787635e+00 | 0.094994331 | 0.51059453 |
| *Pdk1* | 0.296290584 | 7.776651e+00 | 0.005292583 | 0.11379053 |
| *Pias3* | 0.058183251 | 2.297641e-01 | 0.631698774 | 0.80713678 |
| *Pik3ca* | 0.096808552 | 8.877016e-01 | 0.346100907 | 0.70642295 |
| *Pik3r1* | 0.095573952 | 1.089866e+00 | 0.296500885 | 0.67996208 |
| *Pomc* | 0.176627823 | 1.566584e+00 | 0.210703747 | 0.65061284 |
| *Ramp1* | 0.047310367 | 2.968421e-01 | 0.585868966 | 0.80713678 |
| *Ramp2* | 0.169123693 | 1.994236e+00 | 0.157898716 | 0.65061284 |
| *Ramp3* | -0.123712594 | 1.062564e+00 | 0.302631125 | 0.67996208 |
| *Socs3* | -0.212895768 | 7.455662e-01 | 0.387883642 | 0.72517377 |
| *Stat3* | 0.107367965 | 8.329333e-01 | 0.361425694 | 0.70642295 |
| *c-fos* | 0.173107642 | 1.671137e+00 | 0.196106279 | 0.65061284 |
| *mTor* | 0.070200904 | 3.548380e-01 | 0.551387143 | 0.79032157 |

### T3 Midbrain: HEM vs NON (PM)

| **Gene** | **log_2_ FC** | **Likelihood-ratio test** | **p value** | **q value** |
| --- | --- | --- | --- | --- |
| *Adrbk1* | 0.079835169 | 0.547229834 | 0.45945180 | 0.9839223 |
| *Agrp* | 0.148562707 | 0.501557882 | 0.47881640 | 0.9839223 |
| *Akt1* | 0.027506605 | 0.035985505 | 0.84954543 | 0.9839223 |
| *Akt2* | -0.050432947 | 0.119264077 | 0.72983403 | 0.9839223 |
| *Arrb1* | -0.007805895 | 0.004446910 | 0.94683235 | 0.9839223 |
| *Arrb2* | 0.046811570 | 0.143443868 | 0.70488106 | 0.9839223 |
| *Calcr* | 0.113827258 | 0.588837633 | 0.44286895 | 0.9839223 |
| *Calcr-1a* | 0.304016291 | 2.011183035 | 0.15614352 | 0.9839223 |
| *Calcr-1b* | -0.090255442 | 0.222063006 | 0.63747249 | 0.9839223 |
| *Cart* | -0.097684152 | 0.462807440 | 0.49631483 | 0.9839223 |
| *Foxo1* | -0.016622331 | 0.014007299 | 0.90578841 | 0.9839223 |
| *Gsk3a* | 0.012949826 | 0.007626228 | 0.93041058 | 0.9839223 |
| *Gsk3b* | -0.082126060 | 0.298041872 | 0.58511260 | 0.9839223 |
| *Hcrt* | -0.306127404 | 3.990570135 | 0.04575558 | 0.6558300 |
| *Hdac5* | 0.053237122 | 0.138406498 | 0.70987034 | 0.9839223 |
| *Hdc* | 0.033670202 | 0.040596786 | 0.84031823 | 0.9839223 |
| *Hrh1* | 0.064854336 | 0.197513652 | 0.65673528 | 0.9839223 |
| *Amylin* | -1.995525009 | 4.237336076 | 0.03954417 | 0.6558300 |
| *Ins1* | -0.969332327 | 1.092039753 | 0.29601971 | 0.9839223 |
| *Ins2* | 0.609849796 | 1.809785652 | 0.17853395 | 0.9839223 |
| *Ir* | -0.036770595 | 0.067620976 | 0.79483266 | 0.9839223 |
| *Irs1* | -0.140586046 | 0.721009029 | 0.39581313 | 0.9839223 |
| *Irs2* | -0.024543432 | 0.029801103 | 0.86294230 | 0.9839223 |
| *Jak2* | 0.049608489 | 0.113421474 | 0.73628196 | 0.9839223 |
| *Lepr* | 0.228446920 | 2.555812247 | 0.10988932 | 0.9839223 |
| *Lepr-b* | -0.149808294 | 0.699412403 | 0.40298121 | 0.9839223 |
| *Mc4r* | -0.044206240 | 0.091010310 | 0.76289666 | 0.9839223 |
| *Mch* | -0.021694872 | 0.020398435 | 0.88642990 | 0.9839223 |
| *Npy* | -0.020385148 | 0.018685861 | 0.89127090 | 0.9839223 |
| *Ptp1b* | 0.056527119 | 0.161128226 | 0.68811990 | 0.9839223 |
| *Pde3b* | 0.012894382 | 0.008198831 | 0.92785223 | 0.9839223 |
| *Pdk1* | 0.082101769 | 0.343843619 | 0.55761897 | 0.9839223 |
| *Pias3* | 0.002910878 | 0.000406092 | 0.98392234 | 0.9839223 |
| *Pik3ca* | 0.004854328 | 0.001034497 | 0.97434159 | 0.9839223 |
| *Pik3r1* | 0.026909993 | 0.035906654 | 0.84970839 | 0.9839223 |
| *Pomc* | 0.342920770 | 4.287420611 | 0.03839537 | 0.6558300 |
| *Ramp1* | 0.022918859 | 0.025176169 | 0.87392888 | 0.9839223 |
| *Ramp2* | 0.149848761 | 1.100383755 | 0.29418190 | 0.9839223 |
| *Ramp3* | 0.168752209 | 1.429352409 | 0.23187015 | 0.9839223 |
| *Socs3* | -0.063135432 | 0.045121245 | 0.83178124 | 0.9839223 |
| *Stat3* | 0.035688167 | 0.064926341 | 0.79887257 | 0.9839223 |
| *c-fos* | -0.036981079 | 0.042094789 | 0.83743919 | 0.9839223 |
| *mTor* | 0.083803740 | 0.596898437 | 0.43976387 | 0.9839223 |

### T3 Midbrain: HOM vs NON (AM)

| **Gene** | **log_2_ FC** | **Likelihood-ratio test** | **p value** | **q value** |
| --- | --- | --- | --- | --- |
| *Adrbk1* | 0.170268560 | 2.180959955 | 1.397273e-01 | 0.462174867 |
| *Agrp* | 0.607079241 | 15.260293084 | 9.366492e-05 | 0.004027591 |
| *Akt1* | 0.235218322 | 3.935130376 | 4.728747e-02 | 0.287822536 |
| *Akt2* | 0.216312597 | 3.530711655 | 6.024193e-02 | 0.287822536 |
| *Arrb1* | 0.216366011 | 3.212280125 | 7.308757e-02 | 0.297909517 |
| *Arrb2* | 0.033925323 | 0.088880794 | 7.656048e-01 | 0.889756982 |
| *Calcr* | 0.085119410 | 0.517834519 | 4.717669e-01 | 0.703536072 |
| *Calcr-1a* | -0.210078971 | 1.609115499 | 2.046162e-01 | 0.517558574 |
| *Calcr-1b* | -0.025897146 | 0.031984816 | 8.580610e-01 | 0.922415560 |
| *Cart* | -0.101439613 | 0.795220577 | 3.725262e-01 | 0.696462009 |
| *Foxo1* | 0.138313601 | 1.476048105 | 2.243939e-01 | 0.536051994 |
| *Gsk3a* | 0.088311903 | 0.523908402 | 4.691792e-01 | 0.703536072 |
| *Gsk3b* | 0.120433078 | 0.991051385 | 3.194855e-01 | 0.658617707 |
| *Hcrt* | 0.087813838 | 0.504181578 | 4.776685e-01 | 0.703536072 |
| *Hdac5* | 0.057955952 | 0.251782583 | 6.158227e-01 | 0.756582166 |
| *Hdc* | -0.123690384 | 0.921935831 | 3.369672e-01 | 0.658617707 |
| *Hrh1* | 0.147895407 | 1.616245519 | 2.036161e-01 | 0.517558574 |
| *Amylin* | 0.320456780 | 0.602847513 | 4.374936e-01 | 0.703536072 |
| *Ins1* | -0.059818805 | 0.009321204 | 9.230866e-01 | 0.968115254 |
| *Ins2* | 0.070723944 | 0.053697497 | 8.167499e-01 | 0.922415560 |
| *Ir* | 0.201532325 | 3.143941975 | 7.620941e-02 | 0.297909517 |
| *Irs1* | 0.247324346 | 3.852502969 | 4.967181e-02 | 0.287822536 |
| *Irs2* | 0.118983014 | 1.101549477 | 2.939263e-01 | 0.658617707 |
| *Jak2* | 0.159812403 | 1.800451430 | 1.796579e-01 | 0.515019396 |
| *Lepr* | 0.063428053 | 0.309687190 | 5.778722e-01 | 0.730838372 |
| *Lepr-b* | 0.093149246 | 0.472236627 | 4.919600e-01 | 0.703536072 |
| *Mc4r* | 0.004446136 | 0.001478624 | 9.693266e-01 | 0.969626743 |
| *Mch* | 0.042049043 | 0.119677894 | 7.293841e-01 | 0.871208789 |
| *Npy* | 0.240014974 | 3.981966621 | 4.598984e-02 | 0.287822536 |
| *Ptp1b* | 0.217030507 | 3.659143386 | 5.576230e-02 | 0.287822536 |
| *Pde3b* | 0.111236756 | 0.960618447 | 3.270311e-01 | 0.658617707 |
| *Pdk1* | 0.075080766 | 0.439838481 | 5.072004e-01 | 0.703536072 |
| *Pias3* | 0.023505984 | 0.041541307 | 8.384966e-01 | 0.922415560 |
| *Pik3ca* | 0.065588392 | 0.322216634 | 5.702786e-01 | 0.730838372 |
| *Pik3r1* | -0.066181865 | 0.349071467 | 5.546392e-01 | 0.730838372 |
| *Pomc* | 0.446510226 | 11.677012165 | 6.327707e-04 | 0.009069713 |
| *Ramp1* | -0.082999581 | 0.539653014 | 4.625766e-01 | 0.703536072 |
| *Ramp2* | 0.246066375 | 4.587350187 | 3.220877e-02 | 0.287822536 |
| *Ramp3* | -0.004330959 | 0.001449815 | 9.696267e-01 | 0.969626743 |
| *Socs3* | 0.313447985 | 2.315232331 | 1.281122e-01 | 0.459068565 |
| *Stat3* | 0.090200675 | 0.646939023 | 4.212092e-01 | 0.703536072 |
| *c-fos* | 0.449707559 | 12.059795363 | 5.152095e-04 | 0.009069713 |
| *mTor* | 0.162349707 | 2.058289266 | 1.513800e-01 | 0.464952975 |

### T3 Midbrain: HOM vs NON (PM)

| **Gene** | **log_2_ FC** | **Likelihood-ratio test** | **p value** | **q value** |
| --- | --- | --- | --- | --- |
| *Adrbk1* | 0.012251051 | 0.004546851 | 9.462391e-01 | 9.848642e-01 |
| *Agrp* | -0.164681298 | 0.441549773 | 5.063754e-01 | 8.846486e-01 |
| *Akt1* | -0.109898923 | 1.102836066 | 2.936445e-01 | 8.465917e-01 |
| *Akt2* | 0.006134044 | 0.001165683 | 9.727638e-01 | 9.848642e-01 |
| *Arrb1* | 0.085345410 | 0.203600978 | 6.518298e-01 | 9.284467e-01 |
| *Arrb2* | 0.081754547 | 0.207220405 | 6.489548e-01 | 9.284467e-01 |
| *Calcr* | -0.026238758 | 0.019755607 | 8.882218e-01 | 9.848642e-01 |
| *Calcr-1a* | -0.233431473 | 0.808920244 | 3.684397e-01 | 8.801614e-01 |
| *Calcr-1b* | -0.144277127 | 0.415248225 | 5.193177e-01 | 8.846486e-01 |
| *Cart* | -0.703296660 | 13.803197253 | 2.029905e-04 | 2.909530e-03 |
| *Foxo1* | -0.008568504 | 0.002296572 | 9.617780e-01 | 9.848642e-01 |
| *Gsk3a* | 0.059672452 | 0.095446567 | 7.573639e-01 | 9.308098e-01 |
| *Gsk3b* | -0.066934824 | 0.387166108 | 5.337928e-01 | 8.846486e-01 |
| *Hcrt* | -0.454780645 | 5.516905906 | 1.883354e-02 | 1.349737e-01 |
| *Hdac5* | -0.145363672 | 0.930630850 | 3.346991e-01 | 8.465917e-01 |
| *Hdc* | -0.550876098 | 6.769959423 | 9.270494e-03 | 7.972625e-02 |
| *Hrh1* | -0.191159538 | 1.054479231 | 3.044777e-01 | 8.465917e-01 |
| *Amylin* | 0.527732913 | 1.004216830 | 3.162923e-01 | 8.465917e-01 |
| *Ins1* | 0.372498501 | 0.315098735 | 5.745681e-01 | 8.846486e-01 |
| *Ins2* | 0.526068166 | 1.201776323 | 2.729669e-01 | 8.465917e-01 |
| *Ir* | 0.076724483 | 0.182368866 | 6.693453e-01 | 9.284467e-01 |
| *Irs1* | -0.306989216 | 2.267192773 | 1.321391e-01 | 5.681980e-01 |
| *Irs2* | -0.063451021 | 0.122757076 | 7.260632e-01 | 9.308098e-01 |
| *Jak2* | -0.049503029 | 0.096289115 | 7.563291e-01 | 9.308098e-01 |
| *Lepr* | 0.107425778 | 0.352127865 | 5.529111e-01 | 8.846486e-01 |
| *Lepr-b* | -0.129553386 | 0.374853266 | 5.403706e-01 | 8.846486e-01 |
| *Mc4r* | 0.124029393 | 0.459372098 | 4.979176e-01 | 8.846486e-01 |
| *Mch* | -0.310632563 | 4.649801930 | 3.105713e-02 | 1.810013e-01 |
| *Npy* | 0.929162542 | 24.622707419 | 6.972527e-07 | 1.499093e-05 |
| *Ptp1b* | 0.052246760 | 0.084917619 | 7.707406e-01 | 9.308098e-01 |
| *Pde3b* | 0.033386009 | 0.034153400 | 8.533809e-01 | 9.848642e-01 |
| *Pdk1* | 0.003366234 | 0.000359899 | 9.848642e-01 | 9.848642e-01 |
| *Pias3* | 0.022007104 | 0.014438961 | 9.043546e-01 | 9.848642e-01 |
| *Pik3ca* | -0.042259418 | 0.078542333 | 7.792826e-01 | 9.308098e-01 |
| *Pik3r1* | -0.192598722 | 4.015589162 | 4.508147e-02 | 2.153892e-01 |
| *Pomc* | -1.143372713 | 25.810555267 | 3.766232e-07 | 1.499093e-05 |
| *Ramp1* | -0.293725284 | 4.511146169 | 3.367467e-02 | 1.810013e-01 |
| *Ramp2* | 0.176134135 | 0.959193704 | 3.273901e-01 | 8.465917e-01 |
| *Ramp3* | -0.194853168 | 1.136280413 | 2.864397e-01 | 8.465917e-01 |
| *Socs3* | 0.730351232 | 6.873454791 | 8.748537e-03 | 7.972625e-02 |
| *Stat3* | 0.145268006 | 0.665483372 | 4.146308e-01 | 8.846486e-01 |
| *c-fos* | 0.173986766 | 0.696854275 | 4.038427e-01 | 8.846486e-01 |
| *mTor* | 0.099618087 | 0.312663613 | 5.760503e-01 | 8.846486e-01 |

### T3 Left Cortex: HEM vs NON (AM)

| **Gene** | **log_2_ FC** | **Likelihood-ratio test** | **p value** | **q value** |
| --- | --- | --- | --- | --- |
| *Adrbk1* | -0.031015931 | 6.548450e-02 | 7.980285e-01 | 0.9320027456 |
| *Agrp* | -1.150905894 | 3.861470e-01 | 5.343317e-01 | 0.9307016032 |
| *Akt1* | -0.096730757 | 6.467580e-01 | 4.212742e-01 | 0.9307016032 |
| *Akt2* | -0.083905179 | 4.569634e-01 | 4.990465e-01 | 0.9307016032 |
| *Arrb1* | -0.017669229 | 2.032426e-02 | 8.866352e-01 | 0.9429342774 |
| *Arrb2* | -0.050707350 | 1.779892e-01 | 6.731070e-01 | 0.9307016032 |
| *Calcr* | -1.125957252 | 1.195396e+00 | 2.742437e-01 | 0.8423200009 |
| *Calcr-1a* | -0.264883163 | 5.260636e-01 | 4.682665e-01 | 0.9307016032 |
| *Calcr-1b* | 0.957737680 | 4.168639e-01 | 5.185061e-01 | 0.9307016032 |
| *Cart* | -0.237541659 | 3.030996e+00 | 8.168787e-02 | 0.4390723043 |
| *Foxo1* | 0.115442728 | 8.708669e-01 | 3.507154e-01 | 0.9307016032 |
| *Gsk3a* | -0.034908361 | 7.505318e-02 | 7.841166e-01 | 0.9320027456 |
| *Gsk3b* | -0.084830452 | 4.788063e-01 | 4.889635e-01 | 0.9307016032 |
| *Hcrt* | -1.187977653 | 7.645559e-01 | 3.819065e-01 | 0.9307016032 |
| *Hdac5* | 0.014835533 | 1.523712e-02 | 9.017597e-01 | 0.9429342774 |
| *Hdc* | -0.578677299 | 4.237930e+00 | 3.953033e-02 | 0.2833007091 |
| *Hrh1* | 0.001940516 | 2.405211e-04 | 9.876263e-01 | 0.9876263089 |
| *Amylin* | -22.944358900 | 2.799008e-01 | 5.967662e-01 | 0.9307016032 |
| *Ins1* | -21.428526302 | 3.601550e-01 | 5.484202e-01 | 0.9307016032 |
| *Ins2* | -14.541346205 | 1.562689e-01 | 6.926151e-01 | 0.9307016032 |
| *Ir* | -0.012238976 | 9.834120e-03 | 9.210056e-01 | 0.9429342774 |
| *Irs1* | -0.099868091 | 4.440715e-01 | 5.051638e-01 | 0.9307016032 |
| *Irs2* | -0.288753896 | 5.531801e+00 | 1.867387e-02 | 0.2007441386 |
| *Jak2* | 0.026569115 | 4.937068e-02 | 8.241620e-01 | 0.9326043443 |
| *Lepr* | 0.602804642 | 1.877395e+01 | 1.471635e-05 | 0.0006328031 |
| *Lepr-b* | -1.052449166 | 6.707669e+00 | 9.599915e-03 | 0.1375987761 |
| *Mc4r* | 0.134092167 | 8.042292e-01 | 3.698319e-01 | 0.9307016032 |
| *Mch* | -0.632920979 | 6.884947e+00 | 8.692462e-03 | 0.1375987761 |
| *Npy* | -0.021099053 | 2.745371e-02 | 8.683996e-01 | 0.9429342774 |
| *Ptp1b* | -0.035759989 | 8.893734e-02 | 7.655325e-01 | 0.9320027456 |
| *Pde3b* | -0.032721324 | 6.857699e-02 | 7.934201e-01 | 0.9320027456 |
| *Pdk1* | 0.057997575 | 2.314605e-01 | 6.304430e-01 | 0.9307016032 |
| *Pias3* | 0.230238927 | 3.312361e+00 | 6.876062e-02 | 0.4223866911 |
| *Pik3ca* | 0.062653984 | 2.628507e-01 | 6.081684e-01 | 0.9307016032 |
| *Pik3r1* | 0.131926463 | 1.197502e+00 | 2.738215e-01 | 0.8423200009 |
| *Pomc* | 0.175029153 | 3.022192e-01 | 5.824945e-01 | 0.9307016032 |
| *Ramp1* | -0.154376793 | 1.650923e+00 | 1.988333e-01 | 0.7772576118 |
| *Ramp2* | 0.166577649 | 1.829065e+00 | 1.762380e-01 | 0.7772576118 |
| *Ramp3* | -0.170321891 | 1.661948e+00 | 1.973405e-01 | 0.7772576118 |
| *Socs3* | -0.067119489 | 6.290900e-02 | 8.019559e-01 | 0.9320027456 |
| *Stat3* | 0.145212754 | 1.434832e+00 | 2.309774e-01 | 0.8276691266 |
| *c-fos* | -0.283423563 | 4.544782e+00 | 3.301922e-02 | 0.2833007091 |
| *mTor* | 0.051175656 | 1.825708e-01 | 6.691732e-01 | 0.9307016032 |

### T3 Left Cortex: HEM vs NON (PM)

| **Gene** | **log_2_ FC** | **Likelihood-ratio test** | **p value** | **q value** |
| --- | --- | --- | --- | --- |
| *Adrbk1* | -0.12897008 | 0.98061906 | 0.3220460176 | 0.717488164 |
| *Agrp* | -0.11795910 | 0.02912492 | 0.8644909273 | 0.906422236 |
| *Akt1* | -0.14975338 | 1.78557634 | 0.1814660721 | 0.606019542 |
| *Akt2* | -0.03818935 | 0.06786829 | 0.7944662201 | 0.899001249 |
| *Arrb1* | 0.01954985 | 0.01713287 | 0.8958602833 | 0.906422236 |
| *Arrb2* | -0.09844271 | 0.47253237 | 0.4918244602 | 0.802704516 |
| *Calcr* | -1.08152571 | 0.15148888 | 0.6971165702 | 0.867301360 |
| *Calcr-1a* | 3.39976053 | 0.18762082 | 0.6649042059 | 0.867301360 |
| *Calcr-1b* | 0.71259742 | 0.51344231 | 0.4736524909 | 0.802704516 |
| *Cart* | -0.32927899 | 4.42597687 | 0.0353958463 | 0.302704808 |
| *Foxo1* | -0.21498682 | 1.91430655 | 0.1664856640 | 0.606019542 |
| *Gsk3a* | 0.05582531 | 0.13076545 | 0.7176397137 | 0.867301360 |
| *Gsk3b* | -0.09484916 | 0.87200370 | 0.3504011964 | 0.717488164 |
| *Hcrt* | -1.12455551 | 1.99524107 | 0.1577939562 | 0.606019542 |
| *Hdac5* | -0.19235719 | 3.76431601 | 0.0523572678 | 0.321623217 |
| *Hdc* | -0.22045411 | 0.38201837 | 0.5365250149 | 0.823949130 |
| *Hrh1* | -0.54653389 | 12.40150901 | 0.0004289870 | 0.009434657 |
| *Amylin* | 20.28988245 | 1.04434010 | 0.3068142228 | 0.717488164 |
| *Ins1* | -10.19870215 | 0.31071401 | 0.5772423710 | 0.855911102 |
| *Ins2* | -24.88633917 | 2.77627406 | 0.0956705017 | 0.514228946 |
| *Ir* | -0.05543961 | 0.14413135 | 0.7042079492 | 0.867301360 |
| *Irs1* | -0.27297436 | 2.45373786 | 0.1172453766 | 0.560172355 |
| *Irs2* | -0.18642844 | 1.57576495 | 0.2093717743 | 0.643070449 |
| *Jak2* | 0.15810341 | 1.20964281 | 0.2714028743 | 0.717488164 |
| *Lepr* | -0.35060169 | 4.12564645 | 0.0422378802 | 0.302704808 |
| *Lepr-b* | -0.45251163 | 1.77134906 | 0.1832152102 | 0.606019542 |
| *Mc4r* | -0.08451872 | 0.23924031 | 0.6247553389 | 0.867301360 |
| *Mch* | -0.61556717 | 6.08010129 | 0.0136712932 | 0.195955202 |
| *Npy* | 0.14724700 | 0.91261614 | 0.3394212322 | 0.717488164 |
| *Ptp1b* | -0.09689682 | 0.44645391 | 0.5040237657 | 0.802704516 |
| *Pde3b* | 0.01769691 | 0.01381859 | 0.9064222359 | 0.906422236 |
| *Pdk1* | -0.02017832 | 0.02342934 | 0.8783458748 | 0.906422236 |
| *Pias3* | -0.05409129 | 0.13014984 | 0.7182767306 | 0.867301360 |
| *Pik3ca* | 0.10089475 | 0.47025869 | 0.4928681819 | 0.802704516 |
| *Pik3r1* | -0.13344643 | 0.88472939 | 0.3469096023 | 0.717488164 |
| *Pomc* | -0.75645746 | 5.49191336 | 0.0191046242 | 0.205374710 |
| *Ramp1* | -0.35322142 | 12.35918916 | 0.0004388212 | 0.009434657 |
| *Ramp2* | -0.11873797 | 0.66058924 | 0.4163519975 | 0.800234482 |
| *Ramp3* | 0.02061991 | 0.01773418 | 0.8940591427 | 0.906422236 |
| *Socs3* | -0.08072730 | 0.06859878 | 0.7933879890 | 0.899001249 |
| *Stat3* | -0.05038944 | 0.12271084 | 0.7261127667 | 0.867301360 |
| *c-fos* | 0.18504141 | 0.62815631 | 0.4280323974 | 0.800234482 |
| *mTor* | -0.15021960 | 1.09949236 | 0.2943775373 | 0.717488164 |

### T3 Left Cortex: HOM vs NON (AM)

| **Gene** | **log_2_ FC** | **Likelihood-ratio test** | **p value** | **q value** |
| --- | --- | --- | --- | --- |
| *Adrbk1* | 0.001239261 | 1.771258e-04 | 9.893814e-01 | 0.989381378 |
| *Agrp* | 0.998959863 | 1.335697e+00 | 2.477943e-01 | 0.475863595 |
| *Akt1* | -0.063474137 | 3.019019e-01 | 5.826925e-01 | 0.736934690 |
| *Akt2* | 0.109512852 | 9.167204e-01 | 3.383376e-01 | 0.542233933 |
| *Arrb1* | 0.206787583 | 2.943329e+00 | 8.623287e-02 | 0.348331381 |
| *Arrb2* | 0.056790772 | 2.504403e-01 | 6.167652e-01 | 0.757740117 |
| *Calcr* | 0.724600244 | 1.671553e+00 | 1.960507e-01 | 0.438316794 |
| *Calcr-1a* | -4.658486799 | 6.319223e-01 | 4.266511e-01 | 0.625255032 |
| *Calcr-1b* | 0.951799080 | 5.229139e-01 | 4.696013e-01 | 0.639976079 |
| *Cart* | -0.446505603 | 4.316524e+00 | 3.774395e-02 | 0.321728901 |
| *Foxo1* | 0.177764425 | 1.978953e+00 | 1.595007e-01 | 0.420851510 |
| *Gsk3a* | 0.078893011 | 4.074519e-01 | 5.232657e-01 | 0.681831031 |
| *Gsk3b* | 0.124785327 | 1.088248e+00 | 2.968597e-01 | 0.510598653 |
| *Hcrt* | 1.177554020 | 3.478794e+00 | 6.216001e-02 | 0.321728901 |
| *Hdac5* | 0.024903582 | 4.631686e-02 | 8.296008e-01 | 0.891820908 |
| *Hdc* | -0.456982201 | 3.485575e+00 | 6.190584e-02 | 0.321728901 |
| *Hrh1* | 0.094138094 | 5.074121e-01 | 4.762613e-01 | 0.639976079 |
| *Amylin* | 1.513022210 | 6.061940e-01 | 4.362244e-01 | 0.625255032 |
| *Ins1* | -0.135669772 | 5.903724e-04 | 9.806152e-01 | 0.989381378 |
| *Ins2* | -5.275618270 | 1.185103e-01 | 7.306559e-01 | 0.826794826 |
| *Ir* | 0.178983335 | 2.661632e+00 | 1.027952e-01 | 0.348331381 |
| *Irs1* | 0.214584525 | 2.502564e+00 | 1.136611e-01 | 0.349101915 |
| *Irs2* | -0.060301815 | 2.116827e-01 | 6.454517e-01 | 0.770956224 |
| *Jak2* | 0.228613086 | 3.863238e+00 | 4.935499e-02 | 0.321728901 |
| *Lepr* | 0.674784958 | 1.720588e+01 | 3.353975e-05 | 0.001442209 |
| *Lepr-b* | 0.035554549 | 1.481661e-02 | 9.031180e-01 | 0.947172543 |
| *Mc4r* | 0.179867614 | 1.273792e+00 | 2.590565e-01 | 0.475863595 |
| *Mch* | -0.252248889 | 1.496095e+00 | 2.212732e-01 | 0.453083237 |
| *Npy* | 0.222978657 | 2.343172e+00 | 1.258331e-01 | 0.360721573 |
| *Ptp1b* | 0.107811924 | 9.086515e-01 | 3.404725e-01 | 0.542233933 |
| *Pde3b* | 0.125563559 | 1.239340e+00 | 2.655983e-01 | 0.475863595 |
| *Pdk1* | 0.220033946 | 2.733882e+00 | 9.824021e-02 | 0.348331381 |
| *Pias3* | 0.272800570 | 5.884025e+00 | 1.527886e-02 | 0.298554356 |
| *Pik3ca* | 0.192773081 | 2.623236e+00 | 1.053095e-01 | 0.348331381 |
| *Pik3r1* | 0.148129048 | 1.614444e+00 | 2.038683e-01 | 0.438316794 |
| *Pomc* | -0.588379483 | 3.346731e+00 | 6.733861e-02 | 0.321728901 |
| *Ramp1* | -0.199010857 | 3.589074e+00 | 5.816065e-02 | 0.321728901 |
| *Ramp2* | 0.165169895 | 1.829637e+00 | 1.761704e-01 | 0.420851510 |
| *Ramp3* | -0.187741461 | 1.843551e+00 | 1.745353e-01 | 0.420851510 |
| *Socs3* | -0.061110704 | 6.318918e-02 | 8.015245e-01 | 0.883732163 |
| *Stat3* | 0.262028972 | 5.341012e+00 | 2.082937e-02 | 0.298554356 |
| *c-fos* | 0.084404123 | 1.456337e-01 | 7.027434e-01 | 0.816701797 |
| *mTor* | 0.100709893 | 8.071083e-01 | 3.689766e-01 | 0.566642567 |

### T3 Left Cortex: HOM vs NON (PM)

| **Gene** | **log_2_ FC** | **Likelihood-ratio test** | **p value** | **q value** |
| --- | --- | --- | --- | --- |
| *Adrbk1* | -0.26279744 | 6.895467e+00 | 8.641460e-03 | 0.0341011577 |
| *Agrp* | 0.01451325 | 3.385466e-04 | 9.853200e-01 | 0.9853200438 |
| *Akt1* | -0.35811495 | 1.268250e+01 | 3.690930e-04 | 0.0039677498 |
| *Akt2* | -0.30166227 | 1.071508e+01 | 1.062658e-03 | 0.0076157181 |
| *Arrb1* | 0.04935602 | 6.987731e-02 | 7.915155e-01 | 0.8781616383 |
| *Arrb2* | -0.17416079 | 2.336659e+00 | 1.263603e-01 | 0.2469769182 |
| *Calcr* | 1.38946795 | 8.194970e-01 | 3.653273e-01 | 0.6041951074 |
| *Calcr-1a* | -4.98895560 | 1.439936e-01 | 7.043427e-01 | 0.8781616383 |
| *Calcr-1b* | -2.47334501 | 7.726304e-01 | 3.794045e-01 | 0.6042368617 |
| *Cart* | -0.92034786 | 1.570852e+01 | 7.389050e-05 | 0.0015886457 |
| *Foxo1* | 0.01674534 | 5.112342e-03 | 9.429993e-01 | 0.9654516796 |
| *Gsk3a* | -0.16990395 | 3.037780e+00 | 8.134711e-02 | 0.1943291963 |
| *Gsk3b* | -0.18803190 | 4.170755e+00 | 4.112747e-02 | 0.1263201016 |
| *Hcrt* | -0.98569556 | 1.168376e+00 | 2.797352e-01 | 0.5011922931 |
| *Hdac5* | -0.33327105 | 1.129731e+01 | 7.761935e-04 | 0.0066752640 |
| *Hdc* | -0.35775028 | 6.818951e-01 | 4.089349e-01 | 0.6280071846 |
| *Hrh1* | -0.51212082 | 6.519512e+00 | 1.066973e-02 | 0.0382331990 |
| *Amylin* | 25.27682275 | 3.825907e+00 | 5.046602e-02 | 0.1406047408 |
| *Ins1* | -1.87944407 | 1.827408e-01 | 6.690284e-01 | 0.8781616383 |
| *Ins2* | -29.13446224 | 3.409806e+00 | 6.481007e-02 | 0.1639313600 |
| *Ir* | -0.01254147 | 1.069347e-02 | 9.176382e-01 | 0.9624010203 |
| *Irs1* | -0.46317721 | 5.317038e+00 | 2.111788e-02 | 0.0698514604 |
| *Irs2* | -0.31637420 | 2.887577e+00 | 8.926510e-02 | 0.2020210128 |
| *Jak2* | 0.04627474 | 6.652036e-02 | 7.964722e-01 | 0.8781616383 |
| *Lepr* | -0.09053382 | 9.400877e-02 | 7.591414e-01 | 0.8781616383 |
| *Lepr-b* | -0.41216955 | 1.030209e+00 | 3.101095e-01 | 0.5333883634 |
| *Mc4r* | 0.34765531 | 2.604044e+00 | 1.065914e-01 | 0.2182586244 |
| *Mch* | -0.84284451 | 7.531738e+00 | 6.062138e-03 | 0.0289635499 |
| *Npy* | 0.72561535 | 1.457079e+01 | 1.349907e-04 | 0.0019348672 |
| *Ptp1b* | -0.32194759 | 9.446681e+00 | 2.115315e-03 | 0.0113698178 |
| *Pde3b* | -0.04035394 | 4.584272e-02 | 8.304620e-01 | 0.8927465964 |
| *Pdk1* | 0.07287189 | 1.675586e-01 | 6.822908e-01 | 0.8781616383 |
| *Pias3* | -0.06288222 | 1.137458e-01 | 7.359193e-01 | 0.8781616383 |
| *Pik3ca* | -0.05066373 | 7.538654e-02 | 7.836496e-01 | 0.8781616383 |
| *Pik3r1* | -0.26986945 | 6.878566e+00 | 8.723552e-03 | 0.0341011577 |
| *Pomc* | -1.52248780 | 1.027012e+01 | 1.352024e-03 | 0.0083052873 |
| *Ramp1* | -0.48941230 | 2.036669e+01 | 6.393286e-06 | 0.0002749113 |
| *Ramp2* | 0.05712742 | 1.137583e-01 | 7.359052e-01 | 0.8781616383 |
| *Ramp3* | -0.36383731 | 3.765569e+00 | 5.231804e-02 | 0.1406047408 |
| *Socs3* | -0.52969668 | 1.674259e+00 | 1.956890e-01 | 0.3658534214 |
| *Stat3* | 0.10237153 | 3.548927e-01 | 5.513565e-01 | 0.7902776492 |
| *c-fos* | -0.65372549 | 2.690764e+00 | 1.009314e-01 | 0.2170025147 |
| *mTor* | -0.10272646 | 4.848461e-01 | 4.862354e-01 | 0.7209697370 |

### T3 Right Cortex: HEM vs NON (AM)

| **Gene** | **log_2_ FC** | **Likelihood-ratio test** | **p value** | **q value** |
| --- | --- | --- | --- | --- |
| *Adrbk1* | 0.091178050 | 5.608272e-01 | 0.453927163 | 0.75270684 |
| *Agrp* | 1.050232856 | 1.286550e+00 | 0.256684808 | 0.65114089 |
| *Akt1* | 0.002794068 | 5.308168e-04 | 0.981618795 | 0.98161879 |
| *Akt2* | 0.106632284 | 7.021772e-01 | 0.402053093 | 0.72951614 |
| *Arrb1* | 0.106032708 | 7.229479e-01 | 0.395178658 | 0.72951614 |
| *Arrb2* | 0.008650921 | 4.895913e-03 | 0.944216902 | 0.96669826 |
| *Calcr* | 2.205094834 | 2.643516e+00 | 0.103973201 | 0.55885596 |
| *Calcr-1a* | 4.159076731 | 4.678569e-01 | 0.493974767 | 0.75270684 |
| *Calcr-1b* | -0.975560668 | 4.037859e-01 | 0.525140510 | 0.75270684 |
| *Cart* | -0.279045621 | 4.773632e+00 | 0.028898803 | 0.29581044 |
| *Foxo1* | 0.150718611 | 1.363395e+00 | 0.242949900 | 0.65114089 |
| *Gsk3a* | 0.068606662 | 2.839988e-01 | 0.594092138 | 0.75270684 |
| *Gsk3b* | 0.116752266 | 8.824477e-01 | 0.347532144 | 0.71161344 |
| *Hcrt* | 0.202552144 | 1.156825e-01 | 0.733765148 | 0.80429318 |
| *Hdac5* | 0.100593828 | 6.870436e-01 | 0.407171797 | 0.72951614 |
| *Hdc* | 0.863075527 | 1.022767e+01 | 0.001383492 | 0.05949016 |
| *Hrh1* | 0.123988008 | 9.013642e-01 | 0.342416178 | 0.71161344 |
| *Amylin* | -1.747095806 | 1.433993e+00 | 0.231113814 | 0.65114089 |
| *Ins1* | 5.479100865 | 2.496600e-01 | 0.617314567 | 0.75270684 |
| *Ins2* | 21.635496240 | 2.275296e-01 | 0.633361687 | 0.75270684 |
| *Ir* | 0.073215875 | 3.426016e-01 | 0.558331347 | 0.75270684 |
| *Irs1* | 0.254758177 | 2.887604e+00 | 0.089263592 | 0.54833349 |
| *Irs2* | 0.031974150 | 6.471307e-02 | 0.799196095 | 0.83818127 |
| *Jak2* | 0.039140426 | 1.030670e-01 | 0.748179706 | 0.80429318 |
| *Lepr* | 0.189622930 | 1.675863e+00 | 0.195475052 | 0.65114089 |
| *Lepr-b* | 0.596985653 | 3.540714e+00 | 0.059879665 | 0.42913760 |
| *Mc4r* | 0.104628884 | 4.747356e-01 | 0.490816612 | 0.75270684 |
| *Mch* | 0.573177337 | 7.875923e+00 | 0.005009730 | 0.10770918 |
| *Npy* | 0.055935813 | 1.895885e-01 | 0.663259355 | 0.75270684 |
| *Ptp1b* | 0.053283959 | 1.872888e-01 | 0.665182785 | 0.75270684 |
| *Pde3b* | 0.185653205 | 2.136746e+00 | 0.143806615 | 0.61836845 |
| *Pdk1* | 0.266696797 | 4.474883e+00 | 0.034396562 | 0.29581044 |
| *Pias3* | 0.147901250 | 1.361386e+00 | 0.243297484 | 0.65114089 |
| *Pik3ca* | 0.084735889 | 4.771869e-01 | 0.489699287 | 0.75270684 |
| *Pik3r1* | 0.185539741 | 2.333021e+00 | 0.126655882 | 0.60513366 |
| *Pomc* | 0.646586374 | 5.515023e+00 | 0.018853823 | 0.27023814 |
| *Ramp1* | 0.070059162 | 3.297748e-01 | 0.565791710 | 0.75270684 |
| *Ramp2* | 0.062702188 | 2.442531e-01 | 0.621151051 | 0.75270684 |
| *Ramp3* | -0.127809016 | 9.429146e-01 | 0.331529408 | 0.71161344 |
| *Socs3* | 0.153411339 | 3.776444e-01 | 0.538866620 | 0.75270684 |
| *Stat3* | 0.139551869 | 1.270771e+00 | 0.259622098 | 0.65114089 |
| *c-fos* | -0.175595621 | 1.687395e+00 | 0.193944670 | 0.65114089 |
| *mTor* | 0.134293630 | 1.203764e+00 | 0.272570604 | 0.65114089 |

### T3 Right Cortex: HEM vs NON (PM)

| **Gene** | **log_2_ FC** | **Likelihood-ratio test** | **p value** | **q value** |
| --- | --- | --- | --- | --- |
| *Adrbk1* | 0.046659590 | 1.056733e-01 | 0.7451248265 | 0.97780658 |
| *Agrp* | -0.400653837 | 2.063154e-01 | 0.6496708140 | 0.97780658 |
| *Akt1* | 0.053710761 | 1.406952e-01 | 0.7075909040 | 0.97780658 |
| *Akt2* | -0.009664157 | 4.407590e-03 | 0.9470675841 | 0.97780658 |
| *Arrb1* | -0.013380323 | 8.217688e-03 | 0.9277695361 | 0.97780658 |
| *Arrb2* | 0.013738343 | 9.357896e-03 | 0.9229358754 | 0.97780658 |
| *Calcr* | -21.966211575 | 8.027364e-01 | 0.3702764988 | 0.97780658 |
| *Calcr-1a* | 8.537095678 | 3.336164e-01 | 0.5635373646 | 0.97780658 |
| *Calcr-1b* | 0.391529023 | 8.287871e-02 | 0.7734335216 | 0.97780658 |
| *Cart* | -0.431855257 | 7.511724e+00 | 0.0061298684 | 0.13179217 |
| *Foxo1* | -0.086113726 | 3.542318e-01 | 0.5517273199 | 0.97780658 |
| *Gsk3a* | 0.057693932 | 1.455641e-01 | 0.7028110514 | 0.97780658 |
| *Gsk3b* | -0.036467923 | 6.831830e-02 | 0.7938012505 | 0.97780658 |
| *Hcrt* | -0.391578318 | 1.273643e-01 | 0.7211806683 | 0.97780658 |
| *Hdac5* | 0.086777771 | 3.721484e-01 | 0.5418355385 | 0.97780658 |
| *Hdc* | 0.347292536 | 8.731023e-01 | 0.3500978963 | 0.97780658 |
| *Hrh1* | -0.070239116 | 2.150269e-01 | 0.6428555944 | 0.97780658 |
| *Amylin* | -24.781168252 | 1.122757e+00 | 0.2893256165 | 0.97780658 |
| *Ins1* | -0.139676331 | 4.094780e-01 | 0.5222345562 | 0.97780658 |
| *Ins2* | -2.888528302 | 1.261546e-01 | 0.7224528678 | 0.97780658 |
| *Ir* | -0.004017002 | 7.738918e-04 | 0.9778065838 | 0.97780658 |
| *Irs1* | -0.072613274 | 1.650152e-01 | 0.6845805110 | 0.97780658 |
| *Irs2* | 0.065049936 | 2.014410e-01 | 0.6535601920 | 0.97780658 |
| *Jak2* | 0.059461891 | 1.773274e-01 | 0.6736802015 | 0.97780658 |
| *Lepr* | 0.006751876 | 1.765100e-03 | 0.9664882631 | 0.97780658 |
| *Lepr-b* | -1.713189283 | 1.228234e+01 | 0.0004572638 | 0.01966235 |
| *Mc4r* | -0.006314029 | 1.312685e-03 | 0.9710981760 | 0.97780658 |
| *Mch* | -0.416322643 | 2.115081e+00 | 0.1458543044 | 0.97780658 |
| *Npy* | -0.009302286 | 3.873793e-03 | 0.9503718662 | 0.97780658 |
| *Ptp1b* | 0.034808223 | 6.027171e-02 | 0.8060670065 | 0.97780658 |
| *Pde3b* | 0.019939923 | 1.838923e-02 | 0.8921320561 | 0.97780658 |
| *Pdk1* | -0.048230398 | 1.159552e-01 | 0.7334635235 | 0.97780658 |
| *Pias3* | -0.072483262 | 2.363771e-01 | 0.6268351059 | 0.97780658 |
| *Pik3ca* | 0.097859700 | 4.576008e-01 | 0.4987473744 | 0.97780658 |
| *Pik3r1* | -0.046517253 | 1.073566e-01 | 0.7431738705 | 0.97780658 |
| *Pomc* | -0.669561341 | 1.882566e+00 | 0.1700427587 | 0.97780658 |
| *Ramp1* | -0.098870861 | 4.760905e-01 | 0.4901985148 | 0.97780658 |
| *Ramp2* | -0.008626044 | 3.544495e-03 | 0.9525254631 | 0.97780658 |
| *Ramp3* | 0.259308828 | 2.824582e+00 | 0.0928310388 | 0.97780658 |
| *Socs3* | -0.425238868 | 1.381701e+00 | 0.2398116242 | 0.97780658 |
| *Stat3* | -0.015808990 | 1.216501e-02 | 0.9121753541 | 0.97780658 |
| *c-fos* | 0.095675254 | 2.741785e-01 | 0.6005424847 | 0.97780658 |
| *mTor* | -0.131430578 | 8.493830e-01 | 0.3567269561 | 0.97780658 |

### T3 Right Cortex: HOM vs NON (AM)

| **Gene** | **log_2_ FC** | **Likelihood-ratio test** | **p value** | **q value** |
| --- | --- | --- | --- | --- |
| *Adrbk1* | 0.21262214 | 3.2911533 | 0.069654082 | 0.1939990 |
| *Agrp* | 0.59726775 | 0.3099778 | 0.577693832 | 0.6509432 |
| *Akt1* | 0.23028979 | 3.8711421 | 0.049123083 | 0.1939990 |
| *Akt2* | 0.22103153 | 3.4210866 | 0.064368633 | 0.1939990 |
| *Arrb1* | 0.20378544 | 2.9035295 | 0.088385827 | 0.1998265 |
| *Arrb2* | 0.06093662 | 0.2760267 | 0.599317374 | 0.6509432 |
| *Calcr* | 2.40663485 | 3.4253172 | 0.064203907 | 0.1939990 |
| *Calcr-1a* | 0.00000000 | 0.9343245 | 0.333741730 | 0.4629321 |
| *Calcr-1b* | -2.40042775 | 1.1569554 | 0.282097869 | 0.4043403 |
| *Cart* | -0.27551130 | 4.1351238 | 0.042001994 | 0.1939990 |
| *Foxo1* | 0.07100967 | 0.3604923 | 0.548232949 | 0.6509432 |
| *Gsk3a* | 0.06979435 | 0.3262632 | 0.567867786 | 0.6509432 |
| *Gsk3b* | 0.15147707 | 1.6293353 | 0.201795004 | 0.3114432 |
| *Hcrt* | 0.30085958 | 0.2667377 | 0.605528601 | 0.6509432 |
| *Hdac5* | 0.18548791 | 2.5494165 | 0.110334998 | 0.2156548 |
| *Hdc* | 0.54737993 | 3.8637071 | 0.049341196 | 0.1939990 |
| *Hrh1* | 0.26742809 | 4.7857976 | 0.028695356 | 0.1939990 |
| *Amylin* | -1.98989048 | 1.6199334 | 0.203101075 | 0.3114432 |
| *Ins1* | -9.29782855 | 0.1450166 | 0.703343950 | 0.7200902 |
| *Ins2* | -10.54181427 | 0.2088916 | 0.647637522 | 0.6792296 |
| *Ir* | 0.03975258 | 0.1133103 | 0.736406439 | 0.7364064 |
| *Irs1* | 0.40188714 | 7.8046730 | 0.005211129 | 0.1256873 |
| *Irs2* | 0.32721852 | 7.5971935 | 0.005845923 | 0.1256873 |
| *Jak2* | 0.14840064 | 1.6542586 | 0.198380296 | 0.3114432 |
| *Lepr* | 0.08291417 | 0.4170670 | 0.518404170 | 0.6509432 |
| *Lepr-b* | 0.69313347 | 5.0810747 | 0.024188372 | 0.1939990 |
| *Mc4r* | 0.17585645 | 1.7059457 | 0.191512247 | 0.3114432 |
| *Mch* | 0.34170763 | 2.8226550 | 0.092942554 | 0.1998265 |
| *Npy* | 0.21646142 | 3.0621791 | 0.080134138 | 0.1998265 |
| *Ptp1b* | 0.19201357 | 2.6953497 | 0.100641403 | 0.2060753 |
| *Pde3b* | 0.23125055 | 3.6888382 | 0.054777760 | 0.1939990 |
| *Pdk1* | 0.21710385 | 3.4234476 | 0.064276648 | 0.1939990 |
| *Pias3* | 0.09154326 | 0.5720874 | 0.449430645 | 0.6039224 |
| *Pik3ca* | 0.21455233 | 3.2892177 | 0.069736242 | 0.1939990 |
| *Pik3r1* | 0.14461458 | 1.5711293 | 0.210043081 | 0.3114432 |
| *Pomc* | 0.37479951 | 1.7776717 | 0.182435491 | 0.3114432 |
| *Ramp1* | 0.18169094 | 2.4132253 | 0.120314250 | 0.2249353 |
| *Ramp2* | 0.21505613 | 3.2326070 | 0.072185689 | 0.1939990 |
| *Ramp3* | -0.07911734 | 0.3997921 | 0.527196656 | 0.6509432 |
| *Socs3* | 0.14241608 | 0.3377753 | 0.561116166 | 0.6509432 |
| *Stat3* | 0.21088351 | 3.2507671 | 0.071390041 | 0.1939990 |
| *c-fos* | 0.23629840 | 2.9234669 | 0.087300112 | 0.1998265 |
| *mTor* | 0.24714748 | 4.5193550 | 0.033513463 | 0.1939990 |

### T3 Right Cortex: HOM vs NON (PM)

| **Gene** | **log_2_ FC** | **Likelihood-ratio test** | **p value** | **q value** |
| --- | --- | --- | --- | --- |
| *Adrbk1* | 0.17422164 | 0.927384025 | 3.355436e-01 | 8.642719e-01 |
| *Agrp* | 0.25201478 | 0.096989463 | 7.554728e-01 | 8.946198e-01 |
| *Akt1* | 0.16473927 | 0.831676335 | 3.617882e-01 | 8.642719e-01 |
| *Akt2* | 0.28014459 | 2.385348790 | 1.224776e-01 | 7.513546e-01 |
| *Arrb1* | 0.27368548 | 2.180303481 | 1.397869e-01 | 7.513546e-01 |
| *Arrb2* | 0.09323728 | 0.272269348 | 6.018135e-01 | 8.946198e-01 |
| *Calcr* | -1.09765886 | 0.056085637 | 8.127933e-01 | 8.961567e-01 |
| *Calcr-1a* | 22.96198906 | 0.195531757 | 6.583519e-01 | 8.946198e-01 |
| *Calcr-1b* | 0.13096745 | 0.007090251 | 9.328945e-01 | 9.328945e-01 |
| *Cart* | -0.30393144 | 2.549755971 | 1.103113e-01 | 7.513546e-01 |
| *Foxo1* | 0.06557433 | 0.130039308 | 7.183913e-01 | 8.946198e-01 |
| *Gsk3a* | 0.07559855 | 0.157226123 | 6.917233e-01 | 8.946198e-01 |
| *Gsk3b* | 0.09886182 | 0.286356894 | 5.925646e-01 | 8.946198e-01 |
| *Hcrt* | -0.59721131 | 0.217999109 | 6.405687e-01 | 8.946198e-01 |
| *Hdac5* | -0.01894900 | 0.011134595 | 9.159627e-01 | 9.328945e-01 |
| *Hdc* | 0.12764879 | 0.085644431 | 7.697892e-01 | 8.946198e-01 |
| *Hrh1* | 0.06878063 | 0.133055981 | 7.152843e-01 | 8.946198e-01 |
| *Amylin* | -24.50843445 | 0.876769277 | 3.490881e-01 | 8.642719e-01 |
| *Ins1* | 21.74344109 | 0.366496516 | 5.449202e-01 | 8.946198e-01 |
| *Ins2* | 24.34704226 | 0.681181699 | 4.091801e-01 | 8.946198e-01 |
| *Ir* | 0.11315939 | 0.385596379 | 5.346233e-01 | 8.946198e-01 |
| *Irs1* | -0.29108939 | 1.728891681 | 1.885525e-01 | 7.898493e-01 |
| *Irs2* | -0.05790319 | 0.098216644 | 7.539803e-01 | 8.946198e-01 |
| *Jak2* | 0.19207687 | 1.164798571 | 2.804726e-01 | 8.614515e-01 |
| *Lepr* | 0.57053851 | 8.993203851 | 2.709855e-03 | 5.826188e-02 |
| *Lepr-b* | 0.16622298 | 0.193109608 | 6.603410e-01 | 8.946198e-01 |
| *Mc4r* | 0.25578267 | 1.516941996 | 2.180828e-01 | 7.898493e-01 |
| *Mch* | 0.19378337 | 0.419022722 | 5.174251e-01 | 8.946198e-01 |
| *Npy* | 0.08381308 | 0.191103804 | 6.619995e-01 | 8.946198e-01 |
| *Ptp1b* | 0.02677086 | 0.022224578 | 8.814912e-01 | 9.244908e-01 |
| *Pde3b* | 0.21401545 | 1.372291707 | 2.414185e-01 | 7.985382e-01 |
| *Pdk1* | 0.32506185 | 3.407353703 | 6.490646e-02 | 6.160181e-01 |
| *Pias3* | 0.06531130 | 0.124401287 | 7.243091e-01 | 8.946198e-01 |
| *Pik3ca* | 0.24427355 | 1.794406385 | 1.803902e-01 | 7.898493e-01 |
| *Pik3r1* | -0.03346406 | 0.034558160 | 8.525246e-01 | 9.164639e-01 |
| *Pomc* | 0.32862506 | 0.561594552 | 4.536185e-01 | 8.946198e-01 |
| *Ramp1* | -0.14033037 | 1.501614751 | 2.204231e-01 | 7.898493e-01 |
| *Ramp2* | 0.44424005 | 6.220410250 | 1.262858e-02 | 1.810097e-01 |
| *Ramp3* | 0.08043291 | 0.172273519 | 6.780991e-01 | 8.946198e-01 |
| *Socs3* | -0.09649301 | 0.062967762 | 8.018653e-01 | 8.961567e-01 |
| *Stat3* | 0.32001841 | 3.245267180 | 7.163001e-02 | 6.160181e-01 |
| *c-fos* | -1.55853442 | 43.462522953 | 4.321576e-11 | 1.858278e-09 |
| *mTor* | 0.17821270 | 1.001141449 | 3.170345e-01 | 8.642719e-01 |
