# Supplementary material for: Altered metabolic gene expression in the brain of a triprolyl-human amylin transgenic mouse model of type 2 diabetes
Source: Sci Rep. 2019 Oct 10;9:14588. doi: 10.1038/s41598-019-51088-x (PMC6787337; doi:10.1038/s41598-019-51088-x)
Supplement: Supplementary file 2 — Supplementary File S2 [file 41598_2019_51088_MOESM2_ESM.docx]

# Supplementary File S2. Antibody Characterisation, Full Western Blot Membrane Images and Data

**Article title:** Altered metabolic gene expression in the brain of a triprolyl-human amylin transgenic mouse model of type 2 diabetes

**Journal:** Scientific Reports

**Authors:** Tina Nie, Shaoping Zhang, Greeshma Vazhoor Amarsingh, Hong Liu, Mark J. McCann, Garth J.S. Cooper

**Corresponding author:** Garth J.S. Cooper (School of Biological Sciences, Faculty of Science, the University of Auckland; The Maurice Wilkins Centre for Molecular Biodiscovery, Faculty of Science, the University of Auckland; Centre for Advanced Discovery and Experimental Therapeutics, Faculty of Biology, Medicine & Health, School of Medical Sciences, Division of Cardiovascular Sciences, the University of Manchester), [g.cooper@auckland.ac.nz](mailto:g.cooper@auckland.ac.nz)

## Socs3

### Characterisation

The anti-Socs3 antibody (ab16030, Abcam) was first trialled, using lysates from L44 mouse brain samples, at a dilution of 1/500 (1 µg/ml) as recommended in the manufacturer’s instructions. This gave a positive result with visible bands at ~25 kDa as expected. We trialled this antibody again at a dilution of 1/200 to see if this would improve the signal obtained, however we determined that the difference was marginal and continued with the 1/500 dilution.

We also tested the antibody with a blocking peptide (human SOCS3 peptide ab16199, Abcam). A mixture of 1/200 anti-Socs3 antibody with 1/100 blocking peptide was incubated at room temperature for 30 minutes prior to incubation with the membrane. The intensity of the protein bands at ~25 kDa faded with addition of the peptide, compared to the antibody alone, confirming antibody specificity.

Membranes routinely showed one or two bands staining at ~150 kDa. In some cases, a third band greater than 250 kDa was also visible. These were too large to be monomeric Socs3 protein. The intensity of these larger bands also faded when the blocking peptide was added, indicating they may be multimers or complexes containing Socs3 which did not separate after addition of dithiothreitol or heating. We used only the ~25 kDa band for our analysis as we didn’t know the identity of the high-molecular weight bands.

### Full blot images

Full images of the Western blot membranes are presented below. A negative control (no primary antibody incubation) was run on the left of the ladder and a calibrator sample was run on each gel.


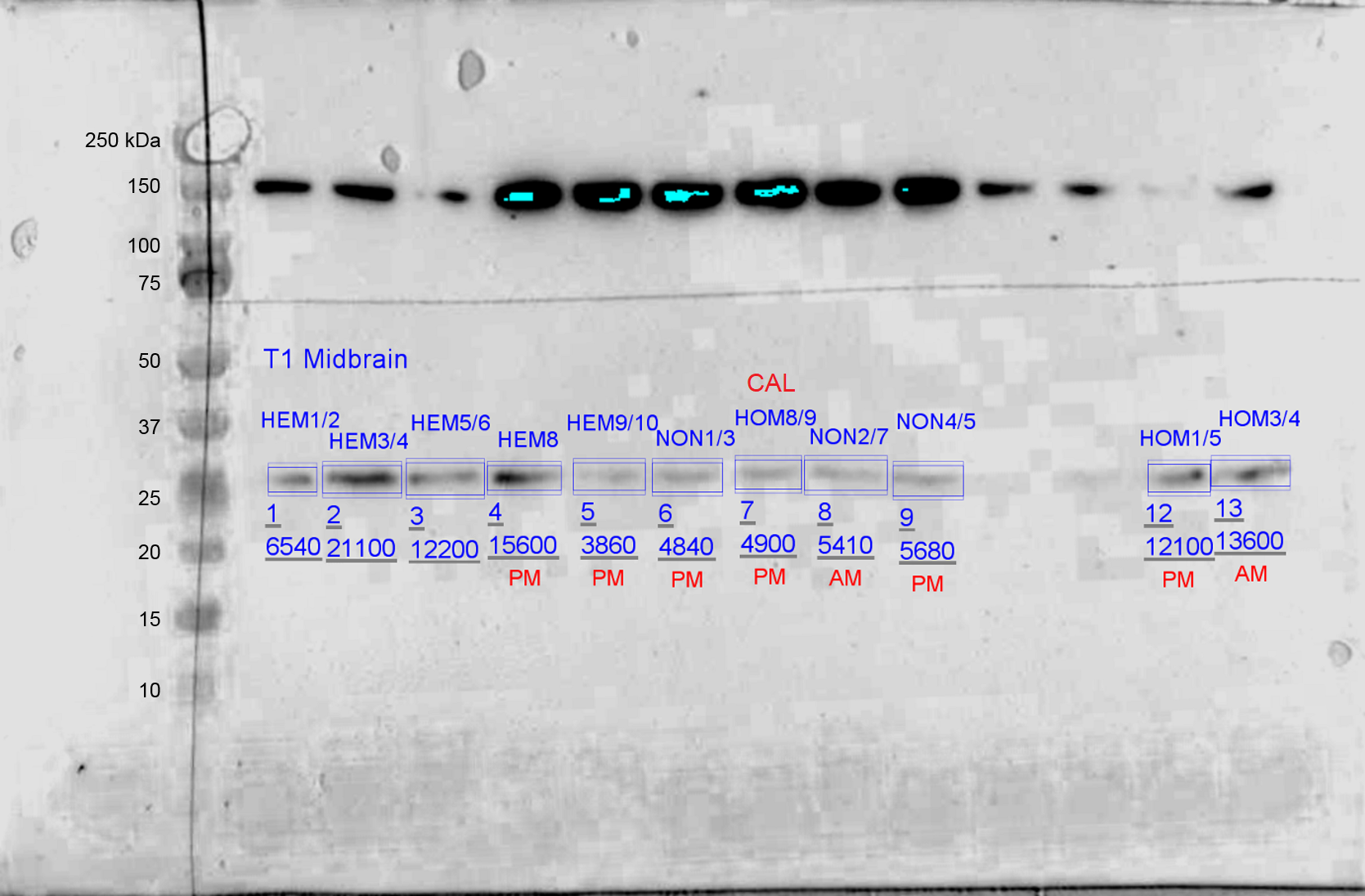


**Figure 1** Socs3 intensity of membrane 1. Exposure time: 2.15.8 minutes. Annotation (top to bottom): sample ID, lane number on gel, band intensity (before calibration) and collection time. CAL = calibrator sample. All following membrane images are presented in this way


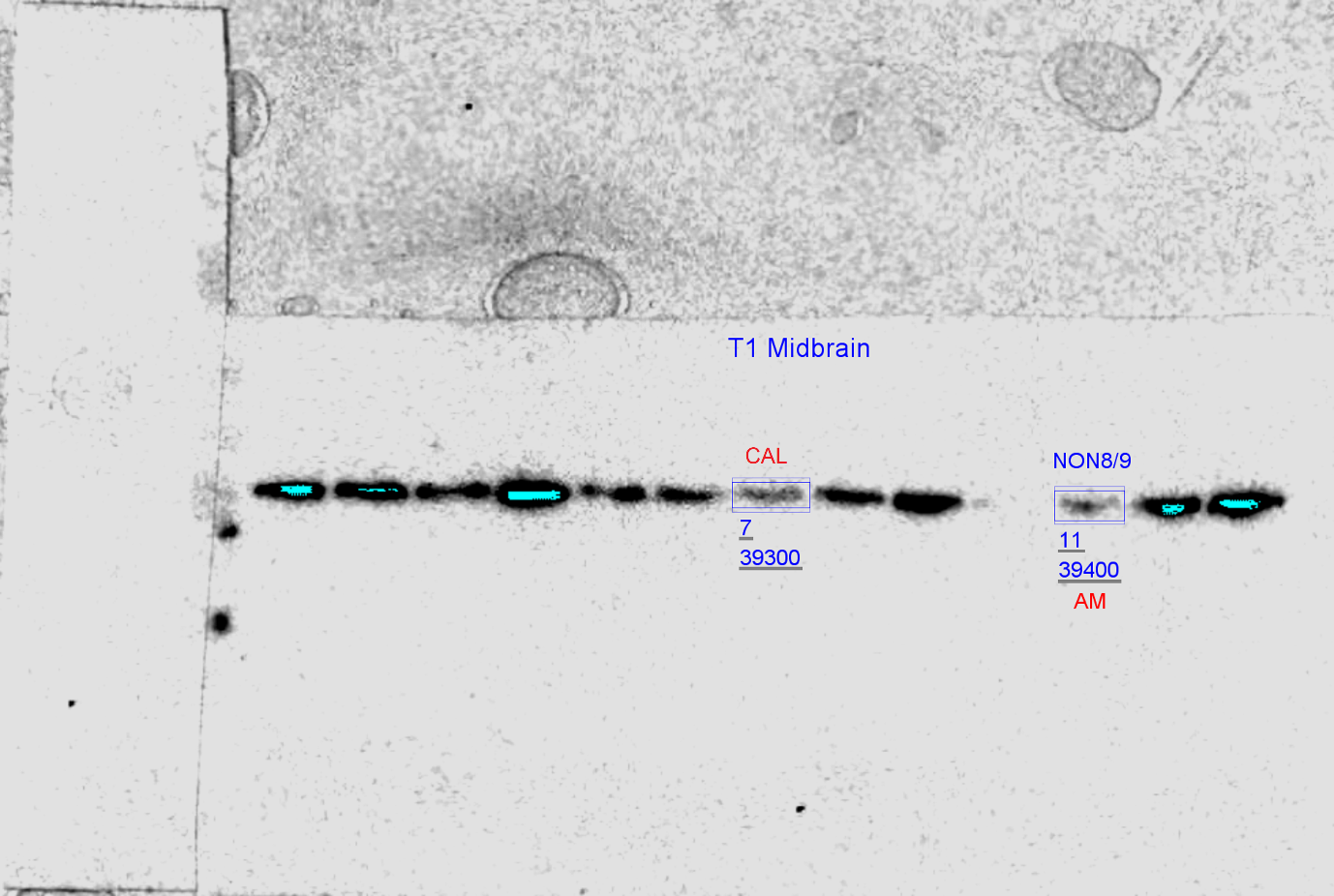


**Figure 2** Socs3 intensity of membrane 1 (second exposure). Exposure time: 60.59.9 minutes. Part of this blot has been cut and removed to avoid oversaturation from the upper bands as seen in the first exposure (Fig 1)


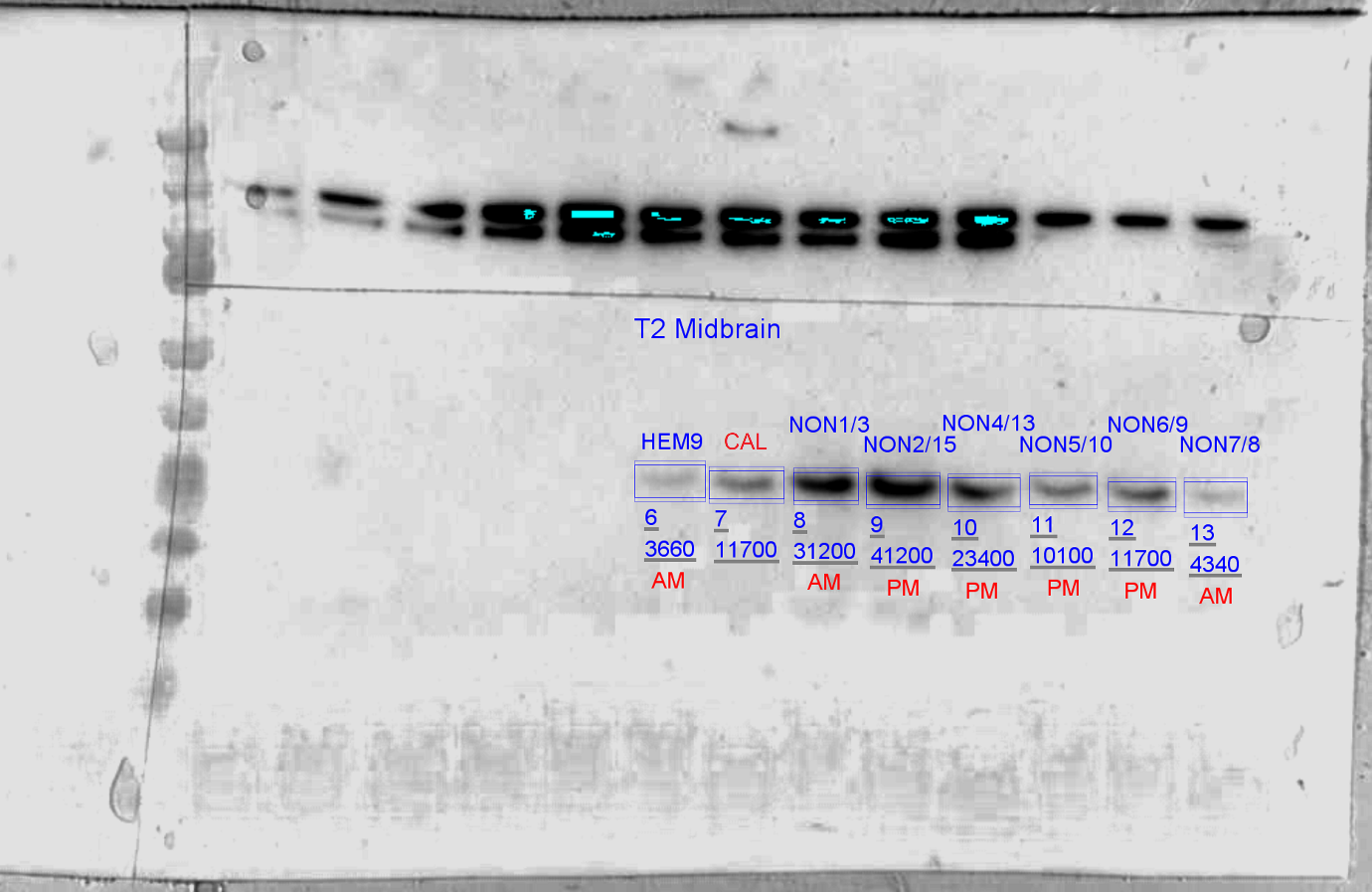


**Figure 3** Socs3 intensity of membrane 2. Exposure time: 9.28.4 minutes


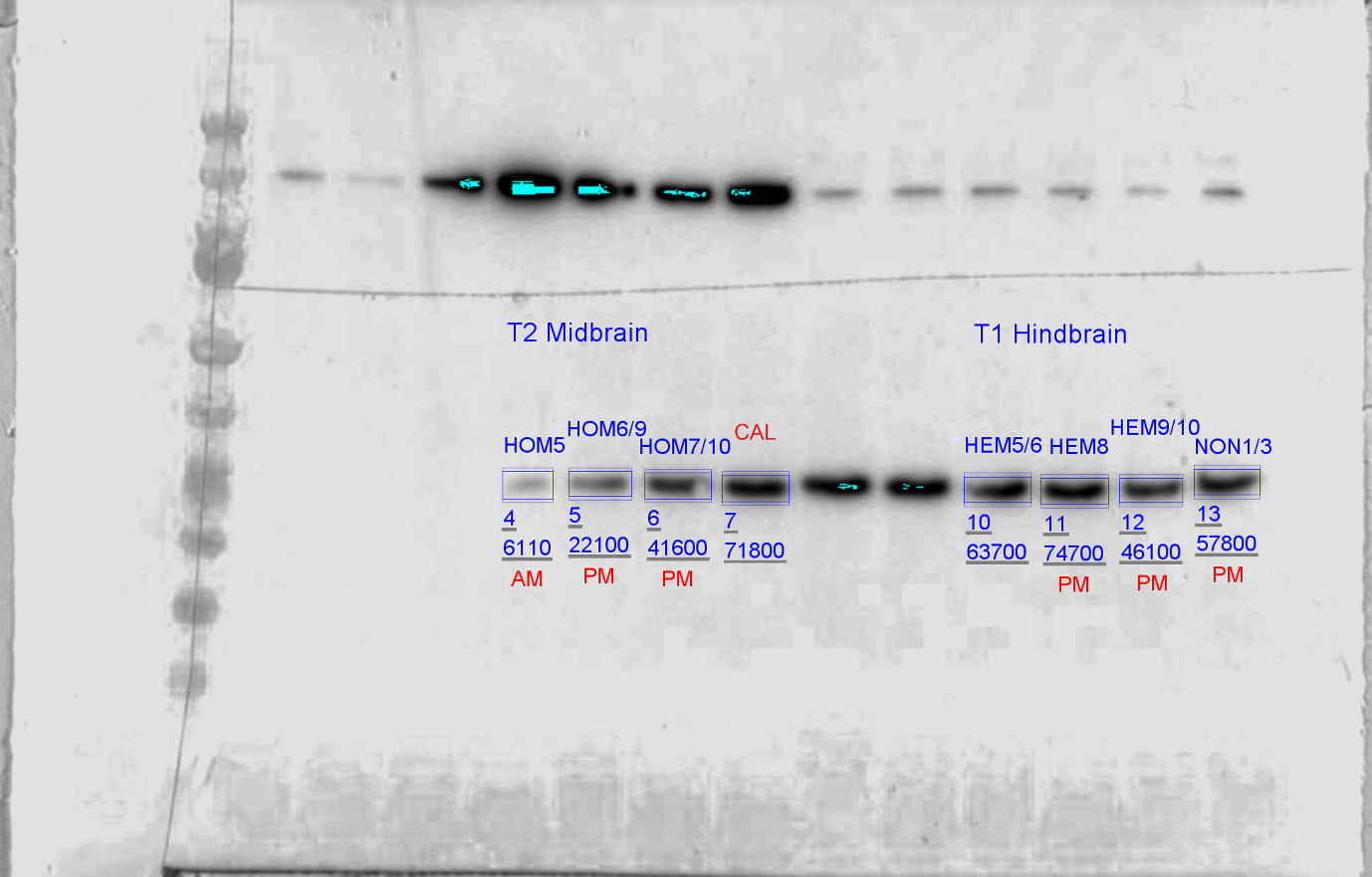


**Figure 4** Socs3 intensity of membrane 3. Exposure time: 1.11.2 minutes


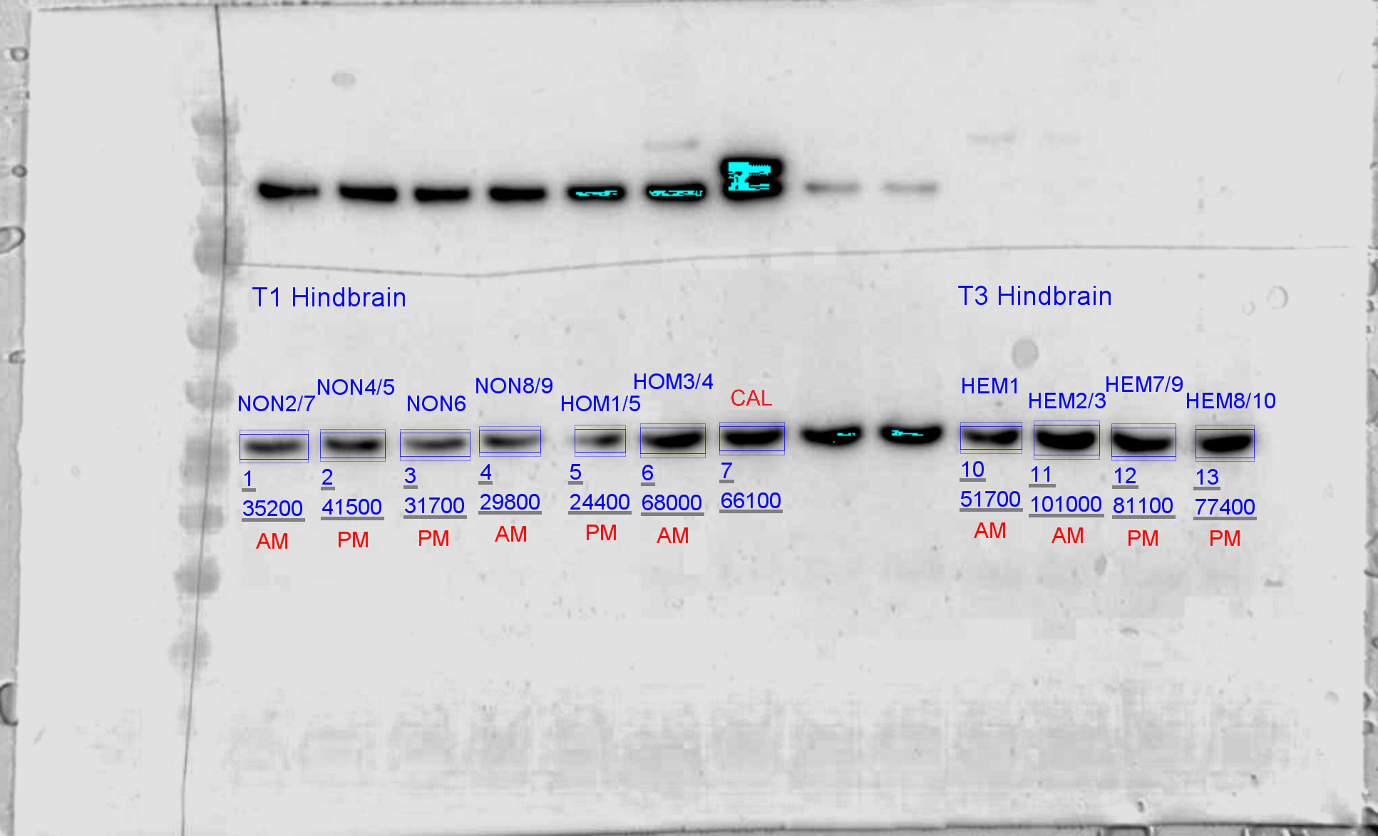


**Figure 5** Socs3 intensity of membrane 4. Exposure time: 7.47.8 minutes


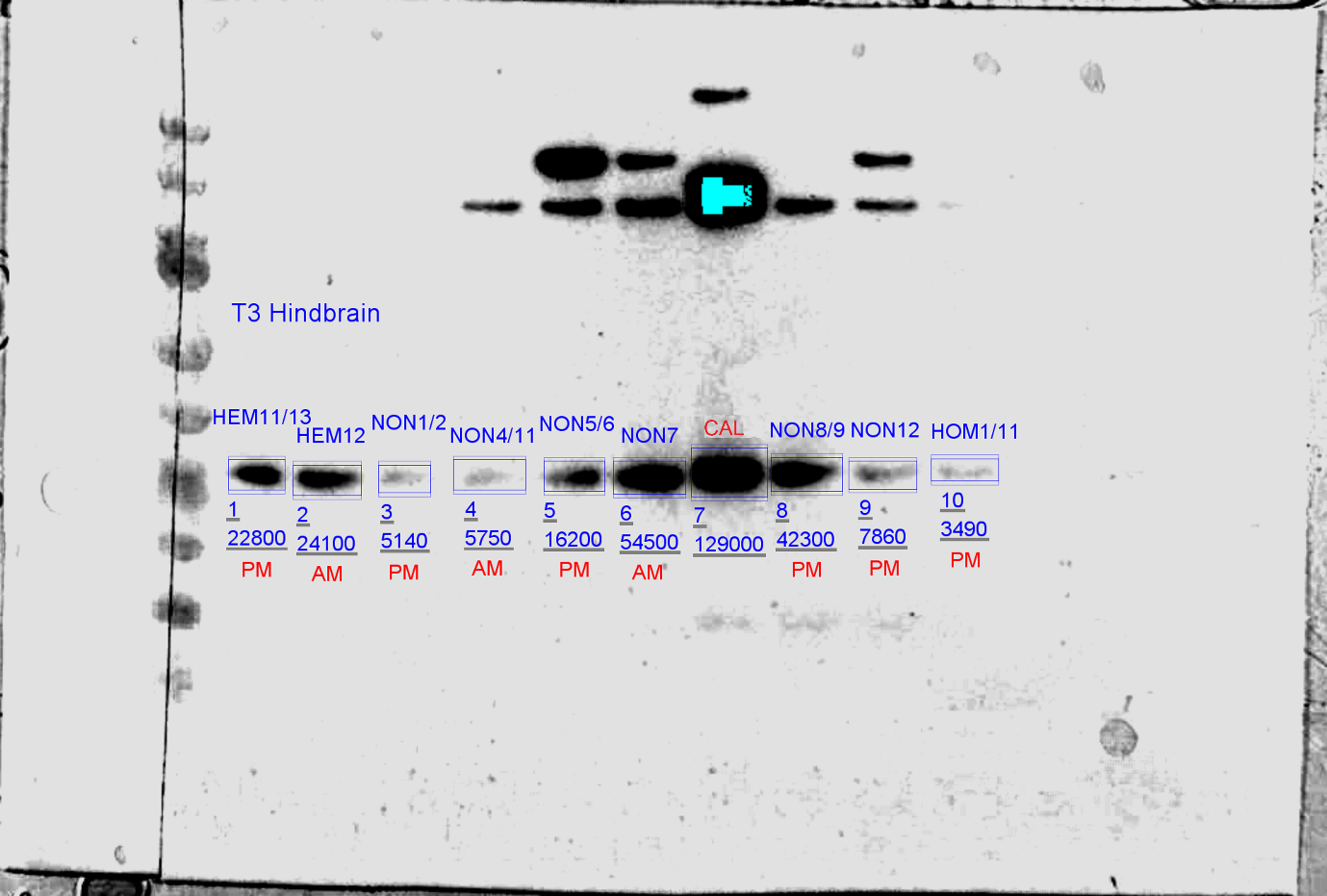


**Figure 6** Socs3 intensity of membrane 5. Exposure time: 13.25.5 minutes


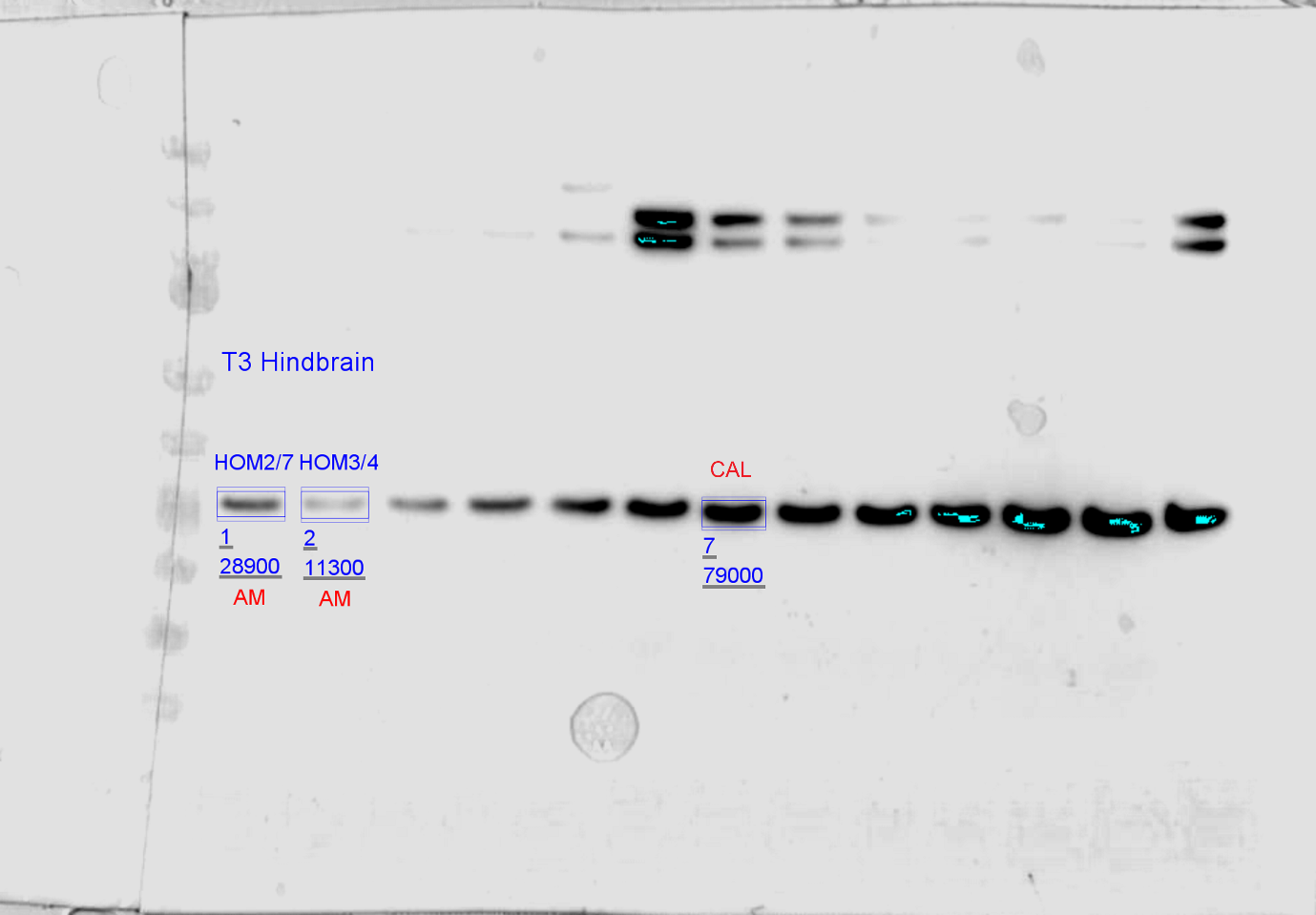


**Figure 7** Socs3 intensity of membrane 6. Exposure time: 3.30.9 minutes


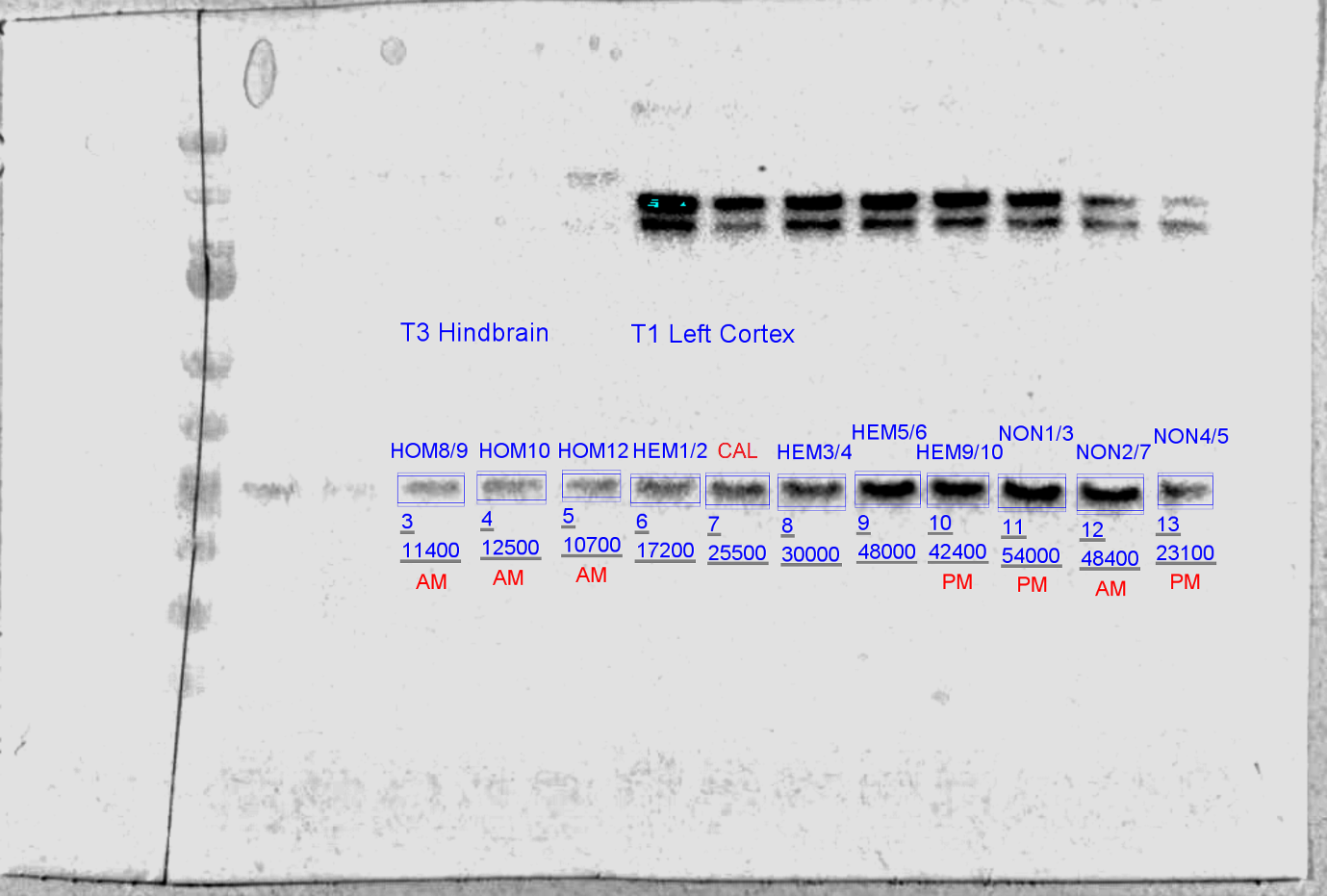


**Figure 8** SocsS3 intensity of membrane 6 (second exposure). Exposure time: 1 second


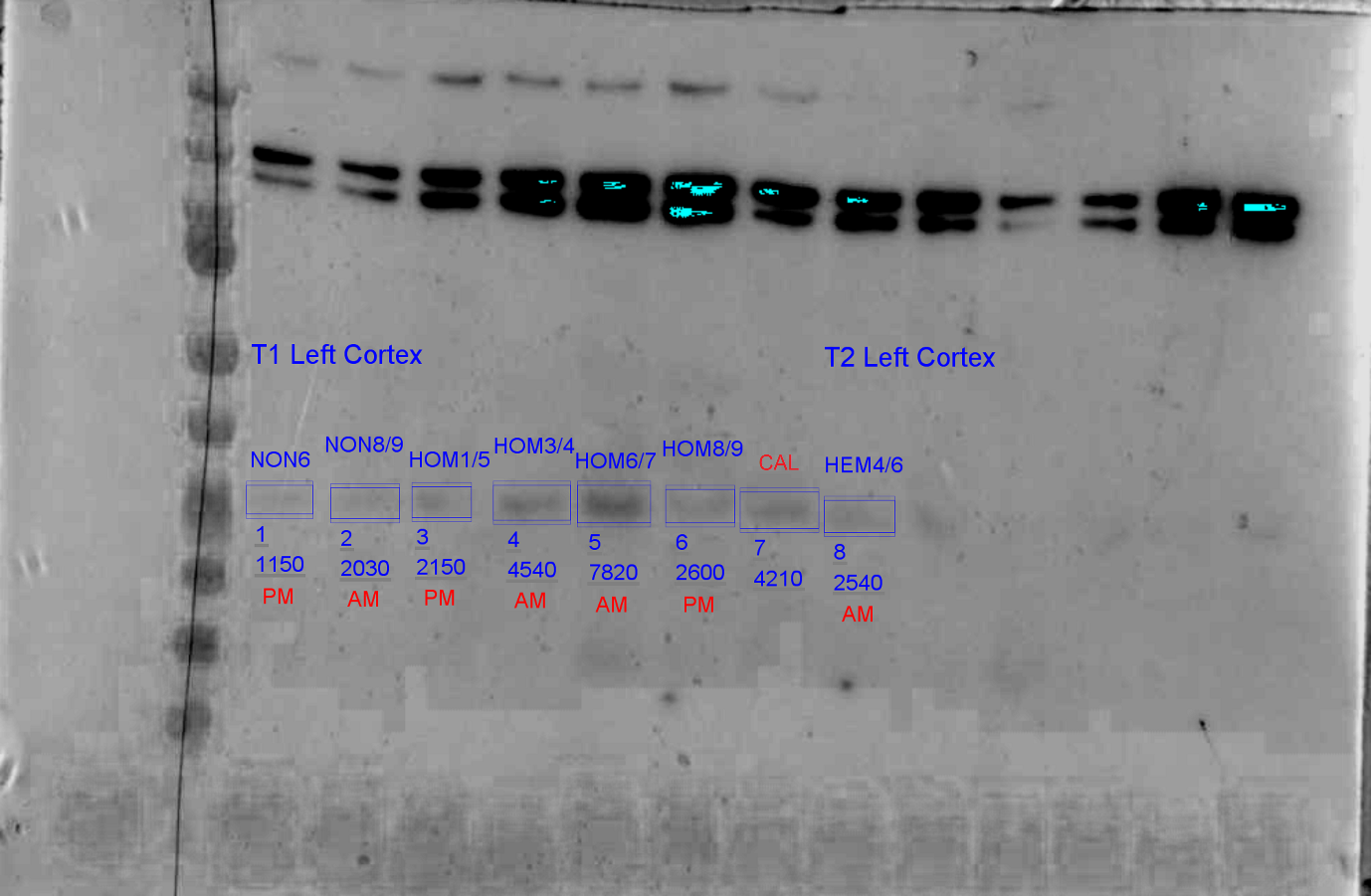


**Figure 9** Socs3 intensity of membrane 7. Exposure time: 7.57.3 minutes


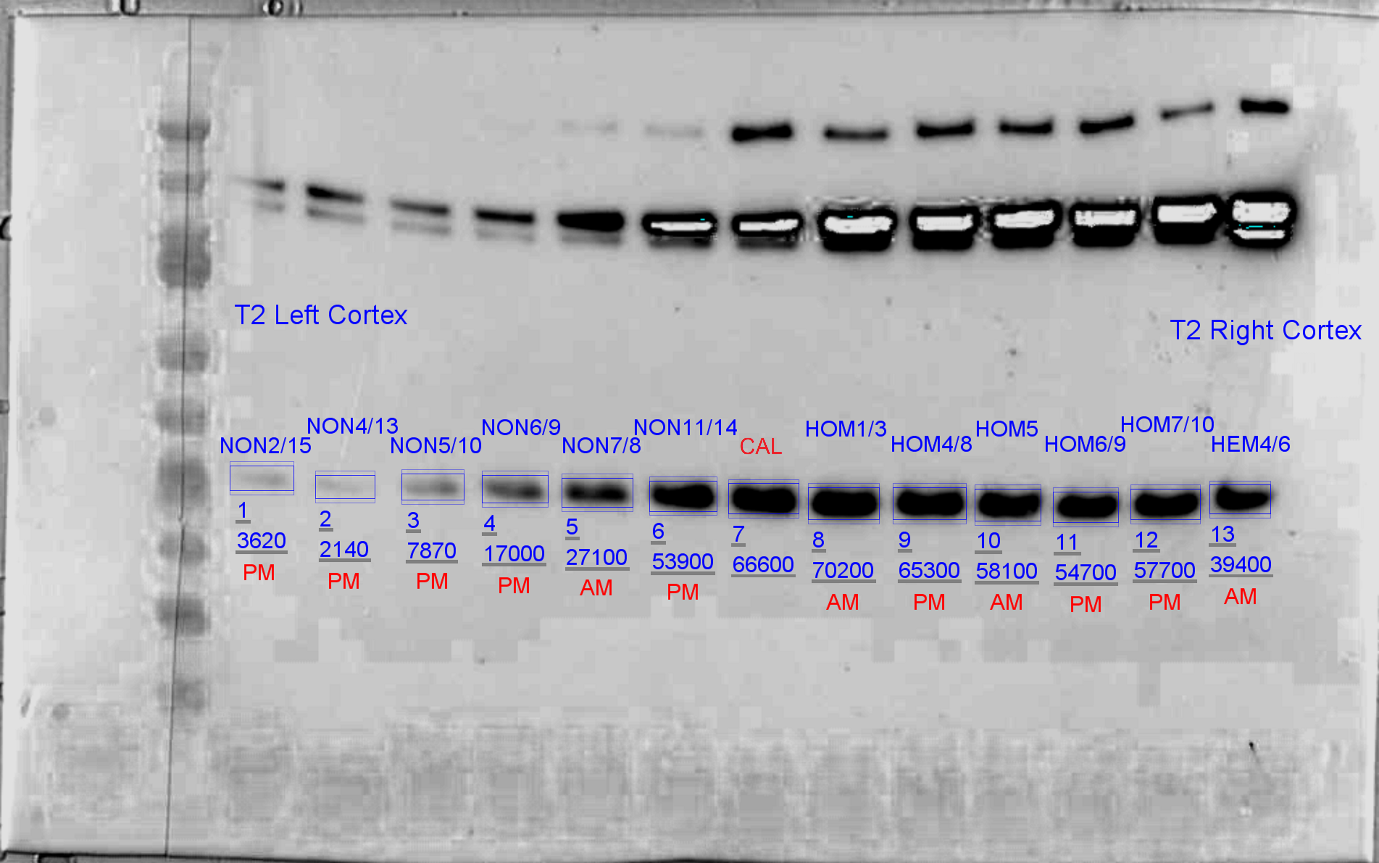


**Figure 10** Socs3 intensity of membrane 8. Exposure time: 10.42.8 minutes


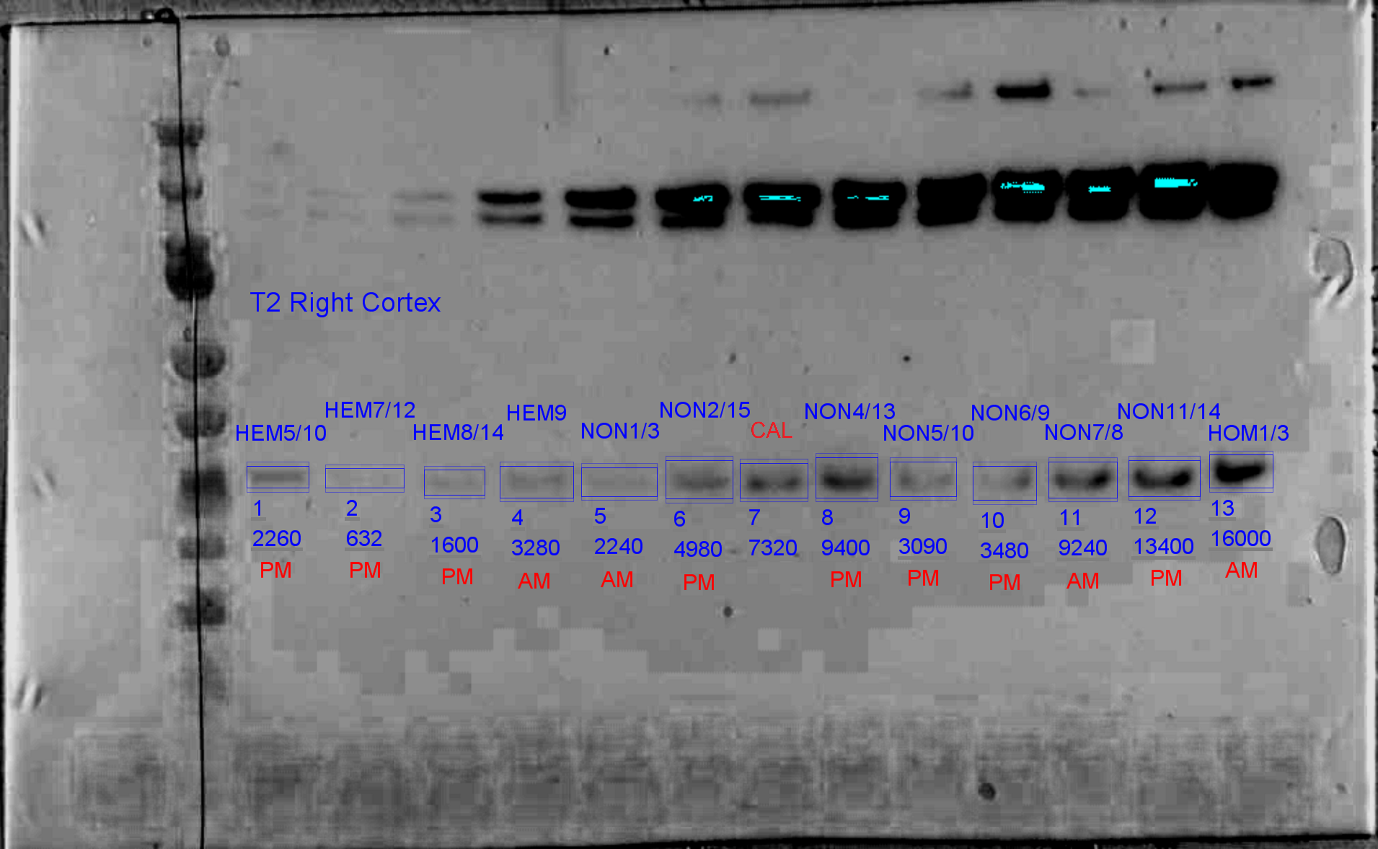


**Figure 11** Socs3 intensity of membrane 9. Exposure time: 1.40.8 minutes


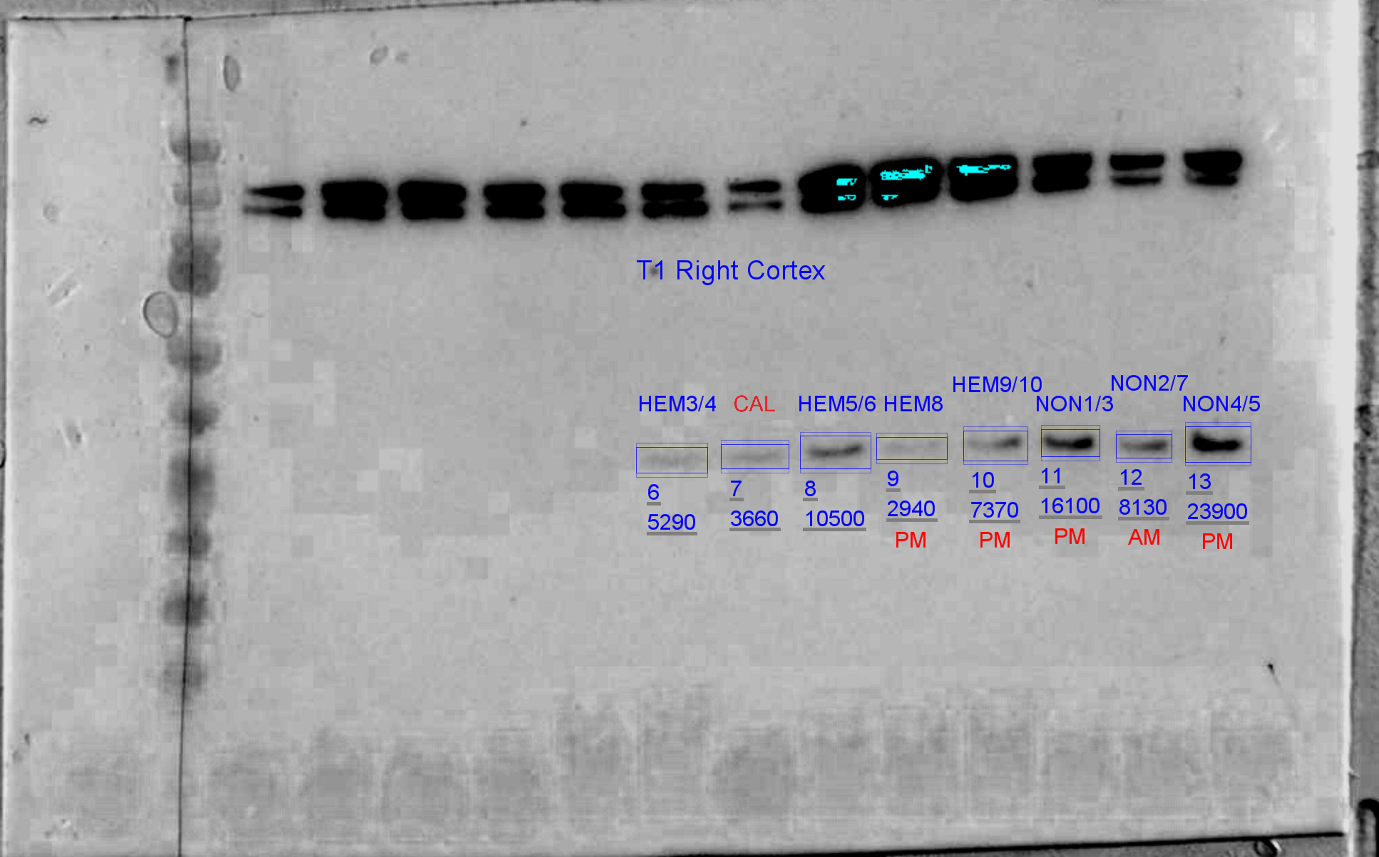


**Figure 12** Socs3 intensity of membrane 10. Exposure time: 6.19 minutes


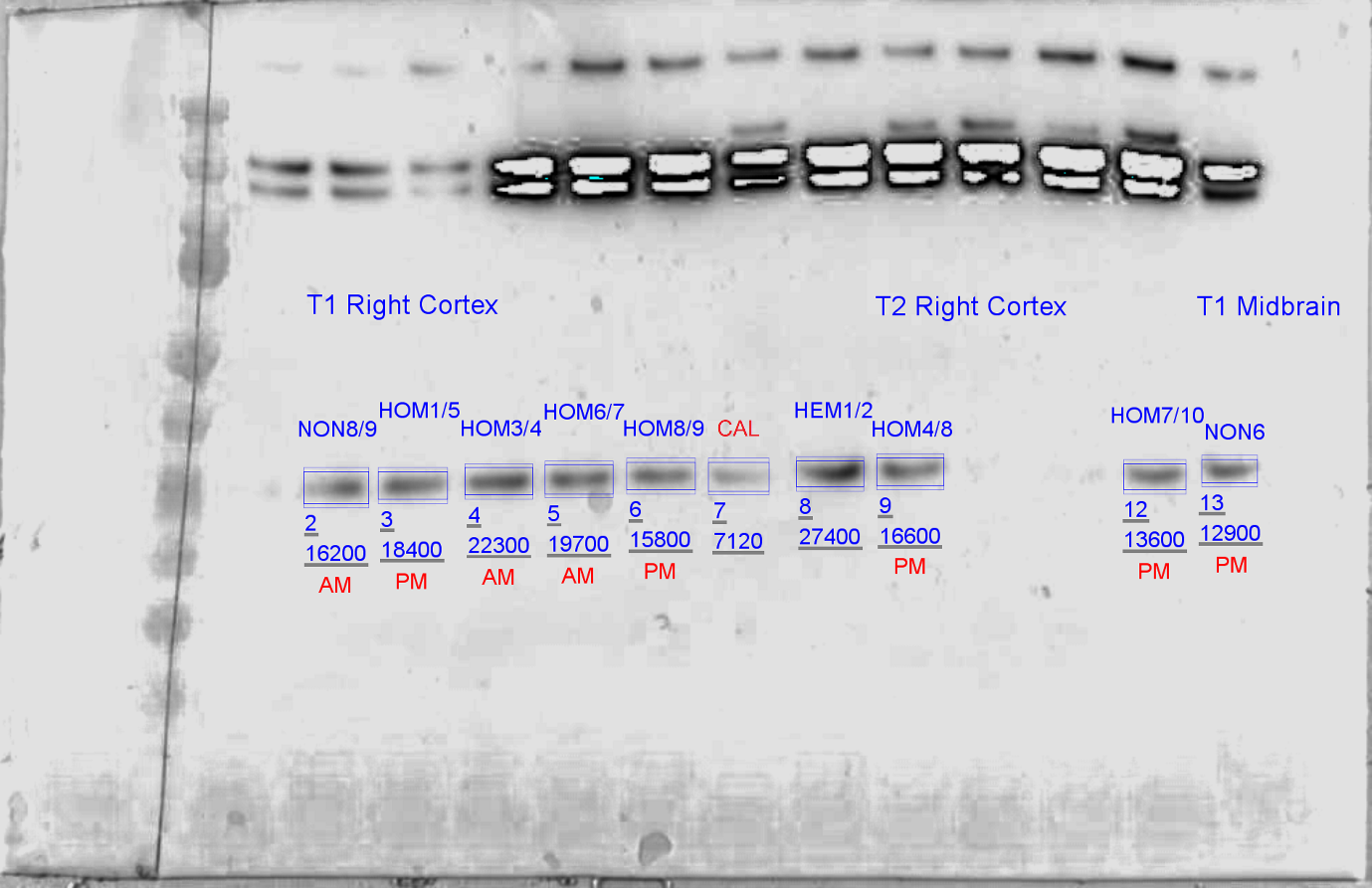


**Figure 13** Socs3 intensity of membrane 11. Exposure time: 20 minutes


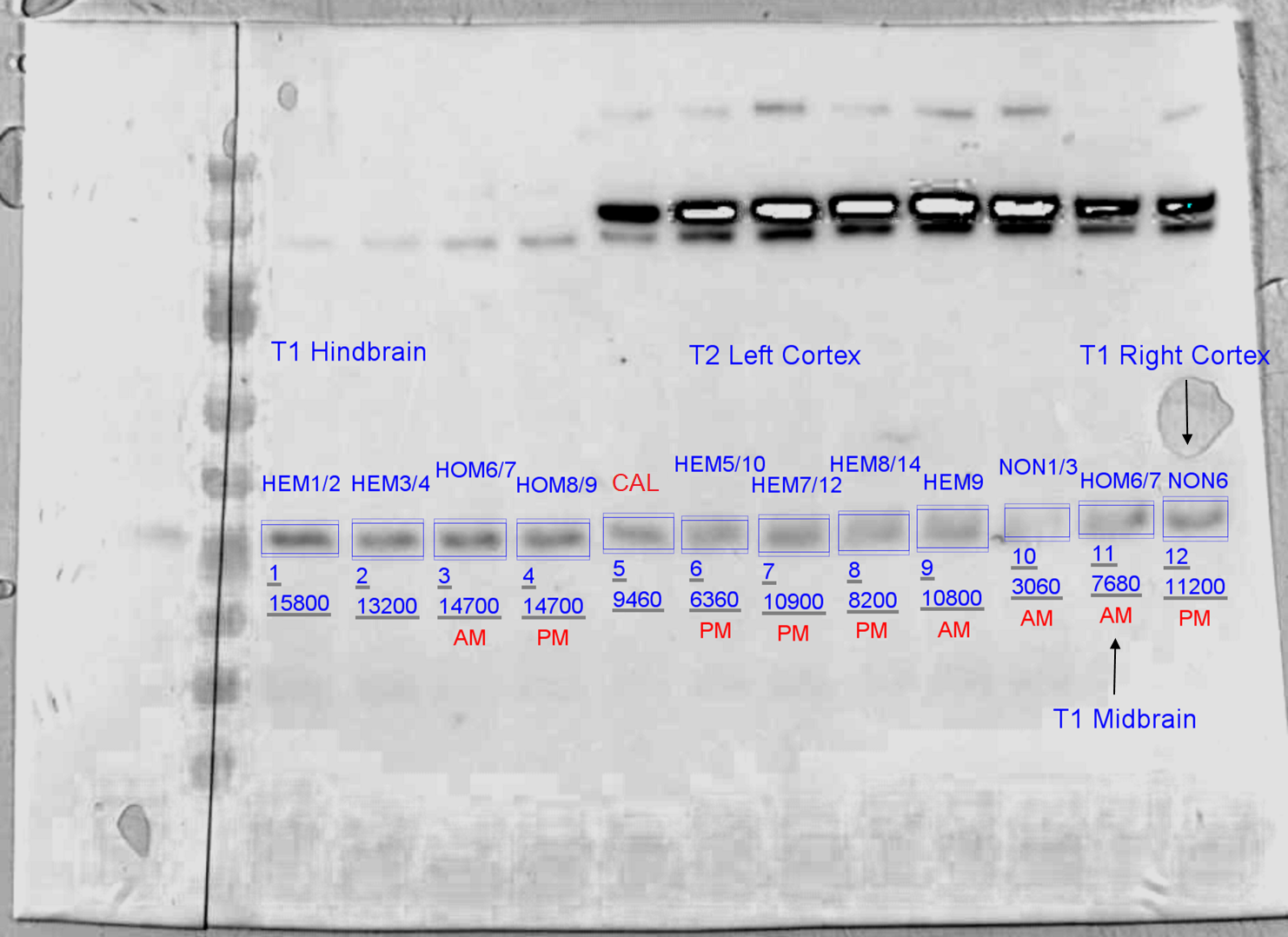


**Figure 14** Socs3 intensity of membrane 12. Exposure time: 5 minutes

On membrane 12 (Fig 14), a faint band at ~25 kDa can be seen to the left of the ladder where the negative control was loaded. However, this secondary antibody has not shown any non-specific binding on the negative control on any of the previous membranes. Thus, we decided these results would be acceptable to use.


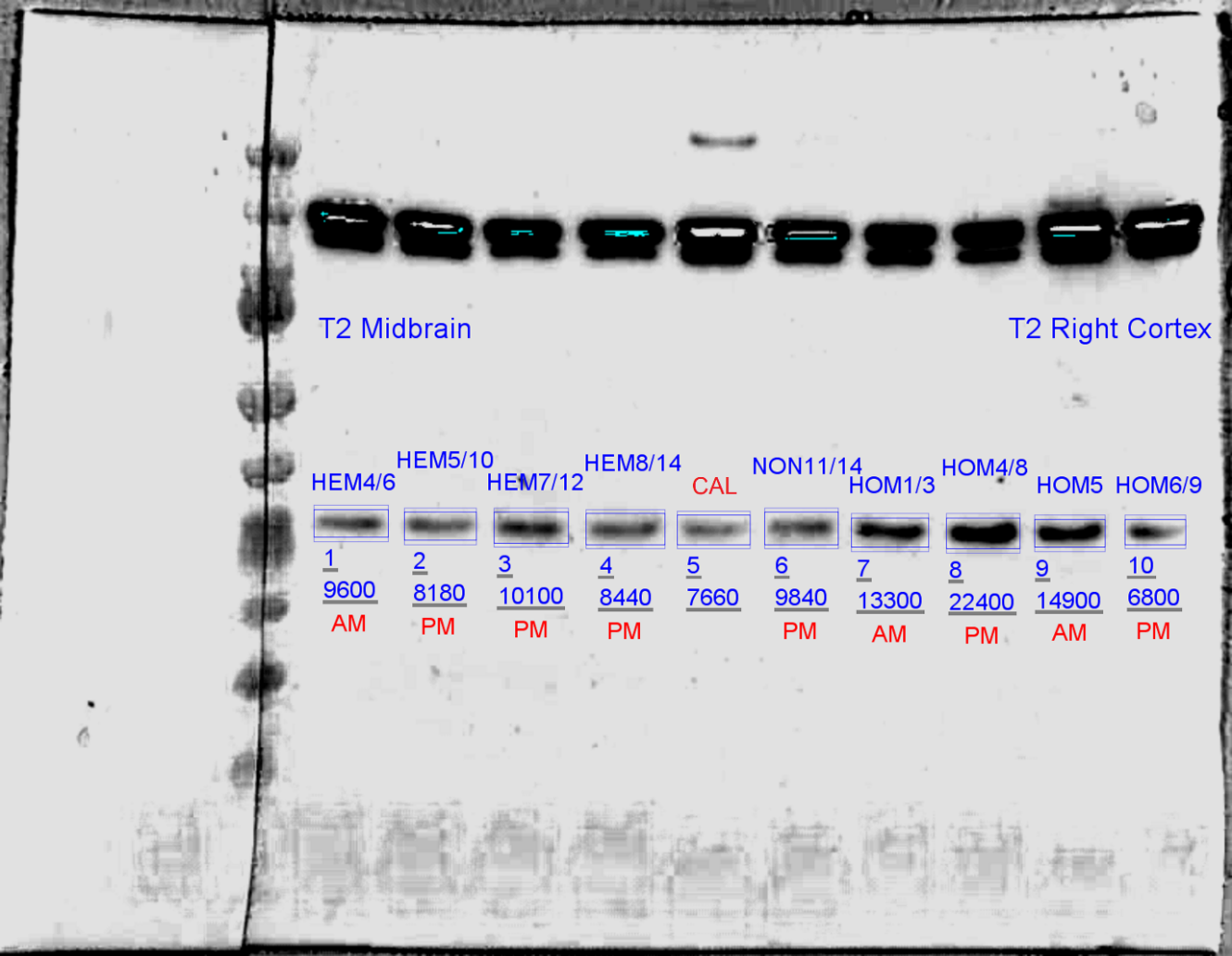


**Figure 15** Socs3 intensity of membrane 13. Exposure time: 5 minutes

### Ponceau S images

Densitometric analysis of a section of protein at ~25 kDa on the Ponceau S stained membrane was used for total protein normalisation. An unstained section of membrane representative of the background was used for background subtraction.


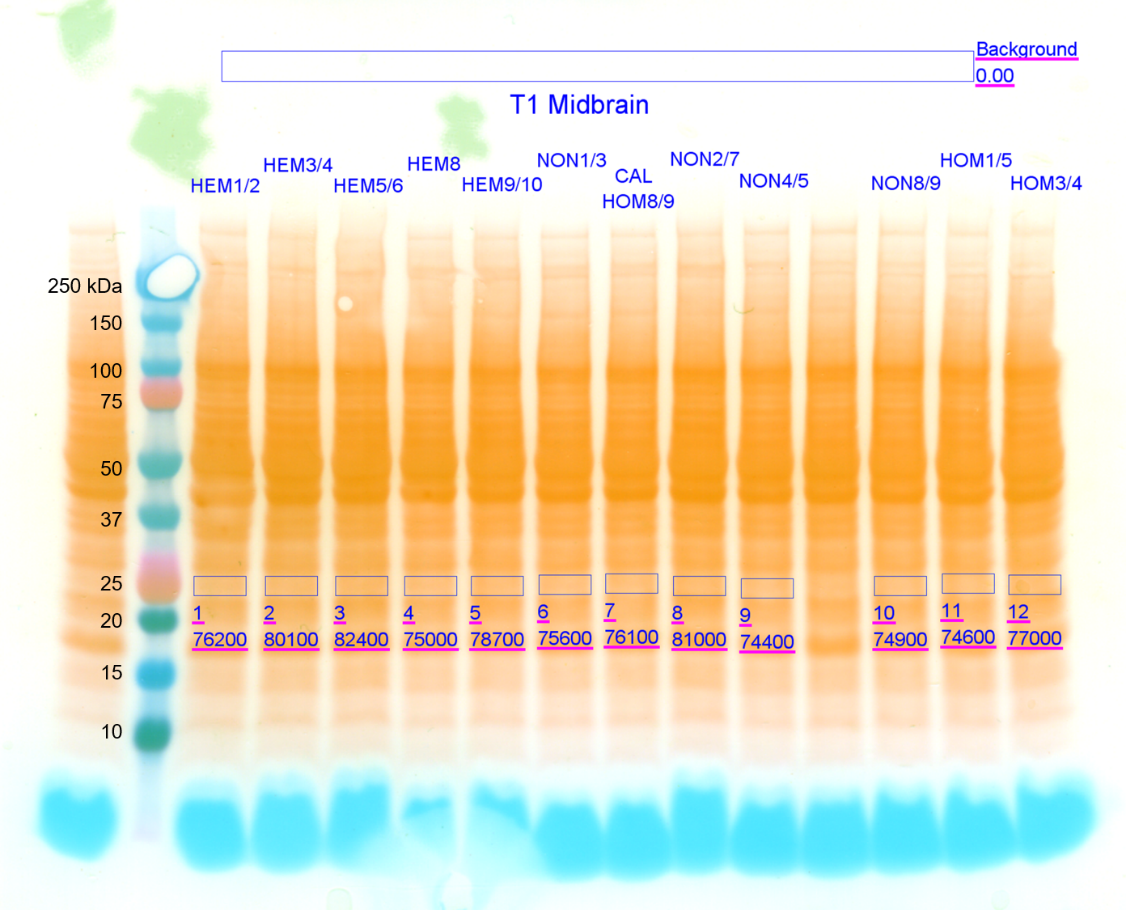


**Figure 16** Ponceau S Image of Socs3 Membrane 1


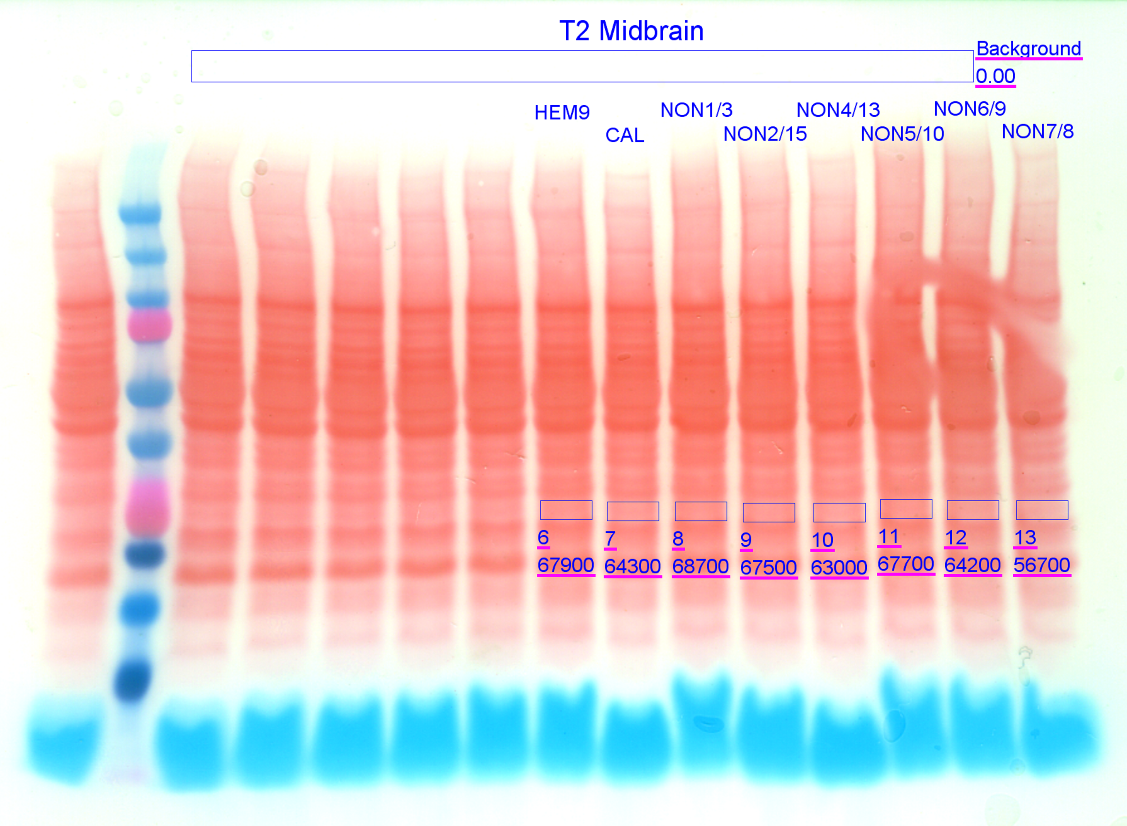


**Figure 17** Ponceau S Image of Socs3 Membrane 2


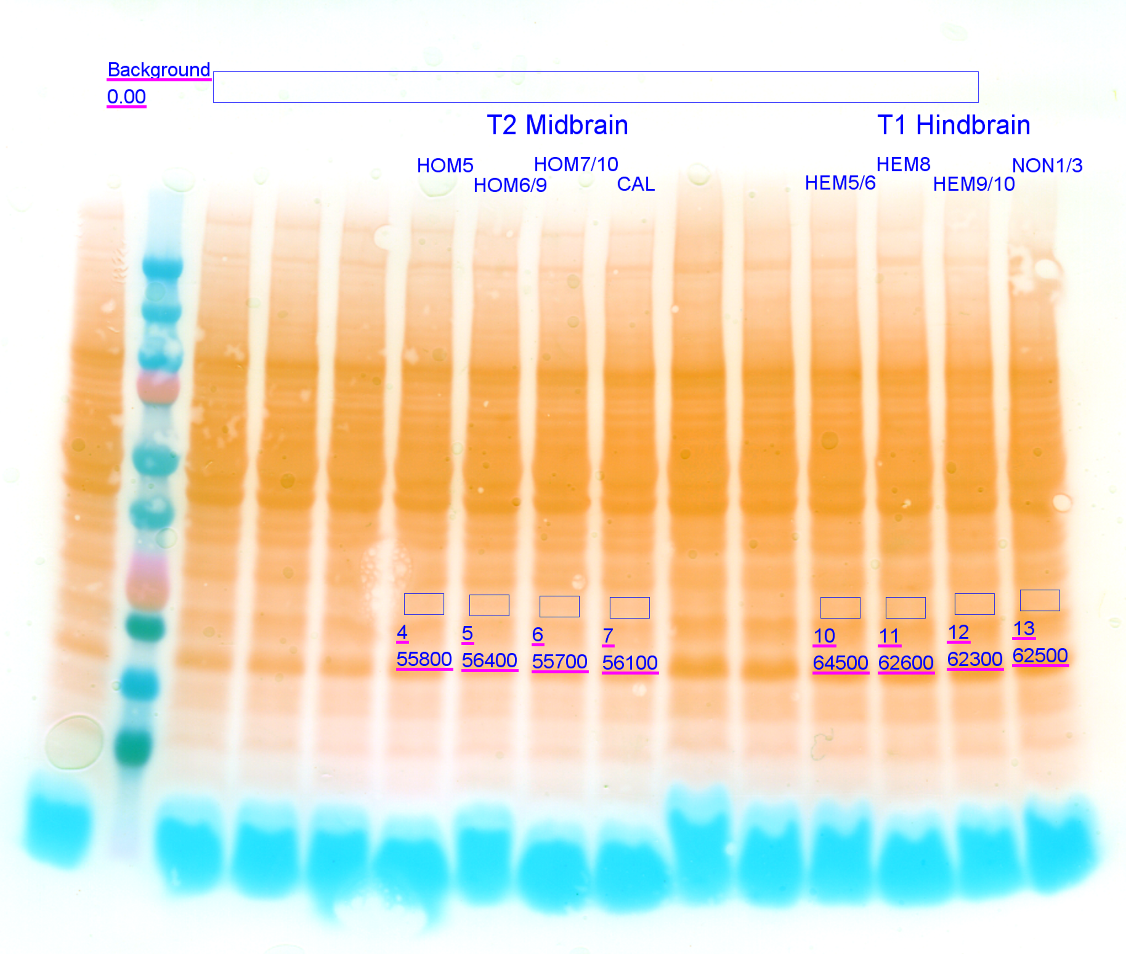


**Figure 18** Ponceau S Image of Socs3 Membrane 3


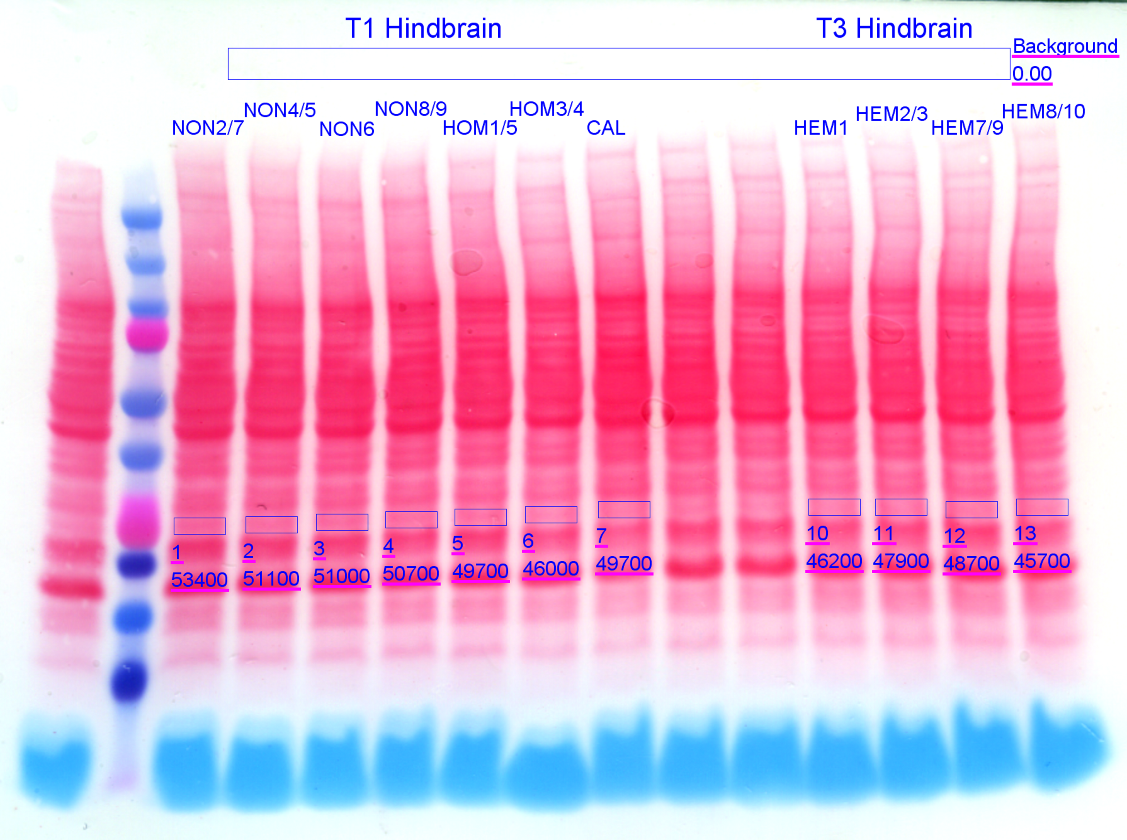


**Figure 19** Ponceau S Image of Socs3 Membrane 4


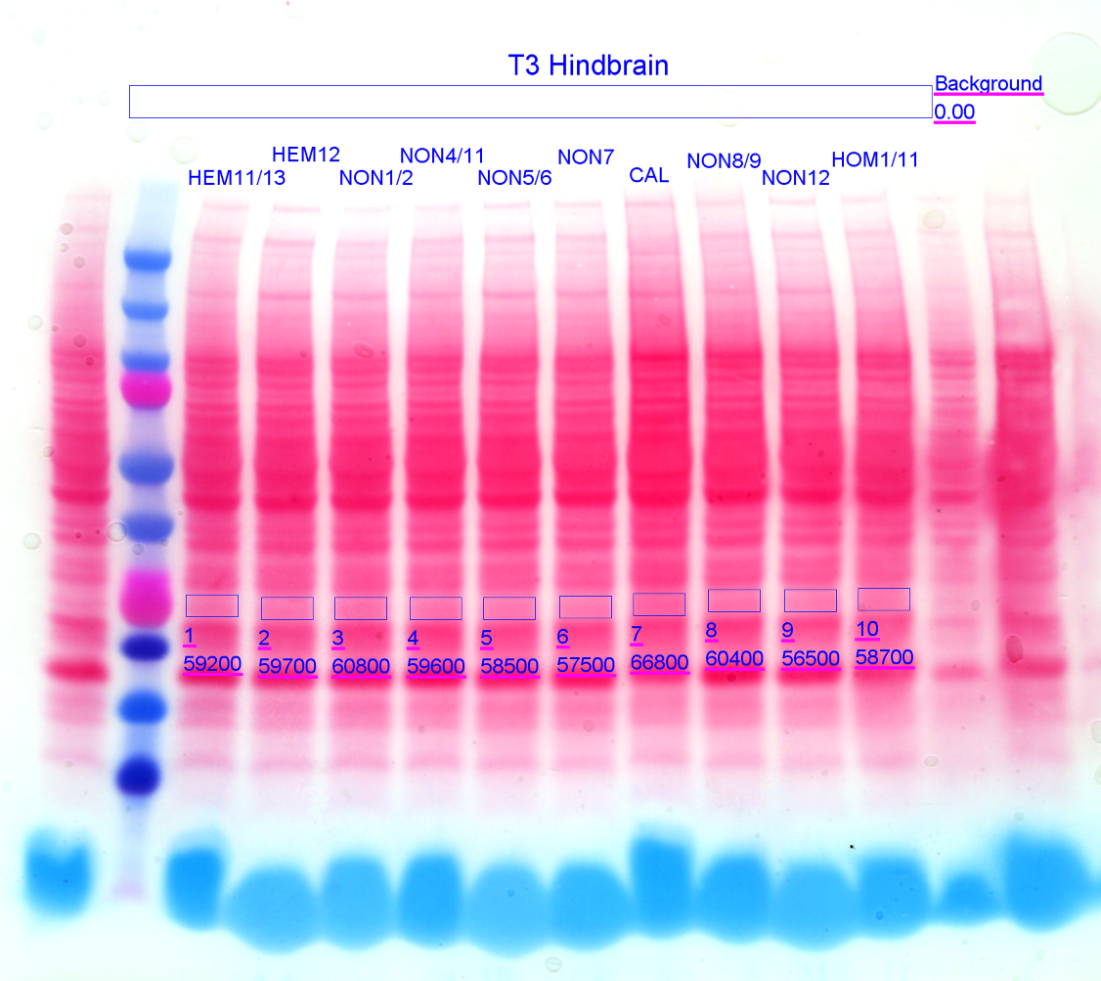


**Figure 20** Ponceau S Image of Socs3 Membrane 5


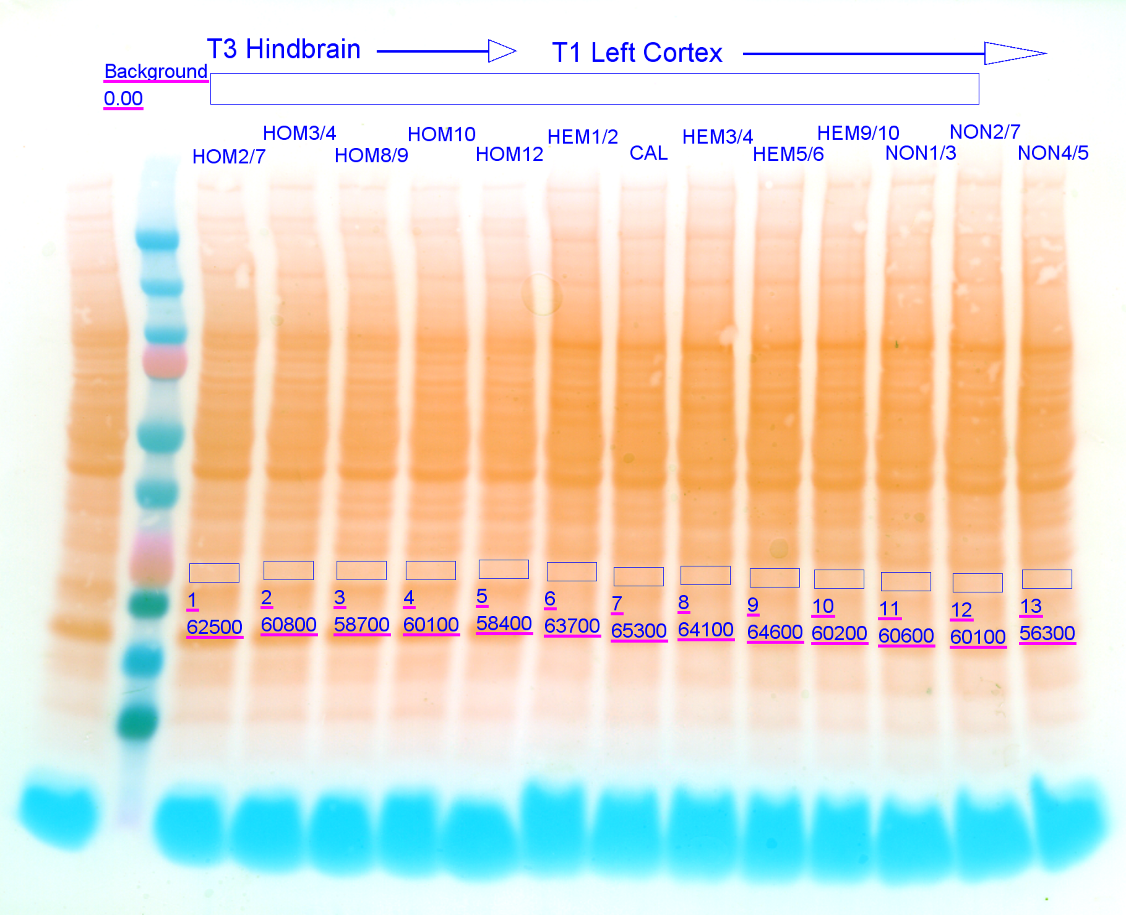


**Figure 21** Ponceau S Image of Socs3 Membrane 6


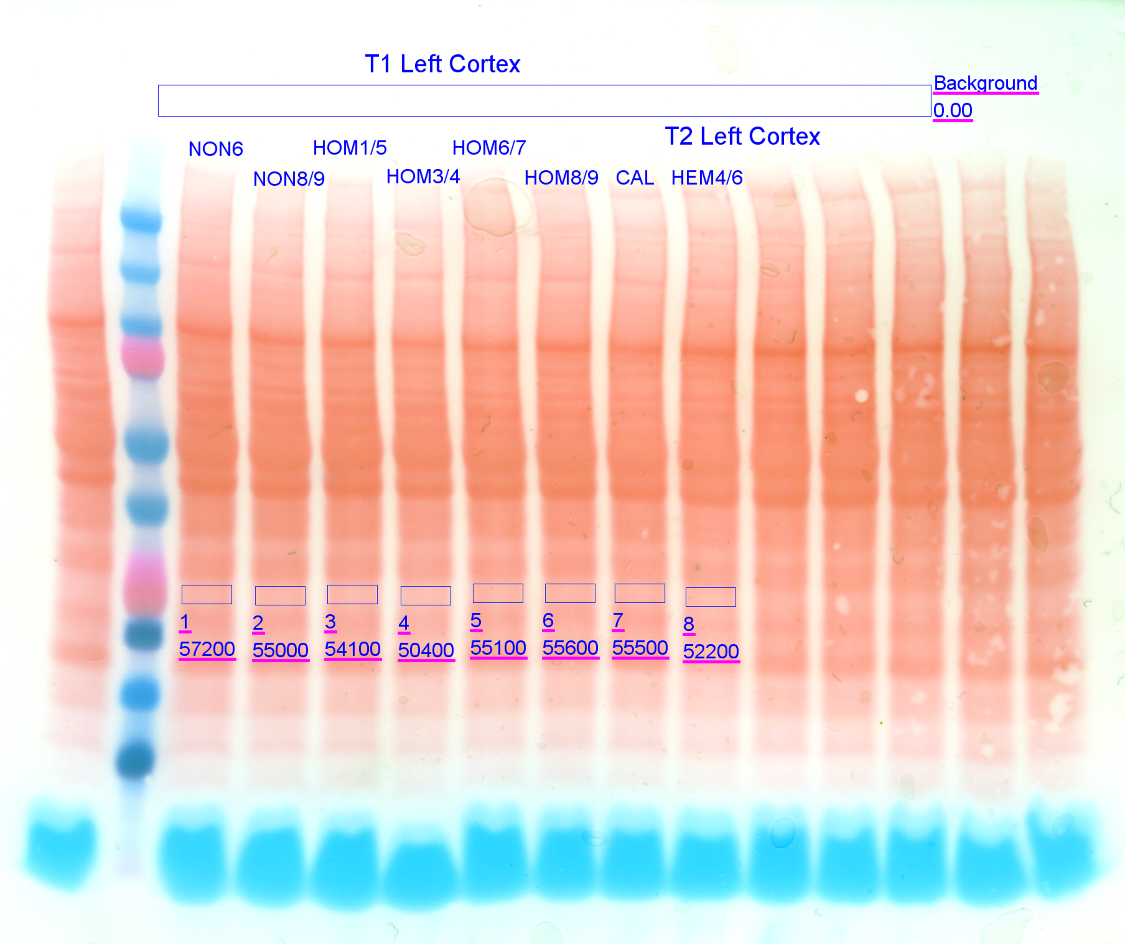


**Figure 22** Ponceau S Image of Socs3 Membrane 7


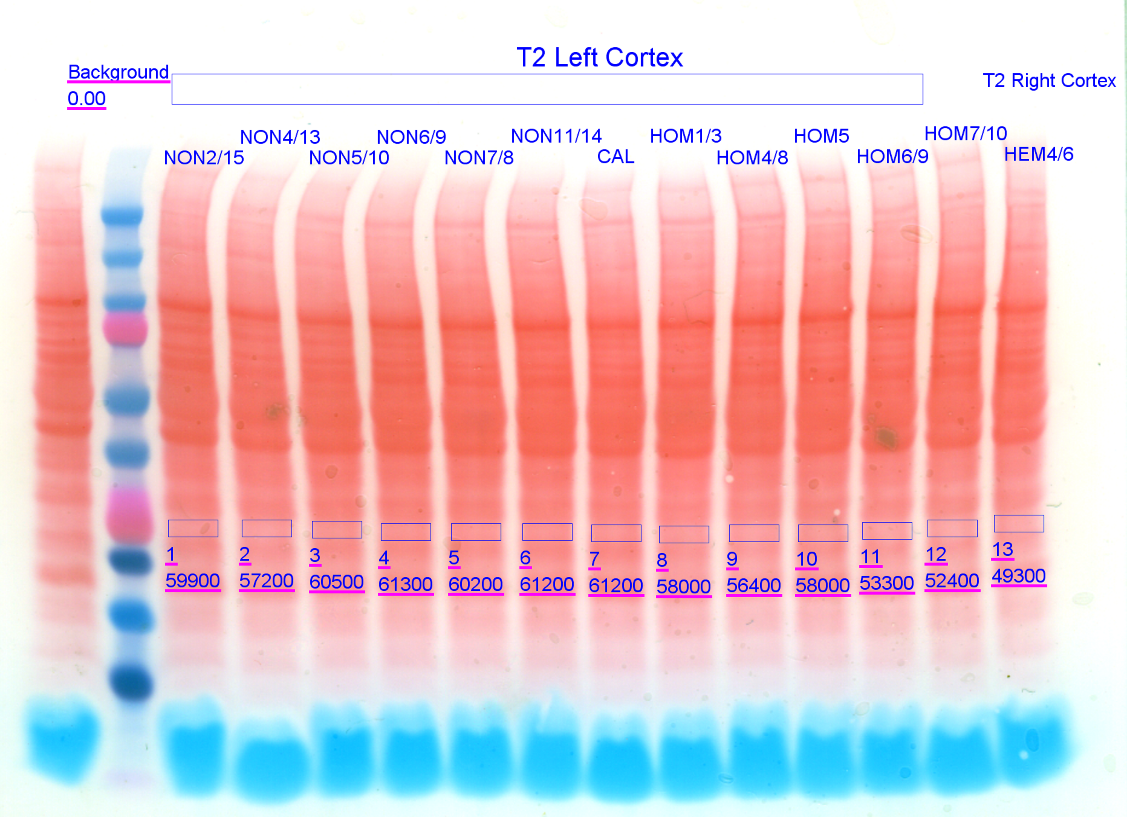


**Figure 23** Ponceau S Image of Socs3 Membrane 8


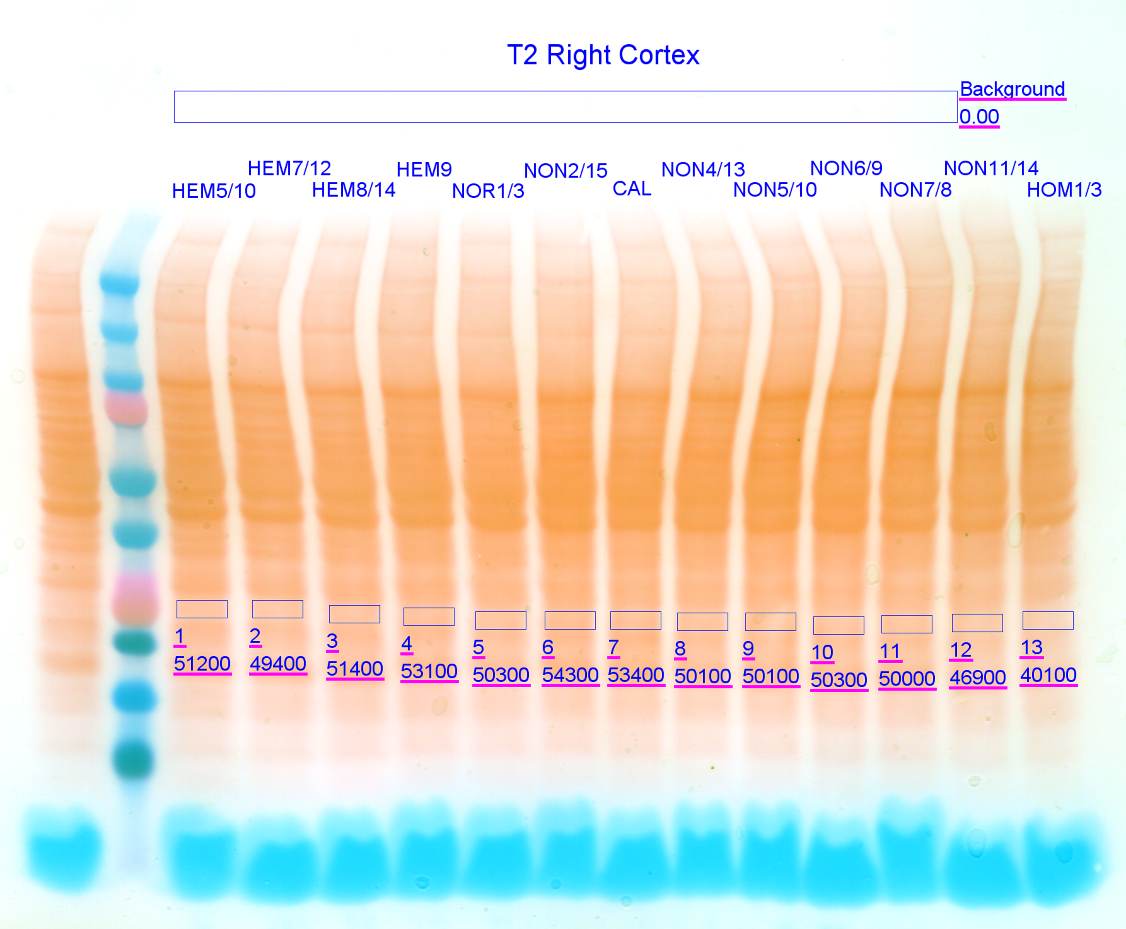


**Figure 24** Ponceau S Image of Socs3 Membrane 9


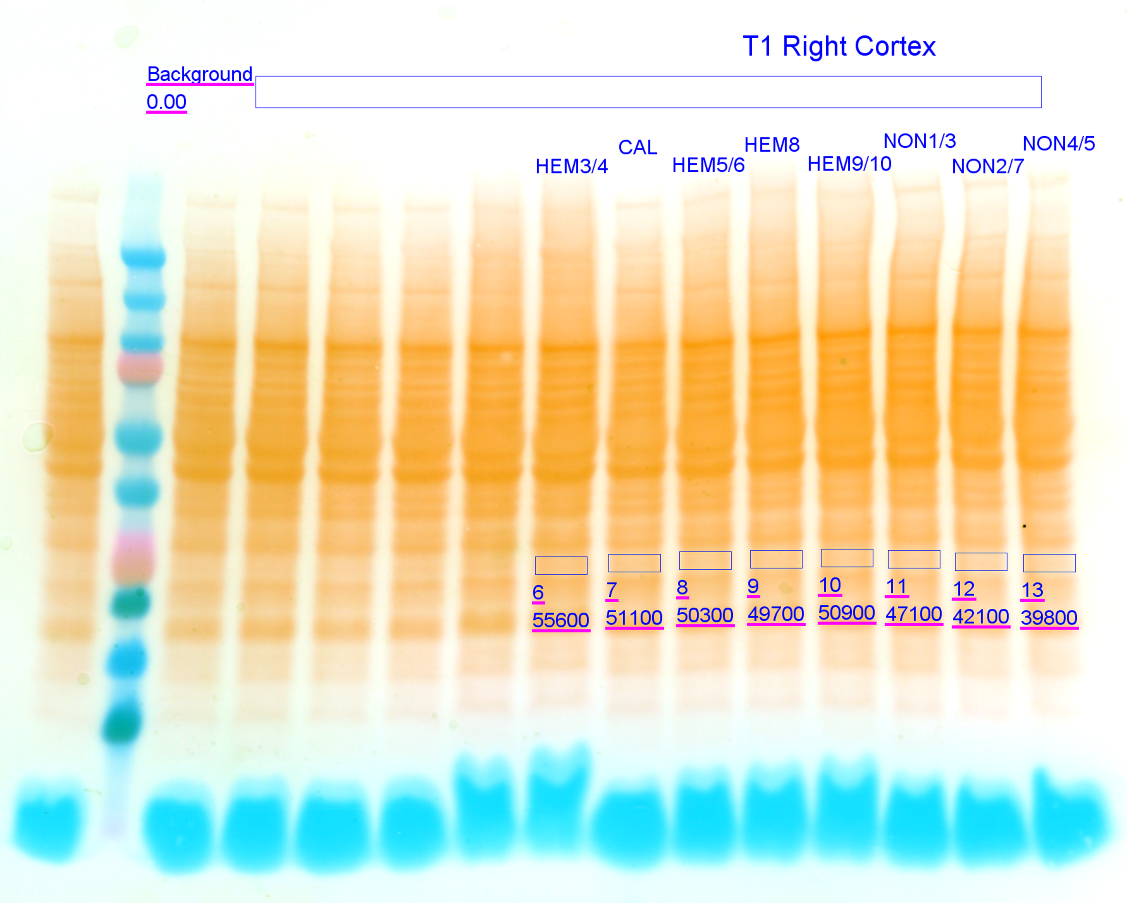


**Figure 25** Ponceau S Image of Socs3 Membrane 10


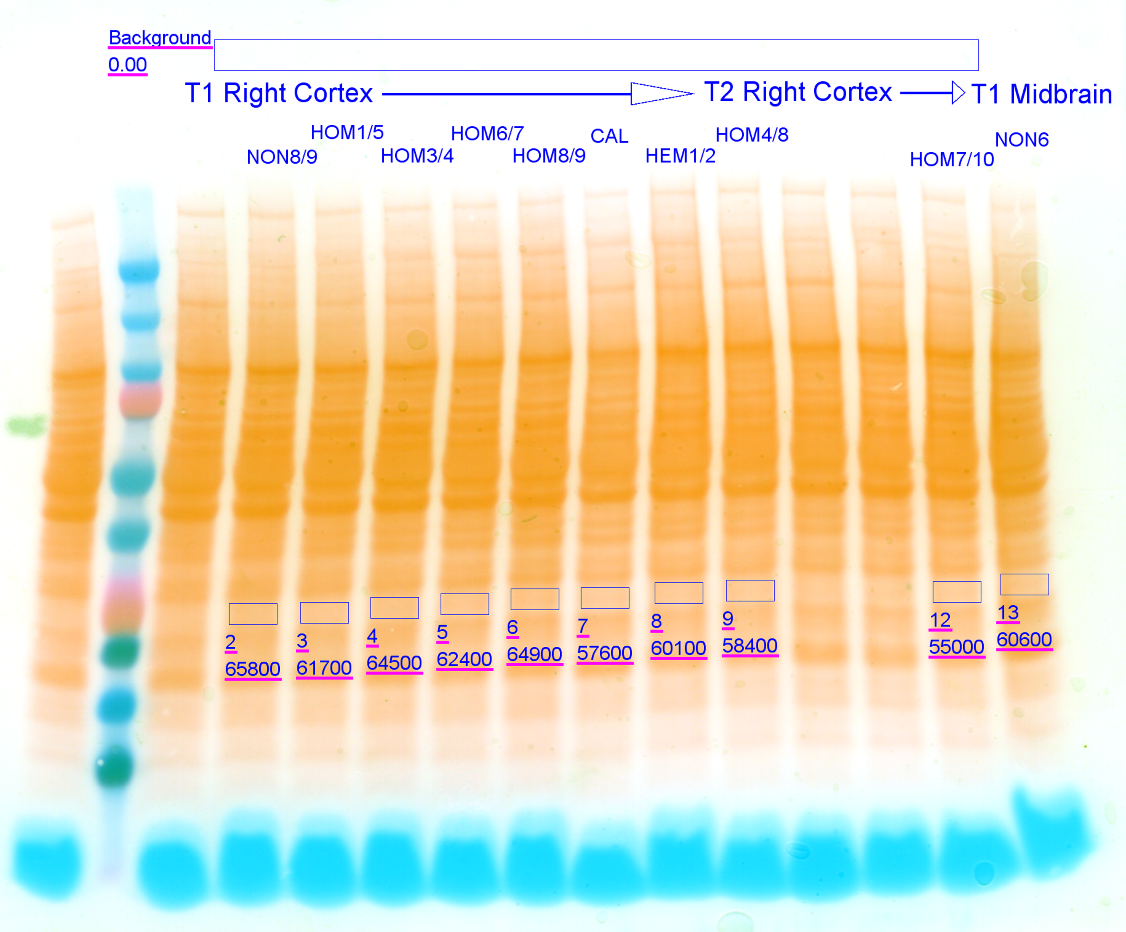


**Figure 26** Ponceau S Image of Socs3 Membrane 11


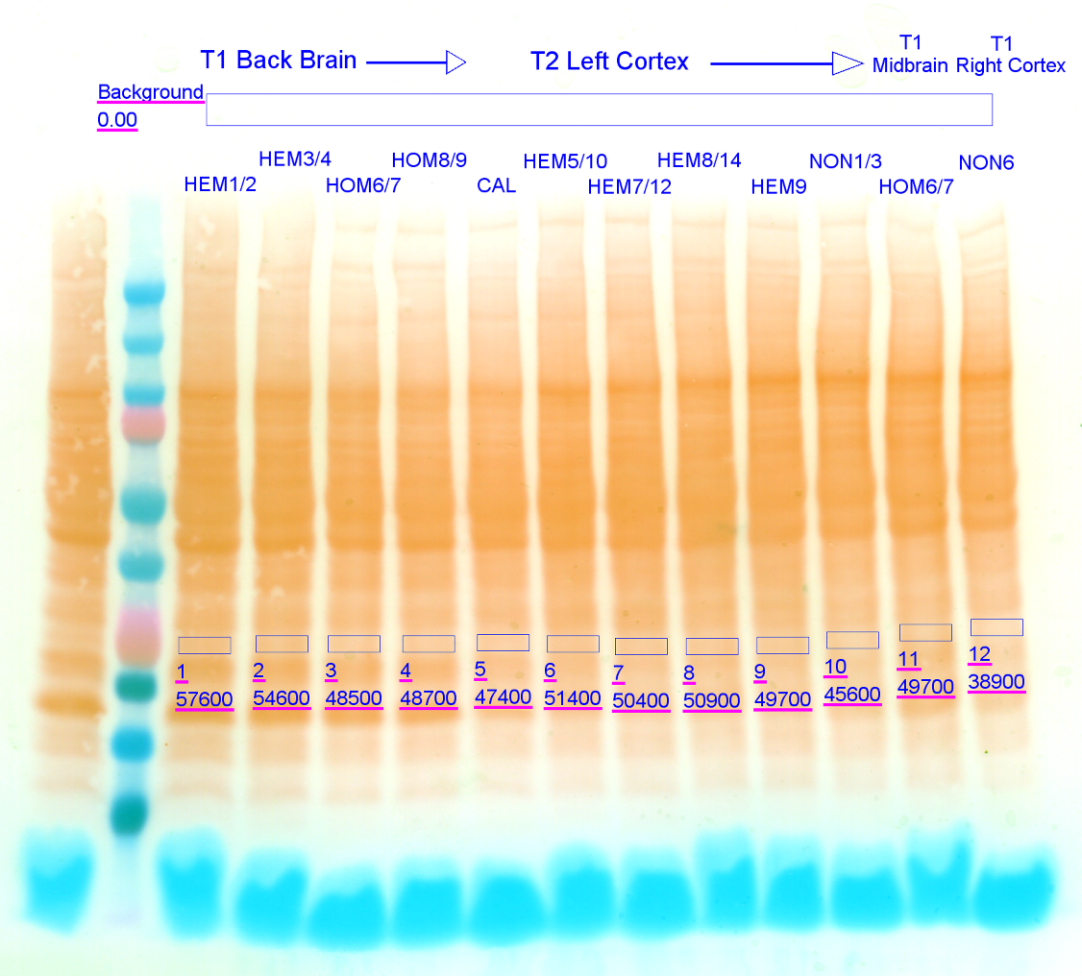


**Figure 27** Ponceau S Image of Socs3 Membrane 12


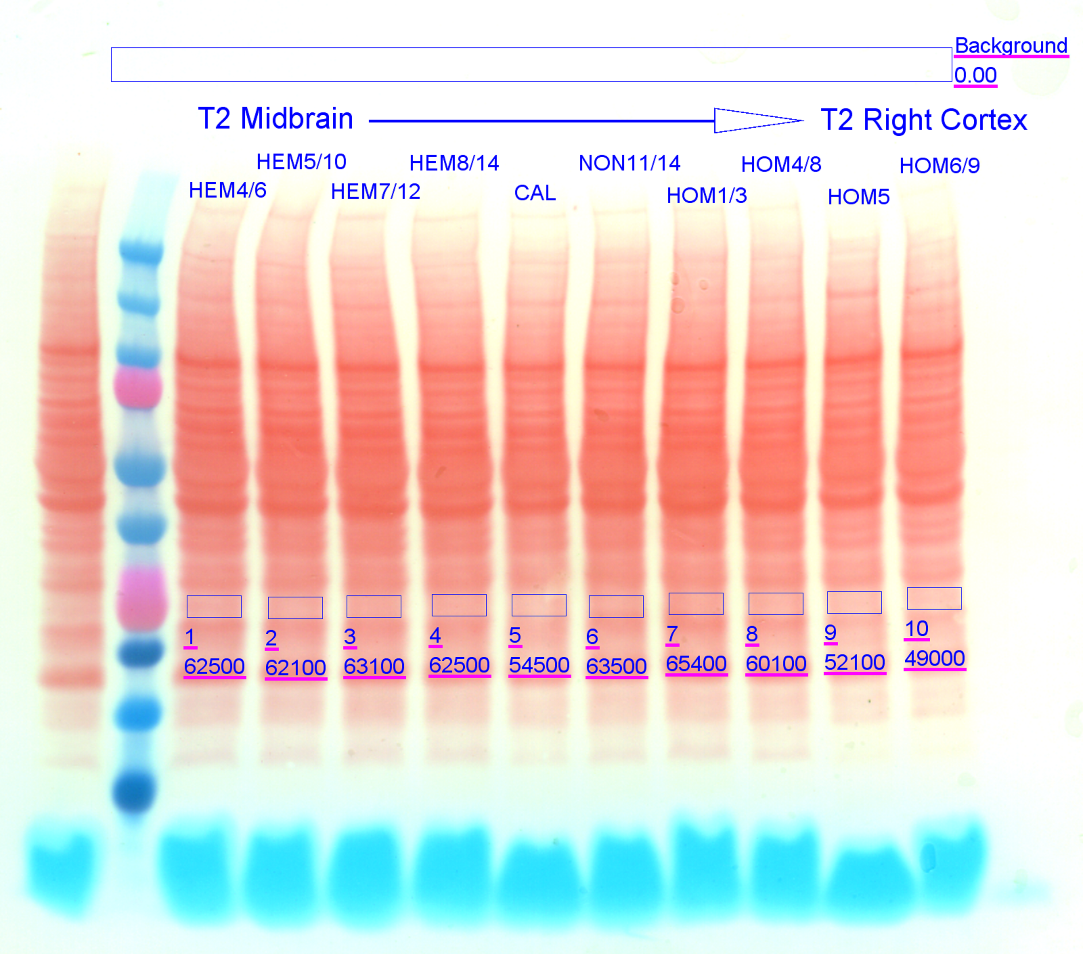


**Figure 28** Ponceau S Image of Socs3 Membrane 13

### Raw data and calibration

| **Sample** | **Signal** | **Ponceau S** | **Signal/**  **Ponceau S** | **Calibration factor** | **Calibrated signal** | **x100,000** | **Log_10_ transformed** |
| --- | --- | --- | --- | --- | --- | --- | --- |
| **Membrane 1** | | | | | | | |
| **T1 Midbrain** |  |  |  |  |  |  |  |
| HEM1/2 | 6535.8 | 76169.3 | 0.085807 |  |  | 8580.68 | 3.9335 |
| HEM3/4 | 21091.6 | 80087.3 | 0.263358 |  |  | 26335.80 | 4.4205 |
| HEM5/6 | 12214.6 | 82382.3 | 0.148268 |  |  | 14826.75 | 4.1710 |
| HEM8 | 15626.0 | 74977.3 | 0.208409 |  |  | 20840.90 | 4.3189 |
| HEM9/10 | 3858.1 | 78667.3 | 0.049044 |  |  | 4904.35 | 3.6906 |
| NON1/3 | 4844.2 | 75639.3 | 0.064044 |  |  | 6404.39 | 3.8065 |
| HOM8/9 calibrator | 4897.9 | 76074.3 | 0.064383 |  |  | 6438.32 | 3.8088 |
| NON2/7 | 5409.1 | 80991.3 | 0.066786 |  |  | 6678.59 | 3.8247 |
| NON4/5 | 5677.9 | 74399.3 | 0.07632 |  |  | 7631.68 | 3.8826 |
| HOM1/5 | 12135.4 | 74626.3 | 0.162616 |  |  | 16261.60 | 4.2112 |
| HOM3/4 | 13582.2 | 77021.3 | 0.176343 |  |  | 17634.35 | 4.2464 |
| **Membrane 1 (2)** | | | | | | | |
| calibrator | 39272.5 | 76074.3 | 0.516239 | 0.124716 | 0.064383 | 6438.32 | 3.8088 |
| NON8/9 | 39371 | 74917.3 | 0.525526 |  | 0.065541 | 6554.15 | 3.8165 |
| **Membrane 2** | | | | | | | |
| **T2 Midbrain** |  |  |  | 0.354556 |  |  |  |
| HEM9 | 3659 | 67893.3 | 0.05389 |  | 0.019108 | 1910.82 | 3.2812 |
| calibrator | 11670 | 64266.3 | 0.181588 |  | 0.064383 | 6438.32 | 3.8088 |
| NON1/3 | 31165 | 68659.3 | 0.453908 |  | 0.160936 | 16093.57 | 4.2067 |
| NON2/15 | 41207 | 67481.3 | 0.610643 |  | 0.216507 | 21650.72 | 4.3355 |
| NON4/13 | 23437 | 62963.3 | 0.37223 |  | 0.13198 | 13197.73 | 4.1205 |
| NON5/10 | 10139 | 67738.3 | 0.149679 |  | 0.053070 | 5306.96 | 3.7248 |
| NON6/9 | 11700 | 64199.3 | 0.182245 |  | 0.064616 | 6461.60 | 3.8103 |
| NON7/8 | 4336 | 56738.3 | 0.076421 |  | 0.027096 | 2709.55 | 3.4329 |
| **Membrane 3** | | | | | | | |
| HOM5 | 6112 | 55828.9 | 0.10948 | 0.05034 | 0.005511 | 551.15 | 2.7413 |
| HOM6/9 | 22099 | 56433.9 | 0.391591 |  | 0.019714 | 1971.41 | 3.2948 |
| HOM7/10 | 41638.5 | 55711.9 | 0.747390 |  | 0.037626 | 3762.63 | 3.5755 |
| calibrator | 71769 | 56118.9 | 1.278874 |  | 0.064383 | 6438.32 | 3.8088 |
| **T1 Hindbrain** |  |  |  |  |  |  |  |
| HEM5/6 | 63715 | 64487.9 | 0.988015 |  | 0.049740 | 4974.03 | 3.6967 |
| HEM8 | 74712 | 62638.9 | 1.192741 |  | 0.060046 | 6004.69 | 3.7785 |
| HEM9/10 | 46124 | 62343.9 | 0.739832 |  | 0.037245 | 3724.58 | 3.5711 |
| NON1/3 | 57825 | 62462.9 | 0.925749 |  | 0.046606 | 4660.56 | 3.6684 |
| **Membrane 4** | | | | | | | |
| NON2/7 | 35192 | 53357.2 | 0.659554 | 0.048402 | 0.031924 | 3192.36 | 3.5041 |
| NON4/5 | 41454 | 51110.0 | 0.811073 |  | 0.039257 | 3925.74 | 3.5939 |
| NON6 | 31713 | 51035.7 | 0.621389 |  | 0.030076 | 3007.63 | 3.4782 |
| NON8/9 | 29754 | 50714.4 | 0.586698 |  | 0.028397 | 2839.72 | 3.4533 |
| HOM1/5 | 24437 | 49734.5 | 0.491349 |  | 0.023782 | 2378.21 | 3.3763 |
| HOM3/4 | 67972 | 45950.2 | 1.479255 |  | 0.071599 | 7159.85 | 3.8549 |
| calibrator | 66122 | 49709.0 | 1.33018 |  | 0.064383 | 6438.32 | 3.8088 |
| **T3 Hindbrain** |  |  |  |  |  |  |  |
| HEM1 | 51703 | 46239.0 | 1.118169 |  | 0.054121 | 5412.14 | 3.7334 |
| HEM2/3 | 100708 | 47906.7 | 2.102168 |  | 0.101749 | 10174.86 | 4.0075 |
| HEM7/9 | 81129 | 48684.5 | 1.666424 |  | 0.080658 | 8065.79 | 3.9066 |
| HEM8/10 | 77432 | 45686.0 | 1.694874 |  | 0.082035 | 8203.49 | 3.9140 |
| **Membrane 5** | | | | | | | |
| HEM11/13 | 22824 | 59224.3 | 0.385382 | 0.033446 | 0.012889 | 1288.94 | 3.1102 |
| HEM12 | 24099 | 59746.6 | 0.403354 |  | 0.013490 | 1349.05 | 3.1300 |
| NON1/2 | 5144 | 60764.1 | 0.084655 |  | 0.002831 | 283.14 | 2.4520 |
| NON4/11 | 5752 | 59557.9 | 0.096578 |  | 0.003230 | 323.01 | 2.5092 |
| NON5/6 | 16231 | 58504.5 | 0.277432 |  | 0.009279 | 927.89 | 2.9675 |
| NON7 | 54466 | 57534.3 | 0.946670 |  | 0.031662 | 3166.21 | 3.5005 |
| calibrator | 128636 | 66823.9 | 1.925000 |  | 0.064383 | 6438.32 | 3.8088 |
| NON8/9 | 42279 | 60436.1 | 0.699565 |  | 0.023398 | 2339.75 | 3.3692 |
| NON12 | 7860 | 56519.0 | 0.13907 |  | 0.004651 | 465.12 | 2.6676 |
| HOM1/11 | 3490 | 58723.8 | 0.059431 |  | 0.001988 | 198.77 | 2.2984 |
| **Membrane 6** | | | | | | | |
| HOM2/7 | 28949 | 62460.2 | 0.463479 | 0.053194 | 0.024654 | 2465.44 | 3.3919 |
| HOM3/4 | 11321 | 60779.2 | 0.186264 |  | 0.009908 | 990.82 | 2.9960 |
| calibrator | 79021 | 65288.2 | 1.210341 |  | 0.064383 | 6438.32 | 3.8088 |
| **Membrane 6 (2)** | | | | | | | |
| HOM8/9 | 11359 | 58668.2 | 0.193614 | 0.165081 | 0.031962 | 3196.20 | 3.5046 |
| HOM10 | 12474 | 60079.2 | 0.20763 |  | 0.034275 | 3427.51 | 3.5350 |
| HOM12 | 10672 | 58438.2 | 0.182620 |  | 0.030147 | 3014.72 | 3.4792 |
| **T1 Left Cortex** |  |  |  |  |  |  |  |
| HEM1/2 | 17239 | 63719.2 | 0.270546 |  | 0.044662 | 4466.21 | 3.6499 |
| calibrator | 25463 | 65288.2 | 0.390009 |  | 0.064383 | 6438.32 | 3.8088 |
| HEM3/4 | 29957 | 64123.2 | 0.467179 |  | 0.077122 | 7712.24 | 3.8872 |
| HEM5/6 | 48027 | 64577.2 | 0.743714 |  | 0.122773 | 12277.32 | 4.0891 |
| HEM9/10 | 42368 | 60180.2 | 0.704019 |  | 0.116220 | 11622.02 | 4.0653 |
| NON1/3 | 53995 | 60595.2 | 0.891077 |  | 0.147100 | 14710.00 | 4.1676 |
| NON2/7 | 48406 | 60058.2 | 0.805984 |  | 0.133052 | 13305.29 | 4.1240 |
| NON4/5 | 23054 | 56343.2 | 0.409171 |  | 0.067546 | 6754.63 | 3.8296 |
| **Membrane 7** | | | | | | | |
| NON6 | 1147 | 57150.6 | 0.020070 | 0.849149 | 0.017042 | 1704.22 | 3.2315 |
| NON8/9 | 2034 | 54979.6 | 0.036996 |  | 0.031415 | 3141.47 | 3.4971 |
| HOM1/5 | 2152 | 54087.6 | 0.039787 |  | 0.03379 | 3378.53 | 3.5287 |
| HOM3/4 | 4541 | 50383.6 | 0.090128 |  | 0.076532 | 7653.25 | 3.8838 |
| HOM6/7 | 7820 | 55111.6 | 0.141894 |  | 0.120489 | 12048.89 | 4.0809 |
| HOM8/9 | 2598 | 55562.6 | 0.046758 |  | 0.039705 | 3970.45 | 3.5988 |
| calibrator | 4210 | 55525.6 | 0.075821 |  | 0.064383 | 6438.32 | 3.8088 |
| **T2 Left Cortex** |  |  |  |  |  |  |  |
| HEM4/6 | 2541 | 52186.6 | 0.048691 |  | 0.041346 | 4134.56 | 3.6164 |
| **Membrane 8** | | | | | | | |
| NON2/15 | 3621 | 59928.3 | 0.060422 | 0.059171 | 0.003575 | 357.52 | 2.5533 |
| NON4/13 | 2143 | 57170.3 | 0.037485 |  | 0.002218 | 221.80 | 2.3460 |
| NON5/10 | 7866 | 60469.3 | 0.130083 |  | 0.007697 | 769.71 | 2.8863 |
| NON6/9 | 17007 | 61293.3 | 0.277469 |  | 0.016418 | 1641.81 | 3.2153 |
| NON7/8 | 27103 | 60156.3 | 0.450543 |  | 0.026659 | 2665.91 | 3.4258 |
| NON11/14 | 53932 | 61175.3 | 0.881598 |  | 0.052165 | 5216.51 | 3.7174 |
| calibrator | 66552 | 61164.3 | 1.088086 |  | 0.064383 | 6438.32 | 3.8088 |
| HOM1/3 | 70247 | 57991.3 | 1.211337 |  | 0.071676 | 7167.61 | 3.8554 |
| HOM4/8 | 65345.5 | 56384.3 | 1.158931 |  | 0.068575 | 6857.51 | 3.8362 |
| HOM5 | 58097 | 58034.3 | 1.001081 |  | 0.059235 | 5923.50 | 3.7726 |
| HOM6/9 | 54667 | 53273.3 | 1.026162 |  | 0.060719 | 6071.90 | 3.7833 |
| HOM7/10 | 57676 | 52383.3 | 1.101038 |  | 0.065150 | 6514.96 | 3.8139 |
| **T2 Right Cortex** |  |  |  |  |  |  |  |
| HEM4/6 | 39359 | 49300.3 | 0.798352 |  | 0.047239 | 4723.93 | 3.6743 |
| **Membrane 9** | | | | | | | |
| HEM5/10 | 2257 | 51232.6 | 0.044054 | 0.470377 | 0.020722 | 2072.20 | 3.3164 |
| HEM7/12 | 632 | 49428.6 | 0.012786 |  | 0.006014 | 601.43 | 2.7792 |
| HEM8/14 | 1595 | 51434.6 | 0.031010 |  | 0.014586 | 1458.65 | 3.1640 |
| HEM9 | 3275 | 53135.6 | 0.061635 |  | 0.028992 | 2899.15 | 3.4623 |
| NON1/3 | 2235 | 50325.6 | 0.044411 |  | 0.020890 | 2088.98 | 3.3199 |
| NON2/15 | 4976 | 54340.6 | 0.091571 |  | 0.043073 | 4307.26 | 3.6342 |
| calibrator | 7315 | 53442.6 | 0.136876 |  | 0.064383 | 6438.32 | 3.8088 |
| NON4/13 | 9401 | 50100.6 | 0.187642 |  | 0.088263 | 8826.26 | 3.9458 |
| NON5/10 | 3088 | 50078.6 | 0.061663 |  | 0.029005 | 2900.49 | 3.4625 |
| NON6/9 | 3485 | 50254.6 | 0.069347 |  | 0.032619 | 3261.91 | 3.5135 |
| NON7/8 | 9240 | 49998.6 | 0.184805 |  | 0.086928 | 8692.80 | 3.9392 |
| NON11/14 | 13381 | 46929.6 | 0.285129 |  | 0.134118 | 13411.81 | 4.1275 |
| HOM1/3 | 15968 | 40100.6 | 0.398198 |  | 0.187303 | 18730.32 | 4.2725 |
| **Membrane 10** | | | | | | | |
| **T1 Right Cortex** |  |  |  |  |  |  |  |
| HEM3/4 | 5291 | 55585.3 | 0.095187 | 0.89762 | 0.085442 | 8544.20 | 3.9317 |
| calibrator | 3663 | 51069.1 | 0.071726 |  | 0.064383 | 6438.32 | 3.8088 |
| HEM5/6 | 10507 | 50338.5 | 0.208727 |  | 0.187358 | 18735.78 | 4.2727 |
| HEM8 | 2937 | 49722.9 | 0.059067 |  | 0.053020 | 5302.02 | 3.7244 |
| HEM9/10 | 7372 | 50911.2 | 0.144801 |  | 0.129977 | 12997.67 | 4.1139 |
| NON1/3 | 16115 | 47092.0 | 0.342202 |  | 0.307168 | 30716.81 | 4.4874 |
| NON2/7 | 8126 | 42114.8 | 0.192949 |  | 0.173195 | 17319.51 | 4.2385 |
| NON4/5 | 23886 | 39786.9 | 0.600349 |  | 0.538886 | 53888.60 | 4.7315 |
| **Membrane 11** | | | | | | | |
| NON8/9 | 16206 | 65782.1 | 0.246359 | 0.521228 | 0.128409 | 12840.90 | 4.1086 |
| HOM1/5 | 18434 | 61732.1 | 0.298613 |  | 0.155645 | 15564.53 | 4.1921 |
| HOM3/4 | 22296 | 64502.1 | 0.345663 |  | 0.180169 | 18016.92 | 4.2557 |
| HOM6/7 | 19682 | 62407.1 | 0.315381 |  | 0.164385 | 16438.52 | 4.2159 |
| HOM8/9 | 15796 | 64939.1 | 0.243243 |  | 0.126785 | 12678.51 | 4.1031 |
| calibrator | 7116 | 57609.1 | 0.123522 |  | 0.064383 | 6438.32 | 3.8088 |
| HEM1/2 | 27409 | 60112.1 | 0.455965 |  | 0.237661 | 23766.15 | 4.3760 |
| **T2 Right Cortex** |  |  |  |  |  |  |  |
| HOM4/8 | 16641 | 58370.1 | 0.285095 |  | 0.148599 | 14859.92 | 4.1720 |
| HOM7/10 | 13644 | 55031.1 | 0.247933 |  | 0.129229 | 12922.93 | 4.1114 |
| **T1 Midbrain** |  |  |  |  |  |  |  |
| NON6 | 12865 | 60648.1 | 0.212125 |  | 0.110566 | 11056.56 | 4.0436 |
| **Membrane 12** | | | | | | | |
| **T1 Hindbrain** |  |  |  |  |  |  |  |
| HEM1/2 | 15850 | 57581.2 | 0.275264 | 0.323033 | 0.088919 | 8891.94 | 3.9490 |
| HEM3/4 | 13243 | 54603.2 | 0.242532 |  | 0.078346 | 7834.59 | 3.8940 |
| HOM6/7 | 14683 | 48498.2 | 0.302754 |  | 0.097800 | 9779.96 | 3.9903 |
| HOM8/9 | 14728 | 48725.2 | 0.302267 |  | 0.097642 | 9764.23 | 3.9896 |
| calibrator | 9457 | 47449.2 | 0.199308 |  | 0.064383 | 6438.32 | 3.8088 |
| **T2 Left Cortex** |  |  |  |  |  |  |  |
| HEM5/10 | 6355 | 51399.2 | 0.123640 |  | 0.039940 | 3993.99 | 3.6014 |
| HEM7/12 | 10899 | 50360.2 | 0.216421 |  | 0.069911 | 6991.12 | 3.8445 |
| HEM8/14 | 8201 | 50899.2 | 0.161122 |  | 0.052048 | 5204.80 | 3.7164 |
| HEM9 | 10766 | 49699.2 | 0.216623 |  | 0.069977 | 6997.66 | 3.8450 |
| NON1/3 | 3057 | 45614.2 | 0.067019 |  | 0.021649 | 2164.93 | 3.3354 |
| **T1 Midbrain** |  |  |  |  |  |  |  |
| HOM6/7 | 7678 | 49696.2 | 0.154499 |  | 0.049908 | 4990.83 | 3.6981 |
| **T1 Right Cortex** |  |  |  |  |  |  |  |
| NON6 | 11178 | 38857.2 | 0.287669 |  | 0.092927 | 9292.67 | 3.9681 |
| **Membrane 13** | | | | | | | |
| **T2 Midbrain** |  |  |  |  |  |  |  |
| HEM4/6 | 9597 | 62514.4 | 0.153517 | 0.457659 | 0.070258 | 7025.83 | 3.8467 |
| HEM5/10 | 8182 | 62134.4 | 0.131682 |  | 0.060266 | 6026.56 | 3.7801 |
| HEM7/12 | 10143 | 63094.4 | 0.160759 |  | 0.073573 | 7357.29 | 3.8667 |
| HEM8/14 | 8439 | 62521.4 | 0.134978 |  | 0.061774 | 6177.38 | 3.7908 |
| calibrator | 7663 | 54471.4 | 0.140679 |  | 0.064383 | 6438.32 | 3.8088 |
| NON11/14 | 9841 | 63469.4 | 0.155051 |  | 0.070961 | 7096.05 | 3.8510 |
| HOM1/3 | 13292 | 65405.4 | 0.203225 |  | 0.093008 | 9300.77 | 3.9685 |
| HOM4/8 | 22381 | 60051.4 | 0.372697 |  | 0.170568 | 17056.83 | 4.2319 |
| **T2 Right Cortex** |  |  |  |  |  |  |  |
| HOM5 | 14864 | 52132.4 | 0.285120 |  | 0.130488 | 13048.78 | 4.1156 |
| HOM6/9 | 6802 | 48997.4 | 0.138824 |  | 0.063534 | 6353.39 | 3.8030 |

**Table 1** Data from Socs3 Western blot membrane images. The calibrated signal, multiplied by 100,000 to reduce the number of decimal places, was used for calculating the mean of each group. The log_10_ transformation was used for statistical analysis.

## Stat3 phosphorylated at tyrosine705

### Characterisation

The dilution of the anti-Stat3 phospho Y705 antibody (ab76315, Abcam) recommended by the manufacturer for Western blot is 1/2000 - 1/20,000. This antibody was first trialled at a dilution of 1/20,000 using lysates from L44 mouse brain samples. No bands were visible using this dilution. We then tested this antibody at dilutions of 1/10,000 and 1/1000. Again, no bands were visible at the 1/10,000 dilution, however three of four samples tested showed bands at ~80 kDa using the 1/1000 dilution. We further tested this antibody at dilutions of 1/200 and 1/500, both of which showed positive results in our samples. We continued using a dilution of 1/500 (2.152 µg/mL) for the following Western blots.

We tested the antibody with a blocking peptide (Stat3 phospho Y705 peptide (ab179551, Abcam)). A mixture of 1/500 primary antibody with 1/46.5 blocking peptide was incubated at room temperature for 30 minutes before incubation with the membrane. The intensity of the protein bands at ~80 kDa faded with addition of the peptide, confirming specificity.

Samples also stained with bands below 150 kDa, below 75 kDa and at 25 kDa. The intensity of the two larger molecular weight bands also faded when the blocking peptide was added. The identity of these bands is unknown. The 25 kDa did not fade with addition of the blocking peptide, so this is likely nonspecific antibody staining.

### Full blot images

Full images of the Western blot membranes are presented below. A negative control (no primary antibody incubation) was run on the left of the ladder and a calibrator sample was run on each gel.


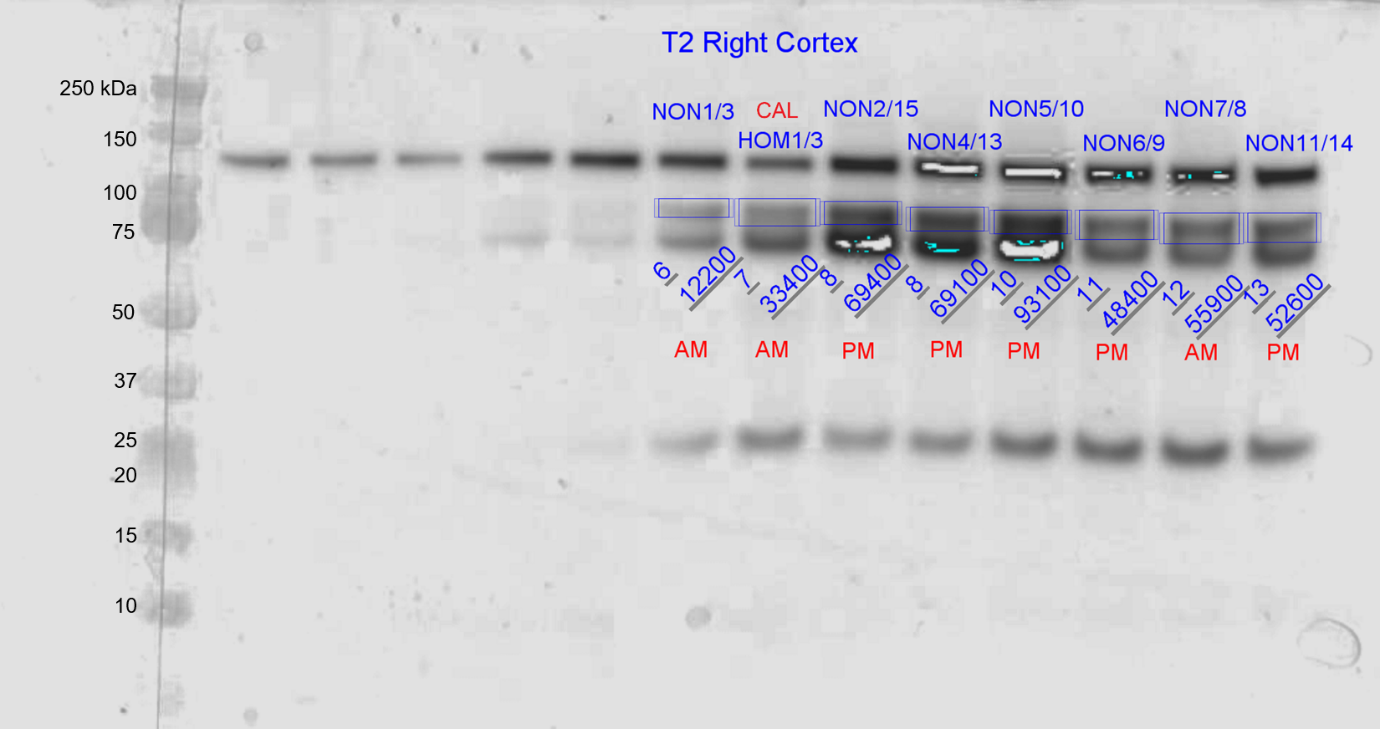


**Figure 29** pStat3 intensity of membrane 1. Exposure time: 15.41 minutes. Annotation (top to bottom): sample ID, lane number on gel, band intensity (before calibration) and collection time. CAL = calibrator sample. All following membrane images are presented in this way


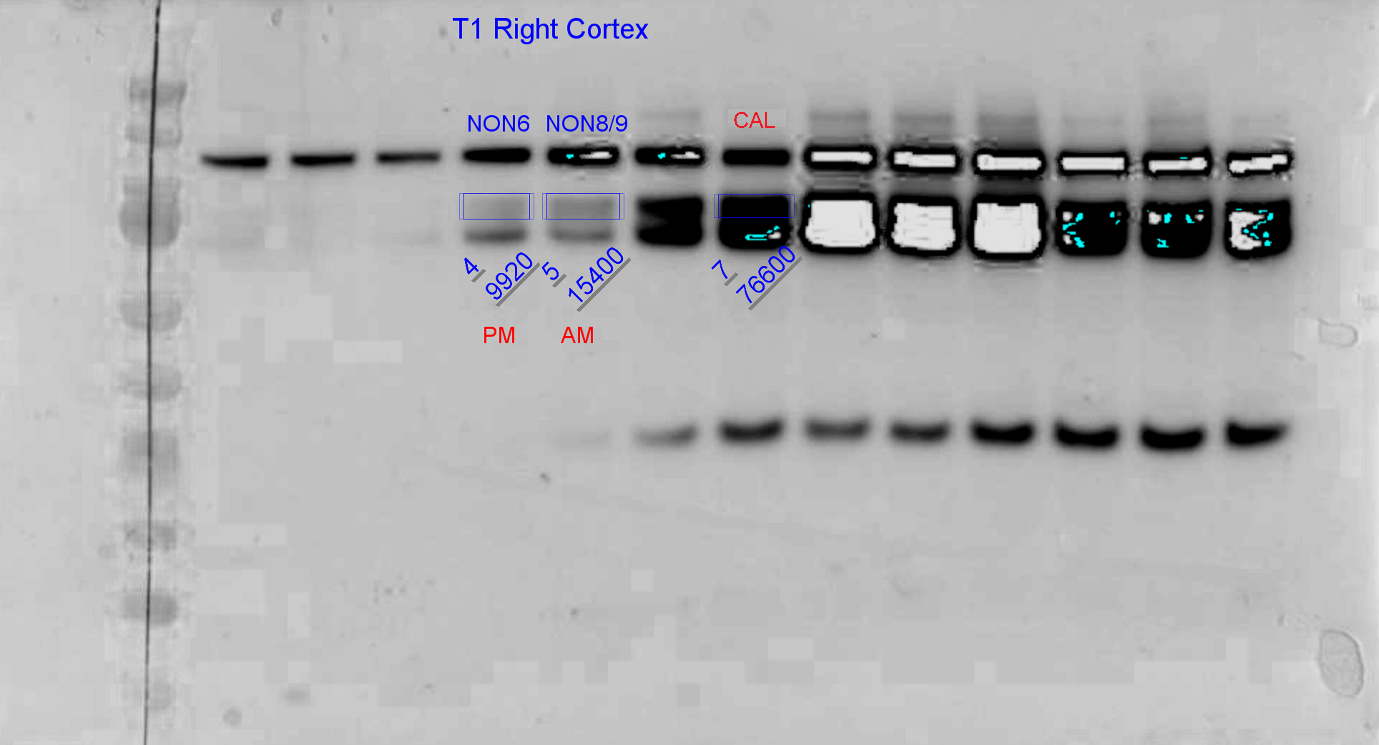


**Figure 30** pStat3 intensity of membrane 1 (second exposure). Exposure time: 25 minutes


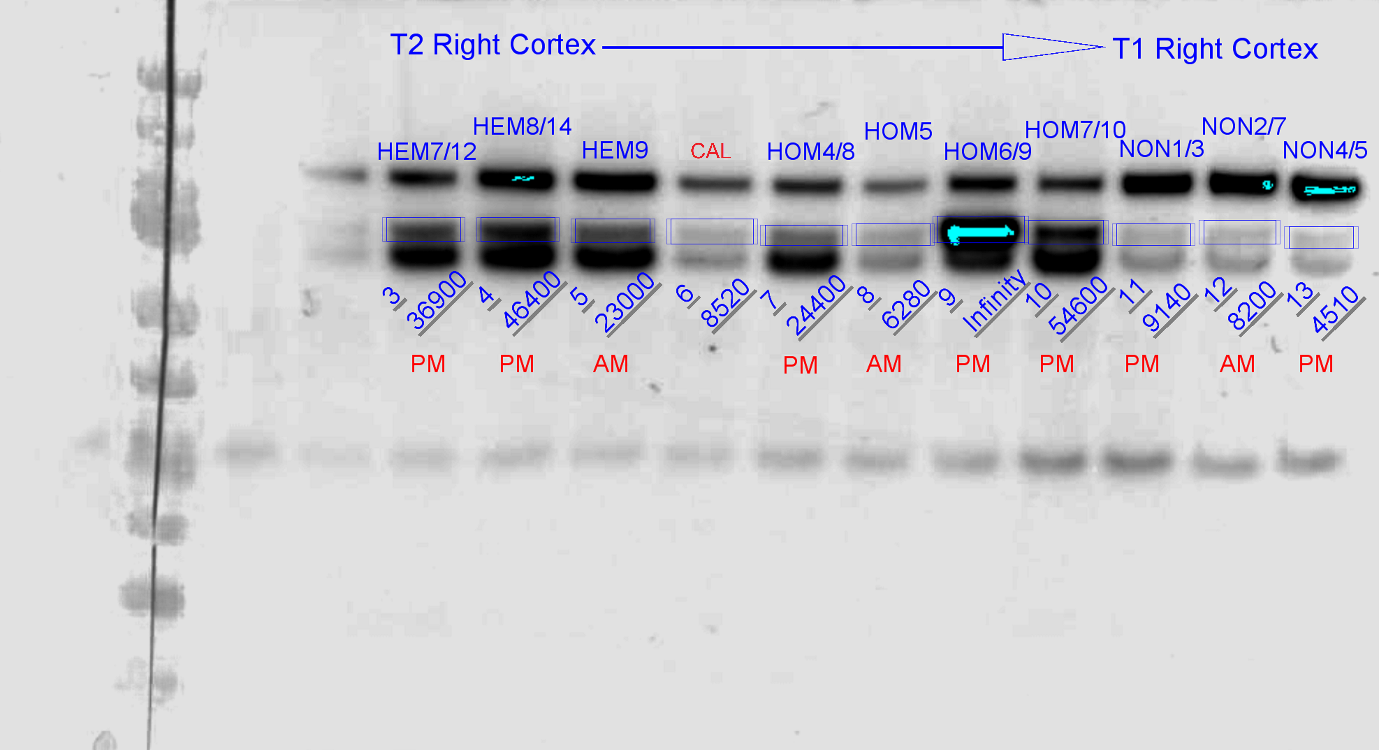


**Figure 31** pStat3 intensity of membrane 2. Exposure time: 2.48.8 minutes


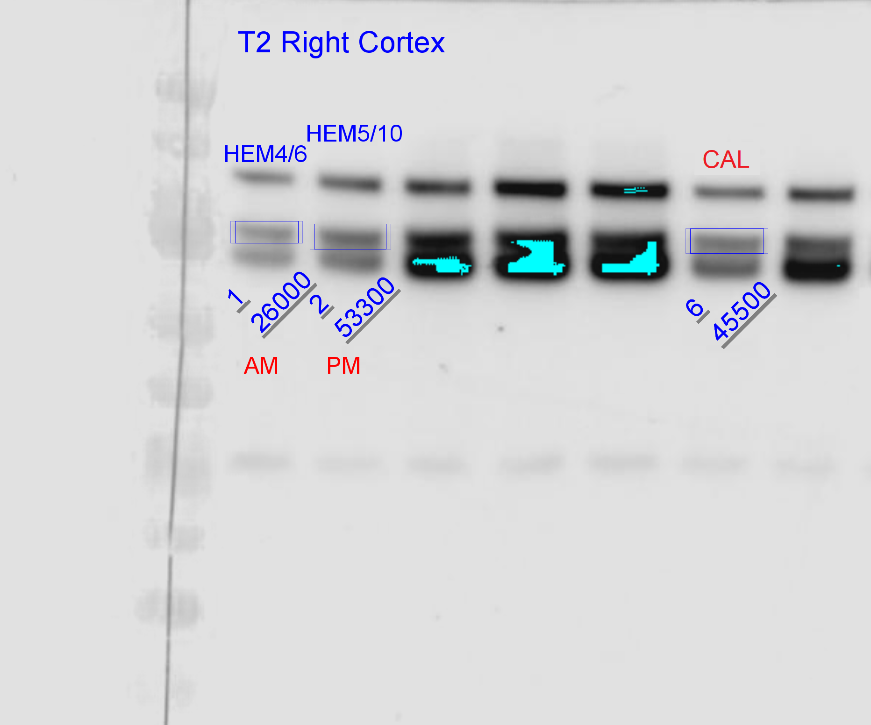


**Figure 32** pStat3 intensity of membrane 2 (second exposure). Exposure time: 6.3.3 minutes. Part of this blot has been cut and removed to avoid oversaturation from those bands on this exposure

### Ponceau S images

Densitometric analysis of a section of protein at ~75 kDa on the Ponceau S stained membrane was used for total protein normalisation. An unstained section of membrane representative of the background was used for background subtraction.

**
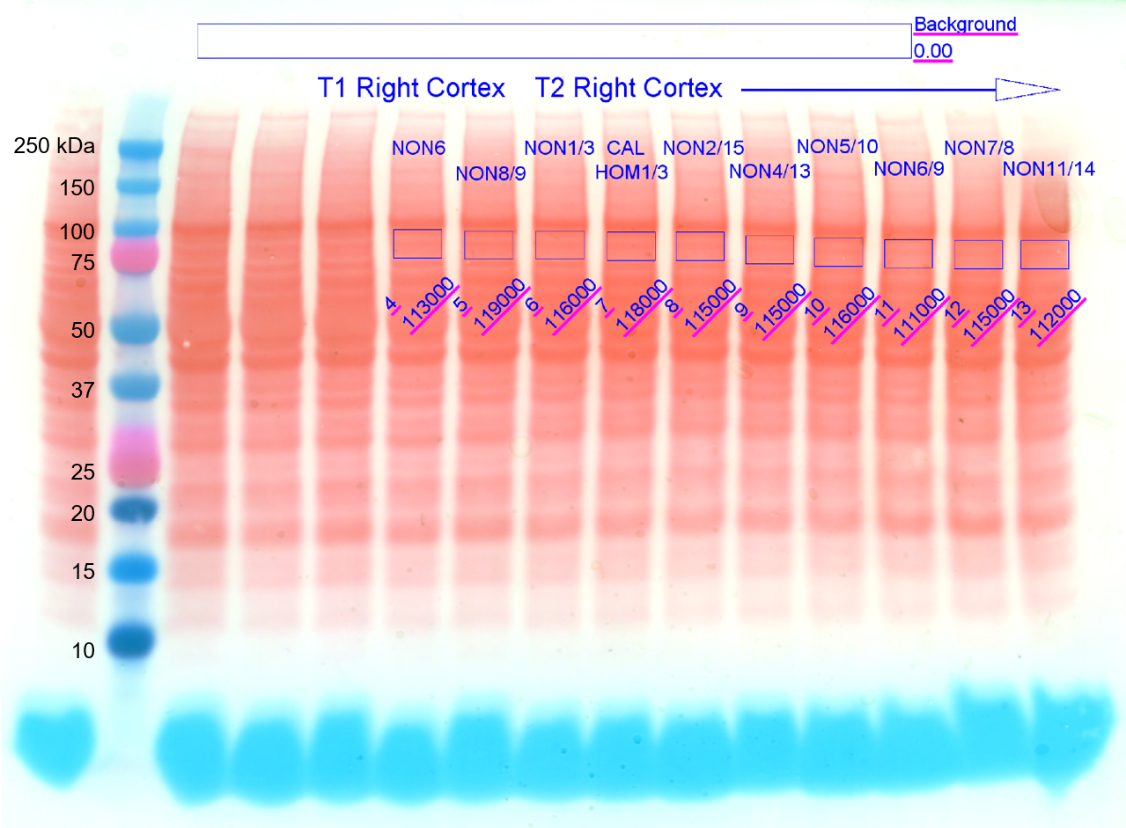
**

**Figure 33** Ponceau S Image of pStat3 Membrane 1


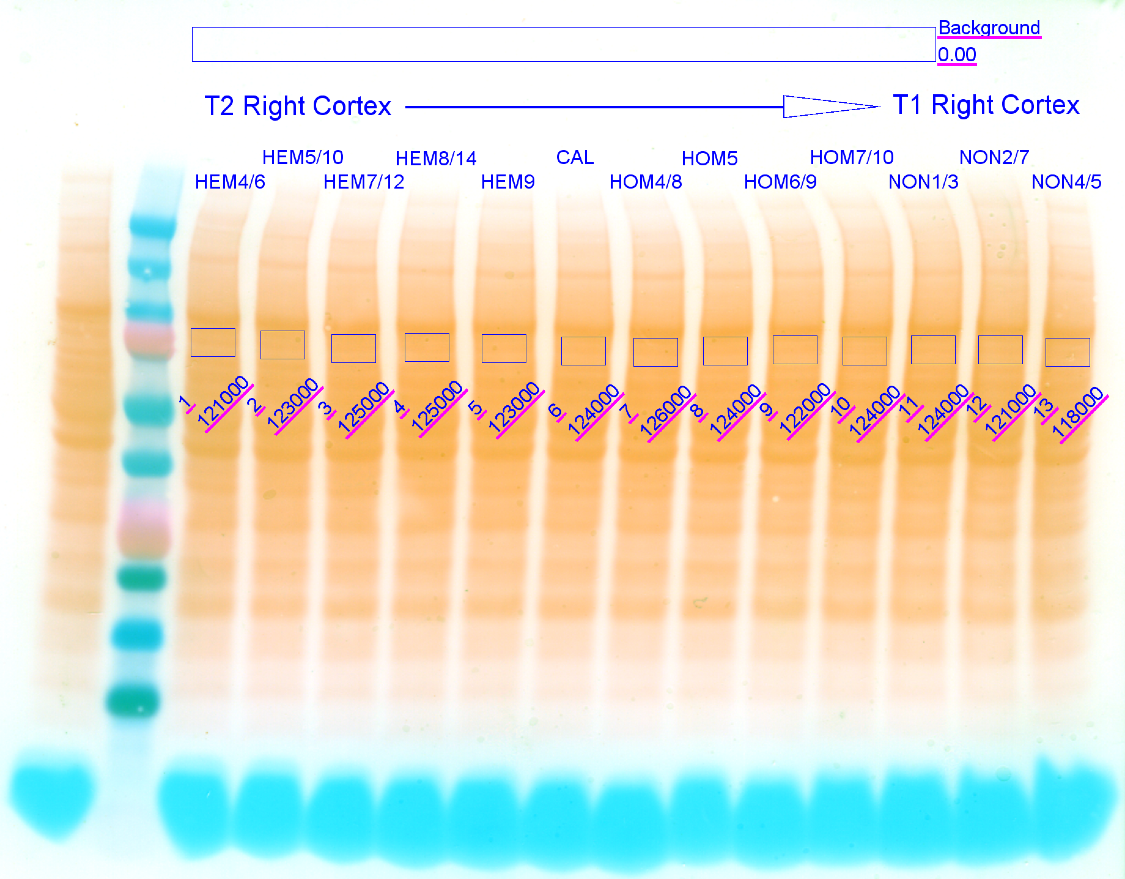


**Figure 34** Ponceau S Image of pStat3 Membrane 2

### Raw data and calibration

| **Sample** | **Signal** | **Ponceau S** | **Signal/**  **Ponceau S** | **Calibration factor** | **Calibrated signal** | **x1000** |
| --- | --- | --- | --- | --- | --- | --- |
| **Membrane 1** | | | | | | |
| **T2 Right Cortex** |  |  |  |  |  |  |
| NON1/3 | 12221 | 116403.5 | 0.104988 |  | 0.104988 | 104.99 |
| HOM1/3 calibrator | 33350.5 | 118281.0 | 0.281960 |  | 0.281960 | 281.96 |
| NON2/15 | 69377 | 115237.0 | 0.602037 |  | 0.602037 | 602.04 |
| NON4/13 | 69137 | 114908.4 | 0.601671 |  | 0.601671 | 601.67 |
| NON5/10 | 93064 | 115677.9 | 0.804510 |  | 0.804510 | 804.51 |
| NON6/9 | 48368 | 110747.6 | 0.436742 |  | 0.436742 | 436.74 |
| NON7/8 | 55894 | 114806.0 | 0.486856 |  | 0.486856 | 486.86 |
| NON1/14 | 52613 | 111856.0 | 0.470364 |  | 0.470363 | 470.36 |
| **Membrane 1 (2)** | | | | | | |
| **T1 Right Cortex** |  |  |  |  |  |  |
| NON6 | 9920 | 113292.8 | 0.087561 | 0.435249 | 0.038110 | 38.11 |
| NON8/9 | 15389 | 118514.1 | 0.129849 |  | 0.056516 | 56.52 |
| calibrator | 76624 | 118281.0 | 0.647813 |  | 0.281960 | 281.96 |
| **Membrane 2** | | | | | | |
| **T2 Right Cortex** |  |  |  |  |  |  |
| HEM7/12 | 36871 | 125201.7 | 0.294493 | 4.094926 | 1.205926 | 1205.93 |
| HEM8/14 | 46428 | 125293.7 | 0.370553 |  | 1.517388 | 1517.39 |
| HEM9 | 23026 | 122998.7 | 0.187205 |  | 0.766591 | 766.59 |
| calibrator | 8524 | 123794.7 | 0.068856 |  | 0.281960 | 281.96 |
| HOM4/8 | 24384 | 126129.7 | 0.193325 |  | 0.791651 | 791.65 |
| HOM5 | 6275 | 123659.7 | 0.050744 |  | 0.207793 | 207.79 |
| HOM6/9 | infinity | 121653.7 |  | | | |
| HOM7/10 | 54632 | 123671.7 | 0.441750 | 4.094926 | 1.808934 | 1808.93 |
| **T1 Right Cortex** |  |  |  |  |  |  |
| NON1/3 | 9141 | 124303.7 | 0.073538 |  | 0.301131 | 301.13 |
| NON2/7 | 8195 | 121140.7 | 0.067649 |  | 0.277016 | 277.01 |
| NON4/5 | 4511 | 117983.7 | 0.038234 |  | 0.156566 | 156.57 |
| **Membrane 2 (2)** | | | | | | |
| **T2 Right Cortex** |  |  |  |  |  |  |
| HEM4/6 | 26047 | 121102.7 | 0.215081 | 0.766843 | 0.164934 | 164.93 |
| HEM5/10 | 53279.5 | 123459.7 | 0.431553 |  | 0.330934 | 330.93 |
| calibrator | 45518 | 123794.7 | 0.367689 |  | 0.281960 | 281.96 |

**Table 2** Data from pStat3 Western blot membrane images. The calibrated signal, multiplied by 1000 to reduce the number of decimal places, was used for calculating the mean of each group.
